# Transcriptomic profiles in major depressive disorder: the role of immunometabolic and cell-cycle-related pathways in depression with different levels of inflammation

**DOI:** 10.1038/s41380-024-02736-w

**Published:** 2024-09-13

**Authors:** Luca Sforzini, Moira Marizzoni, Chiara Bottanelli, Veronika Kunšteková, Valentina Zonca, Samantha Saleri, Melisa Kose, Giulia Lombardo, Nicole Mariani, Maria A. Nettis, Naghmeh Nikkheslat, Courtney Worrell, Zuzanna Zajkowska, Linda Pointon, Philip J. Cowen, Jonathan Cavanagh, Neil A. Harrison, Marco A. Riva, Valeria Mondelli, Edward T. Bullmore, the Neuroimmunology of Mood Disorders and Alzheimer’s Disease (NIMA) Consortium, Annamaria Cattaneo, Carmine M. Pariante

**Affiliations:** 1 Institute of Psychiatry, Psychology and Neuroscience, Department of Psychological Medicine, Maurice Wohl Clinical Neuroscience Institute, King’s College London, London, SE5 9RT UK; 2 National Institute for Health Research (NIHR) Maudsley Biomedical Research Centre at South London and Maudsley NHS Foundation Trust and King’s College London, London, UK; 3 Biological Psychiatric Unit, IRCCS Istituto Centro San Giovanni di Dio Fatebenefratelli, 25125 Brescia, Italy; 4 Department of Pharmacological and Biomolecular Sciences, University of Milan, Milan, 20133 Italy; 5 Institute of Biology, Faculty of Medicine, Slovak Medical University, Limbova 14, 833 03 Bratislava, Slovakia; 6 Institute of Molecular Biomedicine, Faculty of Medicine, Comenius University, Sasinkova 4, 811 08 Bratislava, Slovakia; 7 Department of Psychiatry, School of Clinical Medicine, University of Cambridge, Cambridge, CB2 0SZ UK; 8 University of Oxford Department of Psychiatry, Warneford Hospital, Oxford, OX3 7JX UK; 9 Centre for Immunobiology, School of Infection & Immunity, University of Glasgow, Glasgow, G12 8TF UK; 10 School of Medicine, School of Psychology, Cardiff University Brain Research Imaging Centre, Maindy Road, Cardiff, CF24 4HQ UK

**Keywords:** Depression, Molecular biology

## Abstract

Transcriptomic profiles are important indicators for molecular mechanisms and pathways involved in major depressive disorder (MDD) and its different phenotypes, such as immunometabolic depression. We performed whole-transcriptome and pathway analyses on 139 individuals from the observational, case-control, BIOmarkers in DEPression (BIODEP) study, 105 with MDD and 34 controls. We divided MDD participants based on levels of inflammation, as measured by serum high-sensitivity C-reactive protein (CRP), in *n* = 39 ‘not inflamed’ (CRP < 1 mg/L), *n* = 31 with ‘elevated CRP’ (1–3 mg/L), and *n* = 35 with ‘low-grade inflammation’ (>3 mg/L). We performed whole-blood RNA sequencing using Illumina NextSeq 550 and statistical analyses with the Deseq2 package for R statistics (RUV-corrected) and subsequent pathway analyses with Ingenuity Pathway Analysis. Immunometabolic pathways were activated in individuals with CRP > 1 mg/L, although surprisingly the CRP 1–3 group showed stronger immune activation than the CRP > 3 group. The main pathways identified in the comparison between CRP < 1 group and controls were cell-cycle-related, which may be protective against immunometabolic abnormalities in this ‘non-inflamed’ depressed group. We further divided MDD participants based on exposure and response to antidepressants (*n* = 47 non-responders, *n* = 37 responders, and *n* = 22 unmedicated), and identified specific immunomodulatory and neuroprotective pathways in responders (especially vs. non-responders), which could be relevant to treatment response. In further subgroup analyses, we found that the specific transcriptional profile of responders is independent of CRP levels, and that the inhibition of cell-cycle-related pathways in MDD with CRP < 1 mg/L is present only in those who are currently depressed, and not in the responders. The present study demonstrates immunometabolic and cell-cycle-related transcriptomic pathways associated with MDD and different (CRP-based and treatment-based) MDD phenotypes, while shedding light on potential molecular mechanisms that could prevent or facilitate an individual’s trajectory toward immunometabolic depression and/or treatment-non-responsive depression. The recognition and integration of these mechanisms will facilitate a precision-medicine approach in MDD.

## Introduction

Individuals diagnosed with major depressive disorder (MDD) exhibit significant biological and molecular heterogeneity, which could ultimately influence the overall clinical presentation and the response to treatments [1]. An extensive body of research has been focussing on the role of the immune system and immune-related mechanisms in MDD [2]. Individuals with MDD consistently exhibit higher levels of inflammation compared with non-depressed controls [3]. This immune activation is particularly evident in individuals with MDD non-responsive to conventional treatments [4, 5] and could represent a promising target for treatment [6, 7]. Moreover, as metabolic dysfunctions frequently occur together in the context of immune-related MDD, shared immunometabolic biological pathways could explain the considerable comorbidity between depression and cardiometabolic conditions, such as obesity, metabolic syndrome, or diabetes, and represent a potential target for intervention [8].

Most of the published research in the immunopsychiatry field focuses on C-reactive protein (CRP), an acute-phase protein synthesised by hepatocytes in the liver during inflammation, mainly in response to interleukin (IL)-6 secreted by macrophages and T cells [9]. CRP is widely used in clinical and research settings, as it is easily detected in the blood and reflects both peripheral and central inflammation [10]. According to the “Centres for Disease Control and Prevention” and the “American Heart Association”, values of serum or plasma CRP above 3 mg/L identify a high-risk population for cardiovascular diseases, while values below 1 mg/L are considered normal, therefore identifying a low cardiovascular risk [11]. Based on this notion, people with CRP > 3 mg/L are usually defined as having ‘low-grade inflammation’, and those with 1–3 mg/L as having ‘elevated CRP’ [12]. Compelling evidence demonstrates that individuals with MDD have increased levels of CRP compared with non-depressed controls [12–14], even after adjusting for multiple confounders. Also, there is evidence from clinical trials that different anti-inflammatory medications are effective in reducing depressive symptoms, but only in MDD individuals with CRP levels >3–5 mg/L [15, 16]. Of course, CRP represents a distal outcome of the pathogenesis process related to immune activation, and it is influenced by a variety of clinical and sociodemographic factors [5, 17], such as weight, body mass index (BMI), and lipid profile [9].

An alternative approach to move closer to immune ‘causal mechanisms’ involves examining upstream the protein expression process and studying the transcriptional modifications captured with mRNA expression levels, in the step linking genes (and genotypes) with proteins (and phenotypes) [18]. Gene expression studies are particularly relevant to MDD research, and mRNA levels have frequently been used as markers of the underlying molecular mechanisms and biological processes [19]. Indeed, we have previously demonstrated, using a whole-blood mRNA, qPCR-based, candidate approach, that the molecular immune profiles of depressed patients are not fully captured by CRP levels, with evidence of immune activation even in patients with CRP < 1 mg/L [20]. However, this candidate mRNA approach focused on a limited number of individual pre-selected transcripts. Hypotheses-free approaches, such as RNA sequencing (RNA-seq) are not limited by an a priori knowledge and can be used to investigate the entire transcriptome [21].

We aim to expand upon our previous findings in a new, partially-overlapping sample of MDD patients and healthy controls, by examining the whole-transcriptome whole-blood mRNA expression in CRP-based (<1, 1–3 and. >3 mg/L) and treatment-based (responders, non-responders and unmedicated) groups, followed by pathway analyses. Previous transcriptomic research in people with depression unselected for the inflammatory status identified immune-related pathways as differentiating between cases and controls [22–26]. The identification of mechanistic pathways associated with MDD phenotypes selected based on CRP levels might contribute to addressing the major challenge and urgent need for a precision medicine approach in the management and treatment of MDD. To our knowledge, this is the first study to analyse whole-transcriptome mRNA expression in people with MDD stratified based on CRP levels. Of note, a previous study in another partially-overlapping sample measured the transcriptomic profile using RNA-seq in peripheral blood mononuclear cells (PBMCs) and found no evidence for a differential expression signature when comparing MDD and controls [27]. However, a recent meta-analysis has found that studies measuring differential expression in whole-blood mRNA are more consistent than studies based on PBMCs mRNA [24]. Thus, we have used whole-blood mRNA in this study.

## Methods

### Study design and sample characteristics

We analysed data from the multicentre, non-interventional, case-control BIOmarkers in DEPression (BIODEP) study. More detailed information on the study design, clinical assessments, and inclusion and exclusion criteria have been previously published [5, 20, 28] and are presented in the Supplementary Methods in the Appendix.

In our main mRNA analyses, we examined samples from 139 participants, 105 with MDD and 34 controls. Of these, only around 30–35% of depressed patients (and no healthy controls) were also analysed in the previously mentioned mRNA studies [20, 27]. We divided MDD participants based on their levels of serum CRP ( < 1, 1–3, >3 mg/L), as in other previous papers [11, 12, 20]. We further analysed the mRNA signatures associated with exposure and response to antidepressant treatments, by dividing MDD participants into responders, non-responders, and unmedicated: MDD responders had a HAM-D17 score <7 (*not currently depressed*), while on antidepressant medication(s) at standard dosage for at least 6 weeks; MDD non-responders had a HAM-D17 score >13 (*currently depressed*) while on antidepressant medication(s) at a standard therapeutic dose; MDD unmedicated had a HAM-D17 score >13 (*currently depressed*), they had not been treated with any antidepressant medication and had at least one historical failure to a different antidepressant (see also Supplementary Table S1 in the Appendix).

Descriptive clinical and sociodemographic characteristics of the CRP-based sample are reported in Table 1. As expected by design, serum CRP values were different between the different groups, with higher mean values in the CRP 1–3 and >3 groups compared with the others, but not between CRP < 1 mg/L and controls. Also, as expected, mean BMI followed the values of serum CRP, and was significantly higher in CRP > 3 mg/L (around 30 Kg/m^2^) compared with both CRP < 1 and controls (both around 25 Kg/m^2^), and, similarly, in CRP 1–3 (around 28 Kg/m^2^) compared again with both CRP < 1 and controls. Of note, there were no significant differences within the CRP-based groups in the exposure and response to treatments.

**Table 1:** Clinical and sociodemographic﻿ characteristics.

	MDD serum hsCRP < 1 mg/L *n* = 39	MDD serum hsCRP 1–3 mg/L *n* = 31	MDD serum hsCRP > 3 mg/L *n* = 35	Controls *n* = 34	Group tests (Statistics and *p* values) *and post-hoc analyses*
**Serum hsCRP (mg/L)** *mean (± SD)*	*n* = 39	*n* = 31	*n* = 35	*n* = 34	***H*** = **101.40**, ***p*** **< 0.001** MDD CRP > 3 vs. others MDD CRP 1–3 vs. controls and vs. MDD CRP < 1
	0.55 ( ± 0.25)	1.56 ( ± 0.55)	5.37 ( ± 2.50)	1.04 ( ± 0.88)	
**Age (years)** *mean (± SD)*	*n* = 39	*n* = 31	*n* = 35	*n* = 34	*F* = 0.397, *p* = 0.756
	34.79 ( ± 7.44)	35.94 ( ± 7.68)	36.71 ( ± 7.48)	35.85 ( ± 7.96)	
**Sex** *n (%)*	*n* = 39	*n* = 31	*n* = 35	*n* = 34	χ^2^ = 3.072^*^, *p* = 0.381
	Females: 23 (58.97%)	Females: 22 (70.97%)	Females: 27 (77.14%)	Females: 22 (64.71%)	
**Ethnicity** *n (%)*	*n* = 39	*n* = 29	*n* = 35	*n* = 34	**χ**^**2**^ = 12.772^*^, ***p*** = **0.005**
	White: 36 (92.31%)	White: 25 (86.21%)	White: 30 (85.71%)	White: 21 (61.76%)	
**BMI (Kg/m**^**2**^**)** *mean (± SD)*	*n* = 39	*n* = 30	*n* = 34	*n* = 33	***F*** = **16.527**, ***p*** < **0.001**
	24.57 ( ± 2.58)	27.56 ( ± 4.83)	30.12 ( ± 4.11)	24.52 ( ± 4.03)	MDD CRP > 3 vs. controls and vs. MDD CRP < 1 MDD CRP 1–3 vs. controls and MDD CRP < 1
**Exposure and response to antidepressant treatment** *n (%)*	*n* = 39 MDD treatment responsive: 15 (38.46%) MDD treatment non-responsive: 17 (43.59%) MDD unmedicated: 7 (17.95%)	*n* = 31 MDD treatment responsive: 12 (38.71%) MDD treatment non-responsive: 10 (32.26%)	*n* = 35 MDD treatment responsive: 9 (25.71%) MDD treatment non-responsive: 20 (57.14%)	*n* = 34	χ^2^ = 4.75^*^, *p* = 0.309 Across the MDD subgroups only (no controls)
		MDD unmedicated: 9 (29.03%)	MDD unmedicated: 6 (17.14%)		
**HAM-D17** *mean (± SD)*	*n* = 39	*n* = 31	*n* = 35	*n* = 34	*H* = 1.448, *p* = 0.485
	12.13 ( ± 7.58)	13.16 ( ± 8.75)	14.26 ( ± 6.91)	0.53 ( ± 0.96)	Across the MDD subgroups only (no controls)

*BMI* body mass index, *CRP* C-reactive protein, *HAM-D17* Hamilton Rating Scale for Depression (17-item), *Hs* high-sensitivity, *IL* interleukin. *SD* standard deviation, *F* = ANOVA *F* value, H=Kruskal–Wallis *H* value; post-hoc analyses use Bonferroni correction (specific groups reported have statistically different mean scores (larger or smaller) compared with others); χ^2^ =Pearson Chi-Square; ^*^0% expected count less than 5. Significant tests (*p* < 0.05) are in bold.

### Biomarkers

Details of blood collection and analyses are in our previous papers [5, 20, 28] and Appendix. Blood samples taken from an antecubital vein at a time between 08^00^ and 10^00 ^a.m. on the same day of the clinical assessment.

CRP was measured in serum using Turbidimetry on Beckman Coulter AU analysers.

For whole blood mRNA, whole blood was collected in PaxGene tubes (2.5 mL) (PreAnalytiX, Hombrechtikon, CHE) for participants at each recruitment site and stored at −80 °C [20, 28]. Total RNA was isolated using the PAXgene blood miRNA kit according to the manufacturer’s protocol (PreAnalytiX, Hombrechtikon, CHE PaxGene miRNA kit (Qiagen, Hilden, Germany)). Quantity and quality of RNA were assessed through the NanoDrop spectrophotometer (NanoDrop Technologies, Delaware, USA) and the Agilent BioAnalyzer (Agilent Technologies). The RNA integrity number (RIN) was above 7 for all the samples, which were stored at −80 °C until processing.

### Whole-transcriptome RNA sequencing

Details are in the Appendix, but briefly:

Sequencing was performed on the Illumina NextSeq 550 instrument, applying the High Output Kit v2.5 (150 Cycles) paired-ended, read length 74, and following the NextSeq 550 System Guide (*document #15069765*). Sequencing data were then processed using Salmon, quasi-mapping mode (version 1.4.0) [29]. Gene-level count matrices were imported in R using *tximport* [30]. The differential gene expression was measured using the DESeq2 software package (v1.30.1) [31]. The raw counts pre-processing steps involved: (i) a minimal pre-filtering to keep the transcripts present in at least 2 samples with a count of 10 or more, (ii) normalisation, (iii) outlier detection, (iv) batch effect and unwanted variation removal using the Remove Unwanted Variation (RUV) method [32].

To identify the transcripts differentially expressed between groups (both up- and down-regulated), we applied an unadjusted *p*-value < 0.05, and a Benjamini-Hochberg adjusted FDR cut-off of 0.1 (*q*-value) [33]. We subsequently performed pathway analyses using the QIAGEN Ingenuity Pathway Analysis (IPA) software (QIAGEN, Redwood City, US) on all genes with a *p* < 0.05 and fold change (FC) ± | 1.2 |, and we identified differentially activated pathways (*p* < 0.05 and z-scores ≥|2|). On the same genes, we also conducted enrichment analyses using PANTHER (18.0) Gene Ontology [34] to identify biological processes, molecular functions, and cellular components significant at an FDR < 0.05.

## Results

### Immunometabolic pathways are activated in MDD with elevated CRP (1–3 mg/L) or low-grade inflammation (>3 mg/L) vs. controls, while cell-cycle-related pathways are inhibited in MDD with low levels of CRP ( < 1 mg/L) vs. controls

**Fig. 1:**
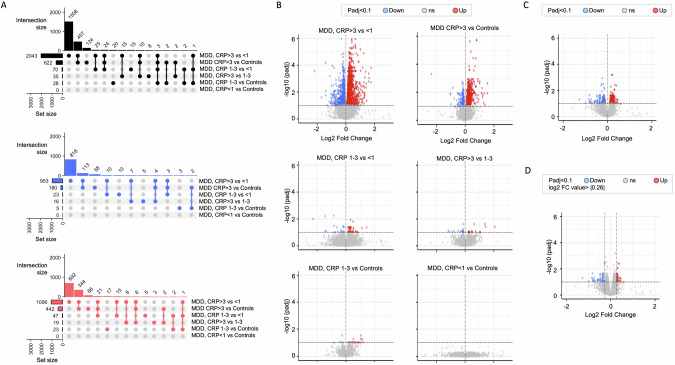
Differentially expressed transcripts (FDR *p*-adjusted <0.1) in CRP-based group comparisons. **A** UpSet plots to summarise key differentially expressed (DE) transcripts. These panels summarise the DE transcript overlap between comparisons for up-or down-regulated DE transcripts (in black), for down-regulated DE transcripts (in blue) and, upregulated DE transcripts (in red). In each panel, the bottom left horizontal bar graph labelled “Set size” shows the total number of DE transcripts per comparison. The circles in each panel’s matrix represent what would be the different Venn diagram sections (unique and overlapping DE transcripts). Connected circles indicate a certain intersection of DE transcripts between comparisons. The top bar graph in each panel summarises the number of DE genes for each unique or overlapping combination. In the top left panel, for example, the first vertical bar shows those DE transcripts that are unique to MDD with CRP > 3 mg/L vs. CRP < 1 mg/L (1508 DE transcripts). The second shows those DE transcripts that are shared only between MDD with CRP > 3 mg/L vs. CRP < 1 mg/L and MDD with CRP > 3 mg/L vs. controls (457 DE transcripts). **B, C, D** Volcano plots of RNA-seq expression analysis (**C, D** MDD with CRP <1 mg/L vs. controls with CRP <1 mg/L). Each dot represents a transcript comparing the conditions stated in the heading. The horizontal line corresponds to a Benjamini-Hochberg FDR-adjusted significance value of <0.1. The vertical lines in **D** correspond to a log2 FC value of |0.26| corresponding to an FC value of |1.2|.

The number of genes differentially expressed (up- and down-regulated, *p*-adjusted <0.1) in each CRP-based group comparison is reported in Fig. 1, together with respective volcano plots. Full lists of differentially-regulated genes (*p*-adjusted<0.1 and FC ± | 1.2|) are presented in the Appendix (Supplementary Table S2).

Functional analyses on FC-controlled *p*-values show a robust immune-related and metabolic mRNA signal in every group comparison between MDD with elevated CRP (1–3 mg/L) or low-grade inflammation (>3 mg/L) and both MDD with no inflammation (<1 mg/L) or healthy controls.

Using pathway analyses (*p* < 0.05, z-scores ≥|2|) on FC-controlled *p*-values, we find shared differentially regulated biological pathways, in both the CRP 1–3 and >3 groups compared with both controls and CRP < 1, all suggestive of immunometabolic activation. For example, the classical immune-related “Multiple Sclerosis Signalling Pathway” and “Pathogen Induced Cytokine Storm Signalling Pathway”, and the metabolic “EIF2 Signalling” and “Oxidative Phosphorylation”, are all activated, while the immune “Coronavirus Pathogenesis Pathway” is inhibited.

In addition to shared pathways, some are differentially regulated in either CRP 1–3 or >3 groups, highlighting a ‘more classical’ immune activation in CRP 1–3 and a greater shift toward metabolic dysfunctions in CRP > 3. For example, the “Role of Hypercytokinemia/hyperchemokinemia in the Pathogenesis of Influenza” and the “Interferon Signalling” are activated in the MDD CRP 1–3 mg/L, but not in the CRP > 3 mg/L, while the “Mitochondrial Dysfunction” is inhibited, and the “Neutrophil Extracellular Trap Signalling Pathway” and “S100 Family Signalling Pathway” are activated, in CRP > 3 mg/L, but not in the CRP 1–3 mg/L.

Surprisingly, we find evidence of a *stronger* activation in the MDD CRP 1–3 group vs. the CRP > 3 group. Specifically, two classical immune-related pathways, the “Role of Hypercytokinemia/hyperchemokinemia in the Pathogenesis of Influenza” and the “IL-33 Signalling Pathway”, are activated in the CRP 1–3 group vs. >3.

**Fig. 2:**
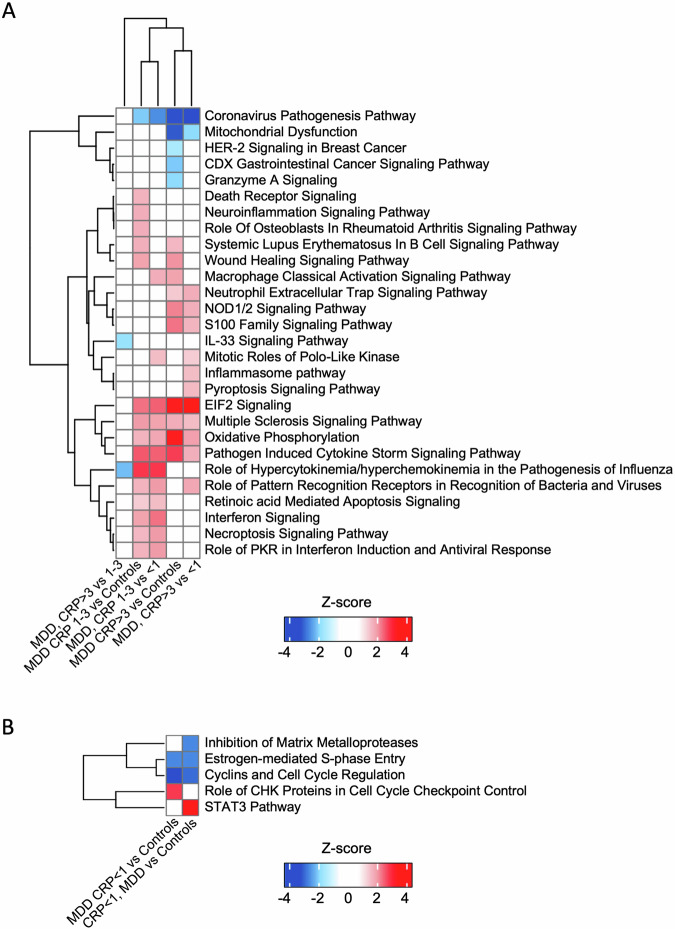
Canonical pathways differentially activated in CRP-based group comparisons. Heatmap of the activation z-score (IPA) of statistically significantly enriched pathways for at least one of the comparisons. Hierarchical clustering was used to group pathways and comparisons. **A** MDD CRP 1–3 and >3 vs. MDD CRP < 1 and controls. **B** MDD CRP < 1 vs. all controls and controls with CRP < 1.

Other pathways differentially activated/inhibited in single comparisons are presented in Fig. 2 and Supplementary Table S5.

Enrichment analyses confirm enriched immunometabolic biological, molecular, and cellular functions in these comparisons (see Summary and Supplementary Table S8 in the Appendix). Consistent with the pathway analyses, the CRP 1–3 group is more typically associated with immune-related and defensive mechanisms, while the CRP > 3 group is enriched in metabolic processes.

As mentioned above, we find no evidence of activation of immune-related pathways in the comparison between CRP < 1 mg/L and controls, which is consistent with the fact that these two groups have comparable levels of CRP and, if anything, CRP levels are numerically higher in controls (around 1 mg/L) than in MDD CRP < 1 mg/L (around 0.55 mg/L).

In additional analyses, we compare MDD with CRP < 1 mg/L vs. controls with CRP < 1 (*n* = 20), who have identical levels of CRP (0.55 vs. 0.45 mg/L). Pathway analyses show that people with CRP < 1 mg/L have *inhibited* cell-cycle-related mechanisms in comparison vs. both all controls or controls with CRP < 1 mg/L (see Fig. 2 and Supplementary Table S5﻿).

Shared pathways, that are inhibited in MDD CRP < 1 mg/L compared with both all controls and controls with CRP < 1 mg/L, include the “Estrogen-mediated S-phase Entry” and the “Cyclins and Cell Cycle Regulation”, while the “Role of CHK Proteins in Cell Cycle Checkpoint Control” is activated in MDD CRP < 1 mg/L vs. all controls only.

Interestingly, two immune pathways are differentially regulated in MDD CRP < 1 vs. controls with CRP < 1, the “Inhibition of Matrix Metalloproteases” (inhibited) and “STAT3 Pathway” (activated) (Fig. 2).

Enrichment analyses confirm the relevance of cell-related mechanisms in the MDD CRP < 1 group.

### Immunometabolic pathways are activated in all the treatment-based MDD groups vs. controls, with a specific transcriptomic profile in responders

The main clinical and sociodemographic features of the participants divided into the treatment-based groups of MDD participants are reported in the Appendix (Supplementary Table S1). Serum CRP levels are numerically higher in responders (around 2 mg/L) and unmedicated (around 2.6) vs. controls (around 1), but this difference is not statistically significant, while CRP levels are significantly elevated in MDD non-responders (around 2.7 mg/L) vs. controls. As expected, HAM-D17 scores are different between treatment-based groups, being significantly lower in responders, who are in remission (mean HAM-D17 of around 3) compared with both non-responders and unmedicated MDD, who both have a current diagnosis of MDD (mean HAM-D17 of around 17–19).

**Fig. 3:**
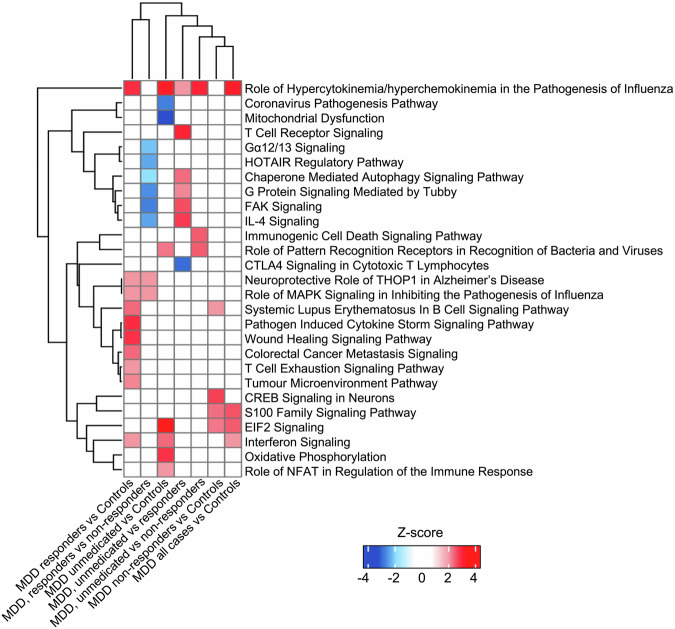
Canonical pathways differentially activated in treatment-based group comparisons. Heatmap of the activation z-score (IPA) of statistically significantly enriched pathways for at least one of the comparisons. Hierarchical clustering was used to group pathways and comparisons.

Differentially expressed transcripts are reported in the Appendix (Supplementary Fig. S2 and Supplementary Table S3). Pathway analyses (FC-controlled *p*-values, *p* < 0.05, z-scores ≥|2|) find differentially regulated pathways in every group comparison (although with fewer genes and pathways compared with the CRP-based grouping) (Fig. 3 and Supplementary Table S6).

Notably, we find activated immunometabolic pathways in all comparisons between MDD groups and controls, partially overlapping with those identified in the main CRP-based categorisation and further corroborating the immunometabolic transcriptional profile of MDD. Of note, the MDD responders have a specific pathway activation profile in comparison with controls or the other MDD groups, with the inhibition of immunometabolic pathways, such as the “IL-4 Signalling”, “FAK Signalling”, “T Cell Receptor Signalling”, “G Protein Signalling Mediated by Tubby”, and “Chaperone Mediated Autophagy Signalling Pathway”, and the activation of the “Neuroprotective Role of THOP1 in Alzheimer’s Disease” and of the anti-inflammatory “CTLA4 Signalling in Cytotoxic T Lymphocytes”.

**Fig. 4:**
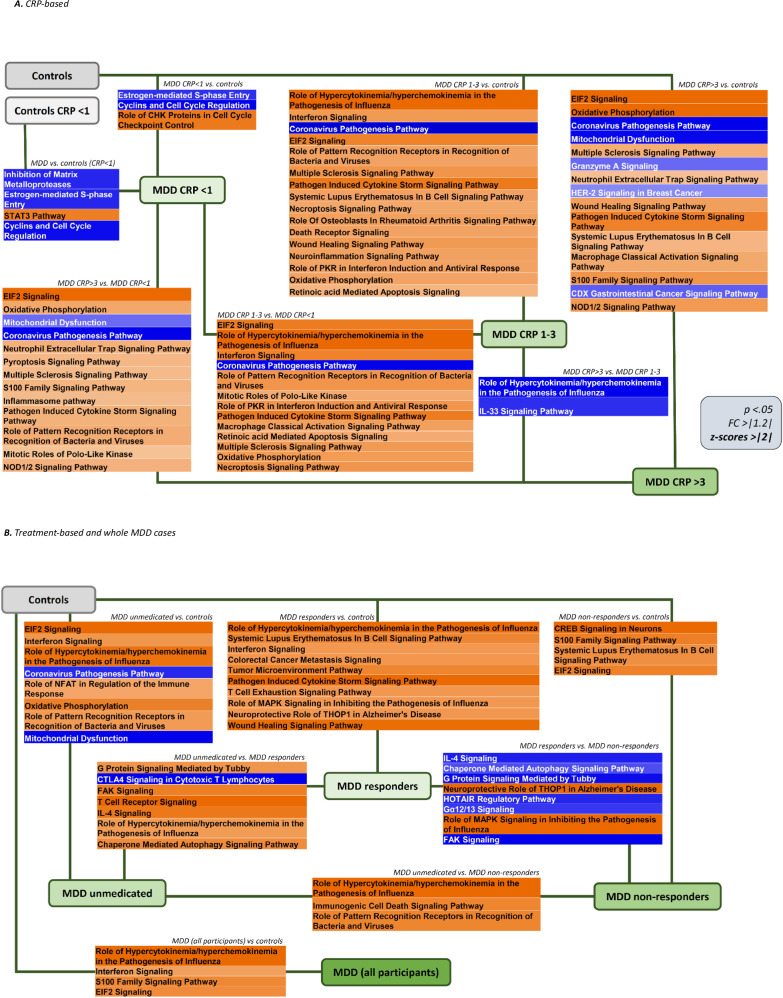
Schematic representation of canonical pathways in group comparisons. Schematic representation of the different pathways involved in group differences **A** in the CRP-based and **B** in the treatment-based MDD groups and all MDD cases compared with controls and one another. Gene transcripts have been selected based on *p* <0.05 and FC > | 1.2 | . The groups analysed in different comparisons are written above the respective box, activation or inhibition refers to the first group. Each box contains pathways for a single comparison, ordered based on *p*-values (lower on the top and higher on the bottom). Orange indicates a predicted activation of the pathway (positive z-scores), blue a predicted inhibition (negative z-scores); the intensity of colours reflects the z-score values. Canonical pathways have been selected based on *p* < 0.05 and z-scores ≥ |2|.

Corroborating our findings, in comparing the entire MDD participants (*n* = 106) with controls (*n* = 34), we find activation of immunometabolic pathways (see Fig. 4 and Supplementary Table S6). Moreover, in enrichment analyses, we confirm immunometabolic processes in all treatment-based groups vs. controls as well as in all depressed patients vs. controls (see Supplementary Table S8).

### In subgroup analyses, the transcriptomic profile of responders (vs. current MDD) shows inhibition of immune pathways independently of CRP levels, while the inhibition of cell-cycle-related pathways in MDD with CRP < 1 mg/L is present only in those who are currently depressed

To identify transcriptomic profiles that cut across CRP and treatment-based groups while limiting the number of subgroup analyses, we grouped patients in current MDD (non-responders and unmedicated), and remitted MDD (responders), and further divided them based on levels of CRP in >1 or <1 mg/L. Pathway analyses on differentially expressed transcripts (FC-controlled *p*-values, *p* < 0.05, z-scores ≥|2|) in all comparisons between these merged groups and controls are reported in the Appendix, both as an extensive narrative and as Supplementary Table S7.

Interestingly, responders have a specific profile compared with current MDD groups, independently of CRP levels. Within CRP > 1 mg/L, responders (vs. current MDD) have inhibition of seven immune-related pathways involving immune cell communication and receptors, still with activation of two inflammatory pathways (Supplementary Table S7 and Fig. S3A). Within CRP < 1 mg/L, responders (vs. current MDD) show mainly inhibition of immune-related pathways, including interferon (type I) and T cell-related signalling, still with activation of NF-κB signalling, immune cell communication, metabolic pathways, and translational and transcriptional regulators (Table S7 and Fig. S3B).

We also confirm inhibition of cell-cycle-related pathways in MDD with CRP < 1 mg/L, but only in those with current MDD, and not in responders. Instead, responders with CRP < 1 show inhibition of pleiotropic immunometabolic pathways and activation of anti-inflammatory pathways (“CTLA4” and “Oxytocin” signalling).

In the separate enrichment analyses, we confirm that transcripts in both current MDD and responders with CRP > 1 mg/L are enriched in immune-related processes and immunometabolic functions (vs. CRP < 1 mg/L and controls), as well as in MDD CRP < 1 vs. controls (independently of treatment status) and responders vs. non-responders (independently of CRP) (Appendix, Table S8).

## Discussion

In the present study, we find a differential transcriptomic activation of immunometabolic and cell-cycle pathways in MDD individuals compared with non-depressed controls, with specific signatures in distinct MDD immune and clinical phenotypes. We demonstrate that numerous immune-related and metabolic pathways are activated in MDD patients with CRP > 1 mg/L (that is, CRP 1–3 and >3) vs. controls or MDD with CRP < 1 mg/L, while MDD with CRP values < 1 mg/L show inhibition of cell-cycle-related pathways vs. controls. Similar immunometabolic pathways are also activated in all treatment-based groups of depressed patients vs. controls, with responders showing a specific immunomodulatory and neuroprotective signature when compared with the other groups. Subgroup analyses further confirm that responders have a specific immunomodulatory transcriptional profile, independently of CRP levels, while the inhibition of cell-cycle-related pathways in MDD with CRP < 1 is only present in those with current MDD, that is, in non-responders and unmedicated, but not in the responders. Our findings expand upon existing evidence on immune-related transcriptomic profiles when comparing depressed patients with controls [22–26], and offer valuable novel insights into the molecular profiles of depression.

Most of the pathways differentiating between, on the one hand, MDD CRP 1–3 and >3, and, on the other hand, MDD with CRP < 1 mg/L and controls, share common molecular mechanisms, enriched in genes involved in the organism’s defence and immune response. Thus, these could represent the transcriptional correlates of the inflammation signalled by CRP levels above 1 mg/L. For example, members of the TNF superfamilies (TNFSF, mainly 9, 10, and 15) are molecular mechanisms involved in the “Wound Healing Signalling Pathway”, the “Systemic Lupus Erythematosus In B Cell Signalling Pathway”, the “Pathogen Induced Cytokine Storm Signalling Pathway” and the “Multiple Sclerosis Signalling Pathway”, all activated in these comparisons. Most of the TNFSF molecules are expressed by, or can target, cells of the immune system, and they typically have a wide range of immune and cell-cycle-related actions, including promoting immune cell differentiation and survival, and the production of inflammatory cytokines and chemokines [35]. The inhibition of the “Coronavirus Pathogenesis Pathway” in these same comparisons, which is opposite to the activation of other immune-related pathways, is consistent with these findings, as the overall pathway function aims to inhibit viral replication, and thus its inhibition might lead to more viral release, respiratory infection, and SARS-CoV replication, in turn leading to more immune activation. Similarly activated are also the metabolic “EIF2 Signalling” and “Oxidative Phosphorylation” pathways, which have a strong immune-related component and could hence be relevant in the broader context of ‘immunometabolic depression’. In particular, EIF (eukaryotic initiation factor)-2 is not only relevant for protein synthesis and energy expenditure [36] but also plays a vital role in immune responses, including the regulation of NF-κB activation and pro-inflammatory gene expression [37]. The pathway “Oxidative Phosphorylation” represents the most important cellular process occurring in the mitochondria, through which stored chemical nutrients generate energy in the form of adenosine triphosphate (ATP). The activation of this pathway typically occurs in response to increased energy demands or metabolic requirements, such as during inflammation [38].

Despite these similarities, it is important to highlight that the immunometabolic signatures associated with the MDD CRP 1–3 and >3 groups do not entirely overlap, and indeed we identify a stronger immune-related signal in patients with CRP 1–3 mg/L compared with those with CRP > 3 mg/L, even though, as expected by design, individuals in the CRP > 3 group have significantly higher serum CRP levels (mean 5.4 vs. 1.6 mg/L in the 1–3 group, Table 1). These findings are consistent with our previous study using candidate mRNA genes, where we also found a disconnection between transcription (immune-related mRNA expression) and translation (protein markers of inflammation) [20]. Notably, depressed patients with CRP between 1 and 3 mg/L already had an unexpected candidate mRNA profile in our previous study; for example, the two immune genes, CCL2 and STAT1, were higher in CRP < 1 and CRP > 3 mg/L groups, but not in those with CRP 1–3 mg/L, and thus did not reflect the gradient of CRP levels. Here, we may be observing the same phenomena at a whole-transcriptome level. Specifically, depressed people with CRP 1–3 mg/L, defined as having elevated CRP but not the low-grade inflammation signalled by values above 3 mg/L [12], appear to have an ‘intermediate’ phenotype, with a transcriptional ‘predisposition’ to inflammation coupled with ‘protective mechanisms’ against its translation in immune protein signal. Consequently, individuals in this group have a greater immune-related transcriptional profile but with lower levels of CRP. Intriguingly, this population shows no antidepressant response in previous randomised control trials with anti-inflammatory interventions, as a response tend to be present only in people with higher levels of CRP ( > 3–5 mg/L) [15, 16], even though they show here the same or even more immune transcriptional activation than those with CRP > 3. It is possible to speculate that this lack of effects is due to the lack of downstream protein-level inflammation, which might be ultimately responsible for the depressogenic effects of peripheral inflammation on the brain and thus is the actual target for the antidepressant action of anti-inflammatories [39].

Supporting this notion, the MDD CRP > 3 group has a profile that is in part ‘qualitatively﻿’ different from those with CRP 1–3, with activation of both immune and metabolic pathways, possibly indicating the downstream consequences of the clinically-significant ‘low-grade inflammation’. For example, the inhibition of the “Mitochondrial Dysfunction” pathway is mainly due to a regulation of several mitochondria-related elements, such as ATP5, COX, and NDUF, which might represent adaptative translational mechanisms following cellular damage and stress [40]. Indeed, Scaini et al. [41] also showed stronger evidence of dysregulation of mitochondrial dynamics in MDD patients with higher CRP levels. Compared with controls, the CRP 1–3 group is enriched in genes more classically immune-related (such as type-I-interferon-related), while the CRP > 3 group is also enriched in metabolic genes (such as mitochondria-related energy production). It is also conceivable that this metabolic transcriptional signature reflects heterogeneity in metabolic status, as observable in a numerical (even though non-significant) higher BMI (30 vs. 28) and potentially associated metabolic abnormalities in the CRP > 3 vs. 1–3 groups.

In contrast with those with elevated CRP ( > 1 mg/L), the MDD with CRP < 1 mg/L group shows inhibition of pathways involved in the cell cycle and cellular proliferation. RNA-seq evidence of down-regulation of cell-cycle-related genes has already been described in MDD vs. controls [23, 25], although in populations not selected or stratified for the levels of inflammation. Our findings indicate that this transcriptional profile is relevant to MDD patients with no inflammation, and indeed we can speculate that this profile acts as a protective factor against the development of clinically-relevant inflammation. For example, the inhibition of the “Estrogen-mediated S-phase Entry” and “Cyclins and Cell Cycle Regulation”, and the activation of the “Role of CHK Proteins in Cell Cycle Checkpoint Control”, all protect against uncontrolled cellular proliferation. While it is possible that these cell-cycle-related pathways ‘protect’ these patients against the increased CRP, we nevertheless notice some mRNA evidence of immune activation in the comparison with healthy controls with CRP < 1 mg/L. This signature includes the activation of “STAT3 Pathway”, which is activated by IL-6 [42], and the inhibition of “Inhibition of Matrix Metalloproteases”, leading to an up-regulation of the matrix metalloprotease genes, MMP-8, −19, −25, −28, which are also activated in inflammatory conditions [43]. These findings corroborate our previous candidate-gene evidence demonstrating an immune-related transcriptional activation in MDD with CRP < 1 mg/L vs. controls with CRP < 1 mg/L [20], and might indicate a minimal ‘transcriptional immune activation’ which is common to all depressed patients, even in the absence of elevated CRP. Enrichment analyses also confirm enrichment in cell-related genes, including neuron-related genes, in the comparison between MDD < 1 mg/L vs. all the controls, and immune-related genes in the comparison vs. controls with CRP < 1 mg/L.

Treatment-based group comparisons identify pathways activated in unmedicated, non-responder, and responder groups vs. controls, even though we find fewer pathways than in the CRP-based comparisons. Many pathways are common to those identified in the CRP-based comparisons and have been discussed above. However, we want to highlight the specific transcriptomic profile of responders compared with the other MDD groups or controls. Genes in responders are enriched in adaptive immunity functions, with T cell-related activities vs. both non-responders and unmedicated, and B cell-related activities vs. non-responders only. Pathways inhibited in responders vs. both the other MDD groups include the “IL-4 Signalling” pathway, with pleiotropic actions (both pro- and anti-inflammatory) and a primary role in mediating allergic inflammatory responses [44], the signalling regulator “G Protein Signalling Mediated by Tubby” [45], involved in neuronal development and function [46], the cell migration and angiogenesis-related “FAK Signalling” [47], involved in immune-related and inflammatory processes [48], and the “Chaperone Mediated Autophagy Signalling Pathway”, involved in cellular homoeostasis and implicated in neurodegenerative disorders, cancer, and metabolic disorders [49]. In addition, responders have an activation vs. non-responders of the “Neuroprotective Role of THOP1 in Alzheimer’s Disease”, involved in the neuroprotective response to amyloid beta toxicity [50], and the “Role of MAPK Signalling in Inhibiting the Pathogenesis of Influenza”, important in influenza-virus infections and involved in several immune-related processes such as cellular proliferation, differentiation, inflammation, and death [51]. It is possible that this combination of immunomodulatory and neuroprotective pathways in responders may predispose to a better antidepressant response, although, of course, this signature could also just be the consequence of these patients being in remission from their depression.

Analyses merging CRP and treatment-based groups confirm the specific transcriptomic profile of responders, independently of the CRP levels. In patients with CRP > 1 mg/L, responders (vs. current MDD, that is, unmedicated and non-responders) have consistent inhibition of pathways related to the interactions between immune cells and the immune receptors functioning (scavenger, Fc, and B cell receptors). These findings reveal an important role of inhibition of immune cell trafficking, a crucial component of inflammatory responses [52], in this ‘intermediate’ phenotype of responders with CRP > 1 mg/L, who are in remission from depression but still have elevated CRP levels. In patients with CRP < 1 mg/L, responders (again, vs. current MDD) show inhibition of interferon signalling, confirming and expanding upon previous RNA-seq findings identifying upregulation of genes in this pathway in people with MDD [23] or treatment-resistant depression [25] in comparison with controls.

We also confirm inhibition of cell-cycle-related pathways and activation of proinflammatory pathways in MDD with CRP < 1 mg/L, but only in current MDD (not in responders), further corroborating the hypothesis that these transcriptional mechanisms might prevent the development of clinical inflammation in people with current depression. In contrast, responders with CRP < 1 mg/L have not only inhibition of immunometabolic pathways but also activation of the oxytocin signalling pathway, which has several anti-inflammatory properties and has been investigated as a potential therapeutic target in depression [53].

Lastly, individuals with current MDD and responders with CRP > 1 mg/L have immunometabolic activation vs. controls and CRP < 1 groups. Although expected, reflecting different levels of inflammation, this difference suggests a greater influence of CRP levels over treatment status in our study. This consideration is important and indicates that immunometabolic serum and transcriptional differences should take precedence over treatment-based clinical phenotypes when selecting patients for future immunopsychiatry trials.

In terms of limitations, RNA-seq has several advantages compared with candidate-gene approaches, primarily allowing for a hypothesis-free transcriptome-wide analysis of gene expression [21]. However, some drawbacks must be considered. Besides higher costs and complexity of analyses compared with qPCR, a loss of information is possible for selected genes [54] that contain regions with high GC content or repetitive sequences, although reassuringly, our data passed the specific quality control test for the GC content and Salmon corrects for fragment GC-content bias [29]. Also, we did not use qPCR to replicate key genes identified in the mRNA sequencing, but there is now evidence to suggest that RNA-seq methods are robust and do not require independent validation by qPCR [55]. Third, RNA-seq analyses always carry the risk associated with multiple comparisons, but our bioinformatic pipeline has used established approaches to select stringent findings. Specifically, to identify the transcripts differentially expressed between groups, we used an adjusted *p* < 0.1 and an FC ± | 1.2|; for pathway analyses, we used all genes with FC ± | 1.2| and *p* < 0.05; and, for automatically identify potential confounders, we used correction using the RUV method, a data-driven approach [32]; these strategies are all in line with previous transcriptomics studies [22, 56, 57]. We acknowledge that an FDR threshold of 0.1 is not the most stringent, however, this was only used for the initial differential expression analyses, while pathway analyses have been conducted on transcripts differentially expressed based on a *p* < 0.05 and FC ± | 1.2|. Indeed, the FC and z-score thresholds allowed us to infer the direction of pathway activation, enhancing the clinical significance of our findings. Also, our sample size remains rather limited, particularly for inter-group analyses. An additional limitation is the absence of cell-type correction but, to the best of our knowledge, no reliable datasets are available to infer cell-type-specific expression profiles to be used as a reference for whole-blood mRNA. From a clinical point of view, we were unable to assess specific symptom domains consistently associated with inflammation, such as anhedonia, appetite/sleep disturbances, or fatigue [58, 59], because these symptoms are not fully captured by the HAM-D17; future studies should focus on more tailored outcome measures in immunopsychiatry [59]. Finally, the cross-sectional design of the present study is another potential limitation, as it does not consent to measure changes in gene expression (both mRNA and protein levels) over time, and future research will have to expand on these findings with larger samples and longitudinal designs, especially to check if these molecular mechanisms are either antecedents or causally-related to specific clinical phenotypes such as ‘immunometabolic’ or ‘non-responsive’ depression.

In conclusion, using RNA-seq of whole-blood mRNA, we shed light on the potential risks and compensatory molecular mechanisms that could facilitate or prevent the trajectory of an individual patient toward immunometabolic depression and/or treatment-non-responsive depression. This knowledge will ultimately enable a more tailored approach to MDD research. It could, for example, inform stratification strategies for selecting participants for anti-inflammatory trials based on immune-related protein and mRNA data. It could also inform the development of novel antidepressant treatments, either addressing the transcriptional immunometabolic activation observed in MDD with increased levels of inflammation or promoting protective mechanisms observed in non-inflamed MDD patients and responders. By recognising and understanding these mechanisms, we can facilitate the development of tailored strategies for early detection and treatment.

## Supplementary information

Appendix

## Data Availability

The raw data supporting the conclusions of this article are archived in the EMBL’s European Bioinformatics Institute (EMBL-EBI) under accession numbers PRJEB79258.
