# Supplementary material for: Transcriptomic profiles in major depressive disorder: the role of immunometabolic and cell-cycle-related pathways in depression with different levels of inflammation
Source: Mol Psychiatry. 2024 Sep 13;30(4):1308–18. doi: 10.1038/s41380-024-02736-w (PMC11919688; doi:10.1038/s41380-024-02736-w)
Supplement: Supplementary file 1 — Appendix [file 41380_2024_2736_MOESM1_ESM.docx]

**Transcriptomic profiles in major depressive disorder: the role of immuno-metabolic and cell-cycle-related pathways in depression with different levels of inflammation**

*Appendix*

[**NIMA members during the sample collection and data analysis period for the BIODEP study** 3](#_Toc168300394)

[**Supplementary Methods** 5](#_Toc168300395)

[**Study design and sample characteristics** 5](#_Toc168300396)

[**Biomarkers** 5](#_Toc168300397)

[**Whole-transcriptome RNA sequencing** 6](#_Toc168300398)

[**Supplementary Results** 7](#_Toc168300399)

[**Immunometabolic pathways are activated in MDD with elevated CRP (1-3 mg/L) or low-grade inflammation (>3 mg/L) vs. controls, while cell-cycle-related pathways are inhibited in MDD with low levels of CRP (<1 mg/L) vs. controls** 7](#_Toc168300400)

[**Immunometabolic pathways are activated in all the treatment-based MDD groups vs. controls, with a specific transcriptomic profile in responders** 7](#_Toc168300401)

[**In subgroup analyses, the transcriptomic profile of responders (vs. current MDD) shows inhibition of immune pathways independently of CRP levels, while the inhibition of cell-cycle-related pathways in MDD with CRP<1 mg/L is present only in those who are currently depressed** 7](#_Toc168300402)

[***Supplementary Table S1:*** *Clinical and sociodemographic characteristics (treatment-based grouping)* 10](#_Toc168300403)

[***Supplementary Table S2:*** *mRNA transcripts differentially expressed (FDR-adjusted) in CRP-based MDD cases and controls* 12](#_Toc168300404)

[***Supplementary Table S3:*** *mRNA transcripts differentially expressed (FDR-adjusted) in treatment-based MDD cases and controls* 33](#_Toc168300405)

[***Supplementary Table S4:*** *mRNA transcripts differentially expressed (FDR-adjusted) in all MDD cases vs. controls* 36](#_Toc168300406)

[***Supplementary Table S5:*** *Canonical pathways differentially activated in group comparisons (CRP-based MDD groupings)* 37](#_Toc168300407)

[***Supplementary Table S6:*** *Canonical pathways differentially activated in group comparisons (treatment-based MDD groupings and whole MDD cases)* 41](#_Toc168300408)

[***Supplementary Table S7:*** *Canonical pathways differentially activated in group comparisons (merging CRP-based and treatment-based MDD groups)* 43](#_Toc168300409)

[***Supplementary Table S8:*** *PANTHER Gene Ontology enrichment analyses in all group comparisons (CRP-based, treatment-based, and merged groups)* 58](#_Toc168300410)

[***Supplementary Figure S1:*** *Volcano plots of differentially expressed transcripts (p<0.05, FC>|1.2|) in CRP-based group comparisons* 98](#_Toc168300411)

[***Supplementary Figure S2:*** *Differentially expressed transcripts in treatment-based group comparisons* 99](#_Toc168300412)

[***Supplementary Figure S3:*** *Bubble charts of pathway clusters differentially activated in responders vs. current MDD patients (non-responders and unmedicated) selected for similar levels of CRP.* 101](#_Toc168300413)

[**References (Appendix)** 102](#_Toc168300414)

# **NIMA members during the sample collection and data analysis period for the BIODEP study**

Brighton & Sussex University Hospitals NHS Trust

Dominika Wlazly

Cambridgeshire & Peterborough NHS Foundation Trust

Amber Dickinson, Andy Foster, Clare Knight

Cardiff University

Claire Leckey, Paul Morgan, Angharad Morgan, Caroline O'Hagan, Samuel Touchard

GSK

Shahid Khan, Phil Murphy, Christine Parker, Jai Patel, Jill Richardson

Janssen

Paul Acton, Nigel Austin, Anindya Bhattacharya, Nick Carruthers, Peter de Boer, Wayne Drevets, John Isaac, Declan Jones, John Kemp, Hartmuth Kolb, Jeff Nye, Gayle Wittenberg

King’s College London

Gareth Barker, Anna Bogdanova, Heidi Byrom, Diana Cash, Annamaria Cattaneo, Daniela Enache, Tony Gee, Caitlin Hastings, Melisa Kose, Giulia Lombardo, Nicole Mariani, Anna McLaughlin, Valeria Mondelli, Maria Nettis, Naghmeh Nikkheslat, Carmine Pariante, Karen Randall, Julia Schubert, Luca Sforzini, Hannah Sheridan, Camilla Simmons, Nisha Singh, Federico Turkheimer, Vicky Van Loo, Mattia Veronese, Marta Vicente Rodriguez, Toby Wood, Courtney Worrell, Zuzanna Zajkowska

Lundbeck

Brian Campbell, Jan Egebjerg, Hans Eriksson, Francois Gastambide, Karen Husted Adams, Ross Jeggo, Thomas Moeller, Bob Nelson, Niels Plath, Christian Thomsen, Jan Torleif Pederson, Stevin Zorn

NHS Greater Glasgow and Clyde

Catherine Deith, Scott Farmer, John McClean, Andrew McPherson, Nagore Penandes, Paul Scouller, Murray Sutherland

Oxford Health NHS Foundation Trust

Mary Jane Attenburrow, Jithen Benjamin, Helen Jones, Fran Mada, Akintayo Oladejo, Katy Smith

Pfizer

Rita Balice-Gordon, Brendon Binneman, James Duerr, Terence Fullerton, Veeru Goli, Zoe Hughes, Justin Piro, Tarek Samad, Jonathan Sporn

Sussex Partnership NHS Foundation Trust

Liz Hoskins, Charmaine Kohn, Lauren Wilcock

University of Cambridge

Franklin Aigbirhio, Junaid Bhatti, Ed Bullmore, Sam Chamberlain, Marta Correia, Anna Crofts, Tim Fryer, Martin Graves, Alex Hatton, Manfred Kitzbichler, Mary-Ellen Lynall, Christina Maurice, Ciara O'Donnell, Linda Pointon, Peter St George Hyslop, Lorinda Turner, Petra Vertes, Barry Widmer, Guy Williams

University of Glasgow

Jonathan Cavanagh, Alison McColl, Robin Shaw

University of Groningen

Erik Boddeke

University of Oxford

Alison Baird, Stuart Clare, Phil Cowen, I-Shu (Dante) Huang, Sam Hurley, Simon Lovestone, Alejo Nevado-Holgado, Elena Ribe, Anviti Vyas, Laura Winchester

University of Southampton

Madeleine Cleal, Diego Gomez-Nicola, Renzo Mancuso, Hugh Perry

University of Sussex

Mara Cercignani, Charlotte Clarke, Alessandro Colasanti, Neil Harrison, Rosemary Murray

University of Texas

Jason O'Connor

University of Toronto

Howard Mount

# **Supplementary Methods**

**Study design and sample characteristics**

Participants were recruited and assessed in 5 clinical centres in the UK: Brighton, Cambridge, Glasgow, London (KCL), and Oxford. The study was conducted as part of the Wellcome Trust NIMA Consortium. The study was approved by the Research Ethics Committee (National Research Ethics Service East of England, Cambridge Central, United Kingdom; approval number: 15/EE/0092). All the procedures of the study complied with the ethical standards of the relevant national and institutional committees on human experimentation and with the Helsinki Declaration of 1975, as revised in 2008 [1, 2].

All participants provided written informed consent and underwent eligibility screening before participating in any study procedures. Participants were recruited from primary and secondary National Health Service (NHS) health services and the general population. All participants were aged 25-50 years. Individuals with a lifetime history of bipolar disorder or non-affective psychosis were excluded. The healthy controls had no current or past diagnosis of any major psychiatric disorder as defined by DSM-5 [3], and no history of antidepressant drug treatment for any indication. Other exclusion criteria applied to both the healthy control and the MDD participant samples were any lifetime medical disorder or current use of medications likely to compromise interpretation of CRP, alcohol or substance use disorder in the preceding 12 months, and current pregnancy or breastfeeding.

The BIODEP study was designed as two, tiered cohorts. In the primary cohort, depression cases were identified based on MDD diagnosis, as assessed through the SCID-5 [4]. Non-depressed controls have been sampled from the general population to match the patient group in terms of age, sex, and BMI. Participants with MDD were stratified based on exposure and therapeutic response to monoaminergic antidepressant medications and the severity of depressive symptoms measured with the HAM-D17 [5]. In the secondary cohort, MDD patients with current depression (HAM-D scores >13, therefore excluding responders) have been further stratified based on the levels of inflammation, as measured by serum hsCRP.

All participants were recruited from the BIODEP secondary cohort, except the MDD responders, who were included from the primary cohort by study design. These individuals are the only ones overlapping (same samples) with previously published gene expression papers [6–8].

## **Biomarkers**

Biological markers were measured in blood samples taken from an antecubital vein at a time between 08^00^ and 10^00^ a.m. on the same day of the clinical assessment. Before the sampling, participants were instructed to lie supine for 30 minutes, fast for 8 hours, and abstain from strenuous exercise for 72 hours.

CRP was measured in serum as previously described [9]. Peripheral blood samples collected in clotting tubes were left to coagulate at room temperature for 30-60 minutes. Subsequently, tubes were centrifuged for 15 minutes at 1600 g and transferred to a central laboratory (Q^2^ Solutions Laboratory, Livingston, Scotland, UK). Samples were exposed to anti-CRP-antibodies on latex particles, and the increase in light absorption due to complex formation was used to quantify hs-CRP levels, using Turbidimetry on Beckman Coulter AU analysers.

## **Whole-transcriptome RNA sequencing**

*Libraries preparation, control, dilution, and denaturation*

The total RNA (150 ng) extracted from the PAXgene tubes of each participant was processed to prepare dual-indexed libraries using the Illumina Stranded mRNA Prep Ligation Kit. The dual-indexed libraries were then checked for quality and concentration, 1 μL of each sample’s library was analysed with the Agilent 2100 Bioanalyzer and DNA 1000 Kit, while the concentration of the libraries was measured using the Qubit dsDNA BR Assay kit. After libraries had been checked, samples were randomised, and libraries were diluted to the starting concentration for the sequencing system (1 nM). Then, 10 μL of each diluted library (1 nM) were pooled together and processed before being loaded into the NextSeq 550 reagent cartridge.

*Sequencing, data processing, and biostatistical analysis*

Sequencing was performed on the Illumina NextSeq 550 instrument, applying the High Output Kit v2.5 (150 Cycles) paired-ended, read length 74, and following the NextSeq 550 System Guide (*document #15069765*). The raw data generated (binary base call (BCL) files) have been uploaded to the BaseSpace Sequence Hub, Local Run Manager. For all the runs the %Q30 (percentage of bases with a quality score of 30 or higher) was >93% and the %PF (number of clusters that passed the filter) was >84%. BCL files have been converted to FastQ format and checked for quality with the FastQC quality control tool ([*https://www.bioinformatics.babraham.ac.uk/projects/fastqc/*](https://www.bioinformatics.babraham.ac.uk/projects/fastqc/)).

Sequencing data were then processed using Salmon, quasi-mapping mode (version 1.4.0) [10]. Firstly, the human genome was joined together with the end of the human transcriptome (Release 38 (GRCh38.p13)), both the human genome and transcriptome were downloaded from [*https://www.gencodegenes.org/human/*](https://www.gencodegenes.org/human/)), and subsequently it was indexed with chromosome names (as described in [*https://combine-lab.github.io/alevin-tutorial/2019/selective-alignment/*](https://combine-lab.github.io/alevin-tutorial/2019/selective-alignment/)). Secondly, the paired-end reads were directly quantified by using the index previously developed.

Gene-level count matrices were imported in R using *tximport* [11]. The differential gene expression was measured using the DESeq2 software package (v1.30.1) [12]. The raw counts pre-processing steps involved: i) a minimal pre-filtering to keep the transcripts present in at least 2 samples with a count of 10 or more, ii) normalization, iii) outlier detection, iv) batch effect and unwanted variation removal using the Remove Unwanted Variation (RUV) method [13]. RUV uses factor analysis to adjust for known covariates of interest (such as the batch effect) and unknown nuisance variables based on genes that are not expected to be influenced by the biological variables of interest. The subset of data to estimate the factors of unwanted variation was identified using a data-driven approach where Deseq2 was used to run the differential expression analyses and the negative control genes (RUVg) were selected by applying a cut-off of 0.06 to p-values. The number of hidden factors was chosen based on the number of batches (k=8). After checking that no hidden factors were associated with the biological variable of interest (those associated were dropped), they were treated as additional covariates in the models for differential expression analyses.

To identify the transcripts differentially expressed between groups (both up- and down-regulated), we applied an unadjusted p-value <0.05, and a Benjamini-Hochberg adjusted FDR cut-off of 0.1 (q-value) [14]. We subsequently performed pathway analyses using the QIAGEN Ingenuity Pathway Analysis software (QIAGEN, Redwood City, US) on all genes with a p<0.05 and fold change (FC)±|1.2|, and we identified differentially activated pathways (p<0.05 and z-scores ≥|2|). On the same genes, we also conducted enrichment analyses using PANTHER (18.0) Gene Ontology [15] to identify biological processes, molecular functions, and cellular components significant at an FDR<0.05.

# **Supplementary Results**

## **Immunometabolic pathways are activated in MDD with elevated CRP (1-3 mg/L) or low-grade inflammation (>3 mg/L) vs. controls, while cell-cycle-related pathways are inhibited in MDD with low levels of CRP (<1 mg/L) vs. controls**

Enrichment analyses confirm enriched immunometabolic biological, molecular, and cellular functions in these comparisons (Supplementary Table S8). The CRP 1-3 group, compared with both controls and CRP<1, is more typically associated with immune-related and defensive mechanisms, such as “immune” and “defense” response to “biotic stimulus”, “other organism”, “bacterium”, “cytokine”, and “virus”; of note, antiviral mechanisms are particularly prominent, with enrichment in “response to virus” and regulation of “viral genome replication”, “life cycle” and “process” alongside “interferon-mediated signalling” and “response”, particularly “type I” (“alpha” and “beta”). On the other hand, the CRP>3 group (vs. both controls and CRP<1), besides “immune” and “defense” response, is enriched in metabolic processes, such as mitochondria-related “electron transport chain”, “ATP synthesis”, and “cellular” and “aerobic” respiration, together with peptide and RNA “metabolic” and “biosynthetic” process. These differences are supported by the enrichment in immune processes and responses in the comparison between CRP1-3 and >3 groups.

Enrichment analyses confirm the relevance of cell-related mechanisms in MDD CRP<1 group. vs. (all) controls, with enrichment in cell “periphery”, “body”, and “membrane” cellular components, including “neuron projection” and “synapse”; while, in the comparison between MDD and controls with CRP<1 mg/L, the main enriched biological processes are “immune system process” and “immune response” (Table S8).

## **Immunometabolic pathways are activated in all the treatment-based MDD groups vs. controls, with a specific transcriptomic profile in responders**

In enrichment analyses, we confirm immunometabolic processes in all treatment-based groups vs. controls as well as in all depressed patients vs. controls (see Supplementary Table S8). The comparison between responders and both non-responders and unmedicated is enriched in “T cell receptor complex” cellular component; with additional immune-related biological processes (such as “immune response”, “immunoglobulin” and “molecular mediator of immune response” production” and “cell adhesion”) and “antigen binding” molecular function in responders vs. non-responders only.

**In subgroup analyses, the transcriptomic profile of responders (vs. current MDD) shows inhibition of immune pathways independently of CRP levels, while the inhibition of cell-cycle-related pathways in MDD with CRP<1 mg/L is present only in those who are currently depressed**

To identify transcriptomic profiles that cut across CRP and treatment-based groups, we further grouped MDD patients in current MDD (non-responders and unmedicated; HAM-D17>13), and responders (in remission; HAM-D17<7), and divided them based on levels of CRP (> or <1 mg/L).

Pathway analyses on differentially expressed transcripts (FC-controlled p-values, p<0.05, z-scores ≥|2|), demonstrate a consistent immunometabolic activation in *a)* CRP>1 (current MDD and responders) vs. CRP<1 (both groups) and controls, and in *b)* current MDD (CRP>and<1) vs. controls. Responders have a specific immunometabolic transcriptional profile compared with controls and patients with current MDD (non-responders and unmedicated) with similar levels of inflammation. Pathways differentially activated in these comparisons are presented in the Appendix (Supplementary Table S7).

Immunometabolic pathways are activated with elevated levels of CRP (>1 mg/L), both in currently depressed (n=45) and responders (n=21) vs. controls and MDD with CRP<1 mg/L (currently depressed, n=24; and responders, n=15), largely overlapping with pathways identified in CRP-based and treatment-based groups (Supplementary Table S7). Of note, this activation includes not only individuals with current MDD and CRP>1 mg/L vs. responders with CRP<1 mg/L, but also responders with CRP>1 mg/L vs. current MDD CRP<1 mg/L and controls, identifying numerous immunometabolic and cell-cycle-related transcriptomic pathways that in our sample are more clearly associated with increasing levels of inflammation than with treatment status, thus reflecting inflammation as measured with CRP levels.

In groups with no inflammation (CRP<1 mg/L), compared with controls, we confirm inhibited cell-cycle-related pathways (“Cyclins and Cell Cycle Regulation” and “Mitotic G1 phase and G1/S transition”), but only in individuals with current MDD. In addition, pathways linked to immune cell proliferation (“Dendritic Cell Maturation”), cellular communication (“Immunoregulatory interactions between a Lymphoid and a non-Lymphoid cell”, “Communication between Innate and Adaptive Immune Cells”, “Cell surface interactions at the vascular wall”), and immune cell receptors (“Binding and Uptake of Ligands by Scavenger Receptors”, “Fcgamma receptor dependent phagocytosis”, “Fc epsilon receptor signaling”, “Signaling by the B Cell Receptor”) are inhibited. Notably, we still observe activation of immune-related transcriptional pathways (“OAS antiviral response” and “Role of Hypercytokinemia/hyperchemokinemia in the Pathogenesis of Influenza”).

The specific profile associated with responders also emerges in the merged comparisons. Responders with CRP>1 mg/L (vs. current MDD CRP>1) have inhibition of several immune-related pathways, mainly involving the communication between immune cells and immune cell receptors; all these pathways overlap with the comparison between current MDD with CRP<1 mg/L and controls (see below), and might identify transcriptional profiles cutting across different groups and conditions (see Supplementary Table S7 and Figure S3A); in contrast, two inflammatory pathways are activated (“Role of Hypercytokinemia/hyperchemokinemia in the Pathogenesis of Influenza” and “Interferon gamma signaling”). Findings on MDD groups with CRP<1 mg/L confirm the involvement of different immunometabolic pathways in responders vs. current MDD, with differential activation and inhibition (see Supplementary Table S7 and Figure S3B). For instance, we observe a considerable overlap with pathways inhibited in responders with CRP<1 mg/L vs. controls, with additional inhibition of immune-related pathways, including interferon (type I) and T cell-related signalling, together with activation of the metabolic “EIF2 Signaling” and “Selenoamino acid metabolism”, the immune cell trafficking “Communication between Innate and Adaptive Immune Cells”, and pathways involved in translational and transcriptional regulation (Table S7 and Figure S3B). Of note, and as expected by design, there are no significant differences in CRP and BMI levels between responders and current MDD groups with similar levels of CRP (both > and <1 mg/L). Mean CRP levels are *a)* 3.7 vs. 3 mg/L, and *b)* 0.5 vs. 0.6 mg/L, while BMI is *a)* 28.4 vs. 29.9, and *b)* 24.8 vs. 24.1 respectively in currently depressed vs. responders with *a)* CRP>1 and *b)* CRP<1.

Notably, while responders with CRP>1 mg/L show activation of immune pathways reflecting CRP levels, responders with CRP<1 mg/L show a different profile compared with controls, with inhibition of pleiotropic immunometabolic pathways, partially overlapping with the ones identified in responders vs. unmedicated and non-responders (“G Protein Signaling Mediated by Tubby”, “IL-4 Signaling”, “Chaperone Mediated Autophagy Signaling Pathway”). The immune-related “Lipid Antigen Presentation by CD1”, “Regulation of IL-2 Expression in Activated and Anergic T Lymphocytes”, and “T Cell Receptor Signaling”, alongside the negative regulator of NF-κB “NFKBIE Signaling Pathway” are also inhibited, while the anti-inflammatory “CTLA4” and “Oxytocin” signaling pathways are activated, together with the the “Platelet homeostasis” (Supplementary Table S7).

We confirm transcripts in MDD patients with CRP>1 mg/L (both currently depressed and responders) are consistently enriched for immunometabolic functions compared with controls and patients with CRP<1 mg/L, including the aforementioned immune/defensive and mitochondria-related processes (described in CRP-based groups with CRP>1, see also Supplementary Table S8). An enrichment in immune-related processes is also present in patients with CRP<1 vs. controls, primarily involving the acquired immune system. In particular, in current MDD we observe enrichment in biological processes involving “adaptive” and “B cell mediated” immunity, “antigen binding” molecular function, and “IgG immunoglobulin complex” cellular complex; while in responders we observe enrichment in “T cell receptor complex”. When comparing responders with current MDD with similar levels of CRP, both comparisons are enriched in immune-related genes. Notably, in responders vs. currently depressed with CRP>1 mg/L, we mainly observe processes related to adaptive immunity; while in comparison between patients with CRP<1 mg/L, additional metabolic processes are present, largely centred on energy production, including cellular respiration, ATP generation, and NAD(P)H activity (Table S8).

# ***Supplementary Table S1:*** *Clinical and sociodemographic characteristics (treatment-based grouping)*

|  | **MDD treatment responders**  **n= 37** | **MDD serum treatment non-responders**  **n= 47** | **MDD unmedicated**  **n= 22** | **Controls**  **n= 34** | **Group tests (Statistics and p values)**  *and post-hoc analyses* |
| --- | --- | --- | --- | --- | --- |
| **Serum hsCRP (mg/L)**  *mean (± SD)* | n=36  2.01 (±2.23) | n=47  2.72 (±2.59) | n=22  2.61 (±3.03) | n=34  1.04 (±0.88) | **H=11.469, p<.009**  MDD non-responders vs. controls |
| **Age (years)**  *mean (± SD)* | n=37  35.30 (±8.31) | n=47  36.40 (±7.53) | n=22  35.82 (±6.54) | n=34  35.85 (±7.96) | F=0.144, p=.934 |
| **Sex**  *n (%)* | n=37  Females: 26 (70.27%) | n=47  Females: 32 (68.09%) | n=22  Females: 15 (68.18%) | n=34  Females: 22 (64.71%) | χ^2^=0.256*^*^*, p=.968 |
| **Ethnicity**  *n (%)* | n=37  White: 33 (89.19%) | n=46  White: 41 (89.13%) | n=21  White: 18 (85.71%) | n=34  White: 21 (61.76%) | **χ2=12.445**,**  **p=.006** |
| **BMI (Kg/m^2^)**  *mean (± SD)* | n=36  27.72 (±5.70) | n=46  27.29 (±4.03) | n=22  27.01 (±3.75) | n=33  24.52 (±4.03) | **F=3.519, p=.017**  MDD responders vs. controls  MDD non-responders vs. controls |
| **CRP-based MDD groups**  *n (%)* | n=36  MDD CRP <1: 15 (41.67%)  MDD CRP 1-3: 12 (33.34%)  MDD CRP >3: 9 (25%) | n=47  MDD CRP <1: 17 (36.17%)  MDD CRP 1-3: 10 (21.28%)  MDD CRP >3: 20 (42.55%) | n=22  MDD CRP <1: 7 (31.82%)  MDD CRP 1-3: 9 (40.91%)  MDD CRP >3: 6 (27.27%) | n=34 | χ^2^=4.795*^*^*, p=.309  *Across the MDD subgroups only (no controls)* |
| **HAM-D17**  *mean (± SD)* | n=37  3.30 (±1.97) | n=47  18.64 (±3.33) | n=22  17.45 (±3.41) | n=34  0.53 (±0.96) | **H=72.954, p<.001**  *Across the MDD subgroups only (no controls)*    MDD responders vs. MDD non-responders and MDD unmedicated |
| BMI=body mass index; CRP=C-reactive protein; HAM-D17= Hamilton Rating Scale for Depression (17-item); Hs=high-sensitivity; IL= interleukin.  SD=standard deviation; F=ANOVA *F* value; H=Kruskal–Wallis *H* value; post-hoc analyses use Bonferroni correction (specific groups reported have statistically different mean scores (larger or smaller) compared with others); χ^2^ =Pearson Chi-Square; ^*^0%; ^**^12.5% expected count less than 5. Significant tests (p < 0.05) are in bold.  *I**n this grouping, one participant has been included who was not part of the CRP-based main analyses due to missing CRP values.* | | | | | |

# ***Supplementary Table S2:*** *mRNA transcripts differentially expressed (FDR-adjusted) in CRP-based MDD cases and controls*

| *Gene name* | *Gene symbol* | *log2FC* | *p-value* | *FDR-adjusted p-value* |
| --- | --- | --- | --- | --- |
| **CRP-based** | | | | |
| ***MDD CRP <1 mg/L vs. controls*** | | | | |
| // | // | // | // | // |
| ***MDD CRP 1-3 mg/L vs. controls*** | | | | |
| ubiquitin specific peptidase 18 | USP18 | 1.048721 | 5.69E-06 | 0.030232 |
| ISG15 ubiquitin like modifier | ISG15 | 1.043447 | 1.87E-05 | 0.053533 |
| translocase of inner mitochondrial membrane 10 | TIMM10 | 0.524324 | 2.01E-05 | 0.053533 |
| sialic acid binding Ig like lectin 1 | SIGLEC1 | 1.130685 | 2.85E-05 | 0.060527 |
| zinc finger protein 429 | ZNF429 | -0.44132 | 5.17E-05 | 0.091585 |
| 2'-5'-oligoadenylate synthetase 2 | OAS2 | 0.569818 | 0.000107 | 0.098423 |
| EIF3J divergent transcript | EIF3J-DT | -0.29556 | 0.000111 | 0.098423 |
| TNF superfamily member 10 | TNFSF10 | 0.445487 | 0.000119 | 0.098423 |
| 2'-5'-oligoadenylate synthetase 1 | OAS1 | 0.678947 | 0.000123 | 0.098423 |
| SP140 nuclear body protein | SP140 | 0.282493 | 0.000127 | 0.098423 |
| immunoglobulin heavy constant gamma 3 (G3m marker) | IGHG3 | -0.82309 | 0.00013 | 0.098423 |
| radical S-adenosyl methionine domain containing 2 | RSAD2 | 1.187176 | 0.00015 | 0.098423 |
| agrin | AGRN | 0.78646 | 0.000161 | 0.098423 |
| poly(ADP-ribose) polymerase family member 14 | PARP14 | 0.46569 | 0.0002 | 0.098423 |
| 2'-5'-oligoadenylate synthetase 3 | OAS3 | 0.852505 | 0.0002 | 0.098423 |
| synthesis of cytochrome C oxidase 2 | SCO2 | 0.418505 | 0.000226 | 0.098423 |
| receptor transporter protein 4 | RTP4 | 0.477916 | 0.000229 | 0.098423 |
| interleukin 1 receptor antagonist | IL1RN | 0.436944 | 0.000243 | 0.098423 |
| phospholipid scramblase 1 | PLSCR1 | 0.476944 | 0.000251 | 0.098423 |
| cytidine/uridine monophosphate kinase 2 | CMPK2 | 0.909677 | 0.000252 | 0.098423 |
| polyribonucleotide nucleotidyltransferase 1 | PNPT1 | 0.476673 | 0.000253 | 0.098423 |
| interferon induced protein 44 like | IFI44L | 1.031291 | 0.000256 | 0.098423 |
| lymphocyte antigen 6 family member E | LY6E | 0.604928 | 0.000259 | 0.098423 |
| ***MDD CRP >3 mg/L vs. controls*** | | | | |
| caspase recruitment domain family member 16 | CARD16 | 0.788494 | 3.01E-10 | 1.91E-06 |
| S100 calcium binding protein A8 | S100A8 | 0.790451 | 3.22E-10 | 1.91E-06 |
| caspase 4 | CASP4 | 0.326899 | 5.95E-10 | 2.36E-06 |
| S100 calcium binding protein A9 | S100A9 | 0.393401 | 4.23E-09 | 1.26E-05 |
| small nuclear ribonucleoprotein polypeptide G | SNRPG | 0.550026 | 1.53E-08 | 3.65E-05 |
| S100 calcium binding protein A12 | S100A12 | 0.742573 | 1.91E-08 | 3.79E-05 |
| annexin A3 | ANXA3 | 0.688344 | 8.92E-08 | 0.000137 |
| thymosin beta 10 | TMSB10 | 0.280701 | 9.24E-08 | 0.000137 |
| peroxiredoxin 1 | PRDX1 | 0.285243 | 1.83E-07 | 0.000242 |
| glutaredoxin | GLRX | 0.302234 | 2.16E-07 | 0.000257 |
| cystatin A | CSTA | 0.592024 | 2.60E-07 | 0.000282 |
| MT-ND1 pseudogene 23 | MTND1P23 | -2.35021 | 5.09E-07 | 0.000504 |
| ubiquinol-cytochrome c reductase hinge protein | UQCRH | 0.459735 | 1.25E-06 | 0.001134 |
| ribosomal protein L36a pseudogene 37 | RPL36AP37 | 0.888193 | 1.62E-06 | 0.001134 |
| NADH:ubiquinone oxidoreductase subunit S5 | NDUFS5 | 0.549154 | 1.62E-06 | 0.001134 |
| BCL2 related protein A1 | BCL2A1 | 0.470941 | 1.83E-06 | 0.001207 |
| chemokine like factor | CKLF | 0.283545 | 2.09E-06 | 0.001308 |
| cornichon family AMPA receptor auxiliary protein 4 | CNIH4 | 0.351792 | 2.36E-06 | 0.001406 |
| ubiquinol-cytochrome c reductase binding protein | UQCRB | 0.874028 | 2.84E-06 | 0.001611 |
| lysophosphatidic acid receptor 6 | LPAR6 | 0.399704 | 3.43E-06 | 0.001702 |
| plasminogen receptor with a C-terminal lysine | PLGRKT | 0.295478 | 3.45E-06 | 0.001702 |
| ribosomal protein S25 | RPS25 | 0.467541 | 3.46E-06 | 0.001702 |
| ribosomal protein L41 | RPL41 | 0.593219 | 3.78E-06 | 0.001702 |
| haptoglobin | HP | 1.013289 | 3.96E-06 | 0.001702 |
| translocase of outer mitochondrial membrane 7 | TOMM7 | 0.606554 | 4.10E-06 | 0.001702 |
| toll like receptor 5 | TLR5 | 0.458599 | 6.05E-06 | 0.002182 |
| ATP synthase membrane subunit g | ATP5MG | 0.299096 | 6.47E-06 | 0.002264 |
| cathelicidin antimicrobial peptide | CAMP | 1.003492 | 7.24E-06 | 0.002266 |
| SUB1 regulator of transcription | SUB1 | 0.404368 | 7.74E-06 | 0.002337 |
| matrix metallopeptidase 8 | MMP8 | 1.551673 | 7.86E-06 | 0.002337 |
| lamin B1 | LMNB1 | 0.290122 | 8.25E-06 | 0.002394 |
| NADH:ubiquinone oxidoreductase subunit A1 | NDUFA1 | 0.485334 | 8.71E-06 | 0.002441 |
| small integral membrane protein 4 | SMIM4 | 0.63884 | 9.24E-06 | 0.002498 |
| cytochrome c oxidase subunit 7A2 | COX7A2 | 0.300151 | 1.04E-05 | 0.002684 |
| NADH:ubiquinone oxidoreductase subunit B3 | NDUFB3 | 0.487002 | 1.06E-05 | 0.002684 |
| ring-box 1 | RBX1 | 0.311811 | 1.13E-05 | 0.002746 |
| C-type lectin domain family 2 member B | CLEC2B | 0.38024 | 1.20E-05 | 0.002866 |
| ATP synthase F1 subunit epsilon | ATP5F1E | 0.369084 | 1.61E-05 | 0.00349 |
| small nuclear ribonucleoprotein D2 polypeptide | SNRPD2 | 0.364588 | 1.75E-05 | 0.00372 |
| thioredoxin | TXN | 0.36278 | 1.94E-05 | 0.00388 |
| cytochrome c oxidase subunit 7B | COX7B | 0.488031 | 1.95E-05 | 0.00388 |
| glycerol kinase | GK | 0.323751 | 2.09E-05 | 0.00388 |
| Fc gamma receptor Ia | FCGR1A | 0.723715 | 2.09E-05 | 0.00388 |
| ribosomal protein L37 | RPL37 | 0.411368 | 2.10E-05 | 0.00388 |
| ribosomal protein L36a | RPL36A | 0.755769 | 2.12E-05 | 0.00388 |
| ribosomal protein L26 | RPL26 | 0.715118 | 2.12E-05 | 0.00388 |
| ubiquinol-cytochrome c reductase complex III subunit VII | UQCRQ | 0.386392 | 2.23E-05 | 0.003956 |
| glia maturation factor gamma | GMFG | 0.265133 | 2.38E-05 | 0.004113 |
| myosin light chain 6 | MYL6 | 0.300877 | 2.57E-05 | 0.004362 |
| hydroxycarboxylic acid receptor 3 | HCAR3 | 0.409878 | 2.70E-05 | 0.004523 |
| killer cell immunoglobulin like receptor, two Ig domains and long cytoplasmic tail 3 | KIR2DL3 | -0.86053 | 3.06E-05 | 0.004857 |
| TRAF interacting protein with forkhead associated domain | TIFA | 0.344599 | 3.18E-05 | 0.004906 |
| lipocalin 2 | LCN2 | 1.172139 | 3.33E-05 | 0.005075 |
| selenoprotein K | SELENOK | 0.378327 | 3.58E-05 | 0.005392 |
| bolA family member 2 | BOLA2 | 0.278389 | 3.76E-05 | 0.005589 |
| cytochrome c oxidase assembly factor COX14 | COX14 | 0.282544 | 3.81E-05 | 0.005594 |
| ribosomal protein L24 | RPL24 | 0.362785 | 3.97E-05 | 0.005756 |
| ribosomal protein S29 | RPS29 | 0.475621 | 4.27E-05 | 0.006038 |
| mitochondrial ribosomal protein S33 | MRPS33 | 0.440359 | 4.42E-05 | 0.006038 |
| ATP synthase peripheral stalk subunit F6 | ATP5PF | 0.321782 | 4.57E-05 | 0.006179 |
| mitochondrial ribosomal protein L39 | MRPL39 | 0.283904 | 4.72E-05 | 0.006308 |
| absent in melanoma 2 | AIM2 | 0.491705 | 5.00E-05 | 0.006537 |
| ribosomal protein S27 | RPS27 | 0.476034 | 5.00E-05 | 0.006537 |
| tripartite motif containing 25 | TRIM25 | 0.27033 | 5.26E-05 | 0.006808 |
| ribosomal protein L23 | RPL23 | 0.534644 | 5.32E-05 | 0.006809 |
| ribosomal protein L21 | RPL21 | 0.53392 | 5.51E-05 | 0.006931 |
| ATP synthase membrane subunit e | ATP5ME | 0.436105 | 5.71E-05 | 0.006984 |
| proteasome 20S subunit alpha 3 | PSMA3 | 0.275994 | 6.43E-05 | 0.007439 |
| ribosomal protein L36a like | RPL36AL | 0.317053 | 6.60E-05 | 0.007555 |
| ribosomal protein S27 like | RPS27L | 0.4457 | 7.04E-05 | 0.007831 |
| ribosomal protein L39 | RPL39 | 0.579131 | 7.47E-05 | 0.00823 |
| ribosomal protein L22 | RPL22 | 0.406821 | 7.78E-05 | 0.008282 |
| acyl-CoA synthetase long chain family member 1 | ACSL1 | 0.29749 | 8.70E-05 | 0.008775 |
| ribosomal protein L31 | RPL31 | 0.629101 | 8.96E-05 | 0.008959 |
| cytochrome c oxidase subunit 7C | COX7C | 0.429794 | 9.95E-05 | 0.009648 |
| Fc gamma receptor Ib, pseudogene | FCGR1BP | 0.619681 | 0.00011 | 0.010416 |
| ribosomal protein L35 | RPL35 | 0.308622 | 0.000119 | 0.010648 |
| ATP synthase peripheral stalk subunit OSCP | ATP5PO | 0.319131 | 0.000119 | 0.010648 |
| BCL6 transcription repressor | BCL6 | 0.308301 | 0.000123 | 0.010868 |
| caspase 5 | CASP5 | 0.628894 | 0.000125 | 0.010873 |
| proteasome 20S subunit alpha 2 | PSMA2 | 0.305784 | 0.000128 | 0.010967 |
| membrane bound O-acyltransferase domain containing 2 | MBOAT2 | 0.28774 | 0.000129 | 0.010967 |
| ribosomal protein L34 | RPL34 | 0.673941 | 0.000132 | 0.011112 |
| immunoglobulin heavy variable 4-39 | IGHV4-39 | 1.131578 | 0.000135 | 0.01121 |
| hydroxycarboxylic acid receptor 2 | HCAR2 | 0.34771 | 0.000139 | 0.011233 |
| diazepam binding inhibitor, acyl-CoA binding protein | DBI | 0.357681 | 0.000139 | 0.011233 |
| Fc epsilon receptor Ig | FCER1G | 0.274192 | 0.000141 | 0.011233 |
| ribosomal protein S24 | RPS24 | 0.580275 | 0.000143 | 0.011332 |
| ferredoxin reductase | FDXR | -0.267 | 0.000145 | 0.011405 |
| dedicator of cytokinesis 4 | DOCK4 | 0.563777 | 0.000148 | 0.011573 |
| FA core complex associated protein 24 | FAAP24 | -0.50565 | 0.00015 | 0.011694 |
| olfactomedin 4 | OLFM4 | 1.545009 | 0.000151 | 0.011702 |
| DNA polymerase epsilon 4, accessory subunit | POLE4 | 0.364053 | 0.000156 | 0.011903 |
| mitochondrial ribosomal protein L51 | MRPL51 | 0.327008 | 0.000157 | 0.011903 |
| elongation factor for RNA polymerase II 2 | ELL2 | 0.477519 | 0.000159 | 0.011943 |
| lymphocyte antigen 96 | LY96 | 0.407431 | 0.00016 | 0.011943 |
| MT-CO1 pseudogene 12 | MTCO1P12 | -1.03682 | 0.000161 | 0.011994 |
| CEA cell adhesion molecule 6 | CEACAM6 | 1.327699 | 0.000166 | 0.012301 |
| golgin A8 family member O | GOLGA8O | -0.52097 | 0.000173 | 0.012416 |
| SEC11 homolog C, signal peptidase complex subunit | SEC11C | 0.336893 | 0.000175 | 0.012416 |
| zinc finger protein 431 | ZNF431 | -0.40366 | 0.000175 | 0.012416 |
| prefoldin subunit 5 | PFDN5 | 0.470076 | 0.000187 | 0.013074 |
| ribosomal protein S3A | RPS3A | 0.479581 | 0.000188 | 0.013074 |
| RB transcriptional corepressor 1 | RB1 | 0.300494 | 0.000191 | 0.013074 |
| ribosomal protein L21 pseudogene 119 | RPL21P119 | 0.717798 | 0.000199 | 0.013426 |
| ribosomal protein S27a | RPS27A | 0.398573 | 0.000203 | 0.013596 |
| ribosomal protein L17 | RPL17 | 0.426721 | 0.000203 | 0.013596 |
| translation machinery associated 7 homolog | TMA7 | 0.404805 | 0.000216 | 0.01433 |
| COMM domain containing 6 | COMMD6 | 0.501985 | 0.000219 | 0.014433 |
| eukaryotic translation elongation factor 1 beta 2 | EEF1B2 | 0.422023 | 0.00022 | 0.014433 |
| cytochrome c oxidase assembly factor COX16 | COX16 | 0.479801 | 0.000223 | 0.014561 |
| A-kinase anchoring protein 12 | AKAP12 | -1.02007 | 0.000226 | 0.014585 |
| tubulin tyrosine ligase like 5 | TTLL5 | -0.37 | 0.000228 | 0.014646 |
| PET100 cytochrome c oxidase chaperone | PET100 | 0.361252 | 0.00023 | 0.014673 |
| lactotransferrin | LTF | 1.205057 | 0.000231 | 0.014673 |
| cytochrome c oxidase copper chaperone COX17 | COX17 | 0.273562 | 0.000237 | 0.014877 |
| cellular communication network factor 3 | CCN3 | -0.4389 | 0.000238 | 0.014877 |
| tumor protein, translationally-controlled 1 | TPT1 | 0.337113 | 0.000246 | 0.015059 |
| signal recognition particle 14 | SRP14 | 0.266077 | 0.000258 | 0.015638 |
| ribosomal protein S20 | RPS20 | 0.327903 | 0.000266 | 0.015955 |
| ribosomal protein S17 | RPS17 | 0.426801 | 0.000268 | 0.015989 |
| replication protein A3 | RPA3 | 0.280814 | 0.00027 | 0.015989 |
| bactericidal permeability increasing protein | BPI | 0.895953 | 0.00027 | 0.015989 |
| ribosomal protein S23 | RPS23 | 0.403257 | 0.000274 | 0.01613 |
| tigger transposable element derived 3 | TIGD3 | -0.30529 | 0.000295 | 0.016794 |
| ribosomal protein S7 | RPS7 | 0.434668 | 0.000297 | 0.016819 |
| Fc gamma receptor Ic, pseudogene | FCGR1CP | 0.7425 | 0.000301 | 0.016881 |
| histidine triad nucleotide binding protein 1 | HINT1 | 0.294722 | 0.000303 | 0.016906 |
| ER membrane protein complex subunit 3 | EMC3 | 0.277327 | 0.000329 | 0.018048 |
| adenylate kinase 6 | AK6 | 0.526229 | 0.000347 | 0.018847 |
| ubiquinol-cytochrome c reductase, complex III subunit XI | UQCR11 | 0.272979 | 0.000363 | 0.019464 |
| ATP synthase membrane subunit j | ATP5MJ | 0.312492 | 0.000371 | 0.019654 |
| brain expressed X-linked 2 | BEX2 | 0.369448 | 0.000385 | 0.019781 |
| ribosomal protein L11 | RPL11 | 0.396768 | 0.000427 | 0.021138 |
| ribosomal protein L35a | RPL35A | 0.337217 | 0.00043 | 0.021138 |
| ribosomal protein S21 | RPS21 | 0.351153 | 0.000433 | 0.021138 |
| ZNFX1 antisense RNA 1 | ZFAS1 | 0.33009 | 0.000434 | 0.021138 |
| ribosomal protein L7 | RPL7 | 0.388682 | 0.000437 | 0.021143 |
| translocase of outer mitochondrial membrane 5 | TOMM5 | 0.327986 | 0.00044 | 0.021176 |
| CEA cell adhesion molecule 8 | CEACAM8 | 1.210356 | 0.000455 | 0.021652 |
| ribosomal protein S18 | RPS18 | 0.333248 | 0.000485 | 0.022586 |
| ribosomal protein S15a | RPS15A | 0.427383 | 0.000496 | 0.02289 |
| ribosomal protein S14 | RPS14 | 0.269617 | 0.0005 | 0.022961 |
| ribosomal protein L30 | RPL30 | 0.269672 | 0.000526 | 0.023644 |
| CD59 molecule (CD59 blood group) | CD59 | 0.286931 | 0.000531 | 0.023644 |
| ENY2 transcription and export complex 2 subunit | ENY2 | 0.307447 | 0.00054 | 0.023993 |
| DnaJ heat shock protein family (Hsp40) member C15 | DNAJC15 | 0.411966 | 0.000571 | 0.025066 |
| methyltransferase 5, N6-adenosine | METTL5 | 0.278054 | 0.000593 | 0.025789 |
| perilipin 4 | PLIN4 | 0.335664 | 0.000607 | 0.026163 |
| CD180 molecule | CD180 | 0.311474 | 0.000616 | 0.026217 |
| tubulin folding cofactor A | TBCA | 0.288851 | 0.000619 | 0.026217 |
| kringle containing transmembrane protein 1 | KREMEN1 | 0.504643 | 0.000623 | 0.026272 |
| ankyrin repeat domain 22 | ANKRD22 | 0.814224 | 0.000633 | 0.026624 |
| TNF superfamily member 10 | TNFSF10 | 0.342282 | 0.000657 | 0.027313 |
| TEN1-CDK3 readthrough (NMD candidate) | TEN1-CDK3 | -0.3615 | 0.000675 | 0.027886 |
| HAUS augmin like complex subunit 1 | HAUS1 | 0.286274 | 0.000686 | 0.028239 |
| killer cell immunoglobulin like receptor, two Ig domains and long cytoplasmic tail 1 | KIR2DL1 | -0.76821 | 0.000692 | 0.028373 |
| ectodysplasin A | EDA | 0.591667 | 0.000719 | 0.029183 |
| microtubule associated protein 7 | MAP7 | -0.4428 | 0.000748 | 0.030154 |
| mitochondrial ribosomal protein L22 | MRPL22 | 0.322716 | 0.000771 | 0.030982 |
| cytochrome c oxidase subunit 6C | COX6C | 0.407139 | 0.000792 | 0.03151 |
| tumor protein p53 inducible nuclear protein 2 | TP53INP2 | -0.29918 | 0.000795 | 0.03151 |
| LSM3 homolog, U6 small nuclear RNA and mRNA degradation associated | LSM3 | 0.361572 | 0.000802 | 0.031685 |
| ribosomal protein L27 | RPL27 | 0.344951 | 0.000804 | 0.031685 |
| ribosomal protein S12 | RPS12 | 0.281145 | 0.000816 | 0.032055 |
| cysteine rich secretory protein 3 | CRISP3 | 0.935492 | 0.000848 | 0.033008 |
| translocase of inner mitochondrial membrane 8 homolog B | TIMM8B | 0.356199 | 0.000865 | 0.033428 |
| ribosomal protein S10 | RPS10 | 0.299196 | 0.000907 | 0.034591 |
| NADH:ubiquinone oxidoreductase subunit S4 | NDUFS4 | 0.389838 | 0.000938 | 0.035271 |
| membrane spanning 4-domains A4A | MS4A4A | 0.472535 | 0.000953 | 0.035545 |
| RAB13, member RAS oncogene family | RAB13 | 0.427384 | 0.000993 | 0.036339 |
| CD274 molecule | CD274 | 0.545276 | 0.001019 | 0.037045 |
| defective in cullin neddylation 1 domain containing 1 | DCUN1D1 | 0.281605 | 0.001021 | 0.037045 |
| SH3 and PX domains 2A | SH3PXD2A | 0.309447 | 0.001066 | 0.038085 |
| Rho GTPase activating protein 11A | ARHGAP11A | 0.598404 | 0.001067 | 0.038085 |
| mitochondrial ribosomal protein L47 | MRPL47 | 0.361587 | 0.001071 | 0.038085 |
| NADH:ubiquinone oxidoreductase subunit A6 | NDUFA6 | 0.267695 | 0.001076 | 0.038089 |
| growth arrest specific 6 | GAS6 | 0.33521 | 0.001079 | 0.038089 |
| solute carrier family 45 member 3 | SLC45A3 | -0.59237 | 0.001095 | 0.038539 |
| TNF alpha induced protein 6 | TNFAIP6 | 0.526239 | 0.001139 | 0.039738 |
| eukaryotic translation initiation factor 2 subunit alpha | EIF2S1 | 0.274928 | 0.001151 | 0.039924 |
| SAP domain containing ribonucleoprotein | SARNP | 0.331048 | 0.001187 | 0.040987 |
| phospholipid scramblase 1 | PLSCR1 | 0.415398 | 0.001231 | 0.042327 |
| YES proto-oncogene 1, Src family tyrosine kinase | YES1 | -0.33182 | 0.001248 | 0.04262 |
| popeye domain containing 2 | POPDC2 | 0.421925 | 0.001292 | 0.043566 |
| CDC28 protein kinase regulatory subunit 1B | CKS1B | 0.294174 | 0.001433 | 0.046438 |
| allograft inflammatory factor 1 | AIF1 | 0.264038 | 0.001469 | 0.047194 |
| dual specificity phosphatase 2 | DUSP2 | -0.39157 | 0.001476 | 0.047194 |
| myosin light chain 6B | MYL6B | 0.363137 | 0.001591 | 0.049685 |
| ribosomal protein L9 pseudogene 8 | RPL9P8 | 0.886148 | 0.001603 | 0.049937 |
| ribosomal protein S6 | RPS6 | 0.276273 | 0.001629 | 0.050447 |
| ATP binding cassette subfamily A member 7 | ABCA7 | -0.27256 | 0.001647 | 0.050768 |
| cytochrome c oxidase assembly factor 6 | COA6 | 0.273285 | 0.00177 | 0.053718 |
| arginase 1 | ARG1 | 0.620323 | 0.001808 | 0.054588 |
| long intergenic non-protein coding RNA 1410 | LINC01410 | 0.341784 | 0.001835 | 0.054752 |
| uncharacterized LOC100130357 | LOC100130357 | -0.45669 | 0.001845 | 0.054752 |
| LMBR1 domain containing 2 | LMBRD2 | -0.3428 | 0.001845 | 0.054752 |
| signal transducing adaptor family member 1 | STAP1 | 0.318523 | 0.001901 | 0.055546 |
| RAP1 GTPase activating protein | RAP1GAP | 1.291666 | 0.001939 | 0.056266 |
| RAD50 double strand break repair protein | RAD50 | 0.345447 | 0.001953 | 0.05653 |
| zinc finger DHHC-type palmitoyltransferase 2 | ZDHHC2 | 0.32536 | 0.002027 | 0.057691 |
| zinc finger protein 155 | ZNF155 | -0.31811 | 0.002078 | 0.058724 |
| mast cell expressed membrane protein 1 | MCEMP1 | 0.313481 | 0.00213 | 0.059759 |
| calpain 5 | CAPN5 | -0.30344 | 0.002199 | 0.060849 |
| small nuclear ribonucleoprotein D1 polypeptide | SNRPD1 | 0.433962 | 0.002242 | 0.061091 |
| ribosomal protein L26 like 1 | RPL26L1 | 0.331663 | 0.002248 | 0.061091 |
| mitochondrial ribosomal protein S18C | MRPS18C | 0.348334 | 0.002298 | 0.061459 |
| non-SMC condensin II complex subunit G2 | NCAPG2 | 0.735417 | 0.002324 | 0.061459 |
| interleukin 2 receptor subunit alpha | IL2RA | 0.304591 | 0.002335 | 0.061459 |
| alpha hemoglobin stabilizing protein | AHSP | 0.654274 | 0.002602 | 0.066427 |
| small nucleolar RNA host gene 8 | SNHG8 | 0.401895 | 0.002641 | 0.06728 |
| prostaglandin D2 synthase | PTGDS | -0.5324 | 0.002812 | 0.069555 |
| peptidyl-tRNA hydrolase domain containing 1 | PTRHD1 | 0.272788 | 0.002902 | 0.070966 |
| CD248 molecule | CD248 | -0.46785 | 0.002919 | 0.070966 |
| ribosomal protein L13a pseudogene 5 | RPL13AP5 | 0.344068 | 0.002971 | 0.071541 |
| prokineticin 2 | PROK2 | 0.349129 | 0.002986 | 0.071626 |
| perilipin 5 | PLIN5 | 0.319729 | 0.003015 | 0.071888 |
| immunoglobulin heavy variable 3-49 | IGHV3-49 | -0.9803 | 0.003085 | 0.07293 |
| NADH:ubiquinone oxidoreductase subunit B1 | NDUFB1 | 0.271281 | 0.00309 | 0.07293 |
| centromere protein N | CENPN | 0.359507 | 0.003262 | 0.075494 |
| interleukin 3 receptor subunit alpha | IL3RA | -0.5427 | 0.003337 | 0.076781 |
| ribosome production factor 2 homolog | RPF2 | 0.34358 | 0.00354 | 0.079774 |
| B and T lymphocyte associated | BTLA | 0.32937 | 0.003637 | 0.081488 |
| defensin alpha 3 | DEFA3 | 1.369377 | 0.003813 | 0.083594 |
| YjeF N-terminal domain containing 3 | YJEFN3 | -0.28438 | 0.003815 | 0.083594 |
| mitochondrial ribosomal protein S28 | MRPS28 | 0.28873 | 0.004024 | 0.086718 |
| small nuclear ribonucleoprotein polypeptide E | SNRPE | 0.339519 | 0.004145 | 0.088061 |
| glutathione peroxidase 3 | GPX3 | -0.38251 | 0.004238 | 0.089529 |
| RAB15, member RAS oncogene family | RAB15 | -0.3677 | 0.004244 | 0.089529 |
| tetratricopeptide repeat domain 24 | TTC24 | -0.38252 | 0.004271 | 0.089624 |
| reticulon 2 | RTN2 | 0.273709 | 0.004479 | 0.092673 |
| tetratricopeptide repeat domain 28 | TTC28 | -0.49498 | 0.004566 | 0.09334 |
| desmocollin 2 | DSC2 | 0.504975 | 0.004816 | 0.096784 |
| membrane spanning 4-domains A2 | MS4A2 | -0.73191 | 0.005021 | 0.098726 |
| coiled-coil domain containing 167 | CCDC167 | 0.269581 | 0.005212 | 0.099879 |
| ***MDD CRP >3 mg/L vs. MDD CRP <1 mg/L*** | | | | |
| S100 calcium binding protein A9 | S100A9 | 0.560042 | 3.63E-14 | 5.46E-10 |
| annexin A3 | ANXA3 | 1.030537 | 1.49E-13 | 1.12E-09 |
| caspase 4 | CASP4 | 0.407106 | 8.37E-12 | 4.20E-08 |
| S100 calcium binding protein A12 | S100A12 | 0.889569 | 1.64E-11 | 5.88E-08 |
| S100 calcium binding protein A8 | S100A8 | 0.888458 | 1.95E-11 | 5.88E-08 |
| WW domain binding protein 11 | WBP11 | -0.28511 | 1.10E-10 | 2.77E-07 |
| nicotinamide phosphoribosyltransferase | NAMPT | 0.478137 | 2.67E-10 | 5.74E-07 |
| BCL6 transcription repressor | BCL6 | 0.530724 | 3.69E-10 | 6.94E-07 |
| kringle containing transmembrane protein 1 | KREMEN1 | 0.92254 | 4.36E-10 | 7.29E-07 |
| zinc finger MIZ-type containing 1 | ZMIZ1 | -0.3106 | 7.35E-10 | 1.11E-06 |
| chemokine like factor | CKLF | 0.404964 | 9.00E-10 | 1.15E-06 |
| mast cell expressed membrane protein 1 | MCEMP1 | 0.664677 | 9.13E-10 | 1.15E-06 |
| serglycin | SRGN | 0.412265 | 1.24E-09 | 1.44E-06 |
| lamin B1 | LMNB1 | 0.415934 | 1.93E-09 | 2.07E-06 |
| BCL2 related protein A1 | BCL2A1 | 0.656961 | 2.88E-09 | 2.89E-06 |
| absent in melanoma 2 | AIM2 | 0.664896 | 4.38E-09 | 4.03E-06 |
| vanin 2 | VNN2 | 0.548741 | 4.54E-09 | 4.03E-06 |
| beta-2-microglobulin | B2M | 0.374068 | 6.65E-09 | 5.22E-06 |
| lymphocyte antigen 96 | LY96 | 0.679882 | 6.87E-09 | 5.22E-06 |
| acyl-CoA synthetase long chain family member 1 | ACSL1 | 0.477357 | 7.08E-09 | 5.22E-06 |
| F-box and leucine rich repeat protein 5 | FBXL5 | 0.274168 | 7.28E-09 | 5.22E-06 |
| STEAP4 metalloreductase | STEAP4 | 0.403617 | 8.38E-09 | 5.74E-06 |
| Fc gamma receptor Ia | FCGR1A | 1.003378 | 1.01E-08 | 6.59E-06 |
| haptoglobin | HP | 1.228747 | 2.42E-08 | 1.30E-05 |
| bone marrow stromal cell antigen 1 | BST1 | 0.322974 | 2.43E-08 | 1.30E-05 |
| caspase recruitment domain family member 16 | CARD16 | 0.718974 | 2.51E-08 | 1.31E-05 |
| nucleic acid binding protein 1 | NABP1 | 0.33144 | 2.76E-08 | 1.34E-05 |
| signal peptide peptidase like 2A | SPPL2A | 0.279047 | 2.86E-08 | 1.35E-05 |
| NGFI-A binding protein 2 | NAB2 | -0.28424 | 3.52E-08 | 1.55E-05 |
| family with sequence similarity 157 member A | FAM157A | 0.491978 | 3.61E-08 | 1.55E-05 |
| zinc finger protein 438 | ZNF438 | 0.359356 | 3.61E-08 | 1.55E-05 |
| solute carrier family 26 member 8 | SLC26A8 | 0.822973 | 4.27E-08 | 1.79E-05 |
| SRSF protein kinase 1 | SRPK1 | 0.331724 | 7.63E-08 | 2.91E-05 |
| LIM domain kinase 2 | LIMK2 | 0.408453 | 7.75E-08 | 2.91E-05 |
| C1GALT1 specific chaperone 1 | C1GALT1C1 | 0.304787 | 9.12E-08 | 3.19E-05 |
| interleukin 4 receptor | IL4R | 0.389464 | 1.12E-07 | 3.85E-05 |
| Fc epsilon receptor Ig | FCER1G | 0.385768 | 1.30E-07 | 4.34E-05 |
| TRAF interacting protein with forkhead associated domain | TIFA | 0.433409 | 1.40E-07 | 4.60E-05 |
| solute carrier family 22 member 4 | SLC22A4 | 0.505026 | 1.53E-07 | 4.89E-05 |
| caspase 5 | CASP5 | 0.891122 | 1.74E-07 | 5.35E-05 |
| Fc gamma receptor Ib, pseudogene | FCGR1BP | 0.86236 | 1.85E-07 | 5.57E-05 |
| ATP synthase membrane subunit g | ATP5MG | 0.358762 | 2.14E-07 | 6.19E-05 |
| SH3 domain containing GRB2 like, endophilin B1 | SH3GLB1 | 0.292457 | 2.29E-07 | 6.50E-05 |
| MT-CO1 pseudogene 12 | MTCO1P12 | -1.40972 | 2.39E-07 | 6.66E-05 |
| cysteine rich transmembrane module containing 1 | CYSTM1 | 0.523725 | 2.47E-07 | 6.74E-05 |
| FAU ubiquitin like and ribosomal protein S30 fusion | FAU | 0.306258 | 3.91E-07 | 9.65E-05 |
| dysferlin | DYSF | 0.321677 | 4.53E-07 | 0.000108 |
| interleukin 3 receptor subunit alpha | IL3RA | -0.93746 | 4.71E-07 | 0.000111 |
| membrane bound O-acyltransferase domain containing 2 | MBOAT2 | 0.397113 | 5.10E-07 | 0.000118 |
| ring-box 1 | RBX1 | 0.368392 | 5.67E-07 | 0.000127 |
| cytochrome c oxidase subunit 7C | COX7C | 0.539214 | 6.54E-07 | 0.000141 |
| DnaJ heat shock protein family (Hsp40) member C15 | DNAJC15 | 0.602163 | 6.96E-07 | 0.000148 |
| prokineticin 2 | PROK2 | 0.598992 | 7.66E-07 | 0.000153 |
| reactive oxygen species modulator 1 | ROMO1 | 0.295589 | 7.75E-07 | 0.000153 |
| matrix metallopeptidase 8 | MMP8 | 1.74577 | 7.76E-07 | 0.000153 |
| glycerol kinase | GK | 0.424894 | 7.88E-07 | 0.000153 |
| tetratricopeptide repeat domain 28 | TTC28 | -0.81665 | 7.89E-07 | 0.000153 |
| cathelicidin antimicrobial peptide | CAMP | 1.161808 | 7.93E-07 | 0.000153 |
| COMM domain containing 3 | COMMD3 | 0.276749 | 8.44E-07 | 0.000157 |
| thioredoxin | TXN | 0.444584 | 8.58E-07 | 0.000157 |
| CD59 molecule (CD59 blood group) | CD59 | 0.417939 | 8.67E-07 | 0.000157 |
| Fas cell surface death receptor | FAS | 0.410178 | 8.84E-07 | 0.000157 |
| cornichon family AMPA receptor auxiliary protein 4 | CNIH4 | 0.369607 | 9.44E-07 | 0.000162 |
| NOP10 ribonucleoprotein | NOP10 | 0.296748 | 9.68E-07 | 0.000164 |
| CEA cell adhesion molecule 3 | CEACAM3 | 0.300835 | 1.00E-06 | 0.000168 |
| rhophilin associated tail protein 1 like | ROPN1L | 0.372135 | 1.07E-06 | 0.000174 |
| heat shock factor binding protein 1 | HSBP1 | 0.32549 | 1.27E-06 | 0.000199 |
| cytochrome c oxidase subunit 7A2 | COX7A2 | 0.339911 | 1.37E-06 | 0.000213 |
| beta-1,4-galactosyltransferase 5 | B4GALT5 | 0.293719 | 1.44E-06 | 0.000217 |
| ribosomal protein S29 | RPS29 | 0.539341 | 1.56E-06 | 0.000232 |
| glycogen phosphorylase L | PYGL | 0.291722 | 1.74E-06 | 0.000252 |
| Fc gamma receptor Ic, pseudogene | FCGR1CP | 1.018854 | 1.83E-06 | 0.000258 |
| myosin light chain 6 | MYL6 | 0.367718 | 1.96E-06 | 0.000268 |
| Fc alpha receptor | FCAR | 0.423593 | 2.00E-06 | 0.000268 |
| interferon alpha inducible protein 27 like 2 | IFI27L2 | 0.333191 | 2.07E-06 | 0.000271 |
| ribosomal protein L39 | RPL39 | 0.663449 | 2.14E-06 | 0.000273 |
| perilipin 4 | PLIN4 | 0.43482 | 2.16E-06 | 0.000273 |
| MT-ND1 pseudogene 23 | MTND1P23 | -2.17035 | 2.20E-06 | 0.000273 |
| ribosomal protein L41 | RPL41 | 0.60276 | 2.20E-06 | 0.000273 |
| maltase-glucoamylase 2 (putative) | MGAM2 | 0.694363 | 2.21E-06 | 0.000273 |
| small nuclear ribonucleoprotein polypeptide G | SNRPG | 0.454285 | 2.27E-06 | 0.000275 |
| lysophosphatidic acid receptor 6 | LPAR6 | 0.40652 | 2.35E-06 | 0.000276 |
| ubiquinol-cytochrome c reductase complex III subunit VII | UQCRQ | 0.4333 | 2.37E-06 | 0.000276 |
| ELL associated factor 2 | EAF2 | 0.667914 | 2.39E-06 | 0.000276 |
| glia maturation factor gamma | GMFG | 0.294869 | 2.40E-06 | 0.000276 |
| perilipin 5 | PLIN5 | 0.487496 | 2.66E-06 | 0.000299 |
| proteasome maturation protein | POMP | 0.264499 | 2.69E-06 | 0.0003 |
| cysteine rich secretory protein 3 | CRISP3 | 1.378275 | 2.81E-06 | 0.000309 |
| ATPase H+ transporting V1 subunit C1 | ATP6V1C1 | 0.305136 | 3.08E-06 | 0.000331 |
| ribosomal protein L35 | RPL35 | 0.355889 | 3.12E-06 | 0.000331 |
| coiled-coil domain containing 71 like | CCDC71L | 0.40501 | 3.65E-06 | 0.000382 |
| tumor protein p53 inducible nuclear protein 2 | TP53INP2 | -0.39472 | 3.76E-06 | 0.00039 |
| N-alpha-acetyltransferase 38, NatC auxiliary subunit | NAA38 | 0.284354 | 3.98E-06 | 0.000405 |
| ribosomal protein S10 | RPS10 | 0.397569 | 4.24E-06 | 0.00042 |
| oxidized low density lipoprotein receptor 1 | OLR1 | 1.579748 | 4.27E-06 | 0.00042 |
| ribosomal protein L30 | RPL30 | 0.324232 | 4.63E-06 | 0.000447 |
| ribosomal protein S21 | RPS21 | 0.435321 | 4.79E-06 | 0.000459 |
| ribosomal protein S18 | RPS18 | 0.405165 | 5.73E-06 | 0.000536 |
| glutaminyl-peptide cyclotransferase | QPCT | 0.343454 | 5.81E-06 | 0.00054 |
| glutaredoxin | GLRX | 0.293196 | 6.17E-06 | 0.000567 |
| cystatin A | CSTA | 0.528359 | 6.26E-06 | 0.000571 |
| ubiquitin like 5 | UBL5 | 0.273558 | 6.41E-06 | 0.000577 |
| tripartite motif containing 25 | TRIM25 | 0.310755 | 6.43E-06 | 0.000577 |
| small nuclear ribonucleoprotein D2 polypeptide | SNRPD2 | 0.375182 | 6.43E-06 | 0.000577 |
| hydroxycarboxylic acid receptor 3 | HCAR3 | 0.480177 | 6.78E-06 | 0.000597 |
| small nuclear ribonucleoprotein polypeptide E | SNRPE | 0.510951 | 7.04E-06 | 0.00061 |
| regulator of hemoglobinization and erythroid cell expansion | RHEX | -1.11442 | 7.40E-06 | 0.000634 |
| aquaporin 9 | AQP9 | 0.299353 | 7.79E-06 | 0.000659 |
| proteasome 20S subunit alpha 2 | PSMA2 | 0.363491 | 8.06E-06 | 0.000675 |
| ribosomal protein S15a | RPS15A | 0.537521 | 8.11E-06 | 0.000675 |
| alkaline phosphatase, biomineralization associated | ALPL | 0.586856 | 8.61E-06 | 0.000708 |
| MAX dimerization protein 3 | MXD3 | 0.289669 | 8.72E-06 | 0.000712 |
| potassium inwardly rectifying channel subfamily J member 15 | KCNJ15 | 0.462794 | 8.88E-06 | 0.000719 |
| SEC11 homolog C, signal peptidase complex subunit | SEC11C | 0.406102 | 9.09E-06 | 0.000728 |
| ribosomal protein L32 | RPL32 | 0.291488 | 9.45E-06 | 0.000745 |
| ubiquinol-cytochrome c reductase hinge protein | UQCRH | 0.427488 | 1.04E-05 | 0.000811 |
| SH3 domain binding protein 4 | SH3BP4 | -0.47988 | 1.06E-05 | 0.000814 |
| lipocalin 2 | LCN2 | 1.308511 | 1.07E-05 | 0.000818 |
| solute carrier family 11 member 1 | SLC11A1 | 0.348921 | 1.10E-05 | 0.000829 |
| dehydrogenase/reductase 13 | DHRS13 | 0.393677 | 1.10E-05 | 0.000829 |
| ribosomal protein L37 | RPL37 | 0.395929 | 1.12E-05 | 0.000842 |
| COMM domain containing 8 | COMMD8 | 0.310257 | 1.19E-05 | 0.000888 |
| ubiquinol-cytochrome c reductase, complex III subunit XI | UQCR11 | 0.368007 | 1.23E-05 | 0.000912 |
| long intergenic non-protein coding RNA 2009 | LINC02009 | 1.611879 | 1.26E-05 | 0.000924 |
| mitochondrial ribosomal protein L15 | MRPL15 | 0.331293 | 1.26E-05 | 0.000924 |
| toll like receptor 5 | TLR5 | 0.4407 | 1.41E-05 | 0.001009 |
| ribosomal protein S14 | RPS14 | 0.303333 | 1.44E-05 | 0.001025 |
| ribosomal protein S27 | RPS27 | 0.484094 | 1.59E-05 | 0.001091 |
| ribosomal protein S25 | RPS25 | 0.411752 | 1.65E-05 | 0.001121 |
| filamin B | FLNB | -0.27273 | 1.78E-05 | 0.001194 |
| leukocyte immunoglobulin like receptor A5 | LILRA5 | 0.451366 | 1.80E-05 | 0.001197 |
| SUB1 regulator of transcription | SUB1 | 0.415174 | 1.86E-05 | 0.001222 |
| NADH:ubiquinone oxidoreductase subunit B3 | NDUFB3 | 0.498203 | 1.87E-05 | 0.001222 |
| cut like homeobox 2 | CUX2 | -0.68946 | 1.87E-05 | 0.001222 |
| cellular communication network factor 3 | CCN3 | -0.49952 | 2.08E-05 | 0.001318 |
| splicing factor 3b subunit 6 | SF3B6 | 0.31072 | 2.10E-05 | 0.001322 |
| vesicle associated membrane protein 5 | VAMP5 | 0.320121 | 2.14E-05 | 0.001343 |
| grancalcin | GCA | 0.309695 | 2.18E-05 | 0.001365 |
| lin-7 homolog A, crumbs cell polarity complex component | LIN7A | 0.34976 | 2.25E-05 | 0.001376 |
| heat shock protein family B (small) member 11 | HSPB11 | 0.265694 | 2.26E-05 | 0.001376 |
| cytochrome b | CYTB | -0.37819 | 2.26E-05 | 0.001376 |
| secretion associated Ras related GTPase 1B | SAR1B | 0.303313 | 2.32E-05 | 0.001406 |
| replication protein A3 | RPA3 | 0.348783 | 2.41E-05 | 0.001445 |
| hydroxycarboxylic acid receptor 2 | HCAR2 | 0.399103 | 2.50E-05 | 0.001496 |
| RARA antisense RNA 1 | RARA-AS1 | 0.271713 | 2.55E-05 | 0.001518 |
| cytochrome c oxidase subunit 7B | COX7B | 0.493823 | 2.58E-05 | 0.001529 |
| progestin and adipoQ receptor family member 7 | PAQR7 | -0.33117 | 2.60E-05 | 0.001538 |
| proteasome 20S subunit alpha 3 | PSMA3 | 0.282329 | 2.64E-05 | 0.001551 |
| kazrin, periplakin interacting protein | KAZN | 0.661472 | 2.74E-05 | 0.001607 |
| ribosomal protein S20 | RPS20 | 0.358956 | 2.86E-05 | 0.001652 |
| collagen type XVII alpha 1 chain | COL17A1 | 1.580307 | 2.86E-05 | 0.001652 |
| C-type lectin domain family 2 member B | CLEC2B | 0.409943 | 2.93E-05 | 0.001669 |
| ubiquinol-cytochrome c reductase binding protein | UQCRB | 0.777472 | 3.09E-05 | 0.001742 |
| carbonic anhydrase 4 | CA4 | 0.386842 | 3.10E-05 | 0.001742 |
| ribosomal protein L27 | RPL27 | 0.415013 | 3.10E-05 | 0.001742 |
| adhesion G protein-coupled receptor G3 | ADGRG3 | 0.306472 | 3.11E-05 | 0.001744 |
| ribosomal protein L24 | RPL24 | 0.357174 | 3.13E-05 | 0.001744 |
| ATP synthase peripheral stalk subunit F6 | ATP5PF | 0.338503 | 3.20E-05 | 0.001773 |
| glycophorin B (MNS blood group) | GYPB | 1.251774 | 3.40E-05 | 0.001861 |
| caspase 1 | CASP1 | 0.273816 | 3.43E-05 | 0.001865 |
| olfactomedin 4 | OLFM4 | 1.745119 | 3.45E-05 | 0.001867 |
| TNF alpha induced protein 6 | TNFAIP6 | 0.740659 | 3.50E-05 | 0.001873 |
| bolA family member 2 | BOLA2 | 0.285788 | 3.51E-05 | 0.001873 |
| signal sequence receptor subunit 3 | SSR3 | 0.266492 | 3.63E-05 | 0.001914 |
| complement C1q B chain | C1QB | 0.945877 | 3.76E-05 | 0.001935 |
| NADH:ubiquinone oxidoreductase subunit B6 | NDUFB6 | 0.268541 | 3.95E-05 | 0.002026 |
| CD52 molecule | CD52 | 0.33578 | 4.00E-05 | 0.00204 |
| histidine triad nucleotide binding protein 1 | HINT1 | 0.321698 | 4.04E-05 | 0.002053 |
| potassium inwardly rectifying channel subfamily J member 2 | KCNJ2 | 0.446762 | 4.40E-05 | 0.002172 |
| coagulation factor V | F5 | 0.434673 | 4.48E-05 | 0.002203 |
| hyccin PI4KA lipid kinase complex subunit 2 | HYCC2 | 0.368579 | 4.53E-05 | 0.002207 |
| intraflagellar transport associated protein | IFTAP | 0.911719 | 4.99E-05 | 0.002331 |
| RPS3A pseudogene 5 | RPS3AP5 | 0.980888 | 5.00E-05 | 0.002331 |
| killer cell immunoglobulin like receptor, two Ig domains and long cytoplasmic tail 3 | KIR2DL3 | -0.6859 | 5.02E-05 | 0.002331 |
| ribosomal protein S17 | RPS17 | 0.464329 | 5.04E-05 | 0.002331 |
| VPS9 domain containing 1 | VPS9D1 | 0.281706 | 5.05E-05 | 0.002331 |
| CD177 molecule | CD177 | 1.414877 | 5.09E-05 | 0.002331 |
| CD274 molecule | CD274 | 0.703762 | 5.09E-05 | 0.002331 |
| signal recognition particle 14 | SRP14 | 0.296988 | 5.16E-05 | 0.002344 |
| Fc gamma receptor IIa | FCGR2A | 0.263292 | 5.18E-05 | 0.002344 |
| ubiquitin conjugating enzyme E2 J1 | UBE2J1 | 0.340352 | 5.32E-05 | 0.0024 |
| ZNFX1 antisense RNA 1 | ZFAS1 | 0.358614 | 5.35E-05 | 0.002408 |
| ribosomal protein L26 | RPL26 | 0.670156 | 5.64E-05 | 0.002514 |
| ribosomal protein L36a | RPL36A | 0.705871 | 5.85E-05 | 0.002582 |
| ERGIC and golgi 2 | ERGIC2 | 0.381287 | 5.98E-05 | 0.002618 |
| diazepam binding inhibitor, acyl-CoA binding protein | DBI | 0.377497 | 6.21E-05 | 0.002675 |
| ribosomal protein S3A | RPS3A | 0.488093 | 6.46E-05 | 0.002764 |
| BMP2 inducible kinase | BMP2K | 0.269504 | 6.63E-05 | 0.002798 |
| suppressor of cytokine signaling 3 | SOCS3 | 0.505776 | 6.63E-05 | 0.002798 |
| cytochrome c oxidase assembly factor COX14 | COX14 | 0.268584 | 6.94E-05 | 0.002919 |
| proteasome 20S subunit alpha 6 | PSMA6 | 0.263636 | 7.01E-05 | 0.002942 |
| NADH dehydrogenase subunit 4 | ND4 | -0.35342 | 7.11E-05 | 0.002952 |
| ATP synthase membrane subunit e | ATP5ME | 0.426931 | 7.13E-05 | 0.002952 |
| ATP synthase membrane subunit j | ATP5MJ | 0.346318 | 7.25E-05 | 0.002963 |
| KIT proto-oncogene, receptor tyrosine kinase | KIT | -0.47133 | 7.26E-05 | 0.002963 |
| frizzled class receptor 2 | FZD2 | -0.34725 | 7.33E-05 | 0.002963 |
| ribosomal protein L21 | RPL21 | 0.528271 | 7.34E-05 | 0.002963 |
| BAR/IMD domain containing adaptor protein 2 | BAIAP2 | -0.36968 | 7.35E-05 | 0.002963 |
| ribosomal protein L26 like 1 | RPL26L1 | 0.435322 | 7.35E-05 | 0.002963 |
| killer cell immunoglobulin like receptor, three Ig domains and long cytoplasmic tail 1 | KIR3DL1 | -0.84949 | 7.38E-05 | 0.002963 |
| ribosomal protein S27a | RPS27A | 0.4005 | 7.53E-05 | 0.003002 |
| tumor protein, translationally-controlled 1 | TPT1 | 0.360388 | 7.58E-05 | 0.003002 |
| general transcription factor IIH subunit 5 | GTF2H5 | 0.45248 | 7.60E-05 | 0.003002 |
| tigger transposable element derived 3 | TIGD3 | -0.31904 | 7.63E-05 | 0.003002 |
| G protein subunit gamma 10 | GNG10 | 0.406422 | 7.73E-05 | 0.003031 |
| adrenomedullin | ADM | 0.533217 | 8.30E-05 | 0.003182 |
| LIM domain binding 2 | LDB2 | -0.99571 | 8.89E-05 | 0.00333 |
| long intergenic non-protein coding RNA 570 | LINC00570 | 1.046736 | 8.97E-05 | 0.003338 |
| ATP synthase peripheral stalk subunit OSCP | ATP5PO | 0.319347 | 8.98E-05 | 0.003338 |
| RNA, 5.8S ribosomal N2 | RNA5-8SN2 | 1.22974 | 9.51E-05 | 0.00351 |
| prostaglandin D2 synthase | PTGDS | -0.64281 | 9.53E-05 | 0.00351 |
| NADH:ubiquinone oxidoreductase subunit A1 | NDUFA1 | 0.42587 | 9.64E-05 | 0.003533 |
| transmembrane protein 121B | TMEM121B | 0.4871 | 9.79E-05 | 0.003555 |
| RPS3A pseudogene 6 | RPS3AP6 | 0.786677 | 1.00E-04 | 0.003612 |
| ribosomal protein S7 | RPS7 | 0.456778 | 0.000104 | 0.003686 |
| protein kinase C and casein kinase substrate in neurons 1 | PACSIN1 | -0.43608 | 0.000109 | 0.003829 |
| ribosomal protein L17 | RPL17 | 0.427287 | 0.000111 | 0.003865 |
| tissue factor pathway inhibitor | TFPI | 1.124199 | 0.000111 | 0.003865 |
| ribosomal protein S27 like | RPS27L | 0.429172 | 0.000112 | 0.003868 |
| ectodysplasin A | EDA | 0.644303 | 0.000113 | 0.003889 |
| cytochrome c oxidase subunit I | COX1 | -0.31119 | 0.000115 | 0.003953 |
| coiled-coil domain containing 167 | CCDC167 | 0.357093 | 0.000118 | 0.004024 |
| PET100 cytochrome c oxidase chaperone | PET100 | 0.386381 | 0.000118 | 0.004024 |
| ribosomal protein L36a pseudogene 37 | RPL36AP37 | 0.697658 | 0.000119 | 0.004047 |
| oncostatin M | OSM | 0.400193 | 0.00012 | 0.004047 |
| ribosomal protein L23 | RPL23 | 0.487898 | 0.000124 | 0.004163 |
| mitochondrial ribosomal protein L22 | MRPL22 | 0.381837 | 0.000124 | 0.004163 |
| zinc finger protein 566 | ZNF566 | -0.38027 | 0.000125 | 0.004179 |
| arginase 1 | ARG1 | 0.780004 | 0.000127 | 0.00422 |
| COMM domain containing 6 | COMMD6 | 0.506241 | 0.000127 | 0.004223 |
| protein tyrosine phosphatase receptor type S | PTPRS | -0.48846 | 0.000128 | 0.00423 |
| microtubule associated protein 7 | MAP7 | -0.49289 | 0.000129 | 0.004245 |
| translocase of inner mitochondrial membrane 8 homolog B | TIMM8B | 0.403836 | 0.000132 | 0.004335 |
| translocase of outer mitochondrial membrane 7 | TOMM7 | 0.488637 | 0.000134 | 0.004368 |
| ATP synthase F1 subunit epsilon | ATP5F1E | 0.372577 | 0.000135 | 0.004386 |
| lactotransferrin | LTF | 1.280091 | 0.000139 | 0.004517 |
| selenoprotein K | SELENOK | 0.390569 | 0.00014 | 0.004529 |
| long intergenic non-protein coding RNA 2217 | LINC02217 | 1.410413 | 0.000141 | 0.004529 |
| small EDRK-rich factor 2 | SERF2 | 0.274433 | 0.000143 | 0.004568 |
| transmembrane protein 256 | TMEM256 | 0.329976 | 0.000145 | 0.004604 |
| interferon induced transmembrane protein 1 | IFITM1 | 0.344531 | 0.000151 | 0.00477 |
| cytochrome c oxidase assembly factor COX16 | COX16 | 0.483648 | 0.000154 | 0.004845 |
| free fatty acid receptor 3 | FFAR3 | 0.885665 | 0.000159 | 0.004952 |
| eukaryotic translation elongation factor 1 beta 2 | EEF1B2 | 0.411046 | 0.00016 | 0.004969 |
| translocase of outer mitochondrial membrane 5 | TOMM5 | 0.363119 | 0.000161 | 0.005001 |
| cystatin F | CST7 | 0.401413 | 0.000163 | 0.005025 |
| ankyrin repeat and BTB domain containing 3 | ABTB3 | -0.28226 | 0.000166 | 0.005093 |
| FAM161 centrosomal protein A | FAM161A | 0.607356 | 0.000174 | 0.00527 |
| ribosomal protein L21 pseudogene 119 | RPL21P119 | 0.724199 | 0.000176 | 0.005332 |
| tetratricopeptide repeat domain 9 | TTC9 | -0.32126 | 0.000182 | 0.0055 |
| ribosomal protein L7 | RPL7 | 0.39277 | 0.000189 | 0.005612 |
| ribosomal protein L31 | RPL31 | 0.588251 | 0.000198 | 0.005799 |
| ribosomal protein L34 | RPL34 | 0.644377 | 0.000198 | 0.005799 |
| DNA topoisomerase II alpha | TOP2A | 0.724254 | 0.000199 | 0.005832 |
| cAMP responsive element binding protein 5 | CREB5 | 0.306809 | 0.000201 | 0.005867 |
| ribosomal protein L35a | RPL35A | 0.341911 | 0.000201 | 0.005867 |
| ribosomal protein S23 | RPS23 | 0.383744 | 0.000212 | 0.00615 |
| ecotropic viral integration site 2A | EVI2A | 0.268253 | 0.000222 | 0.006369 |
| trophinin associated protein | TROAP | 1.014735 | 0.000227 | 0.006478 |
| solute carrier family 2 member 3 | SLC2A3 | 0.266983 | 0.000228 | 0.006496 |
| WD repeat and FYVE domain containing 3 | WDFY3 | 0.279969 | 0.000229 | 0.006499 |
| reticulon 1 | RTN1 | -0.34888 | 0.000234 | 0.006577 |
| C-C motif chemokine receptor 3 | CCR3 | -0.56782 | 0.00024 | 0.006743 |
| C-type lectin domain family 4 member A | CLEC4A | 0.281612 | 0.000247 | 0.006819 |
| cyclin dependent kinase inhibitor 3 | CDKN3 | 0.845782 | 0.000255 | 0.007013 |
| purinergic receptor P2Y2 | P2RY2 | -0.41134 | 0.000261 | 0.007143 |
| small nuclear ribonucleoprotein D1 polypeptide | SNRPD1 | 0.510581 | 0.000277 | 0.007538 |
| NADH:ubiquinone oxidoreductase subunit S5 | NDUFS5 | 0.408766 | 0.00028 | 0.007579 |
| GSK3B interacting protein | GSKIP | 0.33111 | 0.000291 | 0.007803 |
| ribosomal protein L11 | RPL11 | 0.387284 | 0.000292 | 0.007803 |
| calcium voltage-gated channel auxiliary subunit beta 4 | CACNB4 | -0.70696 | 0.000293 | 0.007832 |
| ribosomal L24 domain containing 1 | RSL24D1 | 0.270039 | 0.000308 | 0.008122 |
| ectonucleotide pyrophosphatase/phosphodiesterase 3 | ENPP3 | -0.8214 | 0.00031 | 0.008138 |
| CRACD like | CRACDL | -0.53152 | 0.000311 | 0.008138 |
| phosphodiesterase 6G | PDE6G | -0.42488 | 0.000311 | 0.008138 |
| N-acetyltransferase 8 like | NAT8L | -0.7807 | 0.000315 | 0.008182 |
| translation machinery associated 7 homolog | TMA7 | 0.406541 | 0.000317 | 0.008188 |
| INSC spindle orientation adaptor protein | INSC | 1.144247 | 0.000318 | 0.008204 |
| MIR3945 host gene | MIR3945HG | 0.5947 | 0.000321 | 0.008224 |
| germinal center associated signaling and motility like | GCSAML | -0.79079 | 0.000321 | 0.008224 |
| StAR related lipid transfer domain containing 9 | STARD9 | -0.31772 | 0.000336 | 0.008541 |
| sulfotransferase family 1A member 4 | SULT1A4 | -1.40971 | 0.000339 | 0.008541 |
| heparanase | HPSE | 0.321668 | 0.000343 | 0.008595 |
| phosphodiesterase 9A | PDE9A | -0.50241 | 0.000343 | 0.008595 |
| potassium channel tetramerization domain containing 1 | KCTD1 | -0.51379 | 0.000347 | 0.008629 |
| Rho GTPase activating protein 11A | ARHGAP11A | 0.599663 | 0.000363 | 0.008968 |
| NADH dehydrogenase subunit 5 | ND5 | -0.33617 | 0.000367 | 0.009022 |
| calcium voltage-gated channel auxiliary subunit gamma 6 | CACNG6 | -0.7997 | 0.000369 | 0.009057 |
| CD3 delta subunit of T-cell receptor complex | CD3D | 0.27172 | 0.00037 | 0.009068 |
| NADH:ubiquinone oxidoreductase subunit S4 | NDUFS4 | 0.403119 | 0.000371 | 0.009074 |
| aldehyde dehydrogenase 1 family member A1 | ALDH1A1 | -0.3425 | 0.000378 | 0.009192 |
| TNF superfamily member 10 | TNFSF10 | 0.372817 | 0.000383 | 0.00927 |
| alpha hemoglobin stabilizing protein | AHSP | 0.920367 | 0.000399 | 0.009567 |
| phospholipid scramblase 1 | PLSCR1 | 0.435472 | 0.0004 | 0.009567 |
| CKLF like MARVEL transmembrane domain containing 2 | CMTM2 | 0.330951 | 0.000412 | 0.009824 |
| dedicator of cytokinesis 4 | DOCK4 | 0.519058 | 0.000417 | 0.009918 |
| killer cell lectin like receptor B1 | KLRB1 | 0.398478 | 0.000424 | 0.010062 |
| major facilitator superfamily domain containing 2A | MFSD2A | -0.31305 | 0.000439 | 0.010312 |
| neuronal cell adhesion molecule | NRCAM | -0.86001 | 0.00044 | 0.010312 |
| cytochrome c oxidase subunit 6C | COX6C | 0.419603 | 0.000461 | 0.01064 |
| chromosome 17 open reading frame 99 | C17orf99 | 1.146604 | 0.00047 | 0.010762 |
| chromosome 2 open reading frame 81 | C2orf81 | -0.34617 | 0.000473 | 0.010805 |
| RWD domain containing 1 | RWDD1 | 0.263061 | 0.000486 | 0.011039 |
| CEA cell adhesion molecule 8 | CEACAM8 | 1.262225 | 0.000488 | 0.011039 |
| SRA stem-loop interacting RNA binding protein | SLIRP | 0.443404 | 0.000497 | 0.011158 |
| allograft inflammatory factor 1 | AIF1 | 0.286903 | 0.000501 | 0.011191 |
| chemerin chemokine-like receptor 1 | CMKLR1 | -0.3753 | 0.000506 | 0.011277 |
| prefoldin subunit 5 | PFDN5 | 0.440094 | 0.00052 | 0.011578 |
| CEA cell adhesion molecule 6 | CEACAM6 | 1.294553 | 0.000522 | 0.011586 |
| cytoplasmic polyadenylation element binding protein 4 | CPEB4 | 0.2851 | 0.000542 | 0.011872 |
| programmed cell death 1 ligand 2 | PDCD1LG2 | 0.908502 | 0.000542 | 0.011875 |
| tRNA-yW synthesizing protein 1 homolog B | TYW1B | -0.54316 | 0.000546 | 0.011895 |
| membrane spanning 4-domains A4A | MS4A4A | 0.476271 | 0.000563 | 0.012195 |
| FAM111 trypsin like peptidase B | FAM111B | 0.559781 | 0.000571 | 0.012313 |
| defective in cullin neddylation 1 domain containing 1 | DCUN1D1 | 0.354262 | 0.000572 | 0.012316 |
| thymidylate synthetase | TYMS | 0.655642 | 0.000573 | 0.012329 |
| carbonic anhydrase 1 | CA1 | 0.971864 | 0.000582 | 0.012446 |
| ribosomal protein S24 | RPS24 | 0.519201 | 0.000607 | 0.012795 |
| uncharacterized LOC101928143 | LOC101928143 | 0.277477 | 0.000616 | 0.012931 |
| TNF receptor superfamily member 21 | TNFRSF21 | -0.52066 | 0.000641 | 0.013272 |
| FGFR1 oncogene partner 2 | FGFR1OP2 | 0.272978 | 0.000651 | 0.013457 |
| matrix remodeling associated 8 | MXRA8 | -0.79975 | 0.000661 | 0.013621 |
| adhesion G protein-coupled receptor A3 | ADGRA3 | -0.60308 | 0.000667 | 0.013666 |
| carbonic anhydrase 8 | CA8 | -1.03472 | 0.000692 | 0.013964 |
| CFAP58 divergent transcript | CFAP58-DT | 0.515621 | 0.000705 | 0.014199 |
| UDP-GlcNAc:betaGal beta-1,3-N-acetylglucosaminyltransferase 5 | B3GNT5 | 0.333052 | 0.000708 | 0.014228 |
| NSE2 (MMS21) homolog, SMC5-SMC6 complex SUMO ligase | NSMCE2 | 0.267137 | 0.000715 | 0.014333 |
| solute carrier family 37 member 3 | SLC37A3 | 0.279447 | 0.000718 | 0.014353 |
| centromere protein N | CENPN | 0.408495 | 0.000725 | 0.014386 |
| sialic acid binding Ig like lectin 17, pseudogene | SIGLEC17P | -0.43878 | 0.000734 | 0.014507 |
| peptidoglycan recognition protein 1 | PGLYRP1 | 0.43724 | 0.000737 | 0.014542 |
| trophoblast glycoprotein | TPBG | 0.810528 | 0.000743 | 0.014638 |
| orosomucoid 1 | ORM1 | 0.768294 | 0.00076 | 0.014845 |
| A-kinase anchoring protein 12 | AKAP12 | -0.96322 | 0.000765 | 0.014931 |
| ribosomal protein L22 like 1 | RPL22L1 | 0.416391 | 0.00077 | 0.014987 |
| DEP domain containing 1B | DEPDC1B | 0.861165 | 0.000786 | 0.015173 |
| thioredoxin domain containing 17 | TXNDC17 | 0.282063 | 0.000787 | 0.015173 |
| GATA binding protein 2 | GATA2 | -0.8428 | 0.000806 | 0.015389 |
| membrane metalloendopeptidase | MME | 0.282499 | 0.000806 | 0.015389 |
| polypeptide N-acetylgalactosaminyltransferase 14 | GALNT14 | 0.644123 | 0.000809 | 0.015403 |
| BMX non-receptor tyrosine kinase | BMX | 0.399749 | 0.000814 | 0.015466 |
| ENY2 transcription and export complex 2 subunit | ENY2 | 0.302303 | 0.000821 | 0.015576 |
| retinoic acid induced 14 | RAI14 | 1.296819 | 0.000862 | 0.016175 |
| lipocalin like 1 | LCNL1 | -0.77395 | 0.000905 | 0.016743 |
| ribosomal protein S6 | RPS6 | 0.273932 | 0.000917 | 0.01689 |
| granzyme K | GZMK | 0.34151 | 0.000929 | 0.016989 |
| NADH:ubiquinone oxidoreductase subunit B1 | NDUFB1 | 0.32351 | 0.000936 | 0.017095 |
| peripheral myelin protein 22 | PMP22 | -0.67808 | 0.000944 | 0.017188 |
| uncharacterized LOC105376805 | LOC105376805 | 0.429481 | 0.000947 | 0.017201 |
| free fatty acid receptor 2 | FFAR2 | 0.346565 | 0.000951 | 0.017254 |
| nudix hydrolase 2 | NUDT2 | 0.322068 | 0.000958 | 0.017319 |
| major facilitator superfamily domain containing 4B | MFSD4B | -0.34558 | 0.000972 | 0.017489 |
| tryptase alpha/beta 1 | TPSAB1 | -1.39131 | 0.000991 | 0.017778 |
| ATP binding cassette subfamily A member 13 | ABCA13 | 1.038826 | 0.000997 | 0.017862 |
| scinderin | SCIN | 1.30513 | 0.001002 | 0.017877 |
| discs large MAGUK scaffold protein 2 | DLG2 | -0.91394 | 0.001012 | 0.017912 |
| G protein-coupled receptor 84 | GPR84 | 0.476407 | 0.001012 | 0.017912 |
| ribosomal protein S12 | RPS12 | 0.280613 | 0.001014 | 0.017912 |
| scavenger receptor class F member 1 | SCARF1 | 0.389537 | 0.001014 | 0.017912 |
| Rho GTPase activating protein 32 | ARHGAP32 | -0.33336 | 0.001041 | 0.018258 |
| histidine decarboxylase | HDC | -0.8876 | 0.001076 | 0.018657 |
| tripartite motif containing 9 | TRIM9 | 0.747982 | 0.001107 | 0.019055 |
| caveolin 2 | CAV2 | -0.92436 | 0.001117 | 0.019118 |
| kinesin family member 18B | KIF18B | 0.851269 | 0.00112 | 0.019118 |
| TNF receptor superfamily member 17 | TNFRSF17 | 0.73186 | 0.001125 | 0.019185 |
| phospholipase D family member 4 | PLD4 | -0.33616 | 0.001135 | 0.019271 |
| protein inhibitor of activated STAT 2 | PIAS2 | -0.26412 | 0.001139 | 0.019321 |
| G protein-coupled receptor 171 | GPR171 | 0.332546 | 0.00114 | 0.019321 |
| hemoglobin subunit delta | HBD | 0.79173 | 0.001142 | 0.019321 |
| programmed cell death 10 | PDCD10 | 0.263982 | 0.001144 | 0.019342 |
| CD69 molecule | CD69 | 0.272351 | 0.001162 | 0.019598 |
| ATP synthase F0 subunit 6 | ATP6 | -0.29228 | 0.001176 | 0.019697 |
| RAD9 checkpoint clamp component B | RAD9B | 0.607333 | 0.001215 | 0.0202 |
| pleckstrin homology like domain family A member 3 | PHLDA3 | 0.913889 | 0.00125 | 0.020697 |
| pro-platelet basic protein | PPBP | 0.625029 | 0.001271 | 0.020991 |
| ubiquitin protein ligase E3D | UBE3D | -0.39027 | 0.001295 | 0.021289 |
| reticulon 2 | RTN2 | 0.330225 | 0.001312 | 0.021498 |
| sestrin 3 | SESN3 | 0.520529 | 0.001329 | 0.021686 |
| cytochrome c oxidase subunit II | COX2 | -0.26976 | 0.001359 | 0.022075 |
| integrin subunit beta 8 | ITGB8 | -0.84288 | 0.001367 | 0.022075 |
| collagen type IX alpha 2 chain | COL9A2 | -0.3328 | 0.0014 | 0.022494 |
| LSM5 homolog, U6 small nuclear RNA and mRNA degradation associated | LSM5 | 0.287323 | 0.001453 | 0.023098 |
| ribosomal protein L7a pseudogene 16 | RPL7AP16 | -0.64658 | 0.001462 | 0.023106 |
| glutathione peroxidase 3 | GPX3 | -0.4182 | 0.001464 | 0.023119 |
| tripartite motif containing 74 | TRIM74 | -0.75188 | 0.001522 | 0.023843 |
| H2B clustered histone 18 | H2BC18 | 0.450531 | 0.001537 | 0.023987 |
| selenium binding protein 1 | SELENBP1 | 0.739211 | 0.00155 | 0.024077 |
| cyclin and CBS domain divalent metal cation transport mediator 2 | CNNM2 | -0.26322 | 0.001555 | 0.024102 |
| corin, serine peptidase | CORIN | 1.280504 | 0.001563 | 0.024199 |
| NME/NM23 nucleoside diphosphate kinase 1 | NME1 | 0.280124 | 0.001566 | 0.024211 |
| olfactory receptor family 6 subfamily N member 1 | OR6N1 | -1.69783 | 0.001583 | 0.024391 |
| SAP domain containing ribonucleoprotein | SARNP | 0.320534 | 0.001583 | 0.024391 |
| radial spoke head component 9 | RSPH9 | 0.674861 | 0.001584 | 0.024391 |
| PRKAR2A antisense RNA 1 | PRKAR2A-AS1 | -0.37734 | 0.001604 | 0.024571 |
| keratin 5 | KRT5 | -0.73644 | 0.001617 | 0.02468 |
| TIFA inhibitor | TIFAB | -0.41039 | 0.001674 | 0.02536 |
| EF-hand calcium binding domain 2 | EFCAB2 | 0.476151 | 0.001698 | 0.025477 |
| DNA polymerase epsilon 4, accessory subunit | POLE4 | 0.293003 | 0.001701 | 0.025492 |
| ZNF232 antisense RNA 1 | ZNF232-AS1 | 0.441852 | 0.001743 | 0.025823 |
| MIR663A host gene | MIR663AHG | 0.76215 | 0.001746 | 0.025832 |
| NLR family apoptosis inhibitory protein | NAIP | 0.323451 | 0.001779 | 0.02611 |
| zinc finger protein 254 | ZNF254 | 0.336523 | 0.001814 | 0.026395 |
| immunoglobulin heavy variable 4-39 | IGHV4-39 | 0.871691 | 0.001824 | 0.026495 |
| erb-b2 receptor tyrosine kinase 2 | ERBB2 | -0.35841 | 0.001851 | 0.026712 |
| NDUFA4 mitochondrial complex associated | NDUFA4 | 0.358768 | 0.001862 | 0.02681 |
| oxysterol binding protein like 5 | OSBPL5 | -0.2706 | 0.001895 | 0.02718 |
| ankyrin repeat and sterile alpha motif domain containing 1B | ANKS1B | 1.240321 | 0.001899 | 0.027217 |
| ribosomal protein L22 | RPL22 | 0.300594 | 0.001916 | 0.027352 |
| ribosomal protein L41 pseudogene 2 | RPL41P2 | 0.579093 | 0.001933 | 0.027458 |
| cyclin A1 | CCNA1 | -0.97482 | 0.001935 | 0.027458 |
| MCTS1 re-initiation and release factor | MCTS1 | 0.317674 | 0.001936 | 0.027458 |
| leukocyte immunoglobulin like receptor A6 | LILRA6 | 0.455495 | 0.001938 | 0.027458 |
| protein phosphatase, Mg2+/Mn2+ dependent 1J | PPM1J | -0.45139 | 0.001943 | 0.0275 |
| myosin light chain kinase family member 4 | MYLK4 | -0.43967 | 0.001977 | 0.027878 |
| ribosomal protein S7 pseudogene 1 | RPS7P1 | 0.637428 | 0.001982 | 0.027924 |
| ring finger protein 227 | RNF227 | -0.33053 | 0.002005 | 0.028117 |
| long intergenic non-protein coding RNA 852 | LINC00852 | -0.31946 | 0.002046 | 0.028372 |
| leucine rich alpha-2-glycoprotein 1 | LRG1 | 0.276106 | 0.002058 | 0.028491 |
| potassium two pore domain channel subfamily K member 17 | KCNK17 | -1.03718 | 0.002079 | 0.028708 |
| thioredoxin reductase 3 | TXNRD3 | -0.49847 | 0.002098 | 0.02889 |
| prostaglandin D2 receptor 2 | PTGDR2 | -0.49298 | 0.002112 | 0.029046 |
| solute carrier family 45 member 3 | SLC45A3 | -0.57375 | 0.002114 | 0.029046 |
| membrane spanning 4-domains A2 | MS4A2 | -0.82889 | 0.002119 | 0.029092 |
| uncharacterized LOC100130357 | LOC100130357 | -0.43804 | 0.002177 | 0.029727 |
| mitochondrial ribosomal protein S18C | MRPS18C | 0.349318 | 0.00219 | 0.029874 |
| YOD1 deubiquitinase | YOD1 | 0.279443 | 0.002202 | 0.030011 |
| 5-oxoprolinase, ATP-hydrolysing | OPLAH | 0.398702 | 0.002228 | 0.030333 |
| secretory leukocyte peptidase inhibitor | SLPI | 0.549163 | 0.002231 | 0.030333 |
| RAD51 recombinase | RAD51 | 0.619497 | 0.002311 | 0.031164 |
| ribosomal protein L21 pseudogene 98 | RPL21P98 | 0.595674 | 0.002339 | 0.031485 |
| HtrA serine peptidase 3 | HTRA3 | 1.125628 | 0.002353 | 0.031609 |
| RALY antisense RNA 1 | RALY-AS1 | 0.438925 | 0.002418 | 0.032312 |
| long intergenic non-protein coding RNA 664 | LINC00664 | 1.02902 | 0.002422 | 0.032313 |
| long intergenic non-protein coding RNA 2289 | LINC02289 | -0.43782 | 0.00244 | 0.032463 |
| long intergenic non-protein coding RNA 892 | LINC00892 | 0.537171 | 0.002487 | 0.033009 |
| regulator of G protein signaling 10 | RGS10 | 0.273609 | 0.002495 | 0.033057 |
| KCNJ2 antisense RNA 1 | KCNJ2-AS1 | 0.40465 | 0.002498 | 0.033064 |
| G protein-coupled receptor 162 | GPR162 | -0.4977 | 0.002506 | 0.033113 |
| fibulin 2 | FBLN2 | -0.51639 | 0.002507 | 0.033113 |
| syndecan 3 | SDC3 | -0.43188 | 0.002508 | 0.033113 |
| ADAM metallopeptidase with thrombospondin type 1 motif 10 | ADAMTS10 | -0.31947 | 0.002651 | 0.034454 |
| MIRLET7B host gene | MIRLET7BHG | -0.26881 | 0.002654 | 0.034457 |
| SH2 domain containing adaptor protein B | SHB | -0.46901 | 0.002714 | 0.035033 |
| ribosomal protein L21 pseudogene 53 | RPL21P53 | 0.667225 | 0.002728 | 0.035083 |
| secretory carrier membrane protein 5 | SCAMP5 | -0.46157 | 0.002738 | 0.035163 |
| microRNA 3648-1 | MIR3648-1 | 0.80679 | 0.002746 | 0.035175 |
| epoxide hydrolase 1 | EPHX1 | -0.26901 | 0.002775 | 0.035457 |
| neurotrophic receptor tyrosine kinase 1 | NTRK1 | -0.68742 | 0.002819 | 0.035725 |
| carboxypeptidase A3 | CPA3 | -0.7233 | 0.002841 | 0.035964 |
| CD248 molecule | CD248 | -0.4181 | 0.002852 | 0.036006 |
| solute carrier family 2 member 5 | SLC2A5 | 0.652074 | 0.002855 | 0.036006 |
| BASP1 antisense RNA 1 | BASP1-AS1 | 0.601569 | 0.002856 | 0.036006 |
| cell division cycle 20 | CDC20 | 0.754303 | 0.002857 | 0.036006 |
| arachidonate 15-lipoxygenase type B | ALOX15B | -0.59234 | 0.002863 | 0.036027 |
| granzyme B | GZMB | -0.35124 | 0.002873 | 0.036127 |
| uncharacterized LOC107985211 | LOC107985211 | -0.55942 | 0.002895 | 0.036239 |
| WNK lysine deficient protein kinase 2 | WNK2 | -0.70648 | 0.002908 | 0.036345 |
| integrin subunit beta 4 | ITGB4 | 0.72641 | 0.003052 | 0.037775 |
| serpin family B member 10 | SERPINB10 | 0.942389 | 0.003061 | 0.037841 |
| Wnt ligand secretion mediator | WLS | 0.410819 | 0.003075 | 0.037963 |
| mucolipin TRP cation channel 3 | MCOLN3 | 0.650049 | 0.003096 | 0.03816 |
| dishevelled binding antagonist of beta catenin 1 | DACT1 | 0.614153 | 0.003213 | 0.039219 |
| KIAA0408 | KIAA0408 | 0.793018 | 0.003244 | 0.039526 |
| family with sequence similarity 13 member A | FAM13A | 0.312088 | 0.003261 | 0.039638 |
| tetratricopeptide repeat domain 26 | TTC26 | 0.543097 | 0.003267 | 0.039681 |
| DNAJC25-GNG10 readthrough | DNAJC25-GNG10 | 0.373864 | 0.003332 | 0.040219 |
| polycystin 1, transient receptor potential channel interacting pseudogene 5 | PKD1P5 | 1.142358 | 0.003335 | 0.040219 |
| phosphodiesterase 2A | PDE2A | 0.522869 | 0.003345 | 0.040251 |
| sterile alpha motif domain containing 9 | SAMD9 | 0.28537 | 0.003346 | 0.040251 |
| glutathione peroxidase 1 | GPX1 | 0.290058 | 0.003357 | 0.040317 |
| adenylate kinase 6 | AK6 | 0.420034 | 0.003361 | 0.040332 |
| bactericidal permeability increasing protein | BPI | 0.783945 | 0.00337 | 0.040379 |
| rogdi atypical leucine zipper | ROGDI | 0.263762 | 0.003401 | 0.040622 |
| KIAA0825 | KIAA0825 | 0.291831 | 0.003412 | 0.040718 |
| non-SMC condensin II complex subunit G2 | NCAPG2 | 0.688345 | 0.003431 | 0.040816 |
| potassium voltage-gated channel subfamily H member 3 | KCNH3 | 0.339039 | 0.003445 | 0.040924 |
| periaxin | PRX | -0.35062 | 0.003498 | 0.04149 |
| armadillo repeat containing X-linked 1 | ARMCX1 | -0.33419 | 0.003518 | 0.04162 |
| interleukin 1 beta | IL1B | 0.273076 | 0.003634 | 0.04256 |
| cyclin B2 | CCNB2 | 0.683737 | 0.003716 | 0.043287 |
| DNA damage induced apoptosis suppressor | DDIAS | 0.282691 | 0.003736 | 0.043485 |
| dolichyl-phosphate mannosyltransferase subunit 2, regulatory | DPM2 | 0.263399 | 0.003826 | 0.044328 |
| uncharacterized LOC100129203 | LOC100129203 | 0.326081 | 0.003836 | 0.044412 |
| cyclin E2 | CCNE2 | 0.698812 | 0.003862 | 0.044541 |
| galectin 12 | LGALS12 | -0.34972 | 0.003906 | 0.044945 |
| LSM3 homolog, U6 small nuclear RNA and mRNA degradation associated | LSM3 | 0.308288 | 0.003912 | 0.04498 |
| growth arrest specific 5 | GAS5 | 0.297842 | 0.003926 | 0.045035 |
| tubulin beta 2B class IIb | TUBB2B | 1.438451 | 0.003979 | 0.045575 |
| killer cell immunoglobulin like receptor, three Ig domains and long cytoplasmic tail 2 | KIR3DL2 | -0.5894 | 0.003988 | 0.045577 |
| 5'-aminolevulinate synthase 2 | ALAS2 | 0.72352 | 0.004007 | 0.04569 |
| FA complementation group L | FANCL | 0.326062 | 0.004059 | 0.046125 |
| long intergenic non-protein coding RNA 1857 | LINC01857 | 0.536683 | 0.004061 | 0.046125 |
| ribosomal protein L39 like | RPL39L | 0.399249 | 0.004073 | 0.046148 |
| collagen type XIII alpha 1 chain | COL13A1 | -0.77837 | 0.004097 | 0.04626 |
| roundabout guidance receptor 1 | ROBO1 | -1.00589 | 0.004123 | 0.046415 |
| perforin 1 | PRF1 | -0.30858 | 0.004193 | 0.047029 |
| SRY-box transcription factor 13 | SOX13 | -0.30113 | 0.004233 | 0.047302 |
| potassium voltage-gated channel subfamily A member 5 | KCNA5 | -0.58417 | 0.004245 | 0.047327 |
| phosphoglucomutase 5 | PGM5 | -0.90595 | 0.004286 | 0.047648 |
| ER membrane protein complex subunit 3 | EMC3 | 0.265596 | 0.004295 | 0.047676 |
| NADH dehydrogenase subunit 1 | ND1 | -0.26876 | 0.004316 | 0.04776 |
| RPAP3 divergent transcript | RPAP3-DT | 0.399716 | 0.004404 | 0.048627 |
| cell division cycle associated 5 | CDCA5 | 0.519091 | 0.004613 | 0.050383 |
| myomesin 2 | MYOM2 | -1.15123 | 0.004621 | 0.050395 |
| lysozyme g1 | LYG1 | -0.31905 | 0.004697 | 0.051049 |
| long intergenic non-protein coding RNA 2596 | LINC02596 | 0.584937 | 0.004718 | 0.051136 |
| leucine zipper transcription factor like 1 | LZTFL1 | 0.322282 | 0.004769 | 0.051338 |
| POM121 membrane glycoprotein (rat) pseudogene | LOC728488 | 0.589796 | 0.004772 | 0.051338 |
| complement C1q A chain | C1QA | 0.543659 | 0.004809 | 0.051624 |
| small nucleolar RNA host gene 8 | SNHG8 | 0.360156 | 0.004842 | 0.051875 |
| Yip1 domain family member 6 | YIPF6 | 0.267563 | 0.004954 | 0.05255 |
| SEM1 26S proteasome subunit | SEM1 | 0.388725 | 0.00496 | 0.052579 |
| poly(ADP-ribose) polymerase family member 9 | PARP9 | 0.295918 | 0.005055 | 0.053248 |
| chloride intracellular channel 3 | CLIC3 | -0.34557 | 0.005092 | 0.053512 |
| C-type lectin domain family 4 member D | CLEC4D | 0.427187 | 0.005096 | 0.053512 |
| ras related dexamethasone induced 1 | RASD1 | -0.45061 | 0.005134 | 0.053662 |
| trophinin | TRO | -0.67057 | 0.005141 | 0.053664 |
| TLC domain containing 4 | TLCD4 | 0.905436 | 0.005172 | 0.053876 |
| TNF superfamily member 13b | TNFSF13B | 0.360072 | 0.005231 | 0.054334 |
| ectonucleotide pyrophosphatase/phosphodiesterase 2 | ENPP2 | -0.45837 | 0.005423 | 0.055905 |
| serpin family E member 2 | SERPINE2 | -0.52571 | 0.005441 | 0.055936 |
| centrin 3 | CETN3 | 0.472633 | 0.005453 | 0.05599 |
| septin 3 | SEPTIN3 | -0.48691 | 0.005465 | 0.056067 |
| stereocilin pseudogene 1 | STRCP1 | -0.71939 | 0.005468 | 0.056069 |
| CDC28 protein kinase regulatory subunit 1B | CKS1B | 0.263638 | 0.005475 | 0.056075 |
| T cell receptor alpha variable 21 | TRAV21 | -0.39802 | 0.005476 | 0.056075 |
| eukaryotic translation initiation factor 3 subunit E pseudogene 3 | EIF3EP3 | -5.28311 | 0.005509 | 0.05631 |
| ankyrin repeat domain 22 | ANKRD22 | 0.662071 | 0.005514 | 0.05631 |
| galectin 3 | LGALS3 | 0.291409 | 0.005526 | 0.056355 |
| NIMA related kinase 11 | NEK11 | -0.49196 | 0.005601 | 0.056604 |
| long intergenic non-protein coding RNA 1093 | LINC01093 | 0.833692 | 0.005625 | 0.056744 |
| defensin alpha 1B | DEFA1B | 1.078246 | 0.005687 | 0.05718 |
| uncharacterized LOC102724701 | LOC102724701 | 1.15131 | 0.005729 | 0.05749 |
| cache domain containing 1 | CACHD1 | -0.53675 | 0.005773 | 0.057854 |
| early growth response 1 | EGR1 | -0.36472 | 0.005788 | 0.057968 |
| ribosomal protein L9 pseudogene 8 | RPL9P8 | 0.77618 | 0.005824 | 0.05817 |
| uncharacterized LOC124908011 | LOC124908011 | -0.61608 | 0.005857 | 0.058449 |
| zinc finger protein 578 | ZNF578 | -0.3439 | 0.006069 | 0.0599 |
| long intergenic non-protein coding RNA 1176 | LINC01176 | -0.45367 | 0.006108 | 0.060132 |
| C1q and TNF related 4 | C1QTNF4 | -0.52586 | 0.006136 | 0.060331 |
| lysosomal associated membrane protein family member 5 | LAMP5 | -0.41533 | 0.006154 | 0.060463 |
| uncharacterized LOC105372421 | LOC105372421 | -0.69057 | 0.006227 | 0.060983 |
| CSF2RB pseudogene 1 | CSF2RBP1 | 0.554292 | 0.006355 | 0.061834 |
| dishevelled associated activator of morphogenesis 2 | DAAM2 | -0.77401 | 0.00647 | 0.062466 |
| long intergenic non-protein coding RNA 1750 | LINC01750 | -0.80758 | 0.006497 | 0.062613 |
| DNAJC3 divergent transcript | DNAJC3-DT | 0.276243 | 0.006527 | 0.062862 |
| killer cell lectin like receptor C2 | KLRC2 | -0.8119 | 0.006549 | 0.062985 |
| ribosomal protein S2 pseudogene 32 | RPS2P32 | 0.589009 | 0.006557 | 0.062992 |
| polypeptide N-acetylgalactosaminyltransferase 4 | GALNT4 | 0.556165 | 0.00659 | 0.063141 |
| SAM and SH3 domain containing 1 | SASH1 | -0.46425 | 0.006686 | 0.06355 |
| anaphase promoting complex subunit 10 | ANAPC10 | 0.289157 | 0.006759 | 0.063944 |
| signal peptide, CUB domain and EGF like domain containing 1 | SCUBE1 | -1.06935 | 0.006794 | 0.064161 |
| LINE1 type transposase domain containing 1 | L1TD1 | 0.907835 | 0.006834 | 0.064393 |
| RNA binding fox-1 homolog 3 | RBFOX3 | -1.06538 | 0.006843 | 0.064393 |
| RPS27A pseudogene 16 | RPS27AP16 | 0.714084 | 0.006849 | 0.064393 |
| FAM20A golgi associated secretory pathway pseudokinase | FAM20A | 0.725976 | 0.006865 | 0.064484 |
| angiomotin | AMOT | -0.29275 | 0.006872 | 0.064484 |
| scavenger receptor class A member 5 | SCARA5 | -0.76876 | 0.006876 | 0.064484 |
| CEA cell adhesion molecule 19 | CEACAM19 | -0.33065 | 0.006919 | 0.06461 |
| plasminogen activator, urokinase | PLAU | 0.416259 | 0.006925 | 0.06461 |
| kirre like nephrin family adhesion molecule 3 | KIRREL3 | -0.83577 | 0.007008 | 0.065202 |
| glutamate receptor interacting protein 1 | GRIP1 | -0.99222 | 0.007019 | 0.06522 |
| cyclin dependent kinase 18 | CDK18 | -0.31515 | 0.007033 | 0.065264 |
| FHF complex subunit HOOK interacting protein 1A | FHIP1A | -0.54877 | 0.007055 | 0.065391 |
| cyclin dependent kinase like 1 | CDKL1 | 0.509206 | 0.007103 | 0.065798 |
| immunoglobulin lambda variable 4-69 | IGLV4-69 | 0.737847 | 0.007108 | 0.065804 |
| G protein regulated inducer of neurite outgrowth 1 | GPRIN1 | 0.345208 | 0.00712 | 0.065874 |
| cyclin dependent kinase inhibitor 2B | CDKN2B | 0.382894 | 0.007132 | 0.065946 |
| mitochondrial ribosomal protein S33 | MRPS33 | 0.290083 | 0.007228 | 0.066623 |
| immunoglobulin lambda variable 3-19 | IGLV3-19 | 0.688921 | 0.007305 | 0.067295 |
| TBC1 domain family member 3D | TBC1D3D | -3.57368 | 0.007349 | 0.06762 |
| mitochondrial ribosomal protein L13 | MRPL13 | 0.309202 | 0.007399 | 0.06787 |
| Fc epsilon receptor Ia | FCER1A | -0.43966 | 0.007399 | 0.06787 |
| acyl-CoA synthetase long chain family member 6 | ACSL6 | 0.346483 | 0.007434 | 0.068056 |
| resistin | RETN | 0.572995 | 0.007466 | 0.068278 |
| zinc finger and BTB domain containing 12 | ZBTB12 | -0.32193 | 0.007492 | 0.068428 |
| plexin B3 | PLXNB3 | 0.820774 | 0.007536 | 0.068751 |
| interferon induced protein with tetratricopeptide repeats 5 | IFIT5 | 0.360972 | 0.007542 | 0.068764 |
| nucleolar protein 3 | NOL3 | 0.270671 | 0.007615 | 0.069077 |
| RNA polymerase II subunit J4, pseudogene | POLR2J4 | -0.3242 | 0.007621 | 0.069077 |
| chromosome 16 open reading frame 96 | C16orf96 | -0.55426 | 0.007673 | 0.069248 |
| transmembrane protein 241 | TMEM241 | -0.26962 | 0.007689 | 0.069288 |
| ubiquitin conjugating enzyme E2 C | UBE2C | 0.417126 | 0.007692 | 0.069288 |
| acyl-CoA thioesterase 13 | ACOT13 | 0.267152 | 0.007707 | 0.069356 |
| leucine rich repeat containing 26 | LRRC26 | -0.51615 | 0.007771 | 0.069795 |
| T cell receptor beta variable 11-2 | TRBV11-2 | -0.4147 | 0.007779 | 0.069826 |
| endoplasmic reticulum protein 27 | ERP27 | -0.4099 | 0.00781 | 0.069976 |
| outer dense fiber of sperm tails 3B | ODF3B | 0.324264 | 0.007857 | 0.070194 |
| MT-ATP6 pseudogene 1 | MTATP6P1 | -0.35056 | 0.00793 | 0.070763 |
| neuronal growth regulator 1 | NEGR1 | -0.77423 | 0.008016 | 0.071185 |
| zinc finger DHHC-type palmitoyltransferase 2 | ZDHHC2 | 0.306653 | 0.008058 | 0.071395 |
| defensin alpha 3 | DEFA3 | 1.266069 | 0.008183 | 0.072081 |
| RAB13, member RAS oncogene family | RAB13 | 0.366064 | 0.008353 | 0.073087 |
| long intergenic non-protein coding RNA 2649 | LINC02649 | 0.278203 | 0.008361 | 0.073087 |
| armadillo repeat containing X-linked 4 | ARMCX4 | -0.33658 | 0.008431 | 0.073528 |
| DOCK8 antisense RNA 1 | DOCK8-AS1 | 0.292917 | 0.008504 | 0.073953 |
| transmembrane serine protease 9 | TMPRSS9 | 0.718207 | 0.008672 | 0.074938 |
| DENND3 antisense RNA 1 | DENND3-AS1 | 0.322709 | 0.008679 | 0.074938 |
| ribonuclease A family member 3 | RNASE3 | 0.795471 | 0.008741 | 0.075265 |
| YES proto-oncogene 1, Src family tyrosine kinase | YES1 | -0.2644 | 0.008745 | 0.075265 |
| tripartite motif-containing 51B, pseudogene | TRIM51BP | -0.90436 | 0.008765 | 0.075312 |
| NADH:ubiquinone oxidoreductase complex assembly factor 4 | NDUFAF4 | 0.297934 | 0.008914 | 0.075944 |
| delta like canonical Notch ligand 1 | DLL1 | -0.34105 | 0.009006 | 0.076424 |
| pantothenate kinase 1 | PANK1 | -0.42882 | 0.009072 | 0.076805 |
| synthesis of cytochrome C oxidase 2 | SCO2 | 0.27955 | 0.009106 | 0.076978 |
| long intergenic non-protein coding RNA 2904 | LINC02904 | -0.77515 | 0.009107 | 0.076978 |
| ubiquitin conjugating enzyme E2 T | UBE2T | 0.386838 | 0.009347 | 0.078431 |
| sprouty RTK signaling antagonist 2 | SPRY2 | -0.3608 | 0.009514 | 0.079392 |
| RAB7B, member RAS oncogene family | RAB7B | -0.54747 | 0.009514 | 0.079392 |
| RNA binding motif single stranded interacting protein 3 | RBMS3 | -0.96648 | 0.009619 | 0.079797 |
| X-linked Kx blood group antigen, Kell and VPS13A binding protein | XK | 0.471109 | 0.0097 | 0.080012 |
| C1q and TNF related 3 | C1QTNF3 | -0.28255 | 0.009792 | 0.080416 |
| solute carrier family 4 member 1 (Diego blood group) | SLC4A1 | 0.555574 | 0.009831 | 0.080517 |
| mannosidase endo-alpha like | MANEAL | -0.32395 | 0.009893 | 0.080985 |
| chitinase 3 like 1 | CHI3L1 | -0.50228 | 0.010024 | 0.081873 |
| photoreceptor disc component | PRCD | -0.71944 | 0.010186 | 0.082886 |
| suppressor APC domain containing 1 | SAPCD1 | -0.52497 | 0.010193 | 0.082899 |
| uncharacterized LOC124900513 | LOC124900513 | 0.277007 | 0.01023 | 0.083152 |
| DLG associated protein 5 | DLGAP5 | 0.650729 | 0.010248 | 0.083211 |
| RNA, U6 small nuclear 5, pseudogene | RNU6-5P | 0.768699 | 0.010255 | 0.083226 |
| ATP synthase F0 subunit 8 | ATP8 | -0.30953 | 0.010371 | 0.083983 |
| elongation factor for RNA polymerase II 2 | ELL2 | 0.316635 | 0.010404 | 0.084159 |
| cell division cycle 45 | CDC45 | 0.613379 | 0.01046 | 0.084469 |
| ubiquitin specific peptidase 45 | USP45 | 0.311289 | 0.010482 | 0.084558 |
| ribonucleotide reductase regulatory subunit M2 | RRM2 | 0.576937 | 0.010544 | 0.084884 |
| KRT73 antisense RNA 1 | KRT73-AS1 | -0.56036 | 0.010583 | 0.085103 |
| leucine zipper tumor suppressor 1 | LZTS1 | 0.490692 | 0.010602 | 0.085122 |
| uncharacterized LOC105379362 | LOC105379362 | 0.547145 | 0.010662 | 0.085556 |
| prolyl 3-hydroxylase 2 | P3H2 | -0.68 | 0.010768 | 0.086138 |
| guanylate kinase 1 | GUK1 | 0.30973 | 0.010868 | 0.086653 |
| F-box protein 36 | FBXO36 | 0.428106 | 0.010914 | 0.086901 |
| lymphatic vessel endothelial hyaluronan receptor 1 | LYVE1 | 0.521912 | 0.010915 | 0.086901 |
| tyrosylprotein sulfotransferase 1 | TPST1 | -0.40627 | 0.010916 | 0.086901 |
| keratin 8 pseudogene 46 | KRT8P46 | 0.371423 | 0.010945 | 0.086997 |
| long intergenic non-protein coding RNA 2458 | LINC02458 | -0.63309 | 0.011273 | 0.089034 |
| defensin alpha 4 | DEFA4 | 1.043913 | 0.011337 | 0.089312 |
| proline rich and Gla domain 4 | PRRG4 | 0.283556 | 0.011567 | 0.090555 |
| coiled-coil domain containing 30 | CCDC30 | -0.38322 | 0.011594 | 0.090673 |
| chromosome 2 open reading frame 76 | C2orf76 | 0.268988 | 0.011643 | 0.09086 |
| T cell receptor beta variable 12-5 | TRBV12-5 | -0.51792 | 0.011705 | 0.091015 |
| EDRF1 divergent transcript | EDRF1-DT | 0.655717 | 0.011736 | 0.091168 |
| FKBP prolyl isomerase 10 | FKBP10 | -0.68675 | 0.011736 | 0.091168 |
| killer cell immunoglobulin like receptor, two Ig domains and long cytoplasmic tail 1 | KIR2DL1 | -0.53269 | 0.011751 | 0.091235 |
| erythrocyte membrane protein band 4.2 | EPB42 | 0.512617 | 0.01191 | 0.091901 |
| EPH receptor A2 | EPHA2 | -0.4924 | 0.011995 | 0.092316 |
| G protein subunit gamma 11 | GNG11 | 0.414206 | 0.012042 | 0.092535 |
| shroom family member 4 | SHROOM4 | 0.542646 | 0.012062 | 0.092598 |
| uncharacterized LOC101929107 | LOC101929107 | 0.590164 | 0.012128 | 0.092913 |
| semaphorin 7A (John Milton Hagen blood group) | SEMA7A | -0.27951 | 0.012157 | 0.092992 |
| tetraspanin 7 | TSPAN7 | 0.566568 | 0.012291 | 0.093637 |
| granulysin | GNLY | -0.35909 | 0.012494 | 0.094946 |
| calpain 5 | CAPN5 | -0.26541 | 0.012509 | 0.094964 |
| actin filament associated protein 1 | AFAP1 | -0.37834 | 0.012541 | 0.095109 |
| netrin G2 | NTNG2 | 0.298687 | 0.012551 | 0.095115 |
| FERM domain containing 3 | FRMD3 | 0.417771 | 0.012588 | 0.095229 |
| MEF2 activating motif and SAP domain containing transcriptional regulator | MAMSTR | -0.48604 | 0.012752 | 0.096277 |
| lipase H | LIPH | 0.835677 | 0.012768 | 0.096346 |
| ALMS1 pseudogene 1 | ALMS1P1 | -0.59024 | 0.012902 | 0.097065 |
| B and T lymphocyte associated | BTLA | 0.272659 | 0.012954 | 0.097363 |
| zinc finger DHHC-type palmitoyltransferase 19 | ZDHHC19 | 0.621462 | 0.012967 | 0.097363 |
| glutamine amidotransferase class 1 domain containing 3 | GATD3 | -0.75915 | 0.013042 | 0.097687 |
| tryptase beta 2 | TPSB2 | -1.30978 | 0.013043 | 0.097687 |
| protocadherin gamma subfamily C, 3 | PCDHGC3 | -0.65005 | 0.013132 | 0.098046 |
| ZEB1 antisense RNA 1 | ZEB1-AS1 | 0.323348 | 0.013184 | 0.098252 |
| glutaredoxin 5 | GLRX5 | 0.367666 | 0.013186 | 0.098252 |
| tropomodulin 1 | TMOD1 | 0.427974 | 0.01319 | 0.098252 |
| interferon alpha inducible protein 27 | IFI27 | 0.883525 | 0.013242 | 0.098496 |
| RAB3A interacting protein like 1 | RAB3IL1 | 0.502752 | 0.013256 | 0.098513 |
| tripartite motif containing 58 | TRIM58 | 0.475641 | 0.013358 | 0.098917 |
| ***MDD CRP 1-3 mg/L vs. MDD CRP <1 mg/L*** | | | | |
| immunoglobulin kappa variable 2D-28 | IGKV2D-28 | -36.8436 | 8.84E-18 | 1.44E-13 |
| tetratricopeptide repeat domain 28 | TTC28 | -0.84681 | 7.54E-07 | 0.005749 |
| ribosomal protein L23a pseudogene 88 | RPL23AP88 | -20.9367 | 1.06E-06 | 0.005749 |
| YWHAH antisense RNA 1 | YWHAH-AS1 | -1.44221 | 1.55E-06 | 0.006316 |
| C-C motif chemokine ligand 3 like 1 | CCL3L1 | -2.28994 | 3.03E-06 | 0.009893 |
| lamin B1 | LMNB1 | 0.315805 | 4.29E-06 | 0.011671 |
| TNF superfamily member 10 | TNFSF10 | 0.496487 | 6.80E-06 | 0.015865 |
| leucine rich repeat containing 26 | LRRC26 | -0.82646 | 1.72E-05 | 0.032731 |
| zinc finger protein 438 | ZNF438 | 0.264493 | 3.20E-05 | 0.040286 |
| heterogeneous nuclear ribonucleoprotein L like | HNRNPLL | 0.278142 | 3.21E-05 | 0.040286 |
| interferon induced transmembrane protein 1 | IFITM1 | 0.400548 | 3.59E-05 | 0.041924 |
| nei like DNA glycosylase 3 | NEIL3 | 1.440228 | 4.56E-05 | 0.042616 |
| Ras association (RalGDS/AF-6) and pleckstrin homology domains 1 | RAPH1 | -0.62323 | 4.70E-05 | 0.042616 |
| beta-2-microglobulin | B2M | 0.32001 | 5.44E-05 | 0.042739 |
| spermidine/spermine N1-acetyltransferase 1 | SAT1 | 0.298233 | 5.50E-05 | 0.042739 |
| methylenetetrahydrofolate dehydrogenase (NADP+ dependent) 2, methenyltetrahydrofolate cyclohydrolase | MTHFD2 | 0.294373 | 6.19E-05 | 0.04591 |
| annexin A3 | ANXA3 | 0.53289 | 6.53E-05 | 0.046375 |
| pleckstrin homology like domain family A member 3 | PHLDA3 | 1.121329 | 6.97E-05 | 0.047444 |
| vesicle associated membrane protein 5 | VAMP5 | 0.317222 | 8.51E-05 | 0.053459 |
| BCL6 transcription repressor | BCL6 | 0.296427 | 9.73E-05 | 0.058856 |
| phospholipid scramblase 1 | PLSCR1 | 0.515767 | 0.000112 | 0.06314 |
| pleckstrin homology and RhoGEF domain containing G4 | PLEKHG4 | -0.33064 | 0.000126 | 0.068747 |
| RNA binding motif single stranded interacting protein 3 | RBMS3 | -1.45156 | 0.000148 | 0.078108 |
| Fas cell surface death receptor | FAS | 0.32004 | 0.000178 | 0.086195 |
| translocase of inner mitochondrial membrane 10 | TIMM10 | 0.439702 | 0.000198 | 0.086195 |
| transmembrane protein 126B | TMEM126B | 0.317219 | 0.0002 | 0.086195 |
| glutamate receptor interacting protein 1 | GRIP1 | -1.37041 | 0.000209 | 0.086195 |
| Fc gamma receptor Ia | FCGR1A | 0.672608 | 0.00022 | 0.086195 |
| synthesis of cytochrome C oxidase 2 | SCO2 | 0.404454 | 0.000224 | 0.086195 |
| cut like homeobox 2 | CUX2 | -0.56615 | 0.000227 | 0.086195 |
| acyl-CoA synthetase long chain family member 1 | ACSL1 | 0.273612 | 0.000232 | 0.086195 |
| ubiquitin associated protein 1 like | UBAP1L | -0.27828 | 0.000234 | 0.086195 |
| ribonucleoprotein, PTB binding 2 | RAVER2 | 0.664285 | 0.000238 | 0.086195 |
| CD3 delta subunit of T-cell receptor complex | CD3D | 0.28535 | 0.000258 | 0.089531 |
| TNF alpha induced protein 6 | TNFAIP6 | 0.690778 | 0.000268 | 0.08991 |
| netrin G2 | NTNG2 | 0.43655 | 0.00027 | 0.08991 |
| legumain | LGMN | -0.34259 | 0.000288 | 0.091656 |
| ubiquitin conjugating enzyme E2 C | UBE2C | 0.496614 | 0.000292 | 0.091656 |
| Fc gamma receptor Ic, pseudogene | FCGR1CP | 0.782575 | 0.000314 | 0.095072 |
| olfactory receptor family 7 subfamily D member 2 | OR7D2 | -3.42201 | 0.000323 | 0.095751 |
| sterile alpha motif domain containing 9 | SAMD9 | 0.378257 | 0.000332 | 0.096479 |
| radial spoke head component 9 | RSPH9 | 0.809828 | 0.000349 | 0.096479 |
| BCL2 related protein A1 | BCL2A1 | 0.467382 | 0.000355 | 0.096562 |
| FHF complex subunit HOOK interacting protein 1A | FHIP1A | -0.73832 | 0.000375 | 0.097272 |
| kringle containing transmembrane protein 1 | KREMEN1 | 0.504856 | 0.000396 | 0.098283 |
| keratin 5 | KRT5 | -0.81727 | 0.000412 | 0.098283 |
| ***MDD CRP >3 mg/L vs. MDD CRP 1-3 mg/L*** | | | | |
| 5'-aminolevulinate synthase 2 | ALAS2 | 1.367898 | 3.57E-06 | 0.024086 |
| S100 calcium binding protein A12 | S100A12 | 0.63038 | 4.29E-06 | 0.024086 |
| carbonic anhydrase 1 | CA1 | 1.426148 | 1.39E-05 | 0.037825 |
| RAP1 GTPase activating protein | RAP1GAP | 2.070322 | 1.47E-05 | 0.037825 |
| A-kinase anchoring protein 12 | AKAP12 | -1.16587 | 5.17E-05 | 0.074627 |
| purinergic receptor P2Y2 | P2RY2 | -0.49454 | 5.81E-05 | 0.074627 |
| myosin light chain 6B | MYL6B | 0.46416 | 6.67E-05 | 0.077864 |
| interleukin 3 receptor subunit alpha | IL3RA | -0.7503 | 7.57E-05 | 0.08105 |
| sestrin 3 | SESN3 | 0.727313 | 9.01E-05 | 0.082699 |
| S100 calcium binding protein A9 | S100A9 | 0.288051 | 0.000107 | 0.088305 |
| germinal center associated signaling and motility like | GCSAML | -0.8924 | 0.000142 | 0.088305 |
| selenium binding protein 1 | SELENBP1 | 1.033283 | 0.000142 | 0.088305 |
| nectin cell adhesion molecule 2 | NECTIN2 | -1.12553 | 0.000155 | 0.088305 |
| defective in cullin neddylation 1 domain containing 1 | DCUN1D1 | 0.474341 | 0.000157 | 0.088305 |
| SLIT-ROBO Rho GTPase activating protein 2 | SRGAP2 | -0.30751 | 0.000164 | 0.088305 |
| zinc finger protein 496 | ZNF496 | -0.32067 | 0.00017 | 0.088305 |
| PTEN induced kinase 1 | PINK1 | 0.297635 | 0.000176 | 0.088305 |
| coiled-coil domain containing 71 like | CCDC71L | 0.320038 | 0.000207 | 0.094961 |
| hemoglobin subunit delta | HBD | 1.045691 | 0.000236 | 0.098303 |
| membrane bound O-acyltransferase domain containing 2 | MBOAT2 | 0.293039 | 0.000247 | 0.098303 |
| TBC1 domain family member 3 | TBC1D3 | 5.736239 | 0.000248 | 0.098303 |
| acyl-CoA synthetase long chain family member 6 | ACSL6 | 0.513662 | 0.000253 | 0.098303 |
| tropomyosin 1 | TPM1 | 0.328917 | 0.000262 | 0.098319 |
| ***MDD CRP <1 vs. controls selected for serum CRP <1 mg/L*** | | | | |
| WD repeat domain 13 | WDR13 | -0.26822 | 1.75E-06 | 0.003172 |
| ribosomal protein S6 kinase A5 | RPS6KA5 | 0.329565 | 2.73E-06 | 0.003725 |
| PAXX non-homologous end joining factor | PAXX | -0.28155 | 3.43E-06 | 0.004011 |
| bromodomain adjacent to zinc finger domain 2B | BAZ2B | 0.396945 | 4.07E-06 | 0.004165 |
| supervillin | SVIL | 0.311247 | 1.36E-05 | 0.008596 |
| lysine demethylase 5A | KDM5A | 0.312292 | 1.36E-05 | 0.008596 |
| proline rich 5 | PRR5 | -0.31504 | 5.13E-05 | 0.022086 |
| PDZK1 interacting protein 1 | PDZK1IP1 | -0.81616 | 5.50E-05 | 0.022494 |
| CBFA2/RUNX1 partner transcriptional co-repressor 3 | CBFA2T3 | 0.273768 | 6.38E-05 | 0.023507 |
| transmembrane protein 86B | TMEM86B | -0.37618 | 7.27E-05 | 0.023507 |
| KLF transcription factor 7 | KLF7 | 0.283647 | 7.55E-05 | 0.023507 |
| caseinolytic mitochondrial matrix peptidase chaperone subunit X | CLPX | 0.288508 | 7.75E-05 | 0.023507 |
| Cbl proto-oncogene | CBL | 0.265929 | 9.62E-05 | 0.024822 |
| mitotic spindle organizing protein 2A | MZT2A | -0.35444 | 0.000102 | 0.024822 |
| glutathione peroxidase 1 | GPX1 | -0.47792 | 0.000104 | 0.024822 |
| tetraspanin 32 | TSPAN32 | -0.31122 | 0.000153 | 0.030053 |
| tRNA methyltransferase 1 like | TRMT1L | 0.316742 | 0.000171 | 0.030053 |
| granzyme K | GZMK | -0.46904 | 0.000172 | 0.030053 |
| centrosomal protein 19 | CEP19 | 0.32461 | 0.000233 | 0.033119 |
| bone morphogenetic protein receptor type 2 | BMPR2 | 0.304651 | 0.000268 | 0.035417 |
| DNA polymerase lambda | POLL | -0.37562 | 0.00028 | 0.035811 |
| dolichyl-phosphate mannosyltransferase subunit 2, regulatory | DPM2 | -0.35306 | 0.000304 | 0.03742 |
| solute carrier family 45 member 4 | SLC45A4 | 0.278597 | 0.000306 | 0.03742 |
| pecanex 2 | PCNX2 | -0.31813 | 0.000335 | 0.038619 |
| chromodomain helicase DNA binding protein 7 | CHD7 | 0.42103 | 0.000578 | 0.04734 |
| myosin light chain 9 | MYL9 | -0.84198 | 0.000585 | 0.04734 |
| granzyme M | GZMM | -0.34187 | 0.000587 | 0.04734 |
| mitogen-activated protein kinase 8 | MAPK8 | 0.312714 | 0.000607 | 0.047767 |
| solute carrier family 1 member 5 | SLC1A5 | -0.34112 | 0.000634 | 0.047767 |
| colony stimulating factor 1 | CSF1 | 0.364104 | 0.000636 | 0.047767 |
| erythrocyte membrane protein band 4.2 | EPB42 | -0.74322 | 0.000672 | 0.04805 |
| tubulin beta 2A class IIa | TUBB2A | -1.26708 | 0.000738 | 0.04951 |
| chromosome 12 open reading frame 75 | C12orf75 | -0.4409 | 0.000741 | 0.04951 |
| SMG1 pseudogene 3 | SMG1P3 | 0.457422 | 0.000828 | 0.052544 |
| T cell receptor gamma constant 2 | TRGC2 | -0.5155 | 0.001047 | 0.059875 |
| Rap guanine nucleotide exchange factor 2 | RAPGEF2 | 0.323923 | 0.001053 | 0.059875 |
| LYL1 basic helix-loop-helix family member | LYL1 | -0.36927 | 0.00106 | 0.059875 |
| calmodulin regulated spectrin associated protein 1 | CAMSAP1 | 0.274033 | 0.001103 | 0.06183 |
| galectin 3 | LGALS3 | -0.44577 | 0.001129 | 0.061898 |
| T cell receptor gamma constant 1 | TRGC1 | -0.57824 | 0.001245 | 0.064059 |
| ADP ribosylation factor like GTPase 11 | ARL11 | 0.288277 | 0.00151 | 0.067001 |
| selenium binding protein 1 | SELENBP1 | -0.80863 | 0.001514 | 0.067001 |
| aryl hydrocarbon receptor | AHR | 0.318369 | 0.00163 | 0.068965 |
| olfactory receptor family 2 subfamily W member 3 | OR2W3 | -0.77268 | 0.001651 | 0.068965 |
| hydroxymethylbilane synthase | HMBS | -0.36081 | 0.001737 | 0.070569 |
| BCL2 like 1 | BCL2L1 | -0.59561 | 0.001806 | 0.070818 |
| signal regulatory protein beta 1 | SIRPB1 | 0.420781 | 0.001808 | 0.070818 |
| nuclear factor I X | NFIX | -0.54507 | 0.001928 | 0.072537 |
| autophagy related 2B | ATG2B | 0.267498 | 0.001973 | 0.072537 |
| C-C motif chemokine ligand 5 | CCL5 | -0.4622 | 0.001986 | 0.072537 |
| cystatin F | CST7 | -0.36327 | 0.002079 | 0.073667 |
| plakophilin 4 | PKP4 | 0.302008 | 0.002405 | 0.080051 |
| small EDRK-rich factor 2 | SERF2 | -0.27485 | 0.002424 | 0.080051 |
| NME/NM23 nucleoside diphosphate kinase 4 | NME4 | -0.32566 | 0.002521 | 0.080212 |
| TATA-box binding protein associated factor 4 | TAF4 | 0.275574 | 0.002611 | 0.080212 |
| ornithine decarboxylase antizyme 1 | OAZ1 | -0.28699 | 0.002628 | 0.080212 |
| solute carrier family 25 member 39 | SLC25A39 | -0.58081 | 0.002666 | 0.080539 |
| spermine oxidase | SMOX | -0.47261 | 0.002688 | 0.080895 |
| tripartite motif containing 58 | TRIM58 | -0.69948 | 0.002736 | 0.081467 |
| solute carrier family 8 member A1 | SLC8A1 | 0.315216 | 0.002804 | 0.082093 |
| Snf2 related CREBBP activator protein | SRCAP | 0.419446 | 0.002835 | 0.082093 |
| solute carrier family 4 member 1 (Diego blood group) | SLC4A1 | -0.7582 | 0.003083 | 0.084626 |
| SLIT-ROBO Rho GTPase activating protein 2B | SRGAP2B | 0.363272 | 0.003226 | 0.086425 |
| immunoglobulin heavy constant gamma 4 (G4m marker) | IGHG4 | -0.7948 | 0.003248 | 0.086566 |
| immunoglobulin heavy constant gamma 1 (G1m marker) | IGHG1 | -0.99198 | 0.003427 | 0.088931 |
| cathepsin W | CTSW | -0.40461 | 0.003447 | 0.088931 |
| insulin receptor substrate 2 | IRS2 | 0.266775 | 0.003547 | 0.088949 |
| biliverdin reductase B | BLVRB | -0.35953 | 0.003669 | 0.090379 |
| N-deacetylase and N-sulfotransferase 1 | NDST1 | 0.280317 | 0.004008 | 0.094009 |
| guanylate kinase 1 | GUK1 | -0.38375 | 0.004203 | 0.095496 |
| protein geranylgeranyltransferase type I subunit beta | PGGT1B | 0.320865 | 0.004286 | 0.096605 |
| vasohibin 1 | VASH1 | 0.466459 | 0.004345 | 0.096758 |
| desmocollin 2 | DSC2 | 0.589959 | 0.004631 | 0.099521 |
| Gene transcripts are listed based on their p- and FDR-adjusted p-values (smaller to the top). FDR= false discovery rate (Benjamini-Hochberg); log2FC= logarithm base 2 of the fold change. | | | | |

# ***Supplementary Table S3:*** *mRNA transcripts differentially expressed (FDR-adjusted) in treatment-based MDD cases and controls*

| *Gene name* | *Gene symbol* | *log2FC* | *p-value* | *FDR-adjusted p-value* |
| --- | --- | --- | --- | --- |
| **Treatment-based** | | | | |
| *MDD unmedicated vs. controls* | | | | |
| // | // | // | // | // |
| *MDD responders vs. controls* | | | | |
| protocadherin alpha 5 | PCDHA5 | -29.4967 | 1.85E-12 | 3.01E-08 |
| small nucleolar RNA, H/ACA box 73A | SNORA73A | -29.0687 | 3.75E-12 | 3.06E-08 |
| immunoglobulin heavy variable 3-49 | IGHV3-49 | -1.3462 | 1.23E-06 | 0.006681 |
| bromodomain adjacent to zinc finger domain 2B | BAZ2B | 0.321041 | 6.26E-06 | 0.020456 |
| NME/NM23 nucleoside diphosphate kinase 4 | NME4 | -0.34854 | 3.33E-05 | 0.088217 |
| DDB1 and CUL4 associated factor 4 like 1 | DCAF4L1 | 0.541296 | 3.78E-05 | 0.088217 |
| *MDD non-responders vs. controls* | | | | |
| LMBR1 domain containing 2 | LMBRD2 | -0.47487 | 2.94E-06 | 0.006136 |
| small integral membrane protein 4 | SMIM4 | 0.606839 | 3.30E-06 | 0.006136 |
| MAGUK p55 scaffold protein 7 | MPP7 | -0.26715 | 4.13E-06 | 0.006136 |
| ferredoxin reductase | FDXR | -0.27858 | 1.28E-05 | 0.015242 |
| MT-ND1 pseudogene 23 | MTND1P23 | -1.61699 | 0.000165 | 0.036301 |
| BTB domain containing 19 | BTBD19 | -0.43557 | 0.000215 | 0.04128 |
| lunapark, ER junction formation factor | LNPK | -0.32461 | 0.00029 | 0.044805 |
| brain expressed X-linked 2 | BEX2 | 0.34408 | 0.000311 | 0.045062 |
| BRCA1 DNA repair associated | BRCA1 | -0.32637 | 0.000394 | 0.052631 |
| GEN1 Holliday junction 5' flap endonuclease | GEN1 | -0.46855 | 0.000415 | 0.053071 |
| zinc finger protein 431 | ZNF431 | -0.35489 | 0.000495 | 0.059908 |
| mitochondrial ribosomal protein L51 | MRPL51 | 0.271432 | 0.000545 | 0.0626 |
| small nuclear ribonucleoprotein polypeptide G | SNRPG | 0.287247 | 0.000552 | 0.0626 |
| ABO, alpha 1-3-N-acetylgalactosaminyltransferase and alpha 1-3-galactosyltransferase | ABO | -0.92803 | 0.000617 | 0.064977 |
| golgin A8 family member O | GOLGA8O | -0.45641 | 0.000644 | 0.066105 |
| mitochondrial ribosomal protein S33 | MRPS33 | 0.317232 | 0.000701 | 0.069388 |
| killer cell lectin like receptor C1 | KLRC1 | -0.63051 | 0.000712 | 0.069388 |
| immunoglobulin heavy variable 3-49 | IGHV3-49 | -1.016 | 0.000788 | 0.072446 |
| caspase recruitment domain family member 16 | CARD16 | 0.370314 | 0.000907 | 0.07817 |
| sushi domain containing 4 | SUSD4 | 0.451468 | 0.001018 | 0.084297 |
| Fc epsilon receptor II | FCER2 | 0.41434 | 0.001046 | 0.08526 |
| CD180 molecule | CD180 | 0.278765 | 0.001195 | 0.089481 |
| tubulin folding cofactor E | TBCE | 0.370554 | 0.001207 | 0.089481 |
| DNA polymerase epsilon 4, accessory subunit | POLE4 | 0.286056 | 0.001218 | 0.089481 |
| LDOC1 regulator of NFKB signaling | LDOC1 | 0.40198 | 0.001274 | 0.091824 |
| crystallin beta-gamma domain containing 3 | CRYBG3 | 0.324973 | 0.001347 | 0.091852 |
| ribosomal protein S25 | RPS25 | 0.283913 | 0.001551 | 0.09822 |
| *MDD responders vs. MDD non-responders* | | | | |
| homeodomain interacting protein kinase 3 | HIPK3 | 0.295907 | 2.92E-07 | 0.000259 |
| MAGUK p55 scaffold protein 7 | MPP7 | 0.28978 | 4.47E-07 | 0.00028 |
| abhydrolase domain containing 3, phospholipase | ABHD3 | 0.29223 | 4.76E-07 | 0.000281 |
| speckle type BTB/POZ protein like | SPOPL | 0.277599 | 1.08E-06 | 0.000412 |
| centrosomal protein 97 | CEP97 | 0.325746 | 1.15E-06 | 0.000412 |
| RPTOR independent companion of MTOR complex 2 | RICTOR | 0.264425 | 2.71E-06 | 0.000719 |
| dedicator of cytokinesis 5 | DOCK5 | 0.285861 | 7.92E-06 | 0.00139 |
| mitochondrial trans-2-enoyl-CoA reductase | MECR | -0.35441 | 8.50E-06 | 0.001434 |
| Dmx like 2 | DMXL2 | 0.273426 | 2.52E-05 | 0.002658 |
| REL proto-oncogene, NF-kB subunit | REL | 0.266336 | 4.15E-05 | 0.003342 |
| MIR22 host gene | MIR22HG | 0.287404 | 7.75E-05 | 0.004844 |
| NME/NM23 nucleoside diphosphate kinase 4 | NME4 | -0.30879 | 9.81E-05 | 0.005642 |
| zinc finger FYVE-type containing 16 | ZFYVE16 | 0.314771 | 0.000113 | 0.00607 |
| bromodomain adjacent to zinc finger domain 2B | BAZ2B | 0.272191 | 0.000124 | 0.006296 |
| pseudouridine synthase 7 like | PUS7L | 0.266964 | 0.00013 | 0.006435 |
| cAMP responsive element binding protein 1 | CREB1 | 0.266484 | 0.000226 | 0.008889 |
| GTF2H2 family member C | GTF2H2C | 0.291337 | 0.000241 | 0.009179 |
| prostaglandin-endoperoxide synthase 2 | PTGS2 | 0.306722 | 0.000263 | 0.009679 |
| tripartite motif containing 33 | TRIM33 | 0.303833 | 0.0003 | 0.010288 |
| nuclear factor of activated T cells 5 | NFAT5 | 0.270465 | 0.000306 | 0.010442 |
| afadin, adherens junction formation factor | AFDN | 0.555474 | 0.000378 | 0.011518 |
| hyccin PI4KA lipid kinase complex subunit 2 | HYCC2 | 0.290312 | 0.000382 | 0.011584 |
| von Willebrand factor C and EGF domains | VWCE | -0.68001 | 0.00043 | 0.012343 |
| diphosphoinositol pentakisphosphate kinase 2 | PPIP5K2 | 0.263369 | 0.000468 | 0.013031 |
| solute carrier family 25 member 40 | SLC25A40 | 0.304608 | 0.000529 | 0.014215 |
| dehydrogenase/reductase 9 | DHRS9 | 0.363891 | 0.000631 | 0.015848 |
| small nucleolar RNA host gene 5 | SNHG5 | 0.443919 | 0.001038 | 0.020856 |
| glutathione peroxidase 1 | GPX1 | -0.31682 | 0.001438 | 0.025599 |
| leucine rich repeat containing 27 | LRRC27 | -0.33325 | 0.001474 | 0.026156 |
| hemoglobin subunit gamma 1 | HBG1 | -1.91573 | 0.001533 | 0.026759 |
| LMBR1 domain containing 2 | LMBRD2 | 0.324213 | 0.001631 | 0.027454 |
| folate receptor gamma | FOLR3 | -0.85723 | 0.001731 | 0.0281 |
| GEN1 Holliday junction 5' flap endonuclease | GEN1 | 0.398533 | 0.001808 | 0.028754 |
| proline rich and Gla domain 4 | PRRG4 | 0.304706 | 0.001826 | 0.028867 |
| tetraspanin 33 | TSPAN33 | -0.27949 | 0.001827 | 0.028867 |
| argonaute RISC catalytic component 3 | AGO3 | 0.265027 | 0.001935 | 0.029829 |
| gamma-glutamyltransferase 1 | GGT1 | -0.30488 | 0.001936 | 0.029829 |
| cytochrome P450 family 4 subfamily F member 3 | CYP4F3 | 0.303404 | 0.002646 | 0.036229 |
| cubilin | CUBN | -0.41204 | 0.002851 | 0.03788 |
| ADNP homeobox 2 | ADNP2 | 0.341982 | 0.00299 | 0.03862 |
| V-set pre-B cell surrogate light chain 3 | VPREB3 | -0.37206 | 0.003023 | 0.038728 |
| tetratricopeptide repeat domain 39B | TTC39B | 0.264551 | 0.003348 | 0.041465 |
| solute carrier family 22 member 15 | SLC22A15 | 0.269089 | 0.003433 | 0.04185 |
| immunoglobulin heavy variable 4-59 | IGHV4-59 | -0.51363 | 0.003589 | 0.043064 |
| myelin protein zero like 1 | MPZL1 | 0.279026 | 0.003668 | 0.043427 |
| immunoglobulin lambda variable 3-19 | IGLV3-19 | -0.56453 | 0.003907 | 0.04501 |
| BMI1 proto-oncogene, polycomb ring finger | BMI1 | 0.265529 | 0.004153 | 0.046571 |
| prepronociceptin | PNOC | -0.27885 | 0.004483 | 0.048378 |
| cytoplasmic polyadenylation element binding protein 2 | CPEB2 | 0.298083 | 0.004504 | 0.048467 |
| tetratricopeptide repeat domain 12 | TTC12 | -0.29514 | 0.004763 | 0.050268 |
| discs large MAGUK scaffold protein 5 | DLG5 | 0.408016 | 0.005018 | 0.051594 |
| serine protease 53 | PRSS53 | 0.359891 | 0.005069 | 0.051942 |
| insulin like growth factor binding protein 4 | IGFBP4 | -0.26616 | 0.005361 | 0.053717 |
| immunoglobulin lambda variable 2-23 | IGLV2-23 | -0.55319 | 0.005545 | 0.054912 |
| thromboxane A2 receptor | TBXA2R | -0.29379 | 0.005671 | 0.055668 |
| transmembrane protein 164 | TMEM164 | 0.27772 | 0.005789 | 0.056409 |
| tyrosylprotein sulfotransferase 1 | TPST1 | 0.462213 | 0.006501 | 0.060356 |
| CD79a molecule | CD79A | -0.28166 | 0.006743 | 0.061741 |
| long intergenic non-protein coding RNA 1410 | LINC01410 | 0.264569 | 0.007095 | 0.063544 |
| autophagy related 9B | ATG9B | -0.2736 | 0.007359 | 0.065095 |
| tetraspanin 2 | TSPAN2 | 0.278077 | 0.00753 | 0.066106 |
| immunoglobulin heavy variable 1-18 | IGHV1-18 | -0.51388 | 0.007745 | 0.066995 |
| matrix metallopeptidase 17 | MMP17 | -0.26561 | 0.007759 | 0.067058 |
| guanylate binding protein 1 | GBP1 | 0.32726 | 0.0078 | 0.06725 |
| zinc finger protein 420 | ZNF420 | -0.26727 | 0.00878 | 0.072299 |
| zinc finger protein 117 | ZNF117 | 0.266115 | 0.009154 | 0.074232 |
| major histocompatibility complex, class I, J (pseudogene) | HLA-J | -1.11176 | 0.009442 | 0.075868 |
| C-type lectin domain family 4 member D | CLEC4D | 0.345815 | 0.009807 | 0.077568 |
| guanylate binding protein 5 | GBP5 | 0.316956 | 0.010031 | 0.07876 |
| immunoglobulin lambda variable 3-25 | IGLV3-25 | -0.49858 | 0.010251 | 0.079976 |
| Fc epsilon receptor II | FCER2 | -0.32185 | 0.012352 | 0.089572 |
| SMG1 pseudogene 5 | SMG1P5 | 0.286685 | 0.012653 | 0.091043 |
| laminin subunit alpha 5 | LAMA5 | -0.45172 | 0.012671 | 0.091043 |
| FLVCR heme transporter 1 | FLVCR1 | 0.282909 | 0.012749 | 0.091148 |
| tescalcin | TESC | -0.30943 | 0.013301 | 0.09402 |
| PDZK1 interacting protein 1 | PDZK1IP1 | -0.41517 | 0.013706 | 0.095987 |
| golgin A8 family member O | GOLGA8O | 0.324984 | 0.014272 | 0.098211 |
| immunoglobulin heavy variable 4-34 | IGHV4-34 | -0.40383 | 0.014334 | 0.098442 |
| *MDD unmedicated vs. MDD responders* | | | | |
| small nucleolar RNA, H/ACA box 73A | SNORA73A | 30.28082 | 2.62E-10 | 4.27E-06 |
| protocadherin alpha 5 | PCDHA5 | 28.6278 | 2.32E-09 | 1.89E-05 |
| immunoglobulin kappa variable 2D-28 | IGKV2D-28 | 25.98795 | 5.84E-08 | 0.000318 |
| ribosomal protein L23a pseudogene 88 | RPL23AP88 | -24.2519 | 4.18E-07 | 0.001706 |
| acetyl-CoA acetyltransferase 1 | ACAT1 | 0.355339 | 6.65E-06 | 0.021724 |
| RAP1 GTPase activating protein | RAP1GAP | -2.11874 | 3.67E-05 | 0.07489 |
| *MDD unmedicated vs. MDD non-responders* | | | | |
| beta-1,4-galactosyltransferase 5 | B4GALT5 | 0.295822 | 2.51E-06 | 0.017919 |
| inhibitor of DNA binding 2 | ID2 | 0.266898 | 0.000156 | 0.08002 |
| protein prenyltransferase alpha subunit repeat containing 1 | PTAR1 | 0.264882 | 0.000336 | 0.096913 |
| DnaJ heat shock protein family (Hsp40) member B2 | DNAJB2 | -0.29476 | 0.000358 | 0.096913 |
| armadillo like helical domain containing 1 | ARMH1 | -0.28073 | 0.000396 | 0.096913 |
| folate receptor gamma | FOLR3 | -1.127 | 0.000467 | 0.099252 |
| Gene transcripts are listed based on their p- and FDR-adjusted p-values (smaller to the top). FDR= false discovery rate (Benjamini-Hochberg); log2FC= logarithm base 2 of the fold change. | | | | |

# ***Supplementary Table S4:*** *mRNA transcripts differentially expressed (FDR-adjusted) in all MDD cases vs. controls*

| *Gene name* | *Gene symbol* | *log2FC* | *p-value* | *FDR-adjusted p-value* |
| --- | --- | --- | --- | --- |
| **Whole MDD cases vs. controls** | | | | |
| immunoglobulin heavy variable 3-49 | IGHV3-49 | -1.14602 | 5.32E-07 | 0.008685 |
| immunoglobulin kappa variable 2D-28 | IGKV2D-28 | 16.72879 | 1.35E-06 | 0.011042 |
| MT-ND4 pseudogene 24 | MTND4P24 | -6.0489 | 5.17E-06 | 0.028145 |
| small nuclear ribonucleoprotein polypeptide G | SNRPG | 0.327505 | 1.59E-05 | 0.058263 |
| caspase recruitment domain family member 16 | CARD16 | 0.423799 | 2.09E-05 | 0.058263 |
| small integral membrane protein 4 | SMIM4 | 0.487295 | 2.50E-05 | 0.058263 |
| Putative uncharacterized protein FLJ44553 | LOC100130691 | -0.57049 | 2.97E-05 | 0.060679 |
| RNA binding protein, mRNA processing factor 2 | RBPMS2 | -1.02292 | 4.04E-05 | 0.067619 |
| immunoglobulin heavy constant gamma 3 (G3m marker) | IGHG3 | -0.68205 | 4.14E-05 | 0.067619 |
| Gene transcripts are listed based on their p- and FDR-adjusted p-values (smaller to the top). FDR= false discovery rate (Benjamini-Hochberg); log2FC= logarithm base 2 of the fold change. | | | | |

# ***Supplementary Table S5:*** *Canonical pathways differentially activated in group comparisons (CRP-based MDD groupings)*

| **Ingenuity Canonical Pathways** | **z-score** | **Molecules** |
| --- | --- | --- |
| ***MDD CRP 1-3 mg/L vs. controls*** | | |
| Role of Hypercytokinemia/hyperchemokinemia in the Pathogenesis of Influenza | 4 | CCL2,CXCL10,EIF2AK2,IFIT2,IFIT3,IL1RN,IRF7,ISG15,MX1,OAS1,OAS2,OAS3,RIGI,RSAD2,STAT1,STAT2 |
| Interferon Signaling | 2.714 | IFI35,IFI6,IFIT1,IFIT3,IFITM1,ISG15,MX1,OAS1,SOCS1,STAT1,STAT2 |
| Coronavirus Pathogenesis Pathway | -2.683 | BST2,CCL2,IRF7,MAPK8,OAS1,OAS2,OAS3,RIGI,RN7SL1,RN7SL2,RPS17,RPS24,RPS25,RPS27,RPS27L,RPS29,RPS3A,RPS7,STAT1,STAT2 |
| EIF2 Signaling | 3.317 | EIF2AK2,RPL17,RPL21,RPL23,RPL26,RPL26L1,RPL31,RPL34,RPL36A,RPL39,RPL41,RPS17,RPS24,RPS25,RPS27,RPS27L,RPS29,RPS3A,RPS7 |
| Role of Pattern Recognition Receptors in Recognition of Bacteria and Viruses | 2.449 | C1QC,EDA,EIF2AK2,IFIH1,IRF7,MAPK8,OAS1,OAS2,OAS3,RIGI,TNFSF10,TNFSF15,TNFSF9 |
| Multiple Sclerosis Signaling Pathway | 2.84 | C1QC,C2,C8G,EDA,GRIN3A,HLA-G,PARP12,PARP14,PARP9,RNF213,SLC4A1,STAT1,TNFSF10,TNFSF15,TNFSF9 |
| Pathogen Induced Cytokine Storm Signaling Pathway | 3.578 | AIM2,CCL2,CCL23,CCL3L1,COL17A1,COL4A3,CXCL10,DHX58,EDA,IFIH1,IL1RL1,IL1RN,IRF7,MAPK8,RIGI,STAT1,TNFSF10,TNFSF15,TNFSF9,ZBP1 |
| Systemic Lupus Erythematosus In B Cell Signaling Pathway | 2.496 | BCL2L1,EDA,IFIH1,IFIT2,IFIT3,IGHG1,IGHG3,IGHV1-46,IGHV3-13,IGHV3-49,IGHV3-53,IGHV6-1,IGKV6-21,IGLV3-9,IGLV5-45,IRF7,ISG15,STAT1,STAT2,TNFSF10,TNFSF15,TNFSF9 |
| Necroptosis Signaling Pathway | 2.333 | EIF2AK2,PLA2G2D,STAT1,STAT2,TIMM10,TIMM8B,TNFSF10,TOMM7,ZBP1 |
| Role Of Osteoblasts In Rheumatoid Arthritis Signaling Pathway | 2.53 | ACVRL1,EDA,MMP19,MMP8,SMAD1,STAT1,STAT2,TNFSF10,TNFSF15,TNFSF9,WNT11 |
| Death Receptor Signaling | 2.449 | MAPK8,PARP12,PARP14,PARP9,TNFSF10,TNFSF15 |
| Wound Healing Signaling Pathway | 2.714 | COL17A1,COL4A3,EDA,IL1RL1,IL1RN,MAPK8,MMP8,STAT1,TNFSF10,TNFSF15,TNFSF9 |
| Neuroinflammation Signaling Pathway | 2.53 | CCL2,CXCL10,GABRR2,GAD1,GRIN3A,HLA-G,IL34,IRF7,MAPK8,PLA2G2D,SLC6A12,STAT1,VCAM1 |
| Role of PKR in Interferon Induction and Antiviral Response | 2.449 | COLEC12,EIF2AK2,IFIH1,MAPK8,RIGI,STAT1,STAT2 |
| Oxidative Phosphorylation | 2.449 | ATP5ME,COX6C,COX7B,COX7C,NDUFS5,UQCRB |
| Retinoic acid Mediated Apoptosis Signaling | 2 | PARP12,PARP14,PARP9,TNFSF10 |
| ***MDD CRP>3 mg/L vs. controls*** | | |
| EIF2 Signaling | 4.69 | ACTA2,EIF2S1,RPL11,RPL17,RPL21,RPL22,RPL22L1,RPL23,RPL24,RPL26,RPL26L1,RPL27,RPL30,RPL31,RPL34,RPL35,RPL35A,RPL36A,RPL36AL,RPL37,RPL39,RPL41,RPL7,RPS10,RPS12,RPS14,RPS15A,RPS17,RPS18,RPS20,RPS21,RPS23,RPS24,RPS25,RPS27,RPS27A,RPS27L,RPS29,RPS3A,RPS4Y1,RPS6,RPS7 |
| Oxidative Phosphorylation | 4.69 | ATP5F1E,ATP5ME,ATP5MG,ATP5PF,ATP5PO,COX17,COX6B2,COX6C,COX7A2,COX7B,COX7C,NDUFA1,NDUFA4,NDUFA6,NDUFB1,NDUFB3,NDUFS4,NDUFS5,UQCR11,UQCRB,UQCRH,UQCRQ |
| Coronavirus Pathogenesis Pathway | -4.264 | OAS1,RB1,RPS10,RPS12,RPS14,RPS15A,RPS17,RPS18,RPS20,RPS21,RPS23,RPS24,RPS25,RPS27,RPS27A,RPS27L,RPS29,RPS3A,RPS4Y1,RPS6,RPS7,TRIM25 |
| Mitochondrial Dysfunction | -4.158 | ATP5F1E,ATP5ME,ATP5MG,ATP5PF,ATP5PO,CACNA1A,CACNA2D2,CAPN5,COX17,COX6B2,COX6C,COX7A2,COX7B,COX7C,GPX3,MT-ND6,NDUFA1,NDUFA4,NDUFA6,NDUFB1,NDUFB3,NDUFS4,NDUFS5,TOMM7,UQCR11,UQCRB,UQCRH,UQCRQ |
| Multiple Sclerosis Signaling Pathway | 2.524 | C1QA,C1QB,C1QC,CAPN5,EDA,GAS5,GRIN2C,GRIN3B,HLA-G,IL12A,IL2RA,IRF4,OSM,PARP9,TLR5,TNFSF10,TNFSF13B,TNFSF15,TNFSF9 |
| Granzyme A Signaling | -2.53 | LMNB1,MT-ND6,NDUFA1,NDUFA4,NDUFA6,NDUFB1,NDUFB3,NDUFS4,NDUFS5,PRF1 |
| Neutrophil Extracellular Trap Signaling Pathway | 2.041 | ATP5F1E,BPI,C1QA,C1QB,C1QC,CAMP,CASP4,CASP5,COL17A1,DEFA1 (includes others),DEFA4,ELANE,LTF,MT-ND6,NDUFA4,NDUFA6,NDUFB1,NDUFB3,NDUFS4,NDUFS5,TIMM8B,TOMM5,TOMM7,YES1 |
| HER-2 Signaling in Breast Cancer | -2 | COX17,COX6B2,COX6C,COX7A2,COX7B,COX7C,ELF3,FCER1G,HBEGF,ITGB4,ITGB8,MS4A2,NRG1,RB1,RPS6,YES1 |
| Wound Healing Signaling Pathway | 3 | ACTA2,ACVR2A,COL17A1,EDA,HBEGF,IL12A,IL1RN,ITGB4,LAMC1,MMP8,MST1R,OSM,TNFSF10,TNFSF13B,TNFSF15,TNFSF9 |
| Pathogen Induced Cytokine Storm Signaling Pathway | 3.9 | AIM2,CKLF,COL17A1,EDA,GZMB,IL12A,IL12RB2,IL1RN,IL23R,OSM,PPBP,PRF1,SLC2A5,SOCS3,TLR5,TNFSF10,TNFSF13B,TNFSF15,TNFSF9 |
| Systemic Lupus Erythematosus In B Cell Signaling Pathway | 2.333 | EDA,IGHD,IGHV1-2,IGHV3-13,IGHV3-49,IGHV4-39,IGKV2D-28,IGKV2D-40,IGKV3D-7,IGLV3-19,IGLV3-9,IGLV4-3,IGLV4-69,IGLV5-37,IGLV5-45,IL12A,OSM,TNFSF10,TNFSF13B,TNFSF15,TNFSF9,YES1 |
| Macrophage Classical Activation Signaling Pathway | 2.714 | BPI,EDA,IL12A,LY96,OSM,PARP9,SOCS3,TNFSF10,TNFSF13B,TNFSF15,TNFSF9 |
| S100 Family Signaling Pathway | 3.307 | ATP5F1E,ATP5PO,AVPR1A,CACNA1A,CACNA2D2,CELSR1,DEFA1 (includes others),DEFA4,FCGR1A,FCGR1BP,FFAR3,FZD5,GLP1R,GPR157,GPR162,GPR84,HBEGF,HCAR2,HCAR3,HRH1,LPAR1,LPAR6,MMP11,MMP25,MMP8,NAPSA,P2RY14,S100A12,S100A8,S100A9,S100P,SUCNR1,VIPR2 |
| CDX Gastrointestinal Cancer Signaling Pathway | -2.714 | CA1,EDA,FZD5,GATA6,HBEGF,IL12A,OSM,TNFSF10,TNFSF13B,TNFSF15,TNFSF9 |
| NOD1/2 Signaling Pathway | 3.162 | DEFA1 (includes others),DEFA4,EDA,IL12A,OSM,TLR5,TNFSF10,TNFSF13B,TNFSF15,TNFSF9 |
| ***MDD CRP 1-3 mg/L vs. MDD CRP<1*** | | |
| EIF2 Signaling | 3.5 | ACTG2,ATF3,EIF2AK2,PIK3R3,RASD1,RPL17,RPL21,RPL22L1,RPL23,RPL26,RPL26L1,RPL27,RPL31,RPL34,RPL36A,RPL39,RPL41,RPL7,RPS15A,RPS17,RPS21,RPS24,RPS27,RPS27L,RPS29,RPS3A,RPS7 |
| Role of Hypercytokinemia/hyperchemokinemia in the Pathogenesis of Influenza | 4 | CCL2,CXCL10,EIF2AK2,IFIT2,IFIT3,IFNB1,IL1RN,IRF7,ISG15,MX1,OAS1,OAS2,OAS3,RIGI,RSAD2,STAT1 |
| Interferon Signaling | 3.317 | IFI35,IFI6,IFIT1,IFIT3,IFITM1,IFITM3,IFNB1,ISG15,MX1,OAS1,STAT1 |
| Coronavirus Pathogenesis Pathway | -3.441 | CCL2,CCNE2,IFNB1,IRF7,OAS1,OAS2,OAS3,RIGI,RN7SL2,RPS15A,RPS17,RPS21,RPS24,RPS27,RPS27L,RPS29,RPS3A,RPS7,STAT1 |
| Role of Pattern Recognition Receptors in Recognition of Bacteria and Viruses | 2.828 | C1QA,C1QB,EDA,EIF2AK2,IFIH1,IFNB1,IRF7,OAS1,OAS2,OAS3,PIK3R3,RIGI,TNFSF10 |
| Mitotic Roles of Polo-Like Kinase | 2.236 | ANAPC10,CCNB1,CDC20,KIF11,KIF23,PKMYT1,PPM1J |
| Role of PKR in Interferon Induction and Antiviral Response | 2.828 | ATF3,CASP5,EIF2AK2,FAS,FCGR1A,IFIH1,IFNB1,RIGI,SCARA5,STAT1 |
| Pathogen Induced Cytokine Storm Signaling Pathway | 3.5 | AIM2,CCL2,CCL3L1,COL17A1,CXCL10,DHX58,EDA,IFIH1,IFNB1,IL1RN,IRF7,RIGI,SOCS3,STAT1,TNFSF10,ZBP1 |
| Macrophage Classical Activation Signaling Pathway | 2.53 | CXCL10,EDA,GBP4,IFNB1,LY96,PARP14,PARP9,SOCS3,STAT1,TNFSF10 |
| Retinoic acid Mediated Apoptosis Signaling | 2.236 | IFNB1,PARP12,PARP14,PARP9,TNFSF10 |
| Multiple Sclerosis Signaling Pathway | 2.714 | C1QA,C1QB,C2,EDA,FAS,PARP12,PARP14,PARP9,PLAU,STAT1,TNFSF10 |
| Oxidative Phosphorylation | 2.646 | ATP5ME,COX6C,COX7B,COX7C,NDUFB1,NDUFB3,UQCRB |
| Necroptosis Signaling Pathway | 2.828 | EIF2AK2,FAS,IFNB1,STAT1,TIMM10,TIMM8B,TNFSF10,ZBP1 |
| ***MDD CRP>3 mg/L vs. MDD CRP<1 mg/L*** | | |
| EIF2 Signaling | 4.796 | ACTA2,FAU,RASD1,RPL11,RPL17,RPL21,RPL22,RPL22L1,RPL23,RPL24,RPL26,RPL26L1,RPL27,RPL30,RPL31,RPL32,RPL34,RPL35,RPL35A,RPL36A,RPL37,RPL39,RPL39L,RPL41,RPL7,RPS10,RPS12,RPS14,RPS15A,RPS17,RPS18,RPS20,RPS21,RPS23,RPS24,RPS25,RPS27,RPS27A,RPS27L,RPS29,RPS3A,RPS6,RPS7 |
| Oxidative Phosphorylation | 2.785 | ATP5F1E,ATP5ME,ATP5MG,ATP5PF,ATP5PO,COX6B2,COX6C,COX7A2,COX7B,COX7C,MT-ATP6,MT-CO1,MT-CO2,MT-CYB,MT-ND1,MT-ND4,MT-ND5,NDUFA1,NDUFA4,NDUFB1,NDUFB3,NDUFB6,NDUFS4,NDUFS5,UQCR11,UQCRB,UQCRH,UQCRQ,VPS9D1 |
| Mitochondrial Dysfunction | -2.53 | ATP5F1E,ATP5ME,ATP5MG,ATP5PF,ATP5PO,CACNA1A,CACNA1E,CACNB4,CACNG6,CAPN5,COX6B2,COX6C,COX7A2,COX7B,COX7C,CREB5,GPX1,GPX3,MT-ATP6,MT-CO1,MT-CO2,MT-CYB,MT-ND1,MT-ND4,MT-ND5,NDUFA1,NDUFA4,NDUFB1,NDUFB3,NDUFB6,NDUFS4,NDUFS5,PPARGC1A,SNCA,TOMM7,UQCR11,UQCRB,UQCRH,UQCRQ,VPS9D1 |
| Coronavirus Pathogenesis Pathway | -4.315 | CASP1,CCNE2,FAU,IFNB1,IL1B,RNU1-1,RNU2-1,RPS10,RPS12,RPS14,RPS15A,RPS17,RPS18,RPS20,RPS21,RPS23,RPS24,RPS25,RPS27,RPS27A,RPS27L,RPS29,RPS3A,RPS6,RPS7,TRIM25 |
| Neutrophil Extracellular Trap Signaling Pathway | 2.535 | ATP5F1E,BPI,C1QA,C1QB,CAMP,CASP1,CASP4,CASP5,COL13A1,COL17A1,COL9A2,DEFA1 (includes others),DEFA4,FCAR,IFNB1,IL1B,JCHAIN,LTF,MMP9,MT-ATP6,MT-CYB,MT-ND1,MT-ND4,MT-ND5,NDUFA4,NDUFB1,NDUFB3,NDUFB6,NDUFS4,NDUFS5,PLCH1,TIMM8B,TOMM5,TOMM7,YES1 |
| Pyroptosis Signaling Pathway | 2.309 | AIM2,CASP1,CASP4,CASP5,GAS5,GBP5,IL1B,NAIP,NGFR,NLRP5,NOL3,TLR5 |
| Multiple Sclerosis Signaling Pathway | 2.236 | C1QA,C1QB,CAPN5,CASP1,EDA,FAS,GAS5,HLA-DQB1,IL1B,LEP,MMP9,OSM,PARP9,PLAU,SLC4A1,TLR5,TNFSF10,TNFSF11,TNFSF13B,TNFSF9 |
| S100 Family Signaling Pathway | 2.38 | ADGRA3,ADGRG3,ATP5F1E,ATP5PO,CACNA1A,CACNA1E,CACNB4,CACNG6,CMKLR1,CREB5,DEFA1 (includes others),DEFA4,ERBB2,FCGR1A,FCGR1BP,FCGR2A,FFAR2,FFAR3,FPR3,FZD2,FZD5,GPR162,GPR171,GPR173,GPR83,GPR84,HBEGF,HCAR2,HCAR3,HRH4,IFNB1,IL1B,JCHAIN,LPAR3,LPAR6,MMP24,MMP8,MMP9,P2RY2,PLCH1,PTGDR2,RTKN,S100A12,S100A8,S100A9,SMAD6,SUCNR1,TRPV6,UTS2R,VIPR2,XCR1 |
| Inflammasome pathway | 2.236 | AIM2,CASP1,CASP5,IL1B,NAIP |
| Pathogen Induced Cytokine Storm Signaling Pathway | 2.502 | AIM2,CASP1,CCL3L1,CKLF,COL13A1,COL17A1,COL9A2,EDA,GZMB,HLA-DQB1,IFNB1,IL1B,LEP,NGFR,NLRP5,OSM,PPBP,PRF1,SLC2A3,SLC2A5,SOCS3,SRGN,TLR5,TNFSF10,TNFSF11,TNFSF13B,TNFSF9 |
| Role of Pattern Recognition Receptors in Recognition of Bacteria and Viruses | 2.646 | C1QA,C1QB,CASP1,CLEC6A,EDA,IFNB1,IL1B,LEP,OSM,TLR5,TNFSF10,TNFSF11,TNFSF13B,TNFSF9 |
| Mitotic Roles of Polo-Like Kinase | 2 | ANAPC10,CCNB1,CCNB2,CDC20,PKMYT1,PPM1J,PPP2R3A |
| NOD1/2 Signaling Pathway | 2.496 | CASP1,DEFA1 (includes others),DEFA4,EDA,IFNB1,IL1B,LEP,OSM,TLR5,TNFSF10,TNFSF11,TNFSF13B,TNFSF9 |
| ***MDD CRP>3 mg/L vs. MDD CRP 1-3 mg/L*** | | |
| Role of Hypercytokinemia/hyperchemokinemia in the Pathogenesis of Influenza | -2.828 | AREG,CCL2,IFIT2,ISG15,MX1,OAS2,OAS3,RSAD2 |
| IL-33 Signaling Pathway | -2.333 | AREG,BCL2L1,CASP5,CCL2,IL1RL1,KIT,MAPK8,TPSAB1/TPSB2,VCAM1 |
| ***MDD CRP<1 mg/L vs. (all) controls*** | | |
| Estrogen-mediated S-phase Entry | -2 | CCNA2,CDK1,E2F1,E2F8 |
| Cyclins and Cell Cycle Regulation | -2.449 | CCNA2,CCNB2,CDK1,E2F1,E2F8,PPP2R3A |
| Role of CHK Proteins in Cell Cycle Checkpoint Control | 2 | CDK1,E2F1,E2F8,PPP2R3A |
| ***MDD CRP<1 mg/L vs. controls CRP<1*** | | |
| Inhibition of Matrix Metalloproteases | -2 | MMP19,MMP25,MMP28,MMP8,SDC1 |
| Estrogen-mediated S-phase Entry | -2 | CCNA2,E2F1,E2F2,E2F7 |
| STAT3 Pathway | 2.449 | BMPR2,FLT1,IGF1R,IL1RL1,IL5RA,INSR,MAP3K9,MAPK11,MAPK8 |
| Cyclins and Cell Cycle Regulation | -2.236 | CCNA2,CCNB2,CDC34,E2F1,E2F2,E2F7 |
| Transcripts are selected for p<.05 and FC>\|1.2\|; pathways are selected for z-scores ≥\|2\| and listed based on p-values (smaller to the top), all p<0.05. | | |

# ***Supplementary Table S6:*** *Canonical pathways differentially activated in group comparisons (treatment-based MDD groupings and whole MDD cases)*

| **Ingenuity Canonical Pathways** | **z-score** | **Molecules** |
| --- | --- | --- |
| ***MDD unmedicated vs. controls*** | | |
| EIF2 Signaling | 3.357 | EIF2AK2,MYCN,PIK3R3,RPL11,RPL17,RPL21,RPL22,RPL23,RPL26,RPL31,RPL34,RPL36A,RPL37,RPL39,RPL41,RPS17,RPS23,RPS24,RPS25,RPS27,RPS27A,RPS27L,RPS3A,RPS7 |
| Interferon Signaling | 2.333 | IFI35,IFI6,IFIT1,IFIT3,IFITM1,ISG15,MX1,OAS1,SOCS1 |
| Role of Hypercytokinemia/hyperchemokinemia in the Pathogenesis of Influenza | 3.162 | EIF2AK2,IFIT2,IFIT3,IRF7,ISG15,MX1,OAS1,OAS2,OAS3,RSAD2 |
| Coronavirus Pathogenesis Pathway | -2.84 | IRF7,OAS1,OAS2,OAS3,RN7SL1,RN7SL2,RPS17,RPS23,RPS24,RPS25,RPS27,RPS27A,RPS27L,RPS3A,RPS7 |
| Role of NFAT in Regulation of the Immune Response | 2 | CD3D,FCGR1A,FCGR3A/FCGR3B,IGHG1,IGHG3,IGHV3-13,IGHV3-30,IGHV3-49,IGHV3-53,IGHV3-64,IGKV1-33,IGKV1-8,IGKV2D-28,IGKV3D-11,IGLC7,IGLV3-10,IGLV3-25,IGLV3-9,PIK3R3,TRAV2,TRBV13,TRBV30,TRBV4-1,TRBV7-3,TRGV8 |
| Oxidative Phosphorylation | 2.828 | ATP5ME,COX7B,NDUFA1,NDUFB3,NDUFS4,NDUFS5,UQCRB,UQCRH |
| Role of Pattern Recognition Receptors in Recognition of Bacteria and Viruses | 2.236 | C1QC,EIF2AK2,IFIH1,IRF7,OAS1,OAS2,OAS3,PIK3R3,TNFSF10 |
| Mitochondrial Dysfunction | -3.357 | ATP5ME,CACNB3,CAPN5,CLIC2,COX7B,MGST1,NDUFA1,NDUFB3,NDUFS4,NDUFS5,PIK3R3,SNCA,TOMM7,UQCRB,UQCRH |
| ***MDD responders vs. controls*** | | |
| Role of Hypercytokinemia/hyperchemokinemia in the Pathogenesis of Influenza | 2.887 | AREG,CCL2,CXCL10,IFIT2,IFIT3,IL12A,IL1RN,ISG15,OAS1,OAS2,STAT1,TLR7 |
| Systemic Lupus Erythematosus In B Cell Signaling Pathway | 2.333 | IFIT2,IFIT3,IGHG1,IGHG3,IGHV1-18,IGHV2-5,IGHV3-49,IGKV1-33,IGKV1D-12,IGKV3-20,IGKV3-7,IGKV3D-11,IGLC1,IGLV3-10,IGLV3-21,IGLV3-25,IGLV5-37,IL12A,ISG15,SHE,STAT1,TLR7,TNFSF10 |
| Interferon Signaling | 2 | IFIT3,ISG15,OAS1,STAT1 |
| Colorectal Cancer Metastasis Signaling | 2.333 | CDH1,FZD5,GNAZ,LRP5,MAPK8,MMP11,MMP25,MMP8,STAT1,TLR7,WNT5B |
| Tumour Microenvironment Pathway | 2.121 | CCL2,CD274,CSF1,HLA-G,IDO1,MMP11,MMP25,MMP8 |
| Pathogen Induced Cytokine Storm Signaling Pathway | 2.887 | CCL2,CDH1,CXCL10,IL12A,IL1RN,IL23R,MAPK8,STAT1,STXBP2,TLR7,TNFSF10,ZBP1 |
| T Cell Exhaustion Signaling Pathway | 2 | ACVR2A,CD274,HLA-G,IL12A,MAPK8,STAT1,TRAV10,TRAV12-1,TRAV34,TRBV15,TRBV24-1,TRBV9 |
| Role of MAPK Signaling in Inhibiting the Pathogenesis of Influenza | 2 | CCL2,CXCL10,MAPK8,PLA2G4A |
| Neuroprotective Role of THOP1 in Alzheimer’s Disease | 2 | HLA-G,HTRA3,PRSS53,TPSAB1/TPSB2,TPSD1 |
| Wound Healing Signaling Pathway | 2.828 | ACVR2A,IL12A,IL1RN,MAPK8,MMP8,STAT1,TNFSF10,TPSAB1/TPSB2 |
| ***MDD non-responders vs. controls*** | | |
| CREB Signaling in Neurons | 2.683 | ADCY2,ADCY4,CACNA1F,CACNA2D2,CACNG6,CACNG8,CELSR1,DRD4,GPRC5B,GRIA1,GRID1,GRIN2B,GRIN2D,GRM2,LPAR1,NMUR1,NPBWR1,NTRK2,P2RY14,TSHR |
| S100 Family Signaling Pathway | 2.294 | CACNA1F,CACNA2D2,CACNG6,CACNG8,CELSR1,DRD4,GPRC5B,GRM2,HBEGF,IGHG3,LPAR1,MMP19,MMP8,NMUR1,NPBWR1,NTRK2,P2RY14,PLA2G2D,S100A8,TSHR,WNT10A,WNT11,WNT6 |
| Systemic Lupus Erythematosus In B Cell Signaling Pathway | 2 | EDA,IGHG3,IGHV3-49,IGHV3-64,IGHV4-39,IGKV2D-28,IGKV3D-11,IGKV3D-7,IGLV1-36,IGLV3-19,IGLV5-37,IL12A,SHE,TNFRSF13C |
| EIF2 Signaling | 2.236 | RPL21,RPL22,RPL26,RPL36A,RPL41,RPS24,RPS25,RPS28 |
| ***MDD responders vs. MDD non-responders*** | | |
| IL-4 Signaling | -2.5 | COL5A1,COL6A1,CREB1,FCER2,RICTOR,TRAV12-1,TRAV17,TRAV21,TRAV23DV6,TRAV34,TRAV4,TRBV15,TRBV6-6,TRBV7-3,TRBV7-4,TRBV9 |
| Chaperone Mediated Autophagy Signaling Pathway | -2 | MMP11,MMP17,NOS3,POU5F1,RICTOR,TRAV12-1,TRAV17,TRAV21,TRAV23DV6,TRAV34,TRAV4,TRBV15,TRBV6-6,TRBV7-3,TRBV7-4,TRBV9 |
| G Protein Signaling Mediated by Tubby | -2.714 | TRAV12-1,TRAV17,TRAV21,TRAV23DV6,TRAV34,TRAV4,TRBV15,TRBV6-6,TRBV7-3,TRBV7-4,TRBV9 |
| Neuroprotective Role of THOP1 in Alzheimer’s Disease | 2 | CREB1,PNOC,PRSS23,PRSS53,TPSAB1/TPSB2,TPSD1 |
| HOTAIR Regulatory Pathway | -2.449 | AGO3,MMP11,MMP17,REL,TWIST2,WNT10A,WNT11 |
| Gα12/13 Signaling | -2.236 | CDH26,F2RL3,LPAR3,MYL6B,REL,TBXA2R |
| Role of MAPK Signaling in Inhibiting the Pathogenesis of Influenza | 2 | ABHD3,CCL2,CXCL10,PTGS2 |
| FAK Signaling | -2.837 | ADGRB2,DRD4,F2RL3,GPR150,GPRC5B,HTR6,IL18RAP,LPAR3,PAK6,TBXA2R,TRAV12-1,TRAV17,TRAV21,TRAV23DV6,TRAV34,TRAV4,TRBV15,TRBV6-6,TRBV7-3,TRBV7-4,TRBV9 |
| ***MDD unmedicated vs. MDD responders*** | | |
| G Protein Signaling Mediated by Tubby | 2.111 | GNA11,GNAL,TRAV8-1,TRBV11-1,TRBV19,TRBV25-1,TRBV7-3,TRBV9,TRGC2,TRGV2,TRGV8 |
| CTLA4 Signaling in Cytotoxic T Lymphocytes | -3.051 | CD8A,CD8B,PIK3R3,PPP2R2B,TRAV8-1,TRBV11-1,TRBV19,TRBV25-1,TRBV7-3,TRBV9,TRGC2,TRGV2,TRGV8 |
| FAK Signaling | 2.558 | ADGRF3,AGO2,BCAR3,CDH1,ELF3,F2R,GPR15,GPR25,GPR55,P2RY6,PIK3R3,SOCS1,TRAV8-1,TRBV11-1,TRBV19,TRBV25-1,TRBV7-3,TRBV9,TRGC2,TRGV2,TRGV8,TSHR |
| T Cell Receptor Signaling | 3 | CD8A,CD8B,PIK3R3,TRAV8-1,TRBV11-1,TRBV19,TRBV25-1,TRBV7-3,TRBV9,TRGC2,TRGV2,TRGV8 |
| IL-4 Signaling | 2.714 | PIK3R3,SOCS1,TRAV8-1,TRBV11-1,TRBV19,TRBV25-1,TRBV7-3,TRBV9,TRGC2,TRGV2,TRGV8 |
| Role of Hypercytokinemia/hyperchemokinemia in the Pathogenesis of Influenza | 2 | AREG,CCL4,CCL5,RSAD2 |
| Chaperone Mediated Autophagy Signaling Pathway | 2.309 | NOS3,PIK3R3,SNCA,TRAV8-1,TRBV11-1,TRBV19,TRBV25-1,TRBV7-3,TRBV9,TRGC2,TRGV2,TRGV8 |
| ***MDD unmedicated vs. MDD non-responders*** | | |
| Role of Hypercytokinemia/hyperchemokinemia in the Pathogenesis of Influenza | 3 | AREG,CCL4,CCL5,EIF2AK2,IFIT2,IFIT3,OAS1,RSAD2,TLR3 |
| Immunogenic Cell Death Signaling Pathway | 2.449 | GZMA,GZMB,GZMH,NGFR,PRF1,TLR3 |
| Role of Pattern Recognition Receptors in Recognition of Bacteria and Viruses | 2.449 | C1QB,C1QC,CCL5,EIF2AK2,LEP,OAS1,PIK3R3,TLR3 |
| ***MDD all cases vs. controls*** | | |
| Role of Hypercytokinemia/hyperchemokinemia in the Pathogenesis of Influenza | 3.162 | CXCL10,IFIT2,IFIT3,IL12A,IL1RN,ISG15,OAS1,OAS2,OAS3,RSAD2 |
| Interferon Signaling | 2 | IFIT1,IFIT3,ISG15,OAS1 |
| S100 Family Signaling Pathway | 2.524 | CACNA1F,CELSR1,FCGR1A,HTR7,IGHG1,IGHG3,LGR4,LPAR1,MMP19,MMP8,NAPSA,NPBWR1,P2RY14,PLA2G2D,S100A8,SUCNR1,TSHR,VCAM1,WNT11 |
| EIF2 Signaling | 2.449 | RPL23,RPL26,RPL31,RPL34,RPL36A,RPL41,RPS24 |
| Transcripts are selected for p<.05 and FC>\|1.2\|; pathways are selected for z-scores ≥\|2\| and listed based on p-values (smaller to the top), all p<0.05. | | |

# ***Supplementary Table S7:*** *Canonical pathways differentially activated in group comparisons (merging CRP-based and treatment-based MDD groups)*

| **Ingenuity Canonical Pathways** | **z-score** | **Molecules** |
| --- | --- | --- |
| ***MDD non-responders/unmedicated with CRP>1 vs. controls*** | | |
| Eukaryotic Translation Elongation | 5.831 | EEF1B2,RPL11,RPL17,RPL21,RPL22,RPL22L1,RPL23,RPL24,RPL26,RPL26L1,RPL27,RPL31,RPL34,RPL35,RPL35A,RPL36A,RPL36AL,RPL37,RPL39,RPL41,RPL7,RPS15A,RPS17,RPS20,RPS21,RPS23,RPS24,RPS25,RPS27,RPS27A,RPS27L,RPS29,RPS3A,RPS7 |
| Eukaryotic Translation Termination | 5.745 | RPL11,RPL17,RPL21,RPL22,RPL22L1,RPL23,RPL24,RPL26,RPL26L1,RPL27,RPL31,RPL34,RPL35,RPL35A,RPL36A,RPL36AL,RPL37,RPL39,RPL41,RPL7,RPS15A,RPS17,RPS20,RPS21,RPS23,RPS24,RPS25,RPS27,RPS27A,RPS27L,RPS29,RPS3A,RPS7 |
| Response of EIF2AK4 (GCN2) to amino acid deficiency | 5.831 | EIF2S1,RPL11,RPL17,RPL21,RPL22,RPL22L1,RPL23,RPL24,RPL26,RPL26L1,RPL27,RPL31,RPL34,RPL35,RPL35A,RPL36A,RPL36AL,RPL37,RPL39,RPL41,RPL7,RPS15A,RPS17,RPS20,RPS21,RPS23,RPS24,RPS25,RPS27,RPS27A,RPS27L,RPS29,RPS3A,RPS7 |
| Selenoamino acid metabolism | 5.831 | HNMT,RPL11,RPL17,RPL21,RPL22,RPL22L1,RPL23,RPL24,RPL26,RPL26L1,RPL27,RPL31,RPL34,RPL35,RPL35A,RPL36A,RPL36AL,RPL37,RPL39,RPL41,RPL7,RPS15A,RPS17,RPS20,RPS21,RPS23,RPS24,RPS25,RPS27,RPS27A,RPS27L,RPS29,RPS3A,RPS7 |
| Eukaryotic Translation Initiation | 5.831 | EIF2S1,RPL11,RPL17,RPL21,RPL22,RPL22L1,RPL23,RPL24,RPL26,RPL26L1,RPL27,RPL31,RPL34,RPL35,RPL35A,RPL36A,RPL36AL,RPL37,RPL39,RPL41,RPL7,RPS15A,RPS17,RPS20,RPS21,RPS23,RPS24,RPS25,RPS27,RPS27A,RPS27L,RPS29,RPS3A,RPS7 |
| SRP-dependent cotranslational protein targeting to membrane | 5.745 | RPL11,RPL17,RPL21,RPL22,RPL22L1,RPL23,RPL24,RPL26,RPL26L1,RPL27,RPL31,RPL34,RPL35,RPL35A,RPL36A,RPL36AL,RPL37,RPL39,RPL41,RPL7,RPS15A,RPS17,RPS20,RPS21,RPS23,RPS24,RPS25,RPS27,RPS27A,RPS27L,RPS29,RPS3A,RPS7 |
| Nonsense-Mediated Decay (NMD) | 5.745 | RPL11,RPL17,RPL21,RPL22,RPL22L1,RPL23,RPL24,RPL26,RPL26L1,RPL27,RPL31,RPL34,RPL35,RPL35A,RPL36A,RPL36AL,RPL37,RPL39,RPL41,RPL7,RPS15A,RPS17,RPS20,RPS21,RPS23,RPS24,RPS25,RPS27,RPS27A,RPS27L,RPS29,RPS3A,RPS7 |
| Major pathway of rRNA processing in the nucleolus and cytosol | 5.745 | RPL11,RPL17,RPL21,RPL22,RPL22L1,RPL23,RPL24,RPL26,RPL26L1,RPL27,RPL31,RPL34,RPL35,RPL35A,RPL36A,RPL36AL,RPL37,RPL39,RPL41,RPL7,RPS15A,RPS17,RPS20,RPS21,RPS23,RPS24,RPS25,RPS27,RPS27A,RPS27L,RPS29,RPS3A,RPS7 |
| EIF2 Signaling | 4.472 | ACTA2,EIF2S1,RPL11,RPL17,RPL21,RPL22,RPL22L1,RPL23,RPL24,RPL26,RPL26L1,RPL27,RPL31,RPL34,RPL35,RPL35A,RPL36A,RPL36AL,RPL37,RPL39,RPL41,RPL7,RPS15A,RPS17,RPS20,RPS21,RPS23,RPS24,RPS25,RPS27,RPS27A,RPS27L,RPS29,RPS3A,RPS7 |
| Interferon alpha/beta signaling | 4.243 | IFI27,IFI35,IFI6,IFIT1,IFIT2,IFIT3,IFIT5,IFITM1,ISG15,MX1,OAS1,OAS2,OAS3,OASL,RSAD2,SOCS1,USP18,XAF1 |
| Electron transport, ATP synthesis, and heat production by uncoupling proteins | 4.359 | ATP5F1E,ATP5ME,ATP5PF,ATP5PO,COX14,COX16,COX6C,COX7B,COX7C,NDUFA1,NDUFA4,NDUFAF2,NDUFB3,NDUFS4,NDUFS5,SCO2,UQCRB,UQCRH,UQCRQ |
| Oxidative Phosphorylation | 4 | ATP5F1E,ATP5ME,ATP5PF,ATP5PO,COX6C,COX7A2,COX7B,COX7C,NDUFA1,NDUFA4,NDUFB3,NDUFS4,NDUFS5,UQCRB,UQCRH,UQCRQ |
| Cell surface interactions at the vascular wall | 3.13 | CEACAM6,CEACAM8,GAS6,IGHV1-2,IGHV1-46,IGHV3-13,IGHV3-33,IGHV3-53,IGHV4-39,IGKV4-1,IGLV2-23,IGLV3-19,IGLV4-69,IGLV5-45,IGLV7-43,OLR1,PROC,SDC2,VPREB3,YES1 |
| Immunoregulatory interactions between a Lymphoid and a non-Lymphoid cell | 2.982 | CLEC2B,FCGR1A,IFITM1,IGHV1-2,IGHV1-46,IGHV3-13,IGHV3-33,IGHV3-53,IGHV4-39,IGKV4-1,IGLV2-23,IGLV3-19,IGLV4-69,IGLV5-45,IGLV7-43,KIR2DL1/KIR2DL3,KIR3DL1,KLRC1,NECTIN2 |
| Interferon Signaling | 2.333 | IFI35,IFI6,IFIT1,IFIT3,IFITM1,ISG15,MX1,OAS1,SOCS1 |
| Binding and Uptake of Ligands by Scavenger Receptors | 3.606 | HP,IGHV1-2,IGHV1-46,IGHV3-13,IGHV3-33,IGHV3-53,IGHV4-39,IGKV4-1,IGLV2-23,IGLV3-19,IGLV4-69,IGLV5-45,IGLV7-43 |
| Fcgamma receptor (FCGR) dependent phagocytosis | 2.84 | FCGR1A,IGHG3,IGHV1-2,IGHV1-46,IGHV3-13,IGHV3-33,IGHV3-53,IGHV4-39,IGKV4-1,IGLV2-23,IGLV3-19,IGLV4-69,IGLV5-45,IGLV7-43,YES1 |
| Coronavirus Pathogenesis Pathway | -4 | OAS1,OAS2,OAS3,RPS15A,RPS17,RPS20,RPS21,RPS23,RPS24,RPS25,RPS27,RPS27A,RPS27L,RPS29,RPS3A,RPS7 |
| Communication between Innate and Adaptive Immune Cells | 3.273 | IGHG3,IGHV1-2,IGHV1-46,IGHV3-13,IGHV3-33,IGHV3-49,IGHV3-53,IGHV4-39,IGHV4-4,IGHV6-1,IGKV3D-11,IGKV4-1,IGLV2-23,IGLV3-19,IGLV3-9,IGLV4-69,IGLV5-45,IGLV7-43,IL1RN,TLR5,TNFSF13B,TRAV23DV6,TRBV7-3,TRDV2,TRGC1,TRGV9 |
| Mitochondrial Dysfunction | -3.71 | ATP5F1E,ATP5ME,ATP5PF,ATP5PO,BACE2,CAPN5,COX6C,COX7A2,COX7B,COX7C,GPX3,MGST1,NDUFA1,NDUFA4,NDUFB3,NDUFS4,NDUFS5,TOMM7,UQCRB,UQCRH,UQCRQ |
| Neutrophil degranulation | 3.8 | ABCA13,ARG1,BPI,CAMP,CEACAM6,CEACAM8,CHI3L1,CRISP3,DEFA1 (includes others),DEFA4,FABP5,GPR84,HP,LCN2,LTF,MGST1,MMP8,OLFM4,OLR1,PPBP,PTPRN2,S100A12,S100A8,SLC27A2,TNFAIP6 |
| OAS antiviral response | 2 | OAS1,OAS2,OAS3,OASL |
| Role of Hypercytokinemia/hyperchemokinemia in the Pathogenesis of Influenza | 3 | IFIT2,IFIT3,IL1RN,ISG15,MX1,OAS1,OAS2,OAS3,RSAD2 |
| Fc epsilon receptor (FCERI) signaling | 3.606 | IGHV1-2,IGHV1-46,IGHV3-13,IGHV3-33,IGHV3-53,IGHV4-39,IGKV4-1,IGLV2-23,IGLV3-19,IGLV4-69,IGLV5-45,IGLV7-43,RPS27A,SEM1 |
| TP53 Regulates Metabolic Genes | 3 | COX14,COX16,COX6C,COX7B,COX7C,NDUFA4,PRDX1,SCO2,TXN |
| Neutrophil Extracellular Trap Signaling Pathway | 2.236 | ATP5F1E,BPI,C1QA,C1QB,CAMP,CASP5,COL4A3,DEFA1 (includes others),DEFA4,IGHG3,LTF,NDUFA4,NDUFB3,NDUFS4,NDUFS5,TIMM10,TIMM8B,TOMM5,TOMM7,YES1 |
| Signaling by the B Cell Receptor (BCR) | 3.464 | IGHV1-2,IGHV1-46,IGHV3-13,IGHV3-33,IGHV3-53,IGHV4-39,IGKV4-1,IGLV2-23,IGLV3-19,IGLV7-43,RPS27A,SEM1 |
| Cytoprotection by HMOX1 | 2.646 | COX14,COX16,COX6C,COX7B,COX7C,NDUFA4,SCO2 |
| Antimicrobial peptides | 2.236 | BPI,CAMP,LCN2,LTF,S100A8 |
| Mitochondrial translation | 2.828 | MRPL22,MRPL39,MRPL47,MRPL51,MRPL53,MRPS18C,MRPS28,MRPS33 |
| Interferon gamma signaling | 2.646 | FCGR1A,OAS1,OAS2,OAS3,OASL,SOCS1,TRIM22 |
| Cristae formation | 2 | ATP5F1E,ATP5ME,ATP5PF,ATP5PO |
| ISG15 antiviral mechanism | 2.236 | HERC5,IFIT1,ISG15,MX1,RPS27A |
| Granzyme A Signaling | -2.236 | NDUFA1,NDUFA4,NDUFB3,NDUFS4,NDUFS5 |
| DNA Damage Bypass | 2 | ISG15,POLE4,RBX1,RPS27A |
| S100 Family Signaling Pathway | 2.294 | ADGRB2,ATP5F1E,ATP5PO,CELSR1,DEFA1 (includes others),DEFA4,EGF,FCGR1A,FFAR3,GPR84,GPRC5B,GRM2,HCAR3,IGHG3,LGR4,LPAR6,MMP8,NMUR1,P2RY14,P2RY6,S100A12,S100A8,WNT10A |
| Multiple Sclerosis Signaling Pathway | 2.333 | C1QA,C1QB,CAPN5,EDA,IL2RA,PARP14,TLR5,TNFSF10,TNFSF13B |
| Metabolism of non-coding RNA | 2 | SNRPD1,SNRPD2,SNRPE,SNRPG |
| Sirtuin Signaling Pathway | -2.236 | ATP5F1E,ATP5PF,NDUFA1,NDUFA4,NDUFB3,NDUFS4,NDUFS5,TIMM10,TIMM8B,TOMM5,TOMM7 |
| Synthesis of DNA | 2.449 | ANAPC10,POLE4,RBX1,RPS27A,SEM1,UBE2C |
| Mitochondrial protein import | 2 | TIMM10,TIMM8B,TOMM5,TOMM7 |
| ***MDD responders with CRP>1 vs. controls*** | | |
| Interferon alpha/beta signaling | 4.264 | HLA-G,IFI35,IFI6,IFIT1,IFIT2,IFIT3,IFIT5,IFITM1,IRF7,ISG15,MX1,MX2,OAS1,OAS2,OAS3,OASL,RSAD2,SOCS3,STAT1,STAT2,USP18,XAF1 |
| Role of Hypercytokinemia/hyperchemokinemia in the Pathogenesis of Influenza | 4.243 | CXCL10,EIF2AK2,IFIT2,IFIT3,IFNLR1,IL1B,IL1RN,IRF7,ISG15,MX1,OAS1,OAS2,OAS3,RIGI,RSAD2,STAT1,STAT2,TLR7 |
| Eukaryotic Translation Elongation | 4.243 | EEF1B2,RPL17,RPL21,RPL23,RPL26,RPL31,RPL34,RPL36A,RPL39,RPL41,RPS15A,RPS17,RPS24,RPS27,RPS27L,RPS29,RPS3A,RPS7 |
| Response of EIF2AK4 (GCN2) to amino acid deficiency | 3.771 | RPL17,RPL21,RPL23,RPL26,RPL31,RPL34,RPL36A,RPL39,RPL41,RPS15A,RPS17,RPS24,RPS27,RPS27L,RPS29,RPS3A,RPS7,TRIB3 |
| Eukaryotic Translation Termination | 4.123 | RPL17,RPL21,RPL23,RPL26,RPL31,RPL34,RPL36A,RPL39,RPL41,RPS15A,RPS17,RPS24,RPS27,RPS27L,RPS29,RPS3A,RPS7 |
| Interferon gamma signaling | 3.638 | FCGR1A,GBP1,GBP4,GBP5,HLA-G,IRF7,MT2A,OAS1,OAS2,OAS3,OASL,PML,SOCS3,STAT1,TRIM22,TRIM25,TRIM5 |
| Selenoamino acid metabolism | 4.123 | RPL17,RPL21,RPL23,RPL26,RPL31,RPL34,RPL36A,RPL39,RPL41,RPS15A,RPS17,RPS24,RPS27,RPS27L,RPS29,RPS3A,RPS7 |
| Coronavirus Pathogenesis Pathway | -3.411 | IL1B,IRF7,OAS1,OAS2,OAS3,RB1,RIGI,RN7SL1,RN7SL2,RPS15A,RPS17,RPS24,RPS27,RPS27L,RPS29,RPS3A,RPS7,STAT1,STAT2,TLR7,TRIM25,ZC3HAV1 |
| SRP-dependent cotranslational protein targeting to membrane | 4.123 | RPL17,RPL21,RPL23,RPL26,RPL31,RPL34,RPL36A,RPL39,RPL41,RPS15A,RPS17,RPS24,RPS27,RPS27L,RPS29,RPS3A,RPS7 |
| Nonsense-Mediated Decay (NMD) | 4.123 | RPL17,RPL21,RPL23,RPL26,RPL31,RPL34,RPL36A,RPL39,RPL41,RPS15A,RPS17,RPS24,RPS27,RPS27L,RPS29,RPS3A,RPS7 |
| Eukaryotic Translation Initiation | 4.123 | RPL17,RPL21,RPL23,RPL26,RPL31,RPL34,RPL36A,RPL39,RPL41,RPS15A,RPS17,RPS24,RPS27,RPS27L,RPS29,RPS3A,RPS7 |
| Interferon Signaling | 3.162 | IFI35,IFI6,IFIT1,IFIT3,IFITM1,ISG15,MX1,OAS1,STAT1,STAT2 |
| ISGylation Signaling Pathway | 2.84 | CHMP5,DTX3L,EIF2AK2,HERC5,IFIH1,IFNLR1,IRF7,ISG15,RIGI,STAT1,STAT2,TLR7,TRIM25,UBE2L6,USP18 |
| EIF2 Signaling | 2.887 | EIF2AK2,RPL17,RPL21,RPL23,RPL26,RPL31,RPL34,RPL36A,RPL39,RPL41,RPS15A,RPS17,RPS24,RPS27,RPS27L,RPS29,RPS3A,RPS7,TRIB3,WARS1 |
| Major pathway of rRNA processing in the nucleolus and cytosol | 3.771 | MPHOSPH6,RPL17,RPL21,RPL23,RPL26,RPL31,RPL34,RPL36A,RPL39,RPL41,RPS15A,RPS17,RPS24,RPS27,RPS27L,RPS29,RPS3A,RPS7 |
| OAS antiviral response | 2.236 | OAS1,OAS2,OAS3,OASL,RIGI |
| ISG15 antiviral mechanism | 3.162 | EIF2AK2,HERC5,IFIT1,ISG15,MX1,MX2,RIGI,STAT1,TRIM25,UBE2L6 |
| Role of Pattern Recognition Receptors in Recognition of Bacteria and Viruses | 2.449 | EDA,EIF2AK2,IFIH1,IL1B,IRF7,OAS1,OAS2,OAS3,PTX3,RIGI,TLR7,TNFSF10,TNFSF15 |
| Systemic Lupus Erythematosus in B Cell Signaling Pathway | 3.051 | EDA,IFIH1,IFIT2,IFIT3,IGHG1,IGHG3,IGHV1-18,IGHV2-5,IGHV3-49,IGHV3-73,IGHV4-61,IGKV1-33,IGKV6-21,IGLV1-51,IGLV3-10,IGLV3-25,IL1B,IRF7,ISG15,STAT1,STAT2,TLR7,TNFSF10,TNFSF15 |
| Antimicrobial peptides | 2.449 | BPI,CAMP,LCN2,LTF,RNASE3,S100A8 |
| Neutrophil degranulation | 4.379 | ABCA13,BPI,CAMP,CEACAM6,CEACAM8,CHIT1,CLEC4D,CRISP3,FCGR3A/FCGR3B,FOLR3,HP,LCN2,LTF,MMP8,OLFM4,OLR1,PTX3,RNASE3,S100A12,S100A8,SERPINB10,TCN1,TNFAIP6 |
| DDX58/IFIH1-mediated induction of interferon-alpha/beta | 2.828 | HERC5,IFIH1,IRF7,ISG15,RIGI,S100A12,TRIM25,UBE2L6 |
| Pathogen Induced Cytokine Storm Signaling Pathway | 2.828 | AIM2,CCL3L1,CXCL10,DHX58,EDA,IFIH1,IL12RB2,IL1B,IL1RN,IRF7,RIGI,SOCS3,STAT1,STX11,TLR7,TNFSF10,TNFSF15,ZBP1 |
| Macrophage Classical Activation Signaling Pathway | 2.309 | BPI,CXCL10,EDA,GBP4,IL1B,PARP14,PARP9,SOCS3,STAT1,STAT2,TNFSF10,TNFSF15 |
| Pyroptosis Signaling Pathway | 2.121 | AIM2,CASP5,GBP1,GBP4,GBP5,IL1B,NOL3,TLR7 |
| Multiple Sclerosis Signaling Pathway | 2.887 | C2,EDA,HLA-G,IL1B,PARP12,PARP14,PARP9,RNF213,STAT1,TLR7,TNFSF10,TNFSF15 |
| Interleukin-20 family signaling | 2 | IFNLR1,SOCS3,STAT1,STAT2 |
| Necroptosis Signaling Pathway | 3 | EIF2AK2,PLA2G4A,RB1,STAT1,STAT2,TIMM10,TNFSF10,TOMM7,ZBP1 |
| Electron transport, ATP synthesis, and heat production by uncoupling proteins | 2.121 | COX6C,COX7B,COX7C,MT-ND6,NDUFB3,NDUFS5,SCO2,UQCRB |
| Role of PKR in Interferon Induction and Antiviral Response | 2.646 | CASP5,EIF2AK2,FCGR1A,IFIH1,IL1B,RIGI,STAT1,STAT2 |
| CGAS-STING Signaling Pathway | 2.828 | CD274,EDA,IDO1,IL1B,STAT1,TNFSF10,TNFSF15,ZBP1 |
| Cytoprotection by HMOX1 | 2.236 | COX6C,COX7B,COX7C,HELZ2,SCO2 |
| RHO GTPase cycle | 2.668 | ARHGAP23,ARHGEF10,ARHGEF17,ARHGEF4,CEP97,DOCK4,DOCK7,EPSTI1,FAM135A,FARP2,FMNL2,LMNB1,PDE5A,PLEKHG1,PTPN13,SOWAHC,SRGAP2 |
| Role of MAPK Signaling in Inhibiting the Pathogenesis of Influenza | 2 | CXCL10,EIF2AK2,IL1B,PLA2G4A,PNPLA4 |
| Oxidative Phosphorylation | 2.449 | COX6C,COX7B,COX7C,NDUFB3,NDUFS5,UQCRB |
| Retinoic acid Mediated Apoptosis Signaling | 2 | PARP12,PARP14,PARP9,TNFSF10 |
| Cachexia Signaling Pathway | 3.051 | CASP5,DDIT4,EDA,EIF2AK2,IL1B,IL1RN,LCN2,SOCS3,STAT1,STAT2,TLR7,TNFSF10,TNFSF15 |
| Mitochondrial protein import | 2 | CMC2,COA6,TIMM10,TOMM7 |
| ***MDD non-responders/unmedicated with CRP<1 vs. controls*** | | |
| Immunoregulatory interactions between a Lymphoid and a non-Lymphoid cell | -3.207 | HLA-G,IGHV2-5,IGHV3-11,IGKC,IGLC1,IGLV1-40,IGLV1-51,IGLV3-21,IGLV4-69,KLRC1,LILRA5,LILRA6,NECTIN2,TREML1 |
| Communication between Innate and Adaptive Immune Cells | -4.123 | HLA-G,IGHG1,IGHG3,IGHV2-5,IGHV3-11,IGHV3-49,IGKC,IGKV1D-13,IGKV3D-11,IGLC1,IGLV1-40,IGLV1-51,IGLV3-10,IGLV3-21,IGLV4-69,JCHAIN,TNFRSF17,TRAV21,TRAV38-1,TRBV6-5,TRBV6-6 |
| Binding and Uptake of Ligands by Scavenger Receptors | -3.162 | IGHV2-5,IGHV3-11,IGKC,IGLC1,IGLV1-40,IGLV1-51,IGLV3-21,IGLV4-69,JCHAIN,SCARF1 |
| OAS antiviral response | 2 | OAS1,OAS2,OAS3,OASL |
| Fcgamma receptor (FCGR) dependent phagocytosis | -3.162 | IGHG1,IGHG3,IGHV2-5,IGHV3-11,IGKC,IGLC1,IGLV1-40,IGLV1-51,IGLV3-21,IGLV4-69 |
| Cell surface interactions at the vascular wall | -2.714 | IGHV2-5,IGHV3-11,IGKC,IGLC1,IGLL1/IGLL5,IGLV1-40,IGLV1-51,IGLV3-21,IGLV4-69,JCHAIN,SDC3 |
| Role of Hypercytokinemia/hyperchemokinemia in the Pathogenesis of Influenza | 2.236 | ISG15,OAS1,OAS2,OAS3,RSAD2 |
| Fc epsilon receptor (FCERI) signaling | -2.828 | IGHV2-5,IGHV3-11,IGKC,IGLC1,IGLV1-40,IGLV1-51,IGLV3-21,IGLV4-69 |
| Signaling by the B Cell Receptor (BCR) | -2.646 | IGHV2-5,IGHV3-11,IGKC,IGLC1,IGLV1-40,IGLV1-51,IGLV3-21 |
| Dendritic Cell Maturation | -2.236 | COL18A1,HLA-G,IGHG1,IGHG3,IGKC,IGLC1,JCHAIN,TRAV21,TRAV38-1,TRBV6-5,TRBV6-6 |
| Cyclins and Cell Cycle Regulation | -2 | CCNA2,CCNB2,E2F1,PPM1J |
| Mitotic G1 phase and G1/S transition | -2.236 | CCNA2,E2F1,RRM2,TOP2A,TYMS |
| ***MDD responders with CRP<1 vs. controls*** | | |
| CTLA4 Signaling in Cytotoxic T Lymphocytes | 2.84 | AHR,AP1S3,MAPK8,PPP2CB,TRAV10,TRAV12-1,TRAV12-3,TRAV13-1,TRAV22,TRBV15,TRBV23-1,TRBV24-1,TRBV29-1,TRGC1,TRGC2 |
| Lipid Antigen Presentation by CD1 | -3.317 | TRAV10,TRAV12-1,TRAV12-3,TRAV13-1,TRAV22,TRBV15,TRBV23-1,TRBV24-1,TRBV29-1,TRGC1,TRGC2 |
| G Protein Signaling Mediated by Tubby | -2.887 | GNA11,TRAV10,TRAV12-1,TRAV12-3,TRAV13-1,TRAV22,TRBV15,TRBV23-1,TRBV24-1,TRBV29-1,TRGC1,TRGC2 |
| NFKBIE Signaling Pathway | -3.317 | TRAV10,TRAV12-1,TRAV12-3,TRAV13-1,TRAV22,TRBV15,TRBV23-1,TRBV24-1,TRBV29-1,TRGC1,TRGC2 |
| IL-4 Signaling | -3.051 | COL6A1,CREB1,TRAV10,TRAV12-1,TRAV12-3,TRAV13-1,TRAV22,TRBV15,TRBV23-1,TRBV24-1,TRBV29-1,TRGC1,TRGC2 |
| Regulation of IL-2 Expression in Activated and Anergic T Lymphocytes | -2.53 | MAPK8,TRAV10,TRAV12-1,TRAV12-3,TRAV13-1,TRAV22,TRBV15,TRBV23-1,TRBV24-1,TRBV29-1 |
| T Cell Receptor Signaling | -2.53 | MAPK8,TRAV10,TRAV12-1,TRAV12-3,TRAV13-1,TRAV22,TRBV15,TRBV23-1,TRBV24-1,TRBV29-1,TRGC1,TRGC2 |
| Chaperone Mediated Autophagy Signaling Pathway | -3.317 | TRAV10,TRAV12-1,TRAV12-3,TRAV13-1,TRAV22,TRBV15,TRBV23-1,TRBV24-1,TRBV29-1,TRGC1,TRGC2 |
| Oxytocin Signaling Pathway | 2.121 | CACNA1F,CACNG6,CCL5,CREB1,GNA11,MAPK8,PPARA,PRKG2 |
| Platelet homeostasis | 2 | KCNMB1,PPP2CB,PRKG2,SLC8A1 |
| \| ***MDD responders with CRP>1 vs. non-responders/unmedicated with CRP>1*** \| \| --- \| | | |
| Binding and Uptake of Ligands by Scavenger Receptors | -4.796 | IGHV1-2,IGHV2-5,IGHV3-48,IGHV3-53,IGHV4-39,IGHV4-59,IGKV1-33,IGKV3-15,IGKV3-20,IGKV4-1,IGLC2,IGLC3,IGLV1-36,IGLV2-11,IGLV2-14,IGLV2-23,IGLV2-8,IGLV3-19,IGLV3-25,IGLV3-27,IGLV4-69,IGLV7-43,IGLV8-61 |
| Immunoregulatory interactions between a Lymphoid and a non-Lymphoid cell | -4.426 | CD200,IGHV1-2,IGHV2-5,IGHV3-48,IGHV3-53,IGHV4-39,IGHV4-59,IGKV1-33,IGKV3-15,IGKV3-20,IGKV4-1,IGLC2,IGLC3,IGLV1-36,IGLV2-11,IGLV2-14,IGLV2-23,IGLV2-8,IGLV3-19,IGLV3-25,IGLV3-27,IGLV4-69,IGLV7-43,IGLV8-61,KIR3DL2,KLRC1,TREML1 |
| Cell surface interactions at the vascular wall | -4.811 | CEACAM1,ESAM,GP6,IGHV1-2,IGHV2-5,IGHV3-48,IGHV3-53,IGHV4-39,IGHV4-59,IGKV1-33,IGKV3-15,IGKV3-20,IGKV4-1,IGLC2,IGLC3,IGLV1-36,IGLV2-11,IGLV2-14,IGLV2-23,IGLV2-8,IGLV3-19,IGLV3-25,IGLV3-27,IGLV4-69,IGLV7-43,IGLV8-61,VPREB3 |
| Communication between Innate and Adaptive Immune Cells | -5.048 | CXCL10,IGHV1-18,IGHV1-2,IGHV2-26,IGHV2-5,IGHV3-48,IGHV3-53,IGHV3-73,IGHV4-39,IGHV4-59,IGHV4-61,IGHV5-51,IGKV1-33,IGKV2-24,IGKV3-15,IGKV3-20,IGKV4-1,IGLC2,IGLC3,IGLV1-36,IGLV2-11,IGLV2-14,IGLV2-23,IGLV2-8,IGLV3-10,IGLV3-19,IGLV3-25,IGLV3-27,IGLV3-9,IGLV4-69,IGLV7-43,IGLV8-61,IL1RN,TRAV1-1,TRAV8-6,TRBV7-3 |
| Fcgamma receptor (FCGR) dependent phagocytosis | -4.796 | IGHV1-2,IGHV2-5,IGHV3-48,IGHV3-53,IGHV4-39,IGHV4-59,IGKV1-33,IGKV3-15,IGKV3-20,IGKV4-1,IGLC2,IGLC3,IGLV1-36,IGLV2-11,IGLV2-14,IGLV2-23,IGLV2-8,IGLV3-19,IGLV3-25,IGLV3-27,IGLV4-69,IGLV7-43,IGLV8-61 |
| Fc epsilon receptor (FCERI) signaling | -4.796 | IGHV1-2,IGHV2-5,IGHV3-48,IGHV3-53,IGHV4-39,IGHV4-59,IGKV1-33,IGKV3-15,IGKV3-20,IGKV4-1,IGLC2,IGLC3,IGLV1-36,IGLV2-11,IGLV2-14,IGLV2-23,IGLV2-8,IGLV3-19,IGLV3-25,IGLV3-27,IGLV4-69,IGLV7-43,IGLV8-61 |
| Signaling by the B Cell Receptor (BCR) | -4.472 | IGHV1-2,IGHV2-5,IGHV3-48,IGHV3-53,IGHV4-39,IGHV4-59,IGKV1-33,IGKV3-15,IGKV3-20,IGKV4-1,IGLC2,IGLC3,IGLV2-11,IGLV2-14,IGLV2-23,IGLV2-8,IGLV3-19,IGLV3-25,IGLV3-27,IGLV7-43 |
| Role of Hypercytokinemia/hyperchemokinemia in the Pathogenesis of Influenza | 2 | CXCL10,IL1RN,STAT1,STAT2 |
| Interferon gamma signaling | 2 | GBP1,GBP4,MT2A,STAT1 |
| ***MDD non-responders/unmedicated with CRP<1 vs. non-responders/unmedicated with CRP>1*** | | |
| Eukaryotic Translation Elongation | -6.557 | EEF1B2,FAU,RPL11,RPL17,RPL21,RPL22,RPL22L1,RPL23,RPL24,RPL26,RPL26L1,RPL27,RPL30,RPL31,RPL32,RPL34,RPL35,RPL35A,RPL36A,RPL37,RPL39,RPL39L,RPL41,RPL7,RPL9,RPS10,RPS12,RPS14,RPS15A,RPS17,RPS18,RPS20,RPS21,RPS23,RPS24,RPS25,RPS27,RPS27A,RPS27L,RPS29,RPS3A,RPS6,RPS7 |
| Response of EIF2AK4 (GCN2) to amino acid deficiency | -6.633 | DDIT3,EIF2S1,FAU,RPL11,RPL17,RPL21,RPL22,RPL22L1,RPL23,RPL24,RPL26,RPL26L1,RPL27,RPL30,RPL31,RPL32,RPL34,RPL35,RPL35A,RPL36A,RPL37,RPL39,RPL39L,RPL41,RPL7,RPL9,RPS10,RPS12,RPS14,RPS15A,RPS17,RPS18,RPS20,RPS21,RPS23,RPS24,RPS25,RPS27,RPS27A,RPS27L,RPS29,RPS3A,RPS6,RPS7 |
| Eukaryotic Translation Termination | -6.481 | FAU,RPL11,RPL17,RPL21,RPL22,RPL22L1,RPL23,RPL24,RPL26,RPL26L1,RPL27,RPL30,RPL31,RPL32,RPL34,RPL35,RPL35A,RPL36A,RPL37,RPL39,RPL39L,RPL41,RPL7,RPL9,RPS10,RPS12,RPS14,RPS15A,RPS17,RPS18,RPS20,RPS21,RPS23,RPS24,RPS25,RPS27,RPS27A,RPS27L,RPS29,RPS3A,RPS6,RPS7 |
| SRP-dependent cotranslational protein targeting to membrane | -6.708 | FAU,RPL11,RPL17,RPL21,RPL22,RPL22L1,RPL23,RPL24,RPL26,RPL26L1,RPL27,RPL30,RPL31,RPL32,RPL34,RPL35,RPL35A,RPL36A,RPL37,RPL39,RPL39L,RPL41,RPL7,RPL9,RPS10,RPS12,RPS14,RPS15A,RPS17,RPS18,RPS20,RPS21,RPS23,RPS24,RPS25,RPS27,RPS27A,RPS27L,RPS29,RPS3A,RPS6,RPS7,SEC11C,SRP14,SSR3 |
| Selenoamino acid metabolism | -6.557 | FAU,HNMT,RPL11,RPL17,RPL21,RPL22,RPL22L1,RPL23,RPL24,RPL26,RPL26L1,RPL27,RPL30,RPL31,RPL32,RPL34,RPL35,RPL35A,RPL36A,RPL37,RPL39,RPL39L,RPL41,RPL7,RPL9,RPS10,RPS12,RPS14,RPS15A,RPS17,RPS18,RPS20,RPS21,RPS23,RPS24,RPS25,RPS27,RPS27A,RPS27L,RPS29,RPS3A,RPS6,RPS7 |
| Eukaryotic Translation Initiation | -6.557 | EIF2S1,FAU,RPL11,RPL17,RPL21,RPL22,RPL22L1,RPL23,RPL24,RPL26,RPL26L1,RPL27,RPL30,RPL31,RPL32,RPL34,RPL35,RPL35A,RPL36A,RPL37,RPL39,RPL39L,RPL41,RPL7,RPL9,RPS10,RPS12,RPS14,RPS15A,RPS17,RPS18,RPS20,RPS21,RPS23,RPS24,RPS25,RPS27,RPS27A,RPS27L,RPS29,RPS3A,RPS6,RPS7 |
| Nonsense-Mediated Decay (NMD) | -6.481 | FAU,RPL11,RPL17,RPL21,RPL22,RPL22L1,RPL23,RPL24,RPL26,RPL26L1,RPL27,RPL30,RPL31,RPL32,RPL34,RPL35,RPL35A,RPL36A,RPL37,RPL39,RPL39L,RPL41,RPL7,RPL9,RPS10,RPS12,RPS14,RPS15A,RPS17,RPS18,RPS20,RPS21,RPS23,RPS24,RPS25,RPS27,RPS27A,RPS27L,RPS29,RPS3A,RPS6,RPS7 |
| Major pathway of rRNA processing in the nucleolus and cytosol | -6.557 | FAU,KRR1,RPL11,RPL17,RPL21,RPL22,RPL22L1,RPL23,RPL24,RPL26,RPL26L1,RPL27,RPL30,RPL31,RPL32,RPL34,RPL35,RPL35A,RPL36A,RPL37,RPL39,RPL39L,RPL41,RPL7,RPL9,RPS10,RPS12,RPS14,RPS15A,RPS17,RPS18,RPS20,RPS21,RPS23,RPS24,RPS25,RPS27,RPS27A,RPS27L,RPS29,RPS3A,RPS6,RPS7 |
| EIF2 Signaling | -4.6 | DDIT3,EIF2S1,FAU,RASD1,RPL11,RPL17,RPL21,RPL22,RPL22L1,RPL23,RPL24,RPL26,RPL26L1,RPL27,RPL30,RPL31,RPL32,RPL34,RPL35,RPL35A,RPL36A,RPL37,RPL39,RPL39L,RPL41,RPL7,RPL9,RPS10,RPS12,RPS14,RPS15A,RPS17,RPS18,RPS20,RPS21,RPS23,RPS24,RPS25,RPS27,RPS27A,RPS27L,RPS29,RPS3A,RPS6,RPS7 |
| Electron transport, ATP synthesis, and heat production by uncoupling proteins | -2.694 | ATP5F1C,ATP5F1E,ATP5ME,ATP5MG,ATP5PF,ATP5PO,COX14,COX16,COX6C,COX7B,COX7C,MT-ATP6,MT-CO1,MT-CO2,MT-CYB,MT-ND1,MT-ND2,MT-ND4,MT-ND5,NDUFA1,NDUFA4,NDUFAF2,NDUFB1,NDUFB3,NDUFS4,NDUFS5,TMEM126B,UQCR11,UQCRB,UQCRH,UQCRQ |
| Oxidative Phosphorylation | -2.268 | ATP5F1C,ATP5F1E,ATP5ME,ATP5MG,ATP5PF,ATP5PO,COX6C,COX7A2,COX7B,COX7C,MT-ATP6,MT-CO1,MT-CO2,MT-CYB,MT-ND1,MT-ND2,MT-ND4,MT-ND5,NDUFA1,NDUFA4,NDUFB1,NDUFB3,NDUFS4,NDUFS5,UQCR11,UQCRB,UQCRH,UQCRQ |
| Neutrophil degranulation | -7.211 | ABCA13,ADGRG3,ARG1,ATP6V1D,B2M,BST1,CAMP,CD177,CD36,CD58,CD59,CEACAM3,CLEC4D,COMMD3,CR1,CRISP3,CYSTM1,FCAR,FCER1G,FCGR2A,FCGR3A/FCGR3B,FPR2,GCA,GMFG,GPR84,HP,HPSE,HSP90AA1,ITGAX,MCEMP1,MME,MMP8,OLFM4,OLR1,ORM1,P2RX1,PGLYRP1,PLAU,PPBP,PSMA2,PYGL,QPCT,RAB18,S100A12,S100A8,S100A9,SLC11A1,SLPI,SRP14,SVIP,TNFAIP6,TXNDC5 |
| Binding and Uptake of Ligands by Scavenger Receptors | -4.583 | CD36,HP,HSP90AA1,IGHV1-46,IGHV2-5,IGHV3-33,IGHV3-53,IGHV4-39,IGKV1-5,IGKV1D-39,IGKV2-30,IGKV4-1,IGLV1-40,IGLV10-54,IGLV2-23,IGLV3-19,IGLV4-69,IGLV5-45,IGLV7-43,JCHAIN,SCARF1 |
| Mitochondrial Dysfunction | 2.466 | ATP5F1C,ATP5F1E,ATP5ME,ATP5MG,ATP5PF,ATP5PO,C9orf72,CACNA1E,CACNB4,CAPN5,COX6C,COX7A2,COX7B,COX7C,CREB5,DHODH,GPX3,MT-ATP6,MT-CO1,MT-CO2,MT-CYB,MT-ND1,MT-ND2,MT-ND4,MT-ND5,NDUFA1,NDUFA4,NDUFB1,NDUFB3,NDUFS4,NDUFS5,PRKAG1,TOMM7,UQCR11,UQCRB,UQCRH,UQCRQ |
| Immunoregulatory interactions between a Lymphoid and a non-Lymphoid cell | -3.78 | B2M,CLEC2B,FCGR1A,FCGR3A/FCGR3B,IGHV1-46,IGHV2-5,IGHV3-33,IGHV3-53,IGHV4-39,IGKV1-5,IGKV1D-39,IGKV2-30,IGKV4-1,IGLV1-40,IGLV10-54,IGLV2-23,IGLV3-19,IGLV4-69,IGLV5-45,IGLV7-43,KIR2DL1/KIR2DL3,KIR3DL1,KIR3DL2,KLRB1,LILRA5,LILRA6,TRAV29DV5,TREML1 |
| Complement cascade | -2.449 | C1QA,C1QB,CD59,CLU,CR1,IGHV1-46,IGHV2-5,IGHV3-33,IGHV3-53,IGHV4-39,IGKV1-5,IGKV1D-39,IGKV2-30,IGKV4-1,IGLV1-40,IGLV10-54,IGLV2-23,IGLV3-19,IGLV4-69,IGLV5-45,IGLV7-43,PROS1 |
| Cell surface interactions at the vascular wall | -4.426 | CD177,CD58,CEACAM3,FCER1G,IGHV1-46,IGHV2-5,IGHV3-33,IGHV3-53,IGHV4-39,IGKV1-5,IGKV1D-39,IGKV2-30,IGKV4-1,IGLV1-40,IGLV10-54,IGLV2-23,IGLV3-19,IGLV4-69,IGLV5-45,IGLV7-43,ITGAX,ITGB3,JCHAIN,OLR1,PROS1,SDC3,YES1 |
| Fcgamma receptor (FCGR) dependent phagocytosis | -3.838 | BAIAP2,FCGR1A,FCGR2A,FCGR3A/FCGR3B,HSP90AA1,IGHV1-46,IGHV2-5,IGHV3-33,IGHV3-53,IGHV4-39,IGKV1-5,IGKV1D-39,IGKV2-30,IGKV4-1,IGLV1-40,IGLV10-54,IGLV2-23,IGLV3-19,IGLV4-69,IGLV5-45,IGLV7-43,YES1 |
| Coronavirus Pathogenesis Pathway | 3.962 | CASP1,DDIT3,E2F1,FAU,RPS10,RPS12,RPS14,RPS15A,RPS17,RPS18,RPS20,RPS21,RPS23,RPS24,RPS25,RPS27,RPS27A,RPS27L,RPS29,RPS3A,RPS6,RPS7,TRIM25 |
| Fc epsilon receptor (FCERI) signaling | -4.583 | FCER1G,IGHV1-46,IGHV2-5,IGHV3-33,IGHV3-53,IGHV4-39,IGKV1-5,IGKV1D-39,IGKV2-30,IGKV4-1,IGLV1-40,IGLV10-54,IGLV2-23,IGLV3-19,IGLV4-69,IGLV5-45,IGLV7-43,PSMA2,PSMA3,PSMA6,RPS27A,SEM1 |
| Phospholipase C Signaling | -2.236 | CREB5,FCER1G,FCGR2A,GNG10,GNG11,GPLD1,GUCY1A1,IGHV1-46,IGHV2-5,IGHV3-33,IGHV3-53,IGHV4-39,IGHV4-61,IGHV6-1,IGKV1-5,IGKV1D-13,IGKV1D-39,IGKV2-24,IGKV2-30,IGKV4-1,IGLV1-40,IGLV10-54,IGLV2-23,IGLV3-19,IGLV3-9,IGLV4-69,IGLV5-45,IGLV7-43,ITGA2B,ITGA7,ITGAX,ITGB3,ITGB4,ITGB8,JCHAIN,MYL6,MYL9,PLD4,RASD1,RHOBTB1,TRAV21,TRAV24,TRAV29DV5,TRAV38-1,TRBV14,TRBV6-6 |
| Signaling by the B Cell Receptor (BCR) | -4.243 | IGHV1-46,IGHV2-5,IGHV3-33,IGHV3-53,IGHV4-39,IGKV1-5,IGKV1D-39,IGKV2-30,IGKV4-1,IGLV1-40,IGLV2-23,IGLV3-19,IGLV7-43,PSMA2,PSMA3,PSMA6,RPS27A,SEM1 |
| Communication between Innate and Adaptive Immune Cells | -5.196 | B2M,FCER1G,IGHV1-46,IGHV2-5,IGHV3-33,IGHV3-53,IGHV4-39,IGHV4-61,IGHV6-1,IGKV1-5,IGKV1D-13,IGKV1D-39,IGKV2-24,IGKV2-30,IGKV4-1,IGLV1-40,IGLV10-54,IGLV2-23,IGLV3-19,IGLV3-9,IGLV4-69,IGLV5-45,IGLV7-43,JCHAIN,TLR5,TLR6,TNFRSF17,TNFSF13B,TRAV21,TRAV24,TRAV29DV5,TRAV38-1,TRBV14,TRBV6-6 |
| Phagosome Formation | -3.086 | ADGRG3,CCR3,CCR9,CD209,CD36,CLEC4D,CMKLR1,CR1,FCAR,FCER1G,FCGR1A,FCGR2A,FCGR3A/FCGR3B,FFAR3,FPR2,FPR3,GIPR,GPER1,GPLD1,GPR153,GPR171,GPR84,HCAR2,HCAR3,ITGA2B,ITGA7,ITGAX,ITGB3,ITGB4,ITGB8,JCHAIN,LIMK2,LPAR6,MYL6,MYL9,P2RY14,PLD4,PNPLA4,RASD1,TLR5,TLR6,UTS2R,XCR1,YES1 |
| TP53 Regulates Metabolic Genes | -2.309 | COX14,COX16,COX6C,COX7B,COX7C,MT-CO1,MT-CO2,NDUFA4,PRDX1,PRKAG1,SESN3,TXN |
| Inflammasome pathway | -2.236 | AIM2,CASP1,CASP5,NAIP,NOD2 |
| Regulation of TLR by endogenous ligand | -2.236 | CD36,LY96,S100A8,S100A9,TLR6 |
| Pyroptosis Signaling Pathway | -2.53 | AIM2,CASP1,CASP4,CASP5,GAS5,GBP5,NAIP,PRKAG1,TLR5,TLR6 |
| Role of NFAT in Regulation of the Immune Response | -2 | FCER1G,FCGR1A,FCGR2A,FCGR3A/FCGR3B,GNG10,GNG11,IGHV1-46,IGHV2-5,IGHV3-33,IGHV3-53,IGHV4-39,IGHV4-61,IGHV6-1,IGKV1-5,IGKV1D-13,IGKV1D-39,IGKV2-24,IGKV2-30,IGKV4-1,IGLV1-40,IGLV10-54,IGLV2-23,IGLV3-19,IGLV3-9,IGLV4-69,IGLV5-45,IGLV7-43,RASD1,TRAV21,TRAV24,TRAV29DV5,TRAV38-1,TRBV14,TRBV6-6 |
| Caspase activation via Death Receptors in the presence of ligand | -2 | CFLAR,FAS,LY96,TNFSF10 |
| Response to elevated platelet cytosolic Ca2+ | -3.464 | CD36,CLU,EGF,F5,ITGA2B,ITGB3,LY6G6F,MMRN1,ORM1,PPBP,PROS1,SRGN |
| Synthesis of DNA | -3.317 | ANAPC10,CDC6,POLE4,PSMA2,PSMA3,PSMA6,RBX1,RPA3,RPS27A,SEM1,UBE2C |
| MyD88:MAL(TIRAP) cascade initiated on plasma membrane | -2.449 | CD36,LY96,RPS27A,S100A8,S100A9,TLR6 |
| Regulation of mitotic cell cycle | -3 | ANAPC10,CCNB1,CDC20,PSMA2,PSMA3,PSMA6,RPS27A,SEM1,UBE2C |
| RIPK1-mediated regulated necrosis | -2.236 | CFLAR,FAS,HSP90AA1,RPS27A,TNFSF10 |
| Class I MHC mediated antigen processing and presentation | -3.545 | ANAPC10,B2M,CD36,CDC20,FBXL13,FCGR1A,LMO7,LY96,PSMA2,PSMA3,PSMA6,RBX1,RPS27A,S100A8,S100A9,SAR1B,SEM1,SOCS3,TLR6,TRAIP,UBE2C,UBE2J1,UBE3D |
| Mitotic G1 phase and G1/S transition | -3.162 | CCNB1,CDC6,CDKN2B,E2F1,PSMA2,PSMA3,PSMA6,RPS27A,SEM1,TOP2A,TYMS |
| Multiple Sclerosis Signaling Pathway | -2.84 | C1QA,C1QB,CAPN5,CASP1,EDA,FAS,GAS5,OSM,PARP9,PLAU,SLC8A1,TLR5,TLR6,TNFSF10,TNFSF13B |
| S100 Family Signaling Pathway | -2.722 | ADGRG3,ANXA1,ATP5F1C,ATP5F1E,ATP5PO,CACNA1E,CACNB4,CCR3,CCR9,CD36,CMKLR1,CREB5,EGF,ERBB2,FCGR1A,FCGR2A,FCGR3A/FCGR3B,FFAR3,FPR2,FPR3,GIPR,GPER1,GPR153,GPR171,GPR84,HCAR2,HCAR3,JCHAIN,LPAR6,MMP15,MMP23B,MMP8,P2RY14,PRKAG1,S100A12,S100A8,S100A9,UTS2R,XCR1 |
| Molecular Mechanisms of Cancer | -2.16 | ADGRG3,CCR3,CCR9,CDKN2B,CFLAR,CMKLR1,E2F1,EGF,FAS,FFAR3,FPR2,FPR3,GIPR,GNG10,GNG11,GPER1,GPR153,GPR171,GPR84,GUCY1A1,HCAR2,HCAR3,IL3RA,IL4R,ITGA2B,ITGA7,ITGAX,ITGB3,ITGB4,ITGB8,LPAR6,LRP6,MMP15,MMP23B,MMP8,NAIP,P2RY14,PRKAG1,RASD1,RHOBTB1,UTS2R,XCR1 |
| Formation of Fibrin Clot (Clotting Cascade) | -2.236 | CD177,F5,GP1BB,GP9,PROS1 |
| NLR signaling pathways | -2.449 | AIM2,CASP1,CASP4,NOD2,RPS27A,TXN |
| Cell Cycle Checkpoints | -3.638 | ANAPC10,BIRC5,CCNB1,CCNB2,CDC20,CDC6,KIF2C,MDC1,PSMA2,PSMA3,PSMA6,RAD9B,RPA3,RPS27,RPS27A,SEM1,UBE2C |
| Interleukin-4 and Interleukin-13 signaling | -3 | ANXA1,BCL6,BIRC5,CD36,HSP90AA1,IL4R,ITGAX,OSM,SOCS3 |
| Pathogen Induced Cytokine Storm Signaling Pathway | -2.236 | AIM2,CASP1,CCL3L1,CCR3,CKLF,COL13A1,COL6A2,EDA,GZMB,NOD2,OSM,PPBP,PRF1,SLC2A1,SOCS3,SRGN,TLR5,TLR6,TNFSF10,TNFSF13B |
| Smooth Muscle Contraction | -2.236 | ANXA1,DYSF,GUCY1A1,MYL6,MYL9 |
| Mitotic Metaphase and Anaphase | -3.357 | ANAPC10,BIRC5,CCNB1,CCNB2,CDC20,KIF2C,LMNB1,PSMA2,PSMA3,PSMA6,RPS27,RPS27A,SEM1,TUBB4A,UBE2C |
| TP53 Regulates Transcription of Cell Death Genes | -2.236 | BCL6,BIRC5,CASP1,FAS,TRIAP1 |
| Mitochondrial translation | -2.828 | CHCHD1,MRPL13,MRPL14,MRPL15,MRPL22,MRPL47,MRPL51,MRPS18C |
| Mitotic G2-G2/M phases | -3.051 | CCNB1,CCNB2,CEP63,E2F1,FOXM1,HSP90AA1,PSMA2,PSMA3,PSMA6,RBX1,RPS27A,SEM1,TUBB4A |
| Mitotic Roles of Polo-Like Kinase | -2 | ANAPC10,CCNB1,CCNB2,CDC20,HSP90AA1,PPM1J |
| DNA Damage Bypass | -2.236 | POLE4,RBX1,RPA3,RPS27A,TRIM25 |
| DNA Replication Pre-Initiation | -2.828 | ANAPC10,CDC6,PSMA2,PSMA3,PSMA6,RPS27A,SEM1,UBE2C |
| TNFR2 non-canonical NF-kB pathway | -2.449 | PSMA2,PSMA3,PSMA6,RPS27A,SEM1,TNFSF13B |
| Regulation of Apoptosis | -2.236 | PSMA2,PSMA3,PSMA6,RPS27A,SEM1 |
| TREM1 Signaling | -2.449 | CASP1,CASP5,ITGAX,NOD2,TLR5,TLR6 |
| G alpha (q) signalling events | -2.111 | ANXA1,FFAR3,FPR2,GNG10,GNG11,GNRH1,LPAR6,PROK2,RGS16,UTS2R,XCR1 |
| Regulation of RUNX2 expression and activity | -2.449 | PSMA2,PSMA3,PSMA6,RBX1,RPS27A,SEM1 |
| Metabolism of non-coding RNA | -2.236 | GEMIN6,SNRPD1,SNRPD2,SNRPE,SNRPG |
| Cellular response to hypoxia | -2.449 | PSMA2,PSMA3,PSMA6,RBX1,RPS27A,SEM1 |
| Neddylation | -3.207 | ANKRD9,BIRC5,COMMD3,COMMD6,COPS2,FBXL13,LMO7,PSMA2,PSMA3,PSMA6,RBX1,RPS27A,SEM1,SOCS3 |
| Protein Ubiquitination Pathway | -3.207 | ANAPC10,B2M,CDC20,DNAJA1,DNAJC15,HSP90AA1,HSPA12A,PSMA2,PSMA3,PSMA6,RBX1,RPS27A,UBE2C,UBE2J1,UBE2T |
| Ephrin Receptor Signaling | -2 | CREB5,EGF,GNG10,GNG11,ITGA2B,ITGA7,ITGAX,ITGB3,ITGB4,ITGB8,LIMK2,RASD1 |
| \| ***MDD responders with CRP<1 vs. non-responders/unmedicated with CRP>1*** \| \| --- \| | | |
| Interferon alpha/beta signaling | -3.051 | EGR1,IFI6,IFIT1,IFIT3,IFIT5,IFITM1,IFITM3,ISG15,MX1,OASL,RSAD2,SOCS1,SOCS3 |
| Eukaryotic Translation Termination | -3.742 | RPL12,RPL21,RPL22,RPL26L1,RPL30,RPL32,RPL36A,RPL37,RPL39,RPL41,RPS23,RPS25,RPS27,RPS29 |
| Eukaryotic Translation Elongation | -3.742 | RPL12,RPL21,RPL22,RPL26L1,RPL30,RPL32,RPL36A,RPL37,RPL39,RPL41,RPS23,RPS25,RPS27,RPS29 |
| Response of EIF2AK4 (GCN2) to amino acid deficiency | -3.742 | RPL12,RPL21,RPL22,RPL26L1,RPL30,RPL32,RPL36A,RPL37,RPL39,RPL41,RPS23,RPS25,RPS27,RPS29 |
| Selenoamino acid metabolism | -3.742 | RPL12,RPL21,RPL22,RPL26L1,RPL30,RPL32,RPL36A,RPL37,RPL39,RPL41,RPS23,RPS25,RPS27,RPS29 |
| Neutrophil degranulation | -5.014 | ABCA13,AOC1,AZU1,BPI,CAMP,CD177,CEACAM6,CEACAM8,CRISP3,CYSTM1,DEFA1 (includes others),DEFA4,FCER1G,HP,LCN2,LTF,MCEMP1,MMP8,MPO,OLFM4,OLR1,RETN,RNASE3,RNASET2,S100A12,S100A8,S100A9,SLC2A5,TNFAIP6 |
| SRP-dependent cotranslational protein targeting to membrane | -3.742 | RPL12,RPL21,RPL22,RPL26L1,RPL30,RPL32,RPL36A,RPL37,RPL39,RPL41,RPS23,RPS25,RPS27,RPS29 |
| Nonsense-Mediated Decay (NMD) | -3.742 | RPL12,RPL21,RPL22,RPL26L1,RPL30,RPL32,RPL36A,RPL37,RPL39,RPL41,RPS23,RPS25,RPS27,RPS29 |
| Eukaryotic Translation Initiation | -3.742 | RPL12,RPL21,RPL22,RPL26L1,RPL30,RPL32,RPL36A,RPL37,RPL39,RPL41,RPS23,RPS25,RPS27,RPS29 |
| Interferon Signaling | -2.121 | IFI6,IFIT1,IFIT3,IFITM1,IFITM3,ISG15,MX1,SOCS1 |
| EIF2 Signaling | -3.317 | AGO3,PIK3R3,RPL12,RPL21,RPL22,RPL26L1,RPL30,RPL32,RPL36A,RPL37,RPL39,RPL41,RPS23,RPS25,RPS27,RPS29,VEGFA |
| Antimicrobial peptides | -2.646 | BPI,CAMP,LCN2,LTF,RNASE3,S100A8,S100A9 |
| Major pathway of rRNA processing in the nucleolus and cytosol | -3.742 | RPL12,RPL21,RPL22,RPL26L1,RPL30,RPL32,RPL36A,RPL37,RPL39,RPL41,RPS23,RPS25,RPS27,RPS29 |
| Communication between Innate and Adaptive Immune Cells | -2.53 | CCL5,CD3D,FCER1G,IGHV3-13,IGHV3-64D,IGHV4-34,IGHV4-61,IGHV7-4-1,IGLV2-18,IGLV2-23,IGLV3-19,IGLV3-9,TRAV17,TRAV8-1,TRBV24-1,TRBV25-1,TRBV27,TRBV7-2,TRBV7-3,TRGC2,TRGV8 |
| Cell surface interactions at the vascular wall | -3.464 | CD177,CEACAM6,CEACAM8,FCER1G,IGHV3-13,IGHV4-34,IGLV2-18,IGLV2-23,IGLV3-19,OLR1,PROS1,SLC7A8 |
| G Protein Signaling Mediated by Tubby | -2.887 | CD3D,FCER1G,GNAL,TRAV17,TRAV8-1,TRBV24-1,TRBV25-1,TRBV27,TRBV7-2,TRBV7-3,TRGC2,TRGV8,TTC21B |
| Mitotic Metaphase and Anaphase | -3.464 | ANAPC10,BIRC5,CCNB2,CDC20,CDCA5,CENPM,CENPN,LMNB1,RPS27,SEM1,TUBB2A,UBE2C |
| IL-4 Signaling | -2.5 | BATF,CD3D,CREB1,CREBBP,FCER1G,PIK3R3,SOCS1,TRAV17,TRAV8-1,TRBV24-1,TRBV25-1,TRBV27,TRBV7-2,TRBV7-3,TRGC2,TRGV8 |
| Cell Cycle Checkpoints | -3.051 | ANAPC10,BARD1,BIRC5,CCNB2,CDC20,CENPM,CENPN,ORC1,PKMYT1,RAD9B,RPS27,SEM1,UBE2C |
| Lipid Antigen Presentation by CD1 | -3.317 | CD3D,FCER1G,TRAV17,TRAV8-1,TRBV24-1,TRBV25-1,TRBV27,TRBV7-2,TRBV7-3,TRGC2,TRGV8 |
| FAK Signaling | -2.117 | ADGRA3,ADGRB2,CCR1,CD3D,ERBB2,FCER1G,FFAR2,FFAR3,FZD2,GPR15,GPR171,GPRC5B,IL2RA,ITGB8,PIK3R3,SHB,SOCS1,SOCS3,TRAV17,TRAV8-1,TRBV24-1,TRBV25-1,TRBV27,TRBV7-2,TRBV7-3,TRGC2,TRGV8 |
| Role of Hypercytokinemia/hyperchemokinemia in the Pathogenesis of Influenza | -2.449 | CCL5,IFIT3,ISG15,MX1,RIGI,RSAD2 |
| Class I MHC mediated antigen processing and presentation | -3.357 | ANAPC10,CDC20,FBXL8,FBXO2,FCGR1A,HERC5,LY96,S100A8,S100A9,SEM1,SH3RF1,SOCS1,SOCS3,TRIM9,UBE2C |
| NFKBIE Signaling Pathway | -3.317 | CD3D,FCER1G,TRAV17,TRAV8-1,TRBV24-1,TRBV25-1,TRBV27,TRBV7-2,TRBV7-3,TRGC2,TRGV8 |
| MyD88:MAL(TIRAP) cascade initiated on plasma membrane | -2 | LY96,S100A8,S100A9,SOCS1 |
| ISG15 antiviral mechanism | -2.236 | HERC5,IFIT1,ISG15,MX1,RIGI |
| Chaperone Mediated Autophagy Signaling Pathway | -2.324 | CD3D,CREBBP,FCER1G,MMP17,MMP8,PIK3R3,TRAV17,TRAV8-1,TRBV24-1,TRBV25-1,TRBV27,TRBV7-2,TRBV7-3,TRGC2,TRGV8 |
| Mitotic Prometaphase | -3 | BIRC5,CCNB2,CDC20,CDCA5,CENPM,CENPN,MZT2A,RPS27,TUBB2A |
| Binding and Uptake of Ligands by Scavenger Receptors | -2.449 | HP,IGHV3-13,IGHV4-34,IGLV2-18,IGLV2-23,IGLV3-19 |
| CTLA4 Signaling in Cytotoxic T Lymphocytes | 3.606 | AP1M2,CD3D,FCER1G,PIK3R3,TRAV17,TRAV8-1,TRBV24-1,TRBV25-1,TRBV27,TRBV7-2,TRBV7-3,TRGC2,TRGV8 |
| T Cell Receptor Signaling | -3.317 | CD3D,DUSP6,FCER1G,PIK3R3,TRAV17,TRAV8-1,TRBV24-1,TRBV25-1,TRBV27,TRBV7-2,TRBV7-3,TRGC2,TRGV8 |
| Electron transport, ATP synthesis, and heat production by uncoupling proteins | -2.449 | ATP5ME,ATP5MG,COX16,COX7C,NDUFAF4,SCO2 |
| Mitotic G1 phase and G1/S transition | -2.449 | CDT1,MYBL2,ORC1,SEM1,TOP2A,TYMS |
| Fc epsilon receptor (FCERI) signaling | -2.121 | FCER1G,IGHV3-13,IGHV4-34,IGLV2-18,IGLV2-23,IGLV3-19,MS4A2,SEM1 |
| DNA Replication Pre-Initiation | -2.236 | ANAPC10,CDT1,ORC1,SEM1,UBE2C |
| ***MDD non-responders/unmedicated with CRP<1 vs. responders with CRP>1*** | | |
| Eukaryotic Translation Elongation | -4.6 | EEF1B2,RPL17,RPL21,RPL23,RPL26,RPL26L1,RPL27,RPL31,RPL34,RPL35A,RPL36A,RPL39,RPL41,RPS10,RPS15A,RPS17,RPS18,RPS21,RPS24,RPS27,RPS27L,RPS28,RPS29,RPS3A,RPS7 |
| Eukaryotic Translation Termination | -4.491 | RPL17,RPL21,RPL23,RPL26,RPL26L1,RPL27,RPL31,RPL34,RPL35A,RPL36A,RPL39,RPL41,RPS10,RPS15A,RPS17,RPS18,RPS21,RPS24,RPS27,RPS27L,RPS28,RPS29,RPS3A,RPS7 |
| Response of EIF2AK4 (GCN2) to amino acid deficiency | -4.6 | DDIT3,RPL17,RPL21,RPL23,RPL26,RPL26L1,RPL27,RPL31,RPL34,RPL35A,RPL36A,RPL39,RPL41,RPS10,RPS15A,RPS17,RPS18,RPS21,RPS24,RPS27,RPS27L,RPS28,RPS29,RPS3A,RPS7 |
| SRP-dependent cotranslational protein targeting to membrane | -4.6 | RPL17,RPL21,RPL23,RPL26,RPL26L1,RPL27,RPL31,RPL34,RPL35A,RPL36A,RPL39,RPL41,RPS10,RPS15A,RPS17,RPS18,RPS21,RPS24,RPS27,RPS27L,RPS28,RPS29,RPS3A,RPS7,SEC11C |
| Selenoamino acid metabolism | -4.491 | RPL17,RPL21,RPL23,RPL26,RPL26L1,RPL27,RPL31,RPL34,RPL35A,RPL36A,RPL39,RPL41,RPS10,RPS15A,RPS17,RPS18,RPS21,RPS24,RPS27,RPS27L,RPS28,RPS29,RPS3A,RPS7 |
| Eukaryotic Translation Initiation | -4.6 | EIF1AX,RPL17,RPL21,RPL23,RPL26,RPL26L1,RPL27,RPL31,RPL34,RPL35A,RPL36A,RPL39,RPL41,RPS10,RPS15A,RPS17,RPS18,RPS21,RPS24,RPS27,RPS27L,RPS28,RPS29,RPS3A,RPS7 |
| Nonsense-Mediated Decay (NMD) | -4.491 | RPL17,RPL21,RPL23,RPL26,RPL26L1,RPL27,RPL31,RPL34,RPL35A,RPL36A,RPL39,RPL41,RPS10,RPS15A,RPS17,RPS18,RPS21,RPS24,RPS27,RPS27L,RPS28,RPS29,RPS3A,RPS7 |
| EIF2 Signaling | -3.357 | AGO2,DDIT3,EIF1AX,EIF2AK2,EIF4E3,RAP2A,RASD1,RPL17,RPL21,RPL23,RPL26,RPL26L1,RPL27,RPL31,RPL34,RPL35A,RPL36A,RPL39,RPL41,RPS10,RPS15A,RPS17,RPS18,RPS21,RPS24,RPS27,RPS27L,RPS28,RPS29,RPS3A,RPS7 |
| Pyroptosis Signaling Pathway | -3.771 | AIM2,CASP1,CASP4,CASP5,GAS5,GBP1,GBP4,GBP5,IL1B,MEFV,NEK7,NOL3,PRKAR1B,TLR1,TLR3,TLR5,TLR6,TLR7 |
| ISGylation Signaling Pathway | -3.3 | CHMP5,DTX3L,EIF2AK2,EP300,FLNB,IFIH1,IRF1,NFAT5,RIGI,STAT1,STAT2,TLR1,TLR3,TLR5,TLR6,TLR7,TRIM25,USP18 |
| Neutrophil degranulation | -6.633 | ABCA13,ADGRG3,ARG1,ATP11A,B2M,CAMP,CD177,CD58,CD59,CEACAM1,CLEC4D,CR1,CRISP3,CYSTM1,DDX3X,DNAJC3,FCAR,FCGR2A,FCGR3A/FCGR3B,FPR2,GCA,GPR84,HP,HPSE,HSPA6,ITGAX,LCN2,LILRB3,LTF,MCEMP1,MME,MMP8,OLFM4,ORM1,PLAU,PSMA2,QPCT,S100A12,S100A8,S100A9,SERPINB10,SLC11A1,SLPI,TNFAIP6 |
| Major pathway of rRNA processing in the nucleolus and cytosol | -4.491 | RPL17,RPL21,RPL23,RPL26,RPL26L1,RPL27,RPL31,RPL34,RPL35A,RPL36A,RPL39,RPL41,RPS10,RPS15A,RPS17,RPS18,RPS21,RPS24,RPS27,RPS27L,RPS28,RPS29,RPS3A,RPS7 |
| Multiple Sclerosis Signaling Pathway | -4.491 | C1QA,C1QB,C2,CASP1,EDA,FAS,GAS5,IL15,IL1B,IRF1,OSM,PARP12,PARP14,PARP9,PLAU,RNF213,SLC8A1,STAT1,TLR1,TLR3,TLR5,TLR6,TLR7,TNFSF10 |
| Role of Pattern Recognition Receptors in Recognition of Bacteria and Viruses | -3.606 | C1QA,C1QB,CASP1,CLEC7A,EDA,EIF2AK2,IFIH1,IL15,IL1B,NOD2,OSM,REL,RIGI,TLR1,TLR3,TLR5,TLR6,TLR7,TNFSF10 |
| Pathogen Induced Cytokine Storm Signaling Pathway | -3.889 | AIM2,CASP1,CASP7,CCL3L1,CKLF,CLEC7A,COL18A1,COL6A2,CSF2RB,CXCL10,EDA,IFIH1,IL15,IL1B,IL1RN,IRF1,NOD2,OSM,RIGI,SLC2A1,SOCS3,SRGN,STAT1,STX11,TLR1,TLR3,TLR5,TLR6,TLR7,TNFSF10,VEGFB,ZBP1 |
| IL-27 Signaling Pathway | -2.183 | B2M,CASP1,CD274,IRF1,JAK3,LPAR6,PTGS2,REL,RORA,SOCS3,STAT1,TLR1,TLR3,TLR5,TLR6,TLR7,TRIM69 |
| Parkinson's Signaling Pathway | -3.657 | CASP1,DDIT3,EDA,FCGR1A,FCGR2A,FCGR3A/FCGR3B,IL15,IL1B,LRRK2,MT-ND1,MT-ND2,MT-ND3,MT-ND4,MT-ND4L,MT-ND5,NDUFB1,NDUFB3,NOS3,OSM,SNCA,TLR1,TLR3,TLR5,TLR6,TLR7,TNFSF10,TUBA8 |
| Role of PKR in Interferon Induction and Antiviral Response | -2.673 | CASP1,CASP5,DNAJC3,EIF2AK2,FAS,FCGR1A,HSPA6,IFIH1,IL1B,IRF1,PDGFB,REL,RIGI,STAT1,STAT2,TLR3 |
| Inflammasome pathway | -2.449 | AIM2,CASP1,CASP5,IL1B,NEK7,NOD2 |
| TREM1 Signaling | -3.162 | CASP1,CASP5,IL1B,ITGAX,NOD2,REL,TLR1,TLR3,TLR5,TLR6,TLR7 |
| Class I MHC mediated antigen processing and presentation | -4.131 | B2M,CDC20,DTX3L,FBXL13,FBXL20,FCGR1A,KLHL13,KLHL2,LMO7,LY96,PSMA2,PSMA4,RBX1,RNF138,RNF19A,RNF213,S100A8,S100A9,SAR1B,SIAH2,SOCS3,TAP2,TLR1,TLR6,TRIM69,UBA6,UBE2C,UBE2E2,UBE2W,UBE3D,WSB1 |
| Role of Hypercytokinemia/hyperchemokinemia in the Pathogenesis of Influenza | -3.317 | CASP1,CXCL10,EIF2AK2,IFIT2,IL1B,IL1RN,RIGI,STAT1,STAT2,TLR3,TLR7 |
| Interferon gamma signaling | -3.464 | B2M,FCGR1A,GBP1,GBP4,GBP5,GBP6,IRF1,MT2A,SOCS3,STAT1,TRIM22,TRIM25 |
| Toll-like Receptor Signaling | -2.646 | EIF2AK2,IL1B,IL1RN,LY96,REL,TLR1,TLR3,TLR5,TLR6,TLR7 |
| Regulation of TLR by endogenous ligand | -2.236 | LY96,S100A8,S100A9,TLR1,TLR6 |
| Colorectal Cancer Metastasis Signaling | -2 | APC,FZD5,GNB4,GNG10,GNG11,GUCY1A1,JAK3,MMP11,MMP8,PRKAR1B,PTGS2,RAP2A,RASD1,REL,STAT1,TGFBR1,TLR1,TLR3,TLR5,TLR6,TLR7,VEGFB |
| Phagosome Formation | -3.812 | ABHD3,ADGRA2,ADGRE2,ADGRG3,ADORA3,AP1S2,CLEC4D,CLEC7A,CR1,DRD4,FCAR,FCGR1A,FCGR2A,FCGR3A/FCGR3B,FFAR2,FPR2,FPR3,FZD5,GPLD1,GPR141,GPR171,GPR25,GPR27,GPR84,HCAR2,HCAR3,ITGA7,ITGAX,ITGB4,JCHAIN,LIMK2,LPAR6,MARCKS,PLA2G4A,PLD4,PTGIR,RAP2A,RASD1,TBXA2R,TLR1,TLR3,TLR5,TLR6,TLR7 |
| NAFLD Signaling Pathway | -3.9 | ATF6,CASP1,CASP7,DDIT3,EDA,IL15,IL1B,JAK3,OSM,PPM1J,PTGS2,SOCS3,SOCS6,TLR1,TLR3,TLR5,TLR6,TLR7,TNFSF10 |
| Oncostatin M Signaling | -2.449 | JAK3,MT2A,OSM,PLAU,RAP2A,RASD1,STAT1 |
| NLR signaling pathways | -2.828 | AIM2,BIRC2,CASP1,CASP4,MEFV,NOD2,TAB3,TXN |
| Neuroinflammation Signaling Pathway | -3.13 | B2M,BIRC2,CASP1,CD200,CFLAR,CREB5,CXCL10,FAS,GABRR2,IL1B,JAK3,NFAT5,PLA2G4A,PTGS2,REL,SLC1A3,SNCA,STAT1,TGFBR1,TLR1,TLR3,TLR5,TLR6,TLR7 |
| NOD1/2 Signaling Pathway | -3.5 | BIRC2,CASP1,EDA,HSPA6,IL15,IL1B,NOD2,OSM,RIGI,TAB3,TLR1,TLR3,TLR5,TLR6,TLR7,TNFSF10 |
| Deubiquitination | -4.146 | APC,BIRC2,CCNA2,CDC20,EP300,H2BC18,H2BC9,IFIH1,NOD2,POLB,PSMA2,PSMA4,RIGI,SIAH2,TGFBR1,TRIM25,USP12,USP15,USP18,VCPIP1,YOD1 |
| Caspase activation via Death Receptors in the presence of ligand | -2 | CFLAR,FAS,LY96,TNFSF10 |
| Cachexia Signaling Pathway | -3.922 | AGO2,CASP1,CASP4,CASP5,CASP7,DDIT4,EDA,EIF2AK2,EP300,GUCY1A1,HSPA6,IL15,IL1B,IL1RN,LCN2,OSM,PHGDH,PRKAR1B,PSMA2,PSMA4,SOCS3,STAT1,STAT2,TGFBR1,TLR7,TNFSF10 |
| Crosstalk between Dendritic Cells and Natural Killer Cells | -2.121 | CD69,CSF2RB,FAS,IL15,IL3RA,KIR3DL1,REL,TLR3,TLR7,TNFSF10 |
| Necroptosis Signaling Pathway | -3.742 | BIRC2,CASP1,CFLAR,EIF2AK2,FAS,PLA2G4A,STAT1,STAT2,TIMM8B,TLR3,TNFSF10,TOMM5,TOMM7,ZBP1 |
| Pyroptosis | -2.236 | CASP1,CASP4,CASP5,IL1B,IRF1 |
| RORA activates gene expression | -2 | EP300,HELZ2,NCOA2,RORA |
| MyD88:MAL(TIRAP) cascade initiated on plasma membrane | -2.449 | LY96,S100A8,S100A9,TAB3,TLR1,TLR6 |
| Macrophage Classical Activation Signaling Pathway | -3.357 | CXCL10,EDA,GBP4,IL15,IL1B,IRF1,LY96,MT-CO1,OSM,PARP14,PARP9,SOCS3,STAT1,STAT2,TNFSF10 |
| Protein Ubiquitination Pathway | -3.9 | B2M,BIRC2,CDC20,DNAJA1,DNAJB14,DNAJC15,DNAJC3,HSPA6,IFT25,PSMA2,PSMA4,RBX1,TAP2,UBE2C,UBE2E2,UBE2T,UBE2W,USP12,USP15,USP18 |
| Molecular Mechanisms of Cancer | -2.429 | ADGRA2,ADGRE2,ADGRG3,ADORA3,APC,ARHGEF10,BIRC2,CASP7,CDKN2B,CFLAR,CHEK1,CSF2RB,DRD4,EP300,FAS,FFAR2,FPR2,FPR3,FZD5,GNB4,GNG10,GNG11,GPR141,GPR171,GPR25,GPR27,GPR84,GUCY1A1,HCAR2,HCAR3,IL1B,IL3RA,ITGA7,ITGAX,ITGB4,JAK3,LPAR6,MMP11,MMP8,NBN,PDGFB,PRKAR1B,PTGIR,RAP2A,RASD1,REL,TBXA2R,TGFA,TGFBR1 |
| Interferon alpha/beta signaling | -2.828 | IFIT2,IFIT5,IFITM1,IRF1,SOCS3,STAT1,STAT2,USP18 |
| TAK1-dependent IKK and NF-kappa-B activation | -2.449 | ALPK1,NOD2,S100A12,TAB3,TIFA,USP18 |
| NF-κB Signaling | -3.357 | EIF2AK2,EP300,FGFR2,IL1B,IL1RN,RAP2A,RASD1,TAB3,TGFA,TGFBR1,TLR1,TLR3,TLR5,TLR6,TLR7,TNFRSF17,TRAV1-1,TRAV12-1,TRAV21,TRAV29DV5,TRAV4,TRBV12-3,TRBV6-5,TRBV6-6,TRBV9 |
| Role of Macrophages, Fibroblasts and Endothelial Cells in Rheumatoid Arthritis | -2.558 | APC,CREB5,DKK3,FCGR1A,FCGR3A/FCGR3B,FZD5,IL15,IL1B,IL1RN,JCHAIN,NFAT5,OSM,PDGFB,RAP2A,RASD1,SOCS3,TLR1,TLR3,TLR5,TLR6,TLR7,VEGFB |
| Activation of IRF by Cytosolic Pattern Recognition Receptors | -2.449 | IFIH1,IFIT2,REL,RIGI,STAT1,STAT2,ZBP1 |
| Interleukin-4 and Interleukin-13 signaling | -2.53 | BCL6,IL1B,ITGAX,JAK3,LCN2,OSM,PTGS2,RORA,SOCS3,STAT1 |
| Atherosclerosis Signaling | -2.333 | ABHD3,ALOX15B,APOL1,COL18A1,IL1B,IL1RN,ORM1,PDGFB,PLA2G4A,REL,S100A8 |
| Interleukin-20 family signaling | -2 | JAK3,SOCS3,STAT1,STAT2 |
| Immunogenic Cell Death Signaling Pathway | -2.828 | CASP1,CFLAR,CXCL10,DDIT3,GZMK,HSPA6,IL1B,TLR3 |
| CGAS-STING Signaling Pathway | -3.317 | CASP1,CD274,EDA,IDO1,IL15,IL1B,IRF1,OSM,STAT1,TNFSF10,ZBP1 |
| Signaling by ALK | -2 | CD274,EP300,JAK3,MDK |
| Breast Cancer Regulation by Stathmin1 | -2.121 | ADGRA2,ADGRE2,ADGRG3,ADORA3,ARHGEF10,CREB5,DRD4,FFAR2,FPR2,FPR3,FZD5,GNB4,GNG10,GNG11,GPR141,GPR171,GPR25,GPR27,GPR84,HCAR2,HCAR3,LPAR6,PDGFB,PPM1J,PPP1R12A,PRKAR1B,PTGIR,RAP2A,RASD1,RB1CC1,TBXA2R,TGFA,TUBA8,VEGFB |
| Dendritic Cell Maturation | -3.207 | B2M,CD58,COL18A1,CREB5,FCGR1A,FCGR2A,FCGR3A/FCGR3B,IL15,IL1B,IL1RN,JCHAIN,REL,STAT1,STAT2,TLR3,TRAV1-1,TRAV12-1,TRAV21,TRAV29DV5,TRAV4,TRBV12-3,TRBV6-5,TRBV6-6,TRBV9 |
| Chaperone Mediated Autophagy Signaling Pathway | 3.138 | CASP1,CHEK1,DDX3X,EP300,FLT3,HSPA6,IL1B,LRRK2,MMP11,MMP8,NOS3,PSMA2,PSMA4,RICTOR,SNCA,SNX10,TPT1,TRAV1-1,TRAV12-1,TRAV21,TRAV29DV5,TRAV4,TRBV12-3,TRBV6-5,TRBV6-6,TRBV9 |
| Protein ubiquitination | -2 | UBA6,UBE2C,UBE2T,UBE2W |
| Mitotic G2-G2/M phases | -3.207 | CCNA2,CCNB1,CCNB2,CEP63,EP300,GTSE1,LIN54,MYBL2,PPP1R12A,PSMA2,PSMA4,RBX1,TPX2,TUBA8 |
| GABA receptor activation | -2.449 | GABRR2,GNB4,GNG10,GNG11,KCNJ15,KCNJ2 |
| Toll-like Receptor Cascades | -2 | LY96,TLR1,TLR6,TLR7 |
| Ephrin Receptor Signaling | -2.449 | CREB5,EPHA2,EPHB6,GNB4,GNG10,GNG11,ITGA7,ITGAX,ITGB4,LIMK2,PDGFB,RAP2A,RASD1,VEGFB |
| PPARα/RXRα Activation | 2.121 | BCL3,EP300,GK,GUCY1A1,HELZ2,IL1B,MED12,PRKAR1B,RAP2A,RASD1,REL,SLC27A1,TGFBR1 |
| RIPK1-mediated regulated necrosis | -2 | BIRC2,CFLAR,FAS,TNFSF10 |
| Mitotic G1 phase and G1/S transition | -3 | CCNA2,CCNB1,CDKN2B,LIN54,MYBL2,ORC4,PSMA2,PSMA4,TOP2A,TYMS |
| Cargo concentration in the ER | -2 | CD59,F5,SAR1B,TGFA |
| ***MDD responders with CRP<1 vs. responders with CRP>1*** | | |
| Interferon alpha/beta signaling | -4.69 | IFI35,IFI6,IFIT1,IFIT2,IFIT3,IFIT5,IFITM1,IFITM3,IRF7,ISG15,ISG20,MX1,MX2,OAS2,OAS3,OASL,RSAD2,SOCS1,SOCS3,STAT1,STAT2,USP18 |
| Role of Hypercytokinemia/hyperchemokinemia in the Pathogenesis of Influenza | -4 | CXCL10,EIF2AK2,IFIT2,IFIT3,IL1B,IL1RN,IRF7,ISG15,ISG20,MX1,OAS2,OAS3,RIGI,RSAD2,STAT1,STAT2 |
| Interferon Signaling | -2.714 | IFI35,IFI6,IFIT1,IFIT3,IFITM1,IFITM3,ISG15,MX1,SOCS1,STAT1,STAT2 |
| Interferon gamma signaling | -3.742 | FCGR1A,GBP1,GBP4,IRF7,MT2A,OAS2,OAS3,OASL,PML,SOCS1,SOCS3,STAT1,TRIM22,TRIM25 |
| Neutrophil degranulation | -5.568 | ABCA13,AOC1,AZU1,BPI,CAMP,CEACAM1,CEACAM6,CEACAM8,CHIT1,CRISP3,CYSTM1,DEFA1 (includes others),ELANE,ENPP4,HP,KRT1,LCN2,LTF,MCEMP1,MMP8,MPO,OLFM4,OLR1,PTX3,RETN,RNASE3,S100A8,SERPINB10,SLC2A5,TCN1,TNFAIP6 |
| ISGylation Signaling Pathway | -2.887 | DTX3L,EIF2AK2,HERC5,IFIH1,IRF7,ISG15,RIGI,STAT1,STAT2,TRIM25,UBE2L6,USP18 |
| ISG15 antiviral mechanism | -3.162 | EIF2AK2,HERC5,IFIT1,ISG15,MX1,MX2,RIGI,STAT1,TRIM25,UBE2L6 |
| Macrophage Classical Activation Signaling Pathway | -2.324 | BPI,CD70,CXCL10,EDA,GBP4,IL1B,LY96,OSM,PARP14,PARP9,SOCS1,SOCS3,STAT1,STAT2,TNFSF10 |
| OAS antiviral response | -2 | OAS2,OAS3,OASL,RIGI |
| Role of Pattern Recognition Receptors in Recognition of Bacteria and Viruses | -2.236 | CD70,EDA,EIF2AK2,IFIH1,IL1B,IRF7,OAS2,OAS3,OSM,PTX3,RIGI,TNFSF10 |
| Antimicrobial peptides | -2.449 | BPI,CAMP,LCN2,LTF,RNASE3,S100A8 |
| Pathogen Induced Cytokine Storm Signaling Pathway | -3.9 | AIM2,CCL3L1,CCR1,CD70,CXCL10,DHX58,EDA,EOMES,IFIH1,IL1B,IL1RN,IRF7,OSM,RIGI,SLC2A5,SOCS3,STAT1,TNFSF10,ZBP1 |
| Class I MHC mediated antigen processing and presentation | -4.359 | CDC20,DTX3L,FBXO2,FBXO6,FBXO9,FCGR1A,HERC5,HERC6,KBTBD8,LY96,MRC2,RNF213,S100A8,SOCS1,SOCS3,TRIM69,UBE2C,UBE2L6,UBE2S |
| Airway Pathology in Chronic Obstructive Pulmonary Disease | -2 | CD70,EDA,ELANE,IL1B,LCN2,MMP8,MPO,OSM,TNFSF10 |
| DDX58/IFIH1-mediated induction of interferon-alpha/beta | -2.646 | HERC5,IFIH1,IRF7,ISG15,RIGI,TRIM25,UBE2L6 |
| Multiple Sclerosis Signaling Pathway | -3.464 | CD70,EDA,IL1B,MPO,OSM,PARP12,PARP14,PARP9,RNF213,SLC4A1,STAT1,TNFSF10 |
| Interleukin-4 and Interleukin-13 signaling | -2.121 | BCL6,IL1B,LCN2,OSM,RORA,SOCS1,SOCS3,STAT1 |
| Role of PKR in Interferon Induction and Antiviral Response | -2.646 | CASP5,EIF2AK2,FCGR1A,IFIH1,IL1B,RIGI,STAT1,STAT2 |
| CGAS-STING Signaling Pathway | -2.828 | CD274,CD70,EDA,IL1B,OSM,STAT1,TNFSF10,ZBP1 |
| Systemic Lupus Erythematosus in B Cell Signaling Pathway | -3.207 | CD70,EDA,IFIH1,IFIT2,IFIT3,IGHV3-73,IGHV7-4-1,IGLV2-14,IGLV3-27,IL1B,IRF7,ISG15,ISG20,OSM,RASD1,STAT1,STAT2,TNFSF10 |
| Regulation of mitotic cell cycle | -2.449 | AURKA,CCNA2,CDC20,CDK2,UBE2C,UBE2S |
| Wound Healing Signaling Pathway | -2.111 | CD70,EDA,HBEGF,IL1B,IL1RN,MMP8,OSM,RASD1,STAT1,TNFSF10,TRPV1 |
| Cell Cycle Checkpoints | -2.309 | BRIP1,CCNA2,CDC20,CDK2,CENPM,GTSE1,H2BC9,KNL1,PKMYT1,UBE2C,UBE2S,WRN |
| Necroptosis Signaling Pathway | -2.121 | EIF2AK2,PLA2G4C,SLC25A10,STAT1,STAT2,TIMM10,TNFSF10,ZBP1 |
| CDX Gastrointestinal Cancer Signaling Pathway | 3 | CD70,CDK2,EDA,FZD3,HBEGF,IL1B,OSM,TNFSF10,WNT10B |
| Retinoic acid Mediated Apoptosis Signaling | -2 | PARP12,PARP14,PARP9,TNFSF10 |
| NOD1/2 Signaling Pathway | -2.121 | CD70,DEFA1 (includes others),EDA,IL1B,IRF7,OSM,RIGI,TNFSF10 |
| FXR/RXR Activation | 2.121 | CD70,EDA,IL1B,IL1RN,OSM,SOCS3,TNFSF10,VLDLR |
| NFKBIE Signaling Pathway | -3.162 | CD70,TNFSF10,TRAV1-1,TRAV14DV4,TRAV16,TRAV22,TRAV27,TRAV41,TRAV8-6,TRBV24-1 |
| \| \| ***MDD responders with CRP<1 vs. non-responders/unmedicated with CRP<1*** \| \| --- \| \| \| --- \| --- \| | | |
| Lipid Antigen Presentation by CD1 | -5.209 | CD1A,CD3E,TRAV12-1,TRAV12-3,TRAV13-1,TRAV16,TRAV17,TRAV21,TRAV22,TRAV24,TRAV27,TRAV29DV5,TRAV38-2DV8,TRAV4,TRAV8-3,TRAV8-4,TRAV9-2,TRBV12-3,TRBV14,TRBV23-1,TRBV24-1,TRBV25-1,TRBV29-1,TRBV4-2,TRBV5-4,TRBV6-5,TRBV6-6,TRBV7-2,TRBV7-6,TRBV9,TRGC2 |
| Communication between Innate and Adaptive Immune Cells | 2.496 | CCL4,CCL5,CD3E,CD8A,IGHG1,IGHV3-43,IGHV3-7,IGHV7-4-1,IGKV1-6,IGKV1D-13,IGKV2-30,IGLV2-14,IGLV4-69,JCHAIN,TNFRSF17,TRAV12-1,TRAV12-3,TRAV13-1,TRAV16,TRAV17,TRAV21,TRAV22,TRAV24,TRAV27,TRAV29DV5,TRAV38-2DV8,TRAV4,TRAV8-3,TRAV8-4,TRAV9-2,TRBV12-3,TRBV14,TRBV23-1,TRBV24-1,TRBV25-1,TRBV29-1,TRBV4-2,TRBV5-4,TRBV6-5,TRBV6-6,TRBV7-2,TRBV7-6,TRBV9,TRGC2 |
| NFKBIE Signaling Pathway | -5.477 | CD3E,TRAV12-1,TRAV12-3,TRAV13-1,TRAV16,TRAV17,TRAV21,TRAV22,TRAV24,TRAV27,TRAV29DV5,TRAV38-2DV8,TRAV4,TRAV8-3,TRAV8-4,TRAV9-2,TRBV12-3,TRBV14,TRBV23-1,TRBV24-1,TRBV25-1,TRBV29-1,TRBV4-2,TRBV5-4,TRBV6-5,TRBV6-6,TRBV7-2,TRBV7-6,TRBV9,TRGC2 |
| G Protein Signaling Mediated by Tubby | -5.477 | CD3E,TRAV12-1,TRAV12-3,TRAV13-1,TRAV16,TRAV17,TRAV21,TRAV22,TRAV24,TRAV27,TRAV29DV5,TRAV38-2DV8,TRAV4,TRAV8-3,TRAV8-4,TRAV9-2,TRBV12-3,TRBV14,TRBV23-1,TRBV24-1,TRBV25-1,TRBV29-1,TRBV4-2,TRBV5-4,TRBV6-5,TRBV6-6,TRBV7-2,TRBV7-6,TRBV9,TRGC2,TTC21B |
| Regulation of IL-2 Expression in Activated and Anergic T Lymphocytes | -5.385 | CD3E,TRAV12-1,TRAV12-3,TRAV13-1,TRAV16,TRAV17,TRAV21,TRAV22,TRAV24,TRAV27,TRAV29DV5,TRAV38-2DV8,TRAV4,TRAV8-3,TRAV8-4,TRAV9-2,TRBV12-3,TRBV14,TRBV23-1,TRBV24-1,TRBV25-1,TRBV29-1,TRBV4-2,TRBV5-4,TRBV6-5,TRBV6-6,TRBV7-2,TRBV7-6,TRBV9 |
| NF-κB Signaling | 2 | CD3E,EP300,IGF1R,TGFA,TNFRSF17,TRAV12-1,TRAV12-3,TRAV13-1,TRAV16,TRAV17,TRAV21,TRAV22,TRAV24,TRAV27,TRAV29DV5,TRAV38-2DV8,TRAV4,TRAV8-3,TRAV8-4,TRAV9-2,TRBV12-3,TRBV14,TRBV23-1,TRBV24-1,TRBV25-1,TRBV29-1,TRBV4-2,TRBV5-4,TRBV6-5,TRBV6-6,TRBV7-2,TRBV7-6,TRBV9,TRGC2 |
| IL-4 Signaling | -5.488 | CD3E,COL6A1,COL6A2,CREB1,FAS,TRAV12-1,TRAV12-3,TRAV13-1,TRAV16,TRAV17,TRAV21,TRAV22,TRAV24,TRAV27,TRAV29DV5,TRAV38-2DV8,TRAV4,TRAV8-3,TRAV8-4,TRAV9-2,TRBV12-3,TRBV14,TRBV23-1,TRBV24-1,TRBV25-1,TRBV29-1,TRBV4-2,TRBV5-4,TRBV6-5,TRBV6-6,TRBV7-2,TRBV7-6,TRBV9,TRGC2 |
| CTLA4 Signaling in Cytotoxic T Lymphocytes | 5.145 | AP1S3,CD3E,CD8A,FOXP3,GPLD1,TRAV12-1,TRAV12-3,TRAV13-1,TRAV16,TRAV17,TRAV21,TRAV22,TRAV24,TRAV27,TRAV29DV5,TRAV38-2DV8,TRAV4,TRAV8-3,TRAV8-4,TRAV9-2,TRBV12-3,TRBV14,TRBV23-1,TRBV24-1,TRBV25-1,TRBV29-1,TRBV4-2,TRBV5-4,TRBV6-5,TRBV6-6,TRBV7-2,TRBV7-6,TRBV9,TRGC2 |
| T Cell Receptor Signaling | -5.303 | CD3E,CD8A,DUSP6,TCF7L2,TRAV12-1,TRAV12-3,TRAV13-1,TRAV16,TRAV17,TRAV21,TRAV22,TRAV24,TRAV27,TRAV29DV5,TRAV38-2DV8,TRAV4,TRAV8-3,TRAV8-4,TRAV9-2,TRBV12-3,TRBV14,TRBV23-1,TRBV24-1,TRBV25-1,TRBV29-1,TRBV4-2,TRBV5-4,TRBV6-5,TRBV6-6,TRBV7-2,TRBV7-6,TRBV9,TRGC2 |
| Chaperone Mediated Autophagy Signaling Pathway | -5.488 | BAG1,CD3E,EP300,IGF1R,NOS3,TRAV12-1,TRAV12-3,TRAV13-1,TRAV16,TRAV17,TRAV21,TRAV22,TRAV24,TRAV27,TRAV29DV5,TRAV38-2DV8,TRAV4,TRAV8-3,TRAV8-4,TRAV9-2,TRBV12-3,TRBV14,TRBV23-1,TRBV24-1,TRBV25-1,TRBV29-1,TRBV4-2,TRBV5-4,TRBV6-5,TRBV6-6,TRBV7-2,TRBV7-6,TRBV9,TRGC2 |
| Eukaryotic Translation Elongation | 4 | EEF1B2,RPL17,RPL23,RPL26,RPL27,RPL31,RPL34,RPL36A,RPL39,RPS15A,RPS21,RPS24,RPS27L,RPS29,RPS3A,RPS7 |
| Eukaryotic Translation Termination | 3.873 | RPL17,RPL23,RPL26,RPL27,RPL31,RPL34,RPL36A,RPL39,RPS15A,RPS21,RPS24,RPS27L,RPS29,RPS3A,RPS7 |
| SRP-dependent cotranslational protein targeting to membrane | 4 | RPL17,RPL23,RPL26,RPL27,RPL31,RPL34,RPL36A,RPL39,RPS15A,RPS21,RPS24,RPS27L,RPS29,RPS3A,RPS7,SEC61A2 |
| Response of EIF2AK4 (GCN2) to amino acid deficiency | 3.873 | RPL17,RPL23,RPL26,RPL27,RPL31,RPL34,RPL36A,RPL39,RPS15A,RPS21,RPS24,RPS27L,RPS29,RPS3A,RPS7 |
| Selenoamino acid metabolism | 3.873 | RPL17,RPL23,RPL26,RPL27,RPL31,RPL34,RPL36A,RPL39,RPS15A,RPS21,RPS24,RPS27L,RPS29,RPS3A,RPS7 |
| Nonsense-Mediated Decay (NMD) | 3.873 | RPL17,RPL23,RPL26,RPL27,RPL31,RPL34,RPL36A,RPL39,RPS15A,RPS21,RPS24,RPS27L,RPS29,RPS3A,RPS7 |
| Eukaryotic Translation Initiation | 3.873 | RPL17,RPL23,RPL26,RPL27,RPL31,RPL34,RPL36A,RPL39,RPS15A,RPS21,RPS24,RPS27L,RPS29,RPS3A,RPS7 |
| FAK Signaling | -3.618 | ACVR2A,ADGRE2,ADORA2B,APC,C5AR2,CCR1,CD3E,GPER1,GPR153,IGF1R,IL3RA,IL9R,ITGA7,ITGAX,TCF7L2,TRAV12-1,TRAV12-3,TRAV13-1,TRAV16,TRAV17,TRAV21,TRAV22,TRAV24,TRAV27,TRAV29DV5,TRAV38-2DV8,TRAV4,TRAV8-3,TRAV8-4,TRAV9-2,TRBV12-3,TRBV14,TRBV23-1,TRBV24-1,TRBV25-1,TRBV29-1,TRBV4-2,TRBV5-4,TRBV6-5,TRBV6-6,TRBV7-2,TRBV7-6,TRBV9,TRGC2 |
| Interferon alpha/beta signaling | -3.317 | IFI6,IFIT1,IFIT3,IRF7,ISG15,MX1,OAS2,OAS3,OASL,RSAD2,USP18 |
| Major pathway of rRNA processing in the nucleolus and cytosol | 3.153 | NOC4L,RPL17,RPL23,RPL26,RPL27,RPL31,RPL34,RPL36A,RPL39,RPS15A,RPS21,RPS24,RPS27L,RPS29,RPS3A,RPS7,WDR18 |
| EIF2 Signaling | 3 | IGF1R,RPL17,RPL23,RPL26,RPL27,RPL31,RPL34,RPL36A,RPL39,RPS15A,RPS21,RPS24,RPS27L,RPS29,RPS3A,RPS7,VEGFA |
| Role of Hypercytokinemia/hyperchemokinemia in the Pathogenesis of Influenza | -3 | CCL4,CCL5,IFIT3,IRF7,ISG15,MX1,OAS2,OAS3,RSAD2 |
| Interferon Signaling | -2.236 | IFI6,IFIT1,IFIT3,ISG15,MX1 |
| Transcriptional regulation by the AP-2 (TFAP2) family of transcription factors | 2 | EP300,TGFA,VEGFA,WWOX |
| Costimulation by the CD28 family | -2.236 | CD3E,TRAC,TRAV29DV5,TRAV8-4,TRBV12-3 |
| Cilium Assembly | 2.333 | CEP63,CEP97,IFT22,LZTFL1,PLK4,RAB3IP,TRAF3IP1,TRIP11,TTC21B |
| Transcripts are selected for p<.05 and FC>\|1.2\|; pathways are selected for z-scores ≥\|2\| and listed based on p-values (smaller to the top), all p<0.05. | | |

# ***Supplementary Table S8:*** *PANTHER Gene Ontology enrichment analyses in all group comparisons (CRP-based, treatment-based, and merged groups)*

| **PANTHER Gene Ontology** | *No. of genes* | *p-value* | *FDR-adjusted p-value* |
| --- | --- | --- | --- |
| **CRP-based** | | | |
| ***MDD CRP 1-3 mg/L vs. controls*** | | | |
| *Biological processes* | | | |
| defense response to virus | 38 | 1.18E-17 | 1.80E-13 |
| defense response to other organism | 72 | 3.68E-15 | 2.80E-11 |
| response to external biotic stimulus | 85 | 2.94E-14 | 7.48E-11 |
| defense response to symbiont | 66 | 2.05E-14 | 7.83E-11 |
| response to other organism | 85 | 2.67E-14 | 8.15E-11 |
| response to virus | 40 | 1.64E-14 | 8.31E-11 |
| innate immune response | 59 | 3.84E-14 | 8.37E-11 |
| defense response | 87 | 4.98E-14 | 9.49E-11 |
| response to biotic stimulus | 86 | 7.58E-14 | 1.28E-10 |
| biological process involved in interspecies interaction between organisms | 90 | 1.41E-13 | 2.15E-10 |
| immune system process | 117 | 2.05E-13 | 2.84E-10 |
| negative regulation of viral genome replication | 16 | 2.94E-13 | 3.73E-10 |
| immune response | 92 | 1.71E-12 | 2.00E-09 |
| regulation of viral life cycle | 22 | 1.75E-11 | 1.91E-08 |
| negative regulation of viral process | 18 | 3.27E-11 | 3.32E-08 |
| regulation of viral process | 23 | 5.92E-11 | 5.64E-08 |
| regulation of response to biotic stimulus | 41 | 1.54E-10 | 1.38E-07 |
| regulation of viral genome replication | 16 | 3.84E-10 | 3.25E-07 |
| response to external stimulus | 105 | 1.47E-09 | 1.12E-06 |
| regulation of response to external stimulus | 63 | 1.42E-09 | 1.14E-06 |
| regulation of immune response | 55 | 4.28E-09 | 3.10E-06 |
| regulation of innate immune response | 34 | 6.56E-09 | 4.54E-06 |
| cytoplasmic translation | 17 | 2.76E-08 | 1.83E-05 |
| response to cytokine | 49 | 4.33E-08 | 2.75E-05 |
| antiviral innate immune response | 11 | 4.95E-08 | 2.79E-05 |
| negative regulation of response to biotic stimulus | 16 | 4.65E-08 | 2.83E-05 |
| regulation of immune system process | 75 | 4.83E-08 | 2.83E-05 |
| regulation of defense response | 47 | 7.86E-08 | 4.28E-05 |
| response to type I interferon | 11 | 9.28E-08 | 4.88E-05 |
| interleukin-27-mediated signaling pathway | 5 | 2.11E-07 | 1.07E-04 |
| negative regulation of innate immune response | 13 | 2.97E-07 | 1.46E-04 |
| positive regulation of immune system process | 52 | 1.38E-06 | 6.56E-04 |
| response to interferon-beta | 8 | 1.69E-06 | 7.80E-04 |
| cellular response to type I interferon | 9 | 1.99E-06 | 8.92E-04 |
| cellular response to cytokine stimulus | 41 | 2.94E-06 | 1.28E-03 |
| positive regulation of immune response | 37 | 6.01E-06 | 2.54E-03 |
| negative regulation of response to external stimulus | 27 | 6.98E-06 | 2.88E-03 |
| B cell mediated immunity | 17 | 9.54E-06 | 3.83E-03 |
| response to bacterium | 38 | 1.01E-05 | 3.95E-03 |
| positive regulation of innate immune response | 21 | 1.10E-05 | 4.20E-03 |
| translation | 26 | 1.19E-05 | 4.30E-03 |
| regulation of response to stimulus | 142 | 1.16E-05 | 4.32E-03 |
| positive regulation of response to biotic stimulus | 22 | 1.40E-05 | 4.97E-03 |
| type I interferon-mediated signaling pathway | 8 | 1.57E-05 | 5.43E-03 |
| negative regulation of immune system process | 30 | 1.85E-05 | 6.26E-03 |
| immunoglobulin mediated immune response | 16 | 2.66E-05 | 8.80E-03 |
| response to stress | 124 | 2.90E-05 | 9.39E-03 |
| positive regulation of response to stimulus | 88 | 3.43E-05 | 1.09E-02 |
| peptide transport | 11 | 3.97E-05 | 1.23E-02 |
| positive regulation of response to external stimulus | 31 | 4.52E-05 | 1.38E-02 |
| regulation of ribonuclease activity | 4 | 4.72E-05 | 1.41E-02 |
| peptide biosynthetic process | 26 | 5.48E-05 | 1.58E-02 |
| cytokine-mediated signaling pathway | 24 | 5.38E-05 | 1.58E-02 |
| cellular response to organic substance | 71 | 5.71E-05 | 1.61E-02 |
| complement activation, classical pathway | 7 | 5.97E-05 | 1.66E-02 |
| MDA-5 signaling pathway | 3 | 6.43E-05 | 1.75E-02 |
| regulation of hydrolase activity | 38 | 6.75E-05 | 1.80E-02 |
| amide transport | 13 | 7.08E-05 | 1.86E-02 |
| positive regulation of defense response | 25 | 8.30E-05 | 2.11E-02 |
| positive regulation of multicellular organismal process | 68 | 8.19E-05 | 2.11E-02 |
| regulation of type I interferon-mediated signaling pathway | 7 | 1.12E-04 | 2.68E-02 |
| lymphocyte mediated immunity | 18 | 1.12E-04 | 2.70E-02 |
| regulation of response to stress | 58 | 1.08E-04 | 2.71E-02 |
| negative regulation of immune response | 15 | 1.11E-04 | 2.72E-02 |
| response to interferon-alpha | 5 | 1.18E-04 | 2.77E-02 |
| regulation of response to cytokine stimulus | 14 | 1.27E-04 | 2.93E-02 |
| humoral immune response mediated by circulating immunoglobulin | 7 | 1.30E-04 | 2.96E-02 |
| interferon-mediated signaling pathway | 8 | 1.33E-04 | 2.99E-02 |
| chromatin remodeling | 3 | 1.42E-04 | 3.14E-02 |
| chromatin organization | 5 | 1.49E-04 | 3.21E-02 |
| regulation of catalytic activity | 59 | 1.49E-04 | 3.24E-02 |
| tube lumen cavitation | 3 | 1.58E-04 | 3.29E-02 |
| salivary gland cavitation | 3 | 1.58E-04 | 3.34E-02 |
| response to organic substance | 92 | 1.66E-04 | 3.42E-02 |
| regulation of molecular function | 78 | 1.75E-04 | 3.42E-02 |
| regulation of cytokine production | 38 | 1.69E-04 | 3.44E-02 |
| negative regulation of cell adhesion | 19 | 1.74E-04 | 3.44E-02 |
| regulation of leukocyte proliferation | 18 | 1.72E-04 | 3.44E-02 |
| regulation of nuclease activity | 5 | 1.93E-04 | 3.72E-02 |
| activation of immune response | 23 | 2.04E-04 | 3.89E-02 |
| adaptive immune response based on somatic recombination of immune receptors built from immunoglobulin superfamily domains | 18 | 2.16E-04 | 4.06E-02 |
| organonitrogen compound biosynthetic process | 57 | 2.29E-04 | 4.26E-02 |
| regulation of cytokine-mediated signaling pathway | 13 | 2.36E-04 | 4.28E-02 |
| negative regulation of type I interferon-mediated signaling pathway | 5 | 2.41E-04 | 4.32E-02 |
| positive regulation of pattern recognition receptor signaling pathway | 8 | 2.35E-04 | 4.32E-02 |
| amide biosynthetic process | 29 | 2.56E-04 | 4.54E-02 |
| regulation of pattern recognition receptor signaling pathway | 13 | 2.67E-04 | 4.67E-02 |
| *Molecular functions* | | | |
| structural constituent of ribosome | 23 | 5.24E-11 | 2.65E-07 |
| mRNA 5'-UTR binding | 6 | 2.40E-05 | 4.06E-02 |
| 2'-5'-oligoadenylate synthetase activity | 3 | 1.64E-05 | 4.15E-02 |
| *Cellular components* | | | |
| cytosolic ribosome | 19 | 3.56E-10 | 3.56E-07 |
| ribosome | 27 | 2.36E-10 | 4.72E-07 |
| ribosomal subunit | 22 | 6.28E-09 | 4.18E-06 |
| cytosolic large ribosomal subunit | 10 | 2.51E-06 | 1.26E-03 |
| small ribosomal subunit | 11 | 7.15E-06 | 2.86E-03 |
| cytosolic small ribosomal subunit | 8 | 2.19E-05 | 7.29E-03 |
| ***MDD CRP>3 mg/L vs. controls*** | | | |
| *Biological processes* | | | |
| cytoplasmic translation | 38 | 1.56E-26 | 2.38E-22 |
| translation | 51 | 1.91E-17 | 9.72E-14 |
| peptide biosynthetic process | 53 | 1.79E-17 | 1.37E-13 |
| amide biosynthetic process | 58 | 9.40E-16 | 3.58E-12 |
| peptide metabolic process | 57 | 6.84E-15 | 2.09E-11 |
| oxidative phosphorylation | 25 | 5.90E-14 | 1.50E-10 |
| immune response | 108 | 1.21E-12 | 2.63E-09 |
| immune system process | 133 | 9.88E-12 | 1.88E-08 |
| ATP synthesis coupled electron transport | 19 | 8.92E-11 | 1.24E-07 |
| mitochondrial ATP synthesis coupled electron transport | 19 | 8.92E-11 | 1.36E-07 |
| aerobic respiration | 25 | 8.34E-11 | 1.41E-07 |
| amide metabolic process | 63 | 1.85E-10 | 2.35E-07 |
| aerobic electron transport chain | 18 | 2.74E-10 | 3.21E-07 |
| organonitrogen compound biosynthetic process | 87 | 3.97E-10 | 4.33E-07 |
| respiratory electron transport chain | 20 | 5.97E-10 | 6.06E-07 |
| cellular respiration | 26 | 7.12E-10 | 6.78E-07 |
| cellular nitrogen compound biosynthetic process | 94 | 8.89E-10 | 7.97E-07 |
| defense response | 87 | 8.64E-09 | 7.32E-06 |
| response to other organism | 82 | 4.66E-08 | 3.74E-05 |
| response to external biotic stimulus | 82 | 4.91E-08 | 3.74E-05 |
| defense response to bacterium | 30 | 6.27E-08 | 4.55E-05 |
| cellular process | 534 | 1.09E-07 | 7.55E-05 |
| response to biotic stimulus | 83 | 1.17E-07 | 7.76E-05 |
| antimicrobial humoral immune response mediated by antimicrobial peptide | 16 | 1.84E-07 | 1.17E-04 |
| defense response to fungus | 12 | 2.30E-07 | 1.40E-04 |
| antimicrobial humoral response | 19 | 3.16E-07 | 1.85E-04 |
| electron transport chain | 20 | 3.90E-07 | 2.20E-04 |
| energy derivation by oxidation of organic compounds | 26 | 4.11E-07 | 2.24E-04 |
| proton motive force-driven ATP synthesis | 13 | 4.60E-07 | 2.42E-04 |
| defense response to other organism | 63 | 5.50E-07 | 2.79E-04 |
| defense response to symbiont | 58 | 6.29E-07 | 3.09E-04 |
| response to external stimulus | 115 | 7.14E-07 | 3.30E-04 |
| proton motive force-driven mitochondrial ATP synthesis | 12 | 7.13E-07 | 3.39E-04 |
| biological process involved in interspecies interaction between organisms | 85 | 8.84E-07 | 3.96E-04 |
| inflammatory response | 41 | 9.72E-07 | 4.23E-04 |
| response to bacterium | 47 | 1.20E-06 | 4.94E-04 |
| ribosomal small subunit biogenesis | 15 | 1.18E-06 | 4.99E-04 |
| cell killing | 19 | 1.52E-06 | 6.10E-04 |
| response to fungus | 12 | 1.65E-06 | 6.46E-04 |
| immune effector process | 36 | 2.27E-06 | 8.64E-04 |
| ATP biosynthetic process | 13 | 2.42E-06 | 9.00E-04 |
| killing of cells of another organism | 15 | 2.71E-06 | 9.60E-04 |
| disruption of cell in another organism | 15 | 2.71E-06 | 9.83E-04 |
| leukocyte mediated immunity | 27 | 3.22E-06 | 1.12E-03 |
| innate immune response in mucosa | 7 | 3.62E-06 | 1.23E-03 |
| disruption of anatomical structure in another organism | 15 | 3.79E-06 | 1.25E-03 |
| mucosal immune response | 8 | 4.19E-06 | 1.36E-03 |
| sensory perception of chemical stimulus | 2 | 5.62E-06 | 1.79E-03 |
| sensory perception of smell | 1 | 6.72E-06 | 2.09E-03 |
| antibacterial humoral response | 11 | 6.89E-06 | 2.10E-03 |
| defense response to Gram-negative bacterium | 13 | 8.70E-06 | 2.60E-03 |
| nucleoside triphosphate biosynthetic process | 14 | 9.51E-06 | 2.68E-03 |
| purine ribonucleoside triphosphate biosynthetic process | 13 | 9.79E-06 | 2.71E-03 |
| humoral immune response | 23 | 9.49E-06 | 2.73E-03 |
| response to stimulus | 316 | 9.46E-06 | 2.77E-03 |
| organ or tissue specific immune response | 8 | 1.12E-05 | 2.94E-03 |
| generation of precursor metabolites and energy | 31 | 1.11E-05 | 2.97E-03 |
| purine nucleoside triphosphate biosynthetic process | 13 | 1.10E-05 | 3.00E-03 |
| erythrocyte homeostasis | 14 | 1.18E-05 | 3.04E-03 |
| mitochondrial electron transport, NADH to ubiquinone | 9 | 1.23E-05 | 3.12E-03 |
| detection of chemical stimulus involved in sensory perception of smell | 1 | 1.42E-05 | 3.56E-03 |
| regulation of defense response | 48 | 1.65E-05 | 4.07E-03 |
| ribonucleoside triphosphate biosynthetic process | 13 | 1.92E-05 | 4.65E-03 |
| adaptive immune response | 43 | 1.98E-05 | 4.72E-03 |
| innate immune response | 47 | 2.16E-05 | 4.98E-03 |
| mitochondrial respiratory chain complex assembly | 13 | 2.14E-05 | 5.02E-03 |
| positive regulation of defense response | 30 | 2.65E-05 | 6.04E-03 |
| regulation of response to external stimulus | 61 | 2.73E-05 | 6.11E-03 |
| ribosome biogenesis | 25 | 3.06E-05 | 6.75E-03 |
| regulation of nucleobase-containing compound metabolic process | 89 | 3.32E-05 | 7.24E-03 |
| respiratory chain complex IV assembly | 7 | 3.46E-05 | 7.44E-03 |
| detection of chemical stimulus involved in sensory perception | 2 | 3.68E-05 | 7.80E-03 |
| myeloid cell homeostasis | 15 | 4.15E-05 | 8.67E-03 |
| regulation of cell killing | 13 | 4.81E-05 | 9.78E-03 |
| purine ribonucleotide biosynthetic process | 18 | 4.81E-05 | 9.90E-03 |
| response to molecule of bacterial origin | 26 | 5.18E-05 | 1.04E-02 |
| ATP metabolic process | 16 | 6.31E-05 | 1.25E-02 |
| 7-methylguanosine cap hypermethylation | 4 | 6.45E-05 | 1.26E-02 |
| lymphocyte mediated immunity | 21 | 8.19E-05 | 1.58E-02 |
| mitochondrial electron transport, cytochrome c to oxygen | 6 | 8.42E-05 | 1.60E-02 |
| purine ribonucleoside triphosphate metabolic process | 17 | 9.94E-05 | 1.87E-02 |
| mitochondrial cytochrome c oxidase assembly | 6 | 1.08E-04 | 1.96E-02 |
| regulation of immune response | 51 | 1.07E-04 | 1.96E-02 |
| modulation of process of another organism | 5 | 1.05E-04 | 1.96E-02 |
| homeostasis of number of cells | 22 | 1.10E-04 | 1.98E-02 |
| positive regulation of response to external stimulus | 35 | 1.13E-04 | 2.00E-02 |
| Unclassified | 57 | 1.24E-04 | 2.14E-02 |
| ribonucleoprotein complex biogenesis | 31 | 1.25E-04 | 2.15E-02 |
| biological_process | 599 | 1.24E-04 | 2.17E-02 |
| response to lipopolysaccharide | 24 | 1.34E-04 | 2.23E-02 |
| ribonucleotide biosynthetic process | 18 | 1.34E-04 | 2.25E-02 |
| myeloid leukocyte activation | 15 | 1.33E-04 | 2.26E-02 |
| response to stress | 146 | 1.46E-04 | 2.35E-02 |
| ribonucleotide metabolic process | 28 | 1.43E-04 | 2.35E-02 |
| cellular nitrogen compound metabolic process | 141 | 1.46E-04 | 2.36E-02 |
| purine nucleoside triphosphate metabolic process | 17 | 1.56E-04 | 2.43E-02 |
| purine nucleotide biosynthetic process | 19 | 1.53E-04 | 2.43E-02 |
| ribonucleoside triphosphate metabolic process | 17 | 1.56E-04 | 2.45E-02 |
| purine ribonucleotide metabolic process | 27 | 1.63E-04 | 2.50E-02 |
| regulation of RNA metabolic process | 84 | 1.70E-04 | 2.59E-02 |
| regulation of response to stimulus | 165 | 1.96E-04 | 2.90E-02 |
| positive regulation of inflammatory response | 14 | 1.96E-04 | 2.92E-02 |
| regulation of RNA biosynthetic process | 76 | 1.95E-04 | 2.95E-02 |
| ribose phosphate biosynthetic process | 18 | 2.03E-04 | 2.97E-02 |
| regulation of DNA-templated transcription | 76 | 2.35E-04 | 3.41E-02 |
| cytochrome complex assembly | 7 | 2.41E-04 | 3.43E-02 |
| rRNA processing | 18 | 2.40E-04 | 3.46E-02 |
| purine-containing compound biosynthetic process | 19 | 2.64E-04 | 3.73E-02 |
| regulation of activated T cell proliferation | 7 | 2.82E-04 | 3.91E-02 |
| response to lipid | 47 | 2.89E-04 | 3.94E-02 |
| tissue migration | 12 | 2.82E-04 | 3.94E-02 |
| regulation of inflammatory response | 26 | 2.87E-04 | 3.95E-02 |
| disruption of plasma membrane integrity in another organism | 3 | 3.07E-04 | 4.07E-02 |
| ERBB2-ERBB4 signaling pathway | 3 | 3.07E-04 | 4.10E-02 |
| disruption of cellular anatomical structure in another organism | 3 | 3.07E-04 | 4.14E-02 |
| cellular component biogenesis | 118 | 3.32E-04 | 4.32E-02 |
| ribose phosphate metabolic process | 28 | 3.31E-04 | 4.36E-02 |
| positive regulation of response to stimulus | 101 | 3.42E-04 | 4.38E-02 |
| cell motility | 56 | 3.41E-04 | 4.41E-02 |
| detection of chemical stimulus | 4 | 3.49E-04 | 4.44E-02 |
| regulation of immune system process | 74 | 3.70E-04 | 4.67E-02 |
| positive regulation of signal transduction by p53 class mediator | 6 | 3.81E-04 | 4.76E-02 |
| nucleoside triphosphate metabolic process | 17 | 4.00E-04 | 4.96E-02 |
| erythrocyte differentiation | 11 | 4.06E-04 | 4.99E-02 |
| *Molecular functions* | | | |
| structural constituent of ribosome | 48 | 2.29E-32 | 1.16E-28 |
| structural molecule activity | 70 | 1.27E-14 | 3.22E-11 |
| ubiquitin ligase inhibitor activity | 5 | 3.66E-06 | 6.19E-03 |
| NAD(P)H dehydrogenase (quinone) activity | 9 | 1.02E-05 | 1.29E-02 |
| olfactory receptor activity | 1 | 1.42E-05 | 1.44E-02 |
| ubiquitin-protein transferase inhibitor activity | 5 | 2.12E-05 | 1.80E-02 |
| oxidoreduction-driven active transmembrane transporter activity | 10 | 4.85E-05 | 2.73E-02 |
| NADH dehydrogenase (ubiquinone) activity | 8 | 3.83E-05 | 2.77E-02 |
| NADH dehydrogenase (quinone) activity | 8 | 4.60E-05 | 2.92E-02 |
| NADH dehydrogenase activity | 8 | 6.53E-05 | 3.31E-02 |
| oxidoreductase activity, acting on NAD(P)H, quinone or similar compound as acceptor | 9 | 7.33E-05 | 3.38E-02 |
| *Cellular components* | | | |
| cytosolic ribosome | 40 | 1.90E-29 | 3.80E-26 |
| ribosomal subunit | 47 | 5.52E-28 | 5.52E-25 |
| ribosome | 50 | 5.71E-26 | 3.80E-23 |
| cytosolic large ribosomal subunit | 21 | 6.72E-17 | 3.36E-14 |
| cytosolic small ribosomal subunit | 19 | 8.53E-17 | 3.41E-14 |
| small ribosomal subunit | 22 | 7.04E-15 | 2.34E-12 |
| large ribosomal subunit | 25 | 3.92E-14 | 1.12E-11 |
| ribonucleoprotein complex | 61 | 2.51E-12 | 6.26E-10 |
| extracellular space | 172 | 3.24E-11 | 7.19E-09 |
| specific granule | 25 | 4.82E-11 | 9.64E-09 |
| respiratory chain complex | 18 | 7.10E-10 | 1.29E-07 |
| mitochondrial protein-containing complex | 33 | 8.99E-10 | 1.50E-07 |
| inner mitochondrial membrane protein complex | 23 | 1.76E-09 | 2.70E-07 |
| mitochondrial inner membrane | 43 | 4.25E-09 | 6.07E-07 |
| respirasome | 18 | 4.67E-09 | 6.22E-07 |
| extracellular region | 199 | 7.13E-09 | 8.39E-07 |
| specific granule lumen | 14 | 6.84E-09 | 8.55E-07 |
| organelle inner membrane | 45 | 1.32E-08 | 1.46E-06 |
| extracellular membrane-bounded organelle | 114 | 2.27E-08 | 2.16E-06 |
| extracellular organelle | 114 | 2.27E-08 | 2.26E-06 |
| extracellular vesicle | 114 | 2.24E-08 | 2.36E-06 |
| extracellular exosome | 112 | 4.16E-08 | 3.78E-06 |
| cytochrome complex | 11 | 5.49E-08 | 4.78E-06 |
| mitochondrial respirasome | 16 | 5.83E-08 | 4.85E-06 |
| vesicle | 183 | 1.23E-07 | 9.86E-06 |
| mitochondrial membrane | 53 | 1.82E-07 | 1.40E-05 |
| cytoplasmic vesicle lumen | 30 | 2.18E-07 | 1.62E-05 |
| mitochondrial envelope | 55 | 2.36E-07 | 1.63E-05 |
| vesicle lumen | 30 | 2.33E-07 | 1.67E-05 |
| tertiary granule | 19 | 1.26E-06 | 8.42E-05 |
| secretory granule lumen | 28 | 1.61E-06 | 1.04E-04 |
| cell periphery | 262 | 2.36E-06 | 1.43E-04 |
| secretory granule | 56 | 2.35E-06 | 1.47E-04 |
| membrane | 376 | 3.91E-06 | 2.30E-04 |
| tertiary granule lumen | 10 | 8.06E-06 | 4.60E-04 |
| organelle envelope | 70 | 1.24E-05 | 6.72E-04 |
| protein-containing complex | 260 | 1.31E-05 | 6.87E-04 |
| envelope | 70 | 1.24E-05 | 6.91E-04 |
| respiratory chain complex IV | 7 | 1.64E-05 | 8.42E-04 |
| cellular anatomical entity | 627 | 1.73E-05 | 8.63E-04 |
| small-subunit processome | 11 | 2.13E-05 | 1.04E-03 |
| complement component C1q complex | 3 | 3.22E-05 | 1.50E-03 |
| complement component C1 complex | 3 | 3.22E-05 | 1.53E-03 |
| mitochondrial proton-transporting ATP synthase complex | 6 | 4.93E-05 | 2.24E-03 |
| cellular_component | 629 | 5.71E-05 | 2.48E-03 |
| Unclassified | 27 | 5.71E-05 | 2.54E-03 |
| proton-transporting ATP synthase complex | 6 | 6.49E-05 | 2.76E-03 |
| plasma membrane | 236 | 7.98E-05 | 3.32E-03 |
| secretory vesicle | 58 | 1.16E-04 | 4.75E-03 |
| cell junction | 103 | 1.25E-04 | 4.99E-03 |
| cytoplasm | 435 | 1.45E-04 | 5.69E-03 |
| mitochondrial respiratory chain complex I | 8 | 1.93E-04 | 7.14E-03 |
| NADH dehydrogenase complex | 8 | 1.93E-04 | 7.28E-03 |
| respiratory chain complex I | 8 | 1.93E-04 | 7.42E-03 |
| preribosome | 12 | 2.19E-04 | 7.98E-03 |
| U4 snRNP | 4 | 2.82E-04 | 9.88E-03 |
| mitochondrial proton-transporting ATP synthase complex, coupling factor F(o) | 4 | 2.82E-04 | 1.01E-02 |
| phagocytic vesicle lumen | 3 | 3.07E-04 | 1.06E-02 |
| Golgi membrane | 7 | 3.32E-04 | 1.13E-02 |
| synapse | 72 | 3.85E-04 | 1.28E-02 |
| methylosome | 4 | 5.80E-04 | 1.90E-02 |
| pICln-Sm protein complex | 3 | 5.99E-04 | 1.93E-02 |
| specific granule membrane | 10 | 6.34E-04 | 2.01E-02 |
| striated muscle thin filament | 5 | 6.75E-04 | 2.11E-02 |
| non-membrane-bounded organelle | 208 | 8.06E-04 | 2.41E-02 |
| proton-transporting ATP synthase complex, coupling factor F(o) | 4 | 7.92E-04 | 2.43E-02 |
| intracellular non-membrane-bounded organelle | 208 | 8.04E-04 | 2.44E-02 |
| mitochondrial respiratory chain complex IV | 5 | 8.30E-04 | 2.44E-02 |
| vacuolar membrane | 4 | 9.31E-04 | 2.66E-02 |
| external side of plasma membrane | 26 | 9.18E-04 | 2.66E-02 |
| mitochondrial respiratory chain complex III | 4 | 1.05E-03 | 2.70E-02 |
| cytolytic granule lumen | 2 | 1.01E-03 | 2.70E-02 |
| cell surface | 48 | 9.96E-04 | 2.73E-02 |
| respiratory chain complex III | 4 | 1.05E-03 | 2.73E-02 |
| calprotectin complex | 2 | 1.01E-03 | 2.74E-02 |
| immunoglobulin complex | 15 | 1.05E-03 | 2.75E-02 |
| oxidoreductase complex | 12 | 9.94E-04 | 2.76E-02 |
| organelle | 488 | 9.87E-04 | 2.78E-02 |
| cell-substrate junction | 27 | 1.19E-03 | 3.02E-02 |
| mitochondrion | 77 | 1.41E-03 | 3.51E-02 |
| myofilament | 5 | 1.46E-03 | 3.60E-02 |
| proteasome core complex, alpha-subunit complex | 3 | 1.60E-03 | 3.90E-02 |
| focal adhesion | 26 | 1.73E-03 | 4.16E-02 |
| anchoring junction | 46 | 1.83E-03 | 4.35E-02 |
| ***MDD CRP 1-3 mg/L vs. MDD CRP<1*** | | | |
| Biological processes | | | |
| response to external biotic stimulus | 84 | 1.39E-14 | 4.25E-11 |
| defense response to symbiont | 65 | 1.69E-14 | 4.30E-11 |
| immune response | 95 | 1.31E-14 | 5.00E-11 |
| response to biotic stimulus | 85 | 3.09E-14 | 5.89E-11 |
| defense response to other organism | 69 | 2.77E-14 | 6.04E-11 |
| response to other organism | 84 | 1.24E-14 | 6.29E-11 |
| innate immune response | 59 | 1.06E-14 | 8.06E-11 |
| defense response to virus | 34 | 7.97E-15 | 1.21E-10 |
| biological process involved in interspecies interaction between organisms | 89 | 9.61E-14 | 1.63E-10 |
| immune system process | 114 | 4.31E-13 | 5.47E-10 |
| response to external stimulus | 113 | 3.67E-13 | 5.59E-10 |
| defense response | 83 | 4.21E-13 | 5.84E-10 |
| cytoplasmic translation | 22 | 7.46E-13 | 8.75E-10 |
| negative regulation of viral genome replication | 15 | 3.07E-12 | 3.35E-09 |
| response to virus | 36 | 3.43E-12 | 3.48E-09 |
| negative regulation of viral process | 17 | 1.90E-10 | 1.81E-07 |
| response to stress | 141 | 2.30E-10 | 2.06E-07 |
| regulation of viral genome replication | 15 | 2.35E-09 | 1.99E-06 |
| regulation of defense response | 49 | 2.87E-09 | 2.30E-06 |
| regulation of response to biotic stimulus | 37 | 9.11E-09 | 6.94E-06 |
| regulation of viral life cycle | 18 | 2.03E-08 | 1.47E-05 |
| regulation of immune response | 52 | 2.46E-08 | 1.71E-05 |
| antiviral innate immune response | 11 | 3.68E-08 | 2.44E-05 |
| regulation of innate immune response | 32 | 3.88E-08 | 2.47E-05 |
| regulation of viral process | 19 | 4.20E-08 | 2.56E-05 |
| regulation of response to external stimulus | 58 | 6.03E-08 | 3.54E-05 |
| interleukin-27-mediated signaling pathway | 5 | 1.82E-07 | 1.03E-04 |
| response to cytokine | 46 | 2.62E-07 | 1.42E-04 |
| response to stimulus | 258 | 4.20E-07 | 2.21E-04 |
| response to bacterium | 40 | 6.02E-07 | 3.06E-04 |
| response to type I interferon | 10 | 7.03E-07 | 3.46E-04 |
| translation | 28 | 7.47E-07 | 3.56E-04 |
| peptide biosynthetic process | 28 | 2.96E-06 | 1.33E-03 |
| regulation of response to stress | 62 | 2.92E-06 | 1.35E-03 |
| negative regulation of innate immune response | 11 | 9.72E-06 | 4.23E-03 |
| positive regulation of mitotic cell cycle | 13 | 1.04E-05 | 4.41E-03 |
| regulation of immune system process | 66 | 1.12E-05 | 4.62E-03 |
| positive regulation of defense response | 26 | 1.37E-05 | 5.48E-03 |
| cellular response to cytokine stimulus | 38 | 1.46E-05 | 5.71E-03 |
| cellular response to type I interferon | 8 | 1.51E-05 | 5.74E-03 |
| mitotic nuclear division | 15 | 1.67E-05 | 6.06E-03 |
| response to interferon-beta | 7 | 1.63E-05 | 6.06E-03 |
| nuclear division | 22 | 1.79E-05 | 6.35E-03 |
| positive regulation of immune response | 35 | 2.68E-05 | 9.27E-03 |
| negative regulation of response to biotic stimulus | 12 | 3.33E-05 | 1.13E-02 |
| positive regulation of response to stimulus | 86 | 3.58E-05 | 1.19E-02 |
| activation of immune response | 24 | 3.86E-05 | 1.25E-02 |
| regulation of ribonuclease activity | 4 | 4.21E-05 | 1.34E-02 |
| negative regulation of biological process | 169 | 5.39E-05 | 1.68E-02 |
| regulation of molecular function | 78 | 5.52E-05 | 1.68E-02 |
| MDA-5 signaling pathway | 3 | 5.90E-05 | 1.73E-02 |
| organelle fission | 22 | 5.81E-05 | 1.74E-02 |
| regulation of response to stimulus | 135 | 6.55E-05 | 1.85E-02 |
| positive regulation of response to external stimulus | 30 | 6.50E-05 | 1.87E-02 |
| peptide metabolic process | 30 | 7.11E-05 | 1.97E-02 |
| positive regulation of chromosome separation | 6 | 7.94E-05 | 2.16E-02 |
| regulation of cytokine production | 38 | 8.29E-05 | 2.22E-02 |
| regulation of type I interferon-mediated signaling pathway | 7 | 9.39E-05 | 2.47E-02 |
| response to organic substance | 91 | 9.96E-05 | 2.57E-02 |
| response to interferon-alpha | 5 | 1.03E-04 | 2.62E-02 |
| regulation of cell cycle phase transition | 25 | 1.05E-04 | 2.63E-02 |
| type I interferon-mediated signaling pathway | 7 | 1.09E-04 | 2.67E-02 |
| amide biosynthetic process | 29 | 1.24E-04 | 3.01E-02 |
| mitotic sister chromatid segregation | 12 | 1.90E-04 | 4.53E-02 |
| negative regulation of type I interferon-mediated signaling pathway | 5 | 2.11E-04 | 4.94E-02 |
| *Molecular functions* | | | |
| structural constituent of ribosome | 24 | 4.28E-12 | 2.17E-08 |
| structural molecule activity | 45 | 2.67E-07 | 6.78E-04 |
| 2'-5'-oligoadenylate synthetase activity | 3 | 1.50E-05 | 2.54E-02 |
| *Cellular components* | | | |
| cytosolic ribosome | 22 | 3.81E-13 | 7.62E-10 |
| ribosome | 28 | 2.35E-11 | 2.34E-08 |
| ribosomal subunit | 24 | 1.18E-10 | 7.86E-08 |
| cytosolic large ribosomal subunit | 12 | 2.18E-08 | 1.09E-05 |
| cytosolic small ribosomal subunit | 10 | 1.78E-07 | 7.10E-05 |
| small ribosomal subunit | 11 | 5.43E-06 | 1.81E-03 |
| large ribosomal subunit | 13 | 7.22E-06 | 2.06E-03 |
| ***MDD CRP>3 mg/L vs. MDD CRP<1 mg/L*** | | | |
| cytoplasmic translation | 43 | 3.67E-26 | 5.60E-22 |
| immune system process | 190 | 1.29E-16 | 9.86E-13 |
| oxidative phosphorylation | 31 | 1.40E-15 | 7.12E-12 |
| immune response | 145 | 1.85E-14 | 7.07E-11 |
| translation | 54 | 4.86E-13 | 1.48E-09 |
| response to external stimulus | 176 | 1.20E-12 | 3.04E-09 |
| peptide biosynthetic process | 54 | 8.15E-12 | 1.77E-08 |
| aerobic respiration | 31 | 1.13E-11 | 2.16E-08 |
| ATP synthesis coupled electron transport | 23 | 1.72E-11 | 2.62E-08 |
| mitochondrial ATP synthesis coupled electron transport | 23 | 1.72E-11 | 2.92E-08 |
| aerobic electron transport chain | 22 | 3.76E-11 | 5.21E-08 |
| cellular respiration | 33 | 5.58E-11 | 5.67E-08 |
| peptide metabolic process | 62 | 6.16E-11 | 5.87E-08 |
| Unclassified | 64 | 5.42E-11 | 5.90E-08 |
| respiratory electron transport chain | 25 | 4.79E-11 | 6.08E-08 |
| biological_process | 869 | 5.42E-11 | 6.35E-08 |
| organonitrogen compound biosynthetic process | 114 | 1.33E-10 | 1.20E-07 |
| amide biosynthetic process | 60 | 2.51E-10 | 2.13E-07 |
| defense response | 118 | 2.89E-10 | 2.32E-07 |
| cellular process | 758 | 3.91E-10 | 2.98E-07 |
| response to stimulus | 460 | 1.51E-09 | 1.10E-06 |
| response to other organism | 112 | 1.65E-09 | 1.14E-06 |
| response to external biotic stimulus | 112 | 1.73E-09 | 1.15E-06 |
| nucleoside triphosphate biosynthetic process | 22 | 3.06E-09 | 1.79E-06 |
| response to biotic stimulus | 114 | 2.98E-09 | 1.82E-06 |
| proton motive force-driven ATP synthesis | 18 | 2.87E-09 | 1.82E-06 |
| energy derivation by oxidation of organic compounds | 36 | 4.02E-09 | 2.27E-06 |
| ATP biosynthetic process | 19 | 4.96E-09 | 2.70E-06 |
| regulation of transcription by RNA polymerase II | 64 | 7.39E-09 | 3.63E-06 |
| regulation of nucleobase-containing compound metabolic process | 118 | 6.96E-09 | 3.66E-06 |
| purine ribonucleoside triphosphate biosynthetic process | 20 | 7.39E-09 | 3.76E-06 |
| response to stress | 222 | 9.20E-09 | 4.25E-06 |
| purine nucleoside triphosphate biosynthetic process | 20 | 8.94E-09 | 4.26E-06 |
| defense response to other organism | 87 | 1.21E-08 | 5.41E-06 |
| positive regulation of nucleobase-containing compound metabolic process | 46 | 1.34E-08 | 5.85E-06 |
| proton motive force-driven mitochondrial ATP synthesis | 16 | 1.75E-08 | 7.42E-06 |
| defense response to symbiont | 80 | 2.00E-08 | 8.22E-06 |
| ribonucleoside triphosphate biosynthetic process | 20 | 2.23E-08 | 8.95E-06 |
| electron transport chain | 26 | 3.59E-08 | 1.40E-05 |
| chemotaxis | 40 | 5.31E-08 | 2.02E-05 |
| mucosal immune response | 11 | 5.73E-08 | 2.13E-05 |
| taxis | 40 | 6.22E-08 | 2.26E-05 |
| defense response to bacterium | 37 | 6.72E-08 | 2.38E-05 |
| antimicrobial humoral response | 24 | 7.03E-08 | 2.43E-05 |
| regulation of RNA metabolic process | 111 | 8.60E-08 | 2.91E-05 |
| cellular nitrogen compound biosynthetic process | 115 | 9.71E-08 | 3.22E-05 |
| amide metabolic process | 72 | 1.28E-07 | 4.14E-05 |
| biological process involved in interspecies interaction between organisms | 115 | 1.64E-07 | 5.22E-05 |
| antimicrobial humoral immune response mediated by antimicrobial peptide | 19 | 1.92E-07 | 5.84E-05 |
| purine ribonucleotide biosynthetic process | 27 | 1.90E-07 | 5.92E-05 |
| innate immune response in mucosa | 9 | 2.26E-07 | 6.75E-05 |
| organ or tissue specific immune response | 11 | 2.39E-07 | 7.00E-05 |
| positive regulation of response to stimulus | 152 | 2.85E-07 | 8.19E-05 |
| positive regulation of RNA metabolic process | 43 | 2.95E-07 | 8.32E-05 |
| innate immune response | 67 | 3.17E-07 | 8.78E-05 |
| locomotion | 40 | 3.34E-07 | 9.10E-05 |
| inflammatory response | 53 | 4.66E-07 | 1.25E-04 |
| ribose phosphate biosynthetic process | 28 | 5.63E-07 | 1.45E-04 |
| response to bacterium | 61 | 5.62E-07 | 1.48E-04 |
| nucleotide biosynthetic process | 32 | 6.39E-07 | 1.60E-04 |
| purine nucleotide biosynthetic process | 29 | 6.38E-07 | 1.62E-04 |
| nucleoside phosphate biosynthetic process | 32 | 7.54E-07 | 1.85E-04 |
| humoral immune response | 31 | 7.67E-07 | 1.86E-04 |
| ribonucleotide biosynthetic process | 27 | 9.55E-07 | 2.24E-04 |
| regulation of RNA biosynthetic process | 104 | 9.54E-07 | 2.27E-04 |
| antibacterial humoral response | 14 | 1.20E-06 | 2.76E-04 |
| regulation of DNA-templated transcription | 104 | 1.42E-06 | 3.24E-04 |
| regulation of immune system process | 110 | 1.56E-06 | 3.45E-04 |
| purine-containing compound biosynthetic process | 29 | 1.54E-06 | 3.45E-04 |
| chromatin organization | 11 | 2.19E-06 | 4.76E-04 |
| purine nucleoside triphosphate metabolic process | 25 | 2.27E-06 | 4.88E-04 |
| purine ribonucleotide metabolic process | 39 | 2.62E-06 | 5.54E-04 |
| detection of chemical stimulus involved in sensory perception | 4 | 2.74E-06 | 5.64E-04 |
| organonitrogen compound metabolic process | 272 | 2.71E-06 | 5.66E-04 |
| nucleoside triphosphate metabolic process | 26 | 3.08E-06 | 6.26E-04 |
| response to cytokine | 67 | 3.25E-06 | 6.51E-04 |
| detection of chemical stimulus | 5 | 3.58E-06 | 7.10E-04 |
| purine ribonucleoside triphosphate metabolic process | 24 | 3.86E-06 | 7.54E-04 |
| ATP metabolic process | 22 | 4.18E-06 | 7.86E-04 |
| detection of chemical stimulus involved in sensory perception of smell | 3 | 4.15E-06 | 7.91E-04 |
| ribose phosphate metabolic process | 41 | 4.13E-06 | 7.96E-04 |
| regulation of inflammatory response | 38 | 4.85E-06 | 8.40E-04 |
| positive regulation of cell communication | 120 | 4.55E-06 | 8.46E-04 |
| mitochondrial electron transport, NADH to ubiquinone | 11 | 4.83E-06 | 8.47E-04 |
| ribosomal small subunit biogenesis | 17 | 4.73E-06 | 8.49E-04 |
| positive regulation of chromosome separation | 9 | 4.68E-06 | 8.50E-04 |
| positive regulation of signaling | 120 | 4.65E-06 | 8.53E-04 |
| ribonucleotide metabolic process | 40 | 4.82E-06 | 8.55E-04 |
| cell chemotaxis | 26 | 5.21E-06 | 8.92E-04 |
| regulation of response to stimulus | 236 | 5.34E-06 | 9.05E-04 |
| regulation of cytokine production | 64 | 6.13E-06 | 1.03E-03 |
| mitochondrial electron transport, cytochrome c to oxygen | 8 | 6.63E-06 | 1.10E-03 |
| positive regulation of inflammatory response | 20 | 7.20E-06 | 1.18E-03 |
| ribonucleoside triphosphate metabolic process | 24 | 7.35E-06 | 1.19E-03 |
| chromatin remodeling | 8 | 8.19E-06 | 1.31E-03 |
| sensory perception of chemical stimulus | 6 | 8.67E-06 | 1.35E-03 |
| positive regulation of RNA biosynthetic process | 43 | 8.59E-06 | 1.35E-03 |
| positive regulation of DNA-templated transcription | 43 | 8.55E-06 | 1.36E-03 |
| defense response to fungus | 12 | 9.05E-06 | 1.39E-03 |
| positive regulation of transcription by RNA polymerase II | 28 | 9.99E-06 | 1.52E-03 |
| erythrocyte homeostasis | 17 | 1.02E-05 | 1.54E-03 |
| positive regulation of signal transduction | 108 | 1.17E-05 | 1.75E-03 |
| generation of precursor metabolites and energy | 39 | 1.86E-05 | 2.73E-03 |
| signaling | 288 | 1.91E-05 | 2.75E-03 |
| regulation of signaling | 205 | 1.86E-05 | 2.76E-03 |
| regulation of cell communication | 205 | 1.91E-05 | 2.77E-03 |
| ERBB2-ERBB4 signaling pathway | 4 | 2.02E-05 | 2.88E-03 |
| cell killing | 21 | 2.07E-05 | 2.92E-03 |
| purine nucleotide metabolic process | 42 | 2.31E-05 | 3.20E-03 |
| cell communication | 295 | 2.31E-05 | 3.22E-03 |
| nucleotide metabolic process | 46 | 2.73E-05 | 3.74E-03 |
| nucleoside phosphate metabolic process | 46 | 3.39E-05 | 4.53E-03 |
| regulation of Notch signaling pathway | 15 | 3.37E-05 | 4.54E-03 |
| protein-DNA complex organization | 16 | 3.34E-05 | 4.55E-03 |
| cellular response to cytokine stimulus | 58 | 3.62E-05 | 4.80E-03 |
| regulation of signal transduction | 182 | 3.66E-05 | 4.81E-03 |
| sensory perception of smell | 5 | 3.95E-05 | 5.15E-03 |
| regulation of immune response | 69 | 4.20E-05 | 5.43E-03 |
| signal transduction | 270 | 4.28E-05 | 5.48E-03 |
| killing of cells of another organism | 16 | 4.45E-05 | 5.61E-03 |
| disruption of cell in another organism | 16 | 4.45E-05 | 5.66E-03 |
| immune response-regulating signaling pathway | 34 | 4.62E-05 | 5.77E-03 |
| positive regulation of Notch signaling pathway | 10 | 4.73E-05 | 5.86E-03 |
| regulation of response to external stimulus | 79 | 5.02E-05 | 6.17E-03 |
| mitotic cell cycle process | 44 | 5.10E-05 | 6.22E-03 |
| response to molecule of bacterial origin | 33 | 5.20E-05 | 6.29E-03 |
| mitochondrial respiratory chain complex assembly | 15 | 5.43E-05 | 6.52E-03 |
| response to fungus | 12 | 5.66E-05 | 6.74E-03 |
| regulation of multicellular organismal process | 178 | 5.90E-05 | 6.97E-03 |
| disruption of anatomical structure in another organism | 16 | 6.18E-05 | 7.24E-03 |
| myeloid cell homeostasis | 18 | 6.38E-05 | 7.42E-03 |
| positive regulation of kinase activity | 30 | 6.72E-05 | 7.76E-03 |
| positive regulation of multicellular organismal process | 109 | 7.34E-05 | 8.35E-03 |
| regulation of defense response | 60 | 7.32E-05 | 8.39E-03 |
| myeloid leukocyte activation | 19 | 7.79E-05 | 8.79E-03 |
| sister chromatid segregation | 18 | 9.23E-05 | 1.03E-02 |
| nucleobase-containing small molecule metabolic process | 49 | 9.68E-05 | 1.08E-02 |
| defense response to Gram-positive bacterium | 16 | 1.04E-04 | 1.14E-02 |
| cellular response to chemical stimulus | 147 | 1.03E-04 | 1.14E-02 |
| response to lipopolysaccharide | 31 | 1.06E-04 | 1.15E-02 |
| purine-containing compound metabolic process | 42 | 1.12E-04 | 1.21E-02 |
| protein-containing complex assembly | 87 | 1.18E-04 | 1.26E-02 |
| immune response-regulating cell surface receptor signaling pathway | 28 | 1.21E-04 | 1.29E-02 |
| cellular response to stimulus | 346 | 1.26E-04 | 1.34E-02 |
| positive regulation of acute inflammatory response | 7 | 1.53E-04 | 1.61E-02 |
| positive regulation of response to external stimulus | 45 | 1.57E-04 | 1.63E-02 |
| response to organic substance | 150 | 1.59E-04 | 1.64E-02 |
| cell motility | 76 | 1.57E-04 | 1.64E-02 |
| mitotic nuclear division | 19 | 1.94E-04 | 1.97E-02 |
| detection of stimulus involved in sensory perception | 9 | 1.93E-04 | 1.98E-02 |
| erythrocyte differentiation | 14 | 2.02E-04 | 2.04E-02 |
| mitotic sister chromatid segregation | 17 | 2.04E-04 | 2.05E-02 |
| cell migration | 65 | 2.09E-04 | 2.09E-02 |
| homeostasis of number of cells | 27 | 2.42E-04 | 2.40E-02 |
| organophosphate biosynthetic process | 44 | 2.55E-04 | 2.46E-02 |
| 7-methylguanosine cap hypermethylation | 4 | 2.53E-04 | 2.46E-02 |
| positive regulation of cytokine production | 41 | 2.51E-04 | 2.46E-02 |
| ERBB4 signaling pathway | 4 | 2.53E-04 | 2.47E-02 |
| hemopoiesis | 53 | 2.72E-04 | 2.61E-02 |
| cell activation | 55 | 2.77E-04 | 2.64E-02 |
| positive regulation of immune system process | 70 | 3.10E-04 | 2.94E-02 |
| defense response to Gram-negative bacterium | 13 | 3.13E-04 | 2.95E-02 |
| regulation of tumor necrosis factor superfamily cytokine production | 19 | 3.31E-04 | 3.10E-02 |
| positive regulation of interleukin-1 beta production | 10 | 3.34E-04 | 3.10E-02 |
| negative regulation of toll-like receptor 9 signaling pathway | 3 | 3.58E-04 | 3.31E-02 |
| positive regulation of transferase activity | 33 | 3.63E-04 | 3.34E-02 |
| positive regulation of protein kinase activity | 25 | 3.67E-04 | 3.35E-02 |
| regulation of kinase activity | 42 | 4.05E-04 | 3.67E-02 |
| positive regulation of defense response | 35 | 4.26E-04 | 3.84E-02 |
| mitochondrial respiratory chain complex I assembly | 10 | 4.38E-04 | 3.91E-02 |
| NADH dehydrogenase complex assembly | 10 | 4.38E-04 | 3.93E-02 |
| regulation of chromosome separation | 11 | 4.76E-04 | 4.22E-02 |
| leukocyte activation | 47 | 4.82E-04 | 4.25E-02 |
| organophosphate metabolic process | 66 | 5.21E-04 | 4.57E-02 |
| RNA capping | 5 | 5.43E-04 | 4.70E-02 |
| cellular response to organic substance | 108 | 5.47E-04 | 4.71E-02 |
| cellular response to molecule of bacterial origin | 21 | 5.52E-04 | 4.73E-02 |
| modulation of process of another organism | 5 | 5.43E-04 | 4.73E-02 |
| carbohydrate derivative metabolic process | 69 | 5.85E-04 | 4.98E-02 |
| Molecular functions | | | |
| structural constituent of ribosome | 50 | 1.96E-27 | 9.95E-24 |
| structural molecule activity | 79 | 4.93E-11 | 1.25E-07 |
| RNA polymerase II transcription regulatory region sequence-specific DNA binding | 23 | 1.55E-09 | 2.61E-06 |
| protein binding | 735 | 2.08E-09 | 2.63E-06 |
| DNA-binding transcription factor activity | 27 | 1.16E-08 | 8.41E-06 |
| transcription regulatory region nucleic acid binding | 28 | 1.14E-08 | 9.67E-06 |
| transcription cis-regulatory region binding | 28 | 1.13E-08 | 1.15E-05 |
| sequence-specific DNA binding | 35 | 3.16E-08 | 1.78E-05 |
| sequence-specific double-stranded DNA binding | 31 | 3.11E-08 | 1.97E-05 |
| DNA-binding transcription factor activity, RNA polymerase II-specific | 26 | 8.95E-08 | 4.54E-05 |
| transcription regulator activity | 46 | 1.03E-07 | 4.76E-05 |
| RNA polymerase II cis-regulatory region sequence-specific DNA binding | 21 | 1.34E-07 | 5.67E-05 |
| double-stranded DNA binding | 36 | 1.47E-07 | 5.74E-05 |
| cis-regulatory region sequence-specific DNA binding | 22 | 1.76E-07 | 6.39E-05 |
| binding | 813 | 1.91E-07 | 6.46E-05 |
| Unclassified | 59 | 3.75E-07 | 1.12E-04 |
| molecular_function | 874 | 3.75E-07 | 1.19E-04 |
| DNA binding | 69 | 8.82E-07 | 2.48E-04 |
| immunoglobulin binding | 9 | 1.20E-06 | 3.21E-04 |
| oxidoreduction-driven active transmembrane transporter activity | 14 | 1.45E-06 | 3.67E-04 |
| olfactory receptor activity | 3 | 4.04E-06 | 9.76E-04 |
| immune receptor activity | 21 | 5.22E-06 | 1.20E-03 |
| ubiquitin-protein transferase inhibitor activity | 6 | 6.22E-06 | 1.37E-03 |
| proton-transporting ATP synthase activity, rotational mechanism | 7 | 7.86E-06 | 1.66E-03 |
| NADH dehydrogenase (ubiquinone) activity | 10 | 1.08E-05 | 2.19E-03 |
| NADH dehydrogenase (quinone) activity | 10 | 1.36E-05 | 2.65E-03 |
| immunoglobulin receptor activity | 6 | 1.87E-05 | 3.51E-03 |
| ubiquitin ligase inhibitor activity | 5 | 2.04E-05 | 3.57E-03 |
| NADH dehydrogenase activity | 10 | 2.11E-05 | 3.57E-03 |
| IgE binding | 4 | 2.02E-05 | 3.65E-03 |
| NAD(P)H dehydrogenase (quinone) activity | 10 | 2.61E-05 | 4.27E-03 |
| calcium ion binding | 57 | 4.98E-05 | 7.88E-03 |
| electron transfer activity | 16 | 6.87E-05 | 1.05E-02 |
| histone modifying activity | 0 | 1.42E-04 | 2.11E-02 |
| proton channel activity | 7 | 1.97E-04 | 2.85E-02 |
| oxidoreductase activity, acting on NAD(P)H, quinone or similar compound as acceptor | 10 | 2.16E-04 | 3.04E-02 |
| ubiquitin-protein transferase regulator activity | 7 | 3.12E-04 | 4.28E-02 |
| Toll-like receptor 4 binding | 3 | 3.58E-04 | 4.78E-02 |
| *Cellular components* | | | |
| cytosolic ribosome | 43 | 8.29E-27 | 1.66E-23 |
| ribosomal subunit | 49 | 3.54E-23 | 3.53E-20 |
| ribosome | 52 | 8.97E-21 | 5.98E-18 |
| specific granule | 38 | 1.90E-17 | 9.51E-15 |
| cytosolic large ribosomal subunit | 23 | 4.49E-16 | 1.79E-13 |
| cytosolic small ribosomal subunit | 20 | 3.33E-15 | 1.11E-12 |
| specific granule lumen | 21 | 1.52E-13 | 4.34E-11 |
| extracellular space | 233 | 1.71E-12 | 4.26E-10 |
| large ribosomal subunit | 27 | 2.23E-12 | 4.95E-10 |
| secretory granule | 89 | 4.33E-12 | 8.65E-10 |
| cell periphery | 390 | 5.41E-12 | 9.84E-10 |
| inner mitochondrial membrane protein complex | 31 | 6.85E-12 | 1.14E-09 |
| small ribosomal subunit | 22 | 8.17E-12 | 1.26E-09 |
| tertiary granule | 31 | 1.13E-11 | 1.41E-09 |
| secretory vesicle | 100 | 1.01E-11 | 1.45E-09 |
| membrane | 553 | 1.09E-11 | 1.45E-09 |
| extracellular region | 279 | 3.41E-11 | 4.01E-09 |
| respiratory chain complex | 21 | 8.02E-10 | 8.91E-08 |
| mitochondrial protein-containing complex | 40 | 1.43E-09 | 1.51E-07 |
| vesicle | 256 | 1.59E-09 | 1.59E-07 |
| cytoplasmic vesicle lumen | 41 | 3.46E-09 | 3.29E-07 |
| plasma membrane | 352 | 3.79E-09 | 3.30E-07 |
| vesicle lumen | 41 | 3.79E-09 | 3.44E-07 |
| extracellular membrane-bounded organelle | 153 | 5.82E-09 | 4.31E-07 |
| cellular anatomical entity | 896 | 5.35E-09 | 4.45E-07 |
| extracellular organelle | 153 | 5.82E-09 | 4.48E-07 |
| extracellular vesicle | 153 | 5.77E-09 | 4.61E-07 |
| respirasome | 21 | 6.90E-09 | 4.93E-07 |
| mitochondrial respirasome | 20 | 8.94E-09 | 6.17E-07 |
| mitochondrial inner membrane | 53 | 1.07E-08 | 7.15E-07 |
| tertiary granule lumen | 15 | 1.35E-08 | 8.45E-07 |
| extracellular exosome | 150 | 1.34E-08 | 8.65E-07 |
| ribonucleoprotein complex | 67 | 2.06E-08 | 1.25E-06 |
| secretory granule lumen | 39 | 2.18E-08 | 1.28E-06 |
| Unclassified | 35 | 5.44E-08 | 3.02E-06 |
| cellular_component | 898 | 5.44E-08 | 3.10E-06 |
| organelle inner membrane | 55 | 7.31E-08 | 3.95E-06 |
| mitochondrial membrane | 69 | 8.21E-08 | 4.32E-06 |
| mitochondrial envelope | 71 | 1.94E-07 | 9.95E-06 |
| cytochrome complex | 12 | 2.19E-07 | 1.09E-05 |
| cell surface | 77 | 2.30E-07 | 1.12E-05 |
| cytoplasm | 627 | 2.52E-07 | 1.20E-05 |
| side of membrane | 64 | 7.90E-07 | 3.67E-05 |
| protein-DNA complex | 30 | 8.42E-07 | 3.82E-05 |
| chromatin | 28 | 1.28E-06 | 5.67E-05 |
| specific granule membrane | 16 | 2.96E-06 | 1.29E-04 |
| mitochondrial proton-transporting ATP synthase complex | 8 | 3.12E-06 | 1.33E-04 |
| tertiary granule membrane | 14 | 4.25E-06 | 1.77E-04 |
| proton-transporting ATP synthase complex | 8 | 4.60E-06 | 1.88E-04 |
| cytoplasmic vesicle | 160 | 1.08E-05 | 4.33E-04 |
| intracellular vesicle | 160 | 1.39E-05 | 5.45E-04 |
| respiratory chain complex IV | 8 | 1.77E-05 | 6.81E-04 |
| proton-transporting ATP synthase complex, coupling factor F(o) | 6 | 1.87E-05 | 7.05E-04 |
| secretory granule membrane | 32 | 3.02E-05 | 1.12E-03 |
| external side of plasma membrane | 38 | 3.34E-05 | 1.21E-03 |
| mitochondrial respiratory chain complex IV | 7 | 6.73E-05 | 2.40E-03 |
| mitochondrial proton-transporting ATP synthase complex, coupling factor F(o) | 5 | 6.94E-05 | 2.44E-03 |
| mitochondrial respiratory chain complex I | 10 | 8.17E-05 | 2.59E-03 |
| anchoring junction | 67 | 7.77E-05 | 2.63E-03 |
| NADH dehydrogenase complex | 10 | 8.17E-05 | 2.63E-03 |
| transporter complex | 41 | 7.73E-05 | 2.66E-03 |
| respiratory chain complex I | 10 | 8.17E-05 | 2.68E-03 |
| cell junction | 140 | 8.14E-05 | 2.71E-03 |
| chromosome | 55 | 9.06E-05 | 2.83E-03 |
| Fc-epsilon receptor I complex | 3 | 9.27E-05 | 2.85E-03 |
| canonical inflammasome complex | 6 | 9.88E-05 | 2.99E-03 |
| small-subunit processome | 12 | 1.14E-04 | 3.42E-03 |
| transmembrane transporter complex | 39 | 1.17E-04 | 3.44E-03 |
| endomembrane system | 267 | 1.20E-04 | 3.47E-03 |
| cell-substrate junction | 37 | 2.39E-04 | 6.82E-03 |
| ankyrin-1 complex | 4 | 2.53E-04 | 7.13E-03 |
| oxidoreductase complex | 16 | 2.91E-04 | 8.07E-03 |
| focal adhesion | 36 | 3.22E-04 | 8.82E-03 |
| postsynaptic density | 29 | 3.39E-04 | 9.16E-03 |
| AIM2 inflammasome complex | 3 | 3.58E-04 | 9.43E-03 |
| IPAF inflammasome complex | 3 | 3.58E-04 | 9.55E-03 |
| mitochondrial respiratory chain complex III | 5 | 3.88E-04 | 9.94E-03 |
| respiratory chain complex III | 5 | 3.88E-04 | 1.01E-02 |
| extracellular matrix | 44 | 4.22E-04 | 1.07E-02 |
| external encapsulating structure | 44 | 4.31E-04 | 1.08E-02 |
| synapse | 96 | 4.44E-04 | 1.09E-02 |
| proton-transporting two-sector ATPase complex | 9 | 5.69E-04 | 1.39E-02 |
| membrane protein complex | 92 | 5.88E-04 | 1.42E-02 |
| asymmetric synapse | 29 | 7.89E-04 | 1.88E-02 |
| neuron to neuron synapse | 31 | 8.40E-04 | 1.98E-02 |
| phagocytic vesicle lumen | 3 | 8.66E-04 | 1.99E-02 |
| Fc receptor complex | 3 | 8.66E-04 | 2.01E-02 |
| proton-transporting two-sector ATPase complex, proton-transporting domain | 6 | 8.98E-04 | 2.04E-02 |
| peptidase inhibitor complex | 4 | 1.07E-03 | 2.38E-02 |
| U4 snRNP | 4 | 1.07E-03 | 2.40E-02 |
| organelle membrane | 210 | 1.15E-03 | 2.53E-02 |
| collagen-containing extracellular matrix | 34 | 1.42E-03 | 3.08E-02 |
| postsynaptic specialization | 29 | 1.52E-03 | 3.26E-02 |
| NLRP1 inflammasome complex | 3 | 1.67E-03 | 3.52E-02 |
| pICln-Sm protein complex | 3 | 1.67E-03 | 3.56E-02 |
| organelle envelope | 82 | 1.84E-03 | 3.79E-02 |
| envelope | 82 | 1.84E-03 | 3.83E-02 |
| cytolytic granule lumen | 2 | 2.05E-03 | 4.14E-02 |
| calprotectin complex | 2 | 2.05E-03 | 4.18E-02 |
| ficolin-1-rich granule | 18 | 2.10E-03 | 4.19E-02 |
| methylosome | 4 | 2.16E-03 | 4.27E-02 |
| transcription regulator complex | 10 | 2.46E-03 | 4.83E-02 |
| ***MDD CRP>3 mg/L vs. MDD CRP 1-3 mg/L*** | | |  |
| Biological processes | | | |
| immune system process | 121 | 5.97E-13 | 9.10E-09 |
| immune response | 95 | 2.95E-12 | 2.25E-08 |
| defense response | 82 | 1.19E-10 | 6.06E-07 |
| response to external biotic stimulus | 75 | 7.97E-09 | 2.43E-05 |
| response to other organism | 75 | 7.61E-09 | 2.90E-05 |
| erythrocyte differentiation | 16 | 1.43E-08 | 3.64E-05 |
| biological process involved in interspecies interaction between organisms | 80 | 2.13E-08 | 4.65E-05 |
| response to biotic stimulus | 75 | 2.55E-08 | 4.86E-05 |
| erythrocyte homeostasis | 16 | 4.92E-08 | 7.50E-05 |
| defense response to other organism | 59 | 4.51E-08 | 7.64E-05 |
| response to stimulus | 283 | 7.44E-08 | 1.03E-04 |
| response to external stimulus | 103 | 1.61E-07 | 2.05E-04 |
| defense response to symbiont | 53 | 1.83E-07 | 2.15E-04 |
| innate immune response | 46 | 5.60E-07 | 6.10E-04 |
| regulation of multicellular organismal process | 123 | 8.18E-07 | 8.32E-04 |
| myeloid cell homeostasis | 16 | 1.25E-06 | 1.19E-03 |
| regulation of viral life cycle | 16 | 2.00E-06 | 1.80E-03 |
| cellular response to chemical stimulus | 102 | 2.27E-06 | 1.92E-03 |
| multicellular organismal-level homeostasis | 38 | 2.57E-06 | 2.06E-03 |
| response to bacterium | 41 | 3.13E-06 | 2.39E-03 |
| circulatory system process | 32 | 9.90E-06 | 7.18E-03 |
| blood circulation | 28 | 1.08E-05 | 7.51E-03 |
| regulation of viral process | 16 | 1.45E-05 | 9.23E-03 |
| erythrocyte development | 8 | 1.41E-05 | 9.35E-03 |
| cell motility | 53 | 2.76E-05 | 1.69E-02 |
| homeostasis of number of cells | 21 | 2.95E-05 | 1.73E-02 |
| positive regulation of Notch signaling pathway | 8 | 3.91E-05 | 2.21E-02 |
| homeostatic process | 65 | 4.18E-05 | 2.27E-02 |
| myeloid cell development | 10 | 4.60E-05 | 2.42E-02 |
| positive regulation of multicellular organismal process | 72 | 4.92E-05 | 2.50E-02 |
| negative regulation of viral process | 11 | 5.59E-05 | 2.58E-02 |
| myeloid cell differentiation | 21 | 5.42E-05 | 2.58E-02 |
| signaling | 180 | 5.26E-05 | 2.59E-02 |
| RNA processing | 7 | 6.90E-05 | 3.09E-02 |
| positive regulation of defense response | 26 | 7.20E-05 | 3.13E-02 |
| nucleic acid metabolic process | 32 | 8.30E-05 | 3.52E-02 |
| protein-containing complex organization | 29 | 8.88E-05 | 3.56E-02 |
| inflammatory response | 32 | 8.85E-05 | 3.64E-02 |
| positive regulation of catalytic activity | 44 | 1.09E-04 | 4.15E-02 |
| negative regulation of viral genome replication | 8 | 1.07E-04 | 4.18E-02 |
| cell communication | 182 | 1.18E-04 | 4.39E-02 |
| positive regulation of erythrocyte differentiation | 6 | 1.29E-04 | 4.68E-02 |
| positive regulation of response to stimulus | 90 | 1.36E-04 | 4.80E-02 |
| *Cellular components* | | | |
| cell periphery | 250 | 9.20E-12 | 1.84E-08 |
| plasma membrane | 230 | 2.28E-10 | 2.28E-07 |
| membrane | 323 | 3.94E-06 | 2.62E-03 |
| specific granule | 16 | 7.25E-06 | 3.63E-03 |
| ankyrin-1 complex | 4 | 3.38E-05 | 1.35E-02 |
| specific granule lumen | 9 | 4.06E-05 | 1.35E-02 |
| cellular anatomical entity | 532 | 5.21E-05 | 1.49E-02 |
| secretory vesicle | 52 | 6.53E-05 | 1.63E-02 |
| nuclear protein-containing complex | 14 | 7.62E-05 | 1.69E-02 |
| secretory granule | 45 | 8.55E-05 | 1.71E-02 |
| haptoglobin-hemoglobin complex | 4 | 9.70E-05 | 1.76E-02 |
| sarcolemma | 13 | 1.19E-04 | 1.99E-02 |
| tertiary granule | 14 | 1.51E-04 | 2.01E-02 |
| hemoglobin complex | 4 | 1.49E-04 | 2.13E-02 |
| catalytic complex | 24 | 1.47E-04 | 2.25E-02 |
| ***MDD CRP<1 mg/L vs. (all) controls*** | | |  |
| *Biological processes* | | | |
| cell periphery | 163 | 3.83E-09 | 7.65E-06 |
| plasma membrane | 151 | 2.86E-08 | 2.86E-05 |
| ankyrin-1 complex | 4 | 5.63E-06 | 3.75E-03 |
| postsynaptic membrane | 16 | 3.05E-05 | 1.02E-02 |
| immunoglobulin complex | 13 | 2.30E-05 | 1.15E-02 |
| plasma membrane region | 43 | 2.92E-05 | 1.17E-02 |
| cell body | 24 | 4.52E-05 | 1.29E-02 |
| IgG immunoglobulin complex | 4 | 5.37E-05 | 1.34E-02 |
| membrane | 208 | 6.80E-05 | 1.51E-02 |
| postsynapse | 26 | 7.64E-05 | 1.53E-02 |
| postsynaptic density | 16 | 1.31E-04 | 2.39E-02 |
| blood microparticle | 10 | 1.83E-04 | 2.82E-02 |
| neuron to neuron synapse | 17 | 2.12E-04 | 2.82E-02 |
| asymmetric synapse | 16 | 2.29E-04 | 2.86E-02 |
| somatodendritic compartment | 30 | 2.03E-04 | 2.90E-02 |
| neuron projection | 42 | 1.79E-04 | 2.98E-02 |
| postsynaptic specialization membrane | 9 | 2.57E-04 | 3.03E-02 |
| monoatomic ion channel complex | 16 | 3.51E-04 | 3.19E-02 |
| plasma membrane bounded cell projection | 62 | 3.07E-04 | 3.23E-02 |
| cyclin A2-CDK1 complex | 2 | 2.93E-04 | 3.25E-02 |
| postsynaptic density membrane | 8 | 3.49E-04 | 3.32E-02 |
| postsynaptic specialization | 16 | 3.40E-04 | 3.39E-02 |
| cell projection | 64 | 4.12E-04 | 3.58E-02 |
| neuronal cell body | 20 | 5.66E-04 | 4.72E-02 |
| cell periphery | 163 | 3.83E-09 | 7.65E-06 |
| ***MDD CRP<1 mg/L vs. controls CRP<1*** | | |  |
| *Biological processes* | | | |
| immune system process | 107 | 2.44E-07 | 3.72E-03 |
| immune response | 82 | 9.68E-07 | 7.38E-03 |
| *Molecular functions* | | | |
| antigen binding | 22 | 5.39E-08 | 2.73E-04 |
| nucleic acid binding | 73 | 6.22E-06 | 1.58E-02 |
| RNA binding | 21 | 1.03E-05 | 1.74E-02 |
| *Cellular components* | | | |
| cell periphery | 254 | 2.34E-10 | 2.34E-07 |
| plasma membrane | 239 | 2.31E-10 | 4.61E-07 |
| immunoglobulin complex | 20 | 4.05E-07 | 2.70E-04 |
| IgG immunoglobulin complex | 6 | 7.20E-07 | 3.60E-04 |
| caveola | 11 | 2.40E-05 | 9.59E-03 |
| ribonucleoprotein complex | 5 | 7.65E-05 | 2.55E-02 |
| membrane | 328 | 1.06E-04 | 3.03E-02 |
| **Treatment-based** | | | |
| ***MDD unmedicated vs. controls*** | | | |
| *Biological processes* | | | |
| negative regulation of viral genome replication | 15 | 2.06E-12 | 3.14E-08 |
| immune system process | 106 | 4.57E-11 | 1.39E-07 |
| cytoplasmic translation | 20 | 3.08E-11 | 1.57E-07 |
| defense response to virus | 29 | 2.15E-11 | 1.64E-07 |
| regulation of viral life cycle | 21 | 4.35E-11 | 1.66E-07 |
| negative regulation of viral process | 17 | 1.23E-10 | 3.13E-07 |
| regulation of viral process | 21 | 7.98E-10 | 1.74E-06 |
| immune response | 81 | 1.10E-09 | 2.09E-06 |
| regulation of viral genome replication | 15 | 1.61E-09 | 2.73E-06 |
| response to virus | 31 | 2.28E-09 | 3.47E-06 |
| translation | 32 | 2.65E-09 | 3.68E-06 |
| peptide biosynthetic process | 33 | 4.03E-09 | 5.11E-06 |
| peptide metabolic process | 36 | 8.19E-08 | 9.60E-05 |
| organonitrogen compound biosynthetic process | 65 | 1.10E-07 | 1.19E-04 |
| amide biosynthetic process | 35 | 1.73E-07 | 1.76E-04 |
| defense response to symbiont | 49 | 1.88E-07 | 1.79E-04 |
| innate immune response | 43 | 3.85E-07 | 3.45E-04 |
| defense response to other organism | 51 | 8.79E-07 | 7.45E-04 |
| antiviral innate immune response | 9 | 3.09E-06 | 2.48E-03 |
| organonitrogen compound metabolic process | 156 | 4.64E-06 | 3.54E-03 |
| response to biotic stimulus | 62 | 5.99E-06 | 4.35E-03 |
| amide metabolic process | 42 | 6.79E-06 | 4.70E-03 |
| adaptive immune response | 36 | 1.02E-05 | 5.98E-03 |
| interleukin-27-mediated signaling pathway | 4 | 1.09E-05 | 6.15E-03 |
| response to external biotic stimulus | 60 | 1.02E-05 | 6.19E-03 |
| regulation of nuclease activity | 6 | 1.01E-05 | 6.39E-03 |
| response to other organism | 60 | 9.90E-06 | 6.56E-03 |
| biological process involved in interspecies interaction between organisms | 64 | 2.19E-05 | 1.19E-02 |
| regulation of immune response | 43 | 3.34E-05 | 1.76E-02 |
| regulation of ribonuclease activity | 4 | 3.78E-05 | 1.92E-02 |
| response to type I interferon | 8 | 4.15E-05 | 2.04E-02 |
| immunoglobulin mediated immune response | 15 | 5.21E-05 | 2.48E-02 |
| MDA-5 signaling pathway | 3 | 5.42E-05 | 2.51E-02 |
| negative regulation of ubiquitin protein ligase activity | 4 | 6.17E-05 | 2.77E-02 |
| B cell mediated immunity | 15 | 7.02E-05 | 2.97E-02 |
| regulation of ubiquitin protein ligase activity | 5 | 6.92E-05 | 3.01E-02 |
| regulation of innate immune response | 25 | 7.68E-05 | 3.08E-02 |
| defense response | 59 | 7.54E-05 | 3.10E-02 |
| positive regulation of signal transduction by p53 class mediator | 6 | 8.27E-05 | 3.23E-02 |
| regulation of response to biotic stimulus | 28 | 1.04E-04 | 3.98E-02 |
| regulation of defense response | 37 | 1.10E-04 | 4.10E-02 |
| regulation of immune system process | 61 | 1.24E-04 | 4.50E-02 |
| *Molecular functions* | | | |
| structural constituent of ribosome | 28 | 5.95E-16 | 3.02E-12 |
| ubiquitin ligase inhibitor activity | 5 | 9.15E-07 | 2.32E-03 |
| ubiquitin-protein transferase inhibitor activity | 5 | 5.42E-06 | 9.16E-03 |
| structural molecule activity | 40 | 1.41E-05 | 1.43E-02 |
| mRNA 5'-UTR binding | 6 | 1.74E-05 | 1.47E-02 |
| 2'-5'-oligoadenylate synthetase activity | 3 | 1.38E-05 | 1.75E-02 |
| *Cellular components* | | | |
| ribosome | 33 | 1.42E-15 | 2.84E-12 |
| cytosolic ribosome | 23 | 2.27E-14 | 2.27E-11 |
| ribosomal subunit | 28 | 3.43E-14 | 2.28E-11 |
| cytosolic large ribosomal subunit | 12 | 1.60E-08 | 8.00E-06 |
| large ribosomal subunit | 16 | 2.39E-08 | 9.55E-06 |
| small ribosomal subunit | 12 | 5.87E-07 | 1.96E-04 |
| cytosolic small ribosomal subunit | 9 | 1.51E-06 | 4.31E-04 |
| mitochondrial inner membrane | 31 | 1.73E-06 | 4.32E-04 |
| organelle inner membrane | 31 | 1.67E-05 | 3.71E-03 |
| ribonucleoprotein complex | 36 | 2.95E-05 | 5.90E-03 |
| immunoglobulin complex | 15 | 5.21E-05 | 9.46E-03 |
| cellular anatomical entity | 474 | 1.06E-04 | 1.62E-02 |
| mitochondrial membrane | 37 | 9.78E-05 | 1.63E-02 |
| nuclear protein-containing complex | 12 | 1.39E-04 | 1.99E-02 |
| blood microparticle | 12 | 1.80E-04 | 1.99E-02 |
| mitochondrion | 65 | 1.70E-04 | 2.00E-02 |
| Unclassified | 19 | 1.61E-04 | 2.01E-02 |
| cellular_component | 476 | 1.61E-04 | 2.15E-02 |
| mitochondrial envelope | 37 | 2.68E-04 | 2.82E-02 |
| ***MDD responders vs. controls*** | | | |
| *Biological processes* | | | |
| response to bacterium | 36 | 4.31E-09 | 6.56E-05 |
| response to external biotic stimulus | 53 | 4.97E-08 | 2.52E-04 |
| response to biotic stimulus | 54 | 7.05E-08 | 2.69E-04 |
| response to other organism | 53 | 4.68E-08 | 3.57E-04 |
| immune response | 59 | 1.43E-07 | 4.36E-04 |
| biological process involved in interspecies interaction between organisms | 55 | 4.47E-07 | 8.52E-04 |
| adaptive immune response | 32 | 3.62E-07 | 9.21E-04 |
| immune system process | 73 | 4.30E-07 | 9.35E-04 |
| negative regulation of response to biotic stimulus | 12 | 9.19E-07 | 1.56E-03 |
| defense response to bacterium | 19 | 2.04E-06 | 3.11E-03 |
| response to stimulus | 186 | 2.41E-06 | 3.35E-03 |
| defense response | 50 | 2.83E-06 | 3.59E-03 |
| regulation of response to biotic stimulus | 25 | 4.67E-06 | 5.48E-03 |
| defense response to other organism | 38 | 1.06E-05 | 9.50E-03 |
| antibody-dependent cellular cytotoxicity | 4 | 1.04E-05 | 9.96E-03 |
| regulation of response to external stimulus | 40 | 1.22E-05 | 1.03E-02 |
| type IIa hypersensitivity | 4 | 1.04E-05 | 1.06E-02 |
| type II hypersensitivity | 4 | 1.04E-05 | 1.14E-02 |
| regulation of immune system process | 50 | 1.61E-05 | 1.29E-02 |
| regulation of defense response | 31 | 1.99E-05 | 1.52E-02 |
| regulation of cytokine-mediated signaling pathway | 12 | 2.37E-05 | 1.72E-02 |
| hypersensitivity | 4 | 3.94E-05 | 2.73E-02 |
| regulation of response to cytokine stimulus | 12 | 4.57E-05 | 3.03E-02 |
| response to external stimulus | 65 | 5.84E-05 | 3.71E-02 |
| positive regulation of cytokine-mediated signaling pathway | 7 | 6.98E-05 | 4.26E-02 |
| *Cellular components* | | | |
| immunoglobulin complex | 15 | 1.06E-06 | 2.13E-03 |
| cell periphery | 148 | 5.21E-05 | 2.08E-02 |
| extracellular region | 107 | 4.36E-05 | 2.18E-02 |
| blood microparticle | 11 | 4.10E-05 | 2.73E-02 |
| synaptic membrane | 19 | 8.98E-05 | 2.99E-02 |
| extracellular space | 88 | 3.22E-05 | 3.22E-02 |
| ***MDD non-responders vs. controls*** | | | |
| *Biological processes* | | | |
| response to stimulus | 203 | 1.63E-07 | 2.49E-03 |
| cellular process | 318 | 2.61E-06 | 1.33E-02 |
| cell surface receptor signaling pathway | 69 | 1.99E-06 | 1.52E-02 |
| response to oxygen-containing compound | 55 | 5.22E-06 | 1.99E-02 |
| response to lipid | 35 | 1.28E-05 | 3.24E-02 |
| positive regulation of cell killing | 9 | 1.18E-05 | 3.58E-02 |
| *Molecular functions* | | | |
| glutamate receptor activity | 6 | 8.59E-06 | 4.36E-02 |
| *Cellular components* | | | |
| cell periphery | 176 | 2.07E-09 | 4.14E-06 |
| plasma membrane | 163 | 1.46E-08 | 1.46E-05 |
| receptor complex | 27 | 5.31E-06 | 3.54E-03 |
| ***MDD responders vs. MDD non-responders*** | | | |
| *Biological processes* | | | |
| adaptive immune response | 38 | 6.85E-10 | 1.04E-05 |
| immunoglobulin production | 14 | 2.87E-07 | 2.19E-03 |
| production of molecular mediator of immune response | 14 | 4.40E-07 | 2.23E-03 |
| cell adhesion | 39 | 2.05E-06 | 7.81E-03 |
| immune response | 56 | 7.48E-06 | 2.28E-02 |
| *Molecular functions* | | | |
| antigen binding | 20 | 5.73E-10 | 2.91E-06 |
| *Cellular components* | | | |
| immunoglobulin complex | 21 | 2.87E-11 | 5.74E-08 |
| cell periphery | 159 | 2.36E-06 | 2.35E-03 |
| laminin-11 complex | 3 | 5.75E-06 | 3.83E-03 |
| T cell receptor complex | 12 | 2.16E-05 | 1.08E-02 |
| ***MDD unmedicated vs. MDD responders*** | | | |
| *Cellular components* | | | |
| T cell receptor complex | 12 | 4.58E-06 | 9.16E-03 |
| ***MDD unmedicated vs. MDD non-responders*** | | | |
| *Biological processes* | | | |
| lymphocyte mediated immunity | 20 | 8.28E-06 | 3.16E-02 |
| adaptive immune response | 38 | 4.39E-06 | 3.35E-02 |
| immune system process | 92 | 7.54E-06 | 3.83E-02 |
| immune response | 73 | 3.89E-06 | 5.93E-02 |
| *Molecular functions* | | | |
| MHC class Ib receptor activity | 4 | 1.28E-05 | 3.26E-02 |
| MHC class I protein complex binding | 4 | 1.28E-05 | 6.51E-02 |
| *Cellular components* | | | |
| side of membrane | 43 | 3.98E-07 | 7.95E-04 |
| external side of plasma membrane | 27 | 5.77E-06 | 5.77E-03 |
| laminin-11 complex | 3 | 1.56E-05 | 1.04E-02 |
| ***MDD all cases vs. controls*** | | | |
| *Biological processes* | | | |
| response to stimulus | 181 | 6.31E-10 | 9.61E-06 |
| immune response | 57 | 1.79E-08 | 9.12E-05 |
| immune system process | 71 | 1.54E-08 | 1.18E-04 |
| response to external biotic stimulus | 48 | 1.75E-07 | 3.81E-04 |
| response to other organism | 48 | 1.71E-07 | 4.34E-04 |
| negative regulation of viral genome replication | 9 | 1.52E-07 | 4.63E-04 |
| negative regulation of response to biotic stimulus | 12 | 2.70E-07 | 5.14E-04 |
| response to biotic stimulus | 49 | 1.46E-07 | 5.56E-04 |
| response to bacterium | 30 | 3.62E-07 | 6.13E-04 |
| defense response | 48 | 5.99E-07 | 9.13E-04 |
| regulation of nuclease activity | 6 | 7.84E-07 | 1.09E-03 |
| biological process involved in interspecies interaction between organisms | 50 | 8.75E-07 | 1.11E-03 |
| regulation of molecular function | 59 | 1.31E-06 | 1.54E-03 |
| defense response to other organism | 37 | 1.47E-06 | 1.60E-03 |
| regulation of response to external stimulus | 39 | 1.57E-06 | 1.60E-03 |
| regulation of response to biotic stimulus | 24 | 2.00E-06 | 1.90E-03 |
| negative regulation of viral process | 10 | 2.34E-06 | 2.10E-03 |
| regulation of multicellular organismal process | 77 | 3.54E-06 | 3.00E-03 |
| negative regulation of multicellular organismal process | 38 | 4.55E-06 | 3.65E-03 |
| response to external stimulus | 63 | 5.32E-06 | 3.69E-03 |
| defense response to bacterium | 17 | 6.67E-06 | 3.77E-03 |
| regulation of BMP signaling pathway | 10 | 5.29E-06 | 3.84E-03 |
| regulation of ribonuclease activity | 4 | 6.62E-06 | 3.88E-03 |
| regulation of defense response | 30 | 5.16E-06 | 3.93E-03 |
| regulation of viral genome replication | 9 | 6.56E-06 | 4.00E-03 |
| regulation of immune response | 33 | 6.54E-06 | 4.15E-03 |
| defense response to symbiont | 33 | 6.41E-06 | 4.25E-03 |
| negative regulation of innate immune response | 9 | 7.96E-06 | 4.33E-03 |
| regulation of catalytic activity | 44 | 8.47E-06 | 4.45E-03 |
| negative regulation of immune system process | 22 | 1.33E-05 | 6.76E-03 |
| regulation of viral life cycle | 11 | 1.62E-05 | 7.94E-03 |
| regulation of immune system process | 46 | 2.04E-05 | 9.71E-03 |
| defense response to virus | 15 | 2.35E-05 | 1.05E-02 |
| adaptive immune response | 26 | 2.29E-05 | 1.06E-02 |
| negative regulation of cytokine-mediated signaling pathway | 8 | 3.27E-05 | 1.28E-02 |
| cell surface receptor signaling pathway | 56 | 3.20E-05 | 1.28E-02 |
| negative regulation of cellular response to growth factor stimulus | 9 | 3.14E-05 | 1.29E-02 |
| signaling | 112 | 3.40E-05 | 1.30E-02 |
| negative regulation of BMP signaling pathway | 7 | 3.00E-05 | 1.31E-02 |
| innate immune response | 28 | 3.09E-05 | 1.31E-02 |
| regulation of innate immune response | 19 | 4.68E-05 | 1.70E-02 |
| negative regulation of cell adhesion | 15 | 4.91E-05 | 1.70E-02 |
| B cell mediated immunity | 12 | 4.88E-05 | 1.73E-02 |
| negative regulation of response to cytokine stimulus | 8 | 4.66E-05 | 1.73E-02 |
| regulation of viral process | 11 | 6.64E-05 | 2.25E-02 |
| cell communication | 113 | 7.21E-05 | 2.39E-02 |
| response to virus | 17 | 7.60E-05 | 2.47E-02 |
| response to lipid | 29 | 8.18E-05 | 2.60E-02 |
| cellular response to peptide | 15 | 9.66E-05 | 3.01E-02 |
| regulation of response to stimulus | 90 | 1.02E-04 | 3.12E-02 |
| cell-cell signaling | 34 | 1.08E-04 | 3.23E-02 |
| interleukin-27-mediated signaling pathway | 3 | 1.22E-04 | 3.57E-02 |
| antiviral innate immune response | 6 | 1.30E-04 | 3.73E-02 |
| positive regulation of response to external stimulus | 21 | 1.48E-04 | 4.19E-02 |
| response to lipopolysaccharide | 15 | 1.52E-04 | 4.22E-02 |
| signal transduction | 103 | 1.72E-04 | 4.59E-02 |
| immunoglobulin mediated immune response | 11 | 1.71E-04 | 4.67E-02 |
| negative regulation of immune response | 11 | 1.88E-04 | 4.94E-02 |
| lymphocyte mediated immunity | 13 | 1.93E-04 | 5.00E-02 |
| response to stimulus | 181 | 6.31E-10 | 9.61E-06 |
| *Molecular functions* | | | |
| 2'-5'-oligoadenylate synthetase activity | 3 | 3.65E-06 | 1.85E-02 |
| antigen binding | 13 | 1.41E-05 | 3.57E-02 |
| *Cellular components* | | | |
| extracellular region | 106 | 2.08E-07 | 4.17E-04 |
| extracellular space | 86 | 7.05E-07 | 7.04E-04 |
| immunoglobulin complex | 14 | 1.42E-06 | 9.49E-04 |
| cell periphery | 139 | 3.18E-06 | 1.59E-03 |
| blood microparticle | 11 | 1.42E-05 | 5.67E-03 |
| plasma membrane | 127 | 2.71E-05 | 9.04E-03 |
| catalytic complex | 10 | 1.58E-04 | 4.50E-02 |
| **Merged groups** | | | |
| ***MDD non-responders/unmedicated with CRP>1 vs. controls*** | | | |
| cytoplasmic translation | 32 | 3.63E-26 | 5.53E-22 |
| translation | 45 | 3.65E-21 | 2.79E-17 |
| peptide biosynthetic process | 46 | 9.54E-21 | 4.85E-17 |
| amide biosynthetic process | 49 | 1.47E-18 | 5.61E-15 |
| immune response | 90 | 2.65E-18 | 8.09E-15 |
| peptide metabolic process | 48 | 1.40E-17 | 3.56E-14 |
| immune system process | 106 | 2.17E-16 | 4.72E-13 |
| amide metabolic process | 53 | 7.68E-14 | 1.46E-10 |
| organonitrogen compound biosynthetic process | 68 | 2.72E-12 | 4.60E-09 |
| innate immune response | 48 | 3.17E-12 | 4.83E-09 |
| defense response to symbiont | 53 | 3.95E-12 | 5.48E-09 |
| defense response to other organism | 56 | 7.89E-12 | 1.00E-08 |
| response to external biotic stimulus | 67 | 2.25E-11 | 2.29E-08 |
| biological process involved in interspecies interaction between organisms | 72 | 2.21E-11 | 2.41E-08 |
| response to other organism | 67 | 2.16E-11 | 2.54E-08 |
| oxidative phosphorylation | 18 | 3.19E-11 | 3.04E-08 |
| response to biotic stimulus | 67 | 6.16E-11 | 5.52E-08 |
| cellular nitrogen compound biosynthetic process | 70 | 8.33E-11 | 7.06E-08 |
| defense response | 67 | 1.28E-10 | 1.02E-07 |
| defense response to virus | 25 | 2.84E-10 | 2.16E-07 |
| response to virus | 29 | 5.65E-10 | 4.10E-07 |
| negative regulation of viral genome replication | 12 | 7.28E-10 | 5.04E-07 |
| aerobic electron transport chain | 14 | 2.29E-09 | 1.51E-06 |
| ATP synthesis coupled electron transport | 14 | 4.81E-09 | 2.93E-06 |
| mitochondrial ATP synthesis coupled electron transport | 14 | 4.81E-09 | 3.06E-06 |
| aerobic respiration | 18 | 5.73E-09 | 3.36E-06 |
| respiratory electron transport chain | 15 | 1.02E-08 | 5.78E-06 |
| cellular respiration | 19 | 1.56E-08 | 8.49E-06 |
| negative regulation of viral process | 13 | 6.30E-08 | 3.31E-05 |
| organonitrogen compound metabolic process | 141 | 7.72E-08 | 3.92E-05 |
| regulation of viral genome replication | 12 | 1.27E-07 | 6.24E-05 |
| response to external stimulus | 82 | 1.94E-07 | 9.25E-05 |
| regulation of viral life cycle | 15 | 2.35E-07 | 1.09E-04 |
| regulation of defense response | 39 | 2.90E-07 | 1.30E-04 |
| adaptive immune response | 34 | 1.12E-06 | 4.89E-04 |
| electron transport chain | 15 | 1.45E-06 | 6.15E-04 |
| regulation of viral process | 15 | 1.69E-06 | 6.96E-04 |
| energy derivation by oxidation of organic compounds | 19 | 1.88E-06 | 7.53E-04 |
| mucosal immune response | 7 | 2.15E-06 | 8.41E-04 |
| regulation of response to biotic stimulus | 28 | 2.37E-06 | 9.03E-04 |
| protein metabolic process | 109 | 2.54E-06 | 9.43E-04 |
| innate immune response in mucosa | 6 | 3.57E-06 | 1.29E-03 |
| negative regulation of response to biotic stimulus | 12 | 4.15E-06 | 1.47E-03 |
| negative regulation of cytokine-mediated signaling pathway | 10 | 4.45E-06 | 1.54E-03 |
| defense response to bacterium | 20 | 4.59E-06 | 1.55E-03 |
| organ or tissue specific immune response | 7 | 5.14E-06 | 1.70E-03 |
| interleukin-27-mediated signaling pathway | 4 | 5.32E-06 | 1.73E-03 |
| gene expression | 82 | 5.70E-06 | 1.81E-03 |
| mitochondrial electron transport, cytochrome c to oxygen | 6 | 6.22E-06 | 1.93E-03 |
| negative regulation of response to cytokine stimulus | 10 | 6.95E-06 | 2.12E-03 |
| Unclassified | 27 | 7.93E-06 | 2.33E-03 |
| biological_process | 386 | 7.93E-06 | 2.37E-03 |
| ribosomal small subunit biogenesis | 11 | 8.51E-06 | 2.45E-03 |
| B cell mediated immunity | 15 | 8.68E-06 | 2.45E-03 |
| 7-methylguanosine cap hypermethylation | 4 | 1.05E-05 | 2.90E-03 |
| positive regulation of defense response | 23 | 1.10E-05 | 3.00E-03 |
| cellular process | 338 | 1.27E-05 | 3.28E-03 |
| response to stress | 103 | 1.27E-05 | 3.33E-03 |
| regulation of transcription by RNA polymerase II | 25 | 1.26E-05 | 3.36E-03 |
| lymphocyte mediated immunity | 17 | 1.87E-05 | 4.67E-03 |
| regulation of ribonuclease activity | 4 | 1.86E-05 | 4.71E-03 |
| defense response to fungus | 8 | 1.95E-05 | 4.80E-03 |
| leukocyte mediated immunity | 19 | 2.14E-05 | 5.17E-03 |
| ribosome biogenesis | 19 | 2.33E-05 | 5.56E-03 |
| ribonucleoprotein complex biogenesis | 24 | 2.41E-05 | 5.64E-03 |
| immunoglobulin mediated immune response | 14 | 2.79E-05 | 6.43E-03 |
| response to stimulus | 206 | 2.93E-05 | 6.67E-03 |
| positive regulation of signal transduction by p53 class mediator | 6 | 3.02E-05 | 6.77E-03 |
| regulation of response to external stimulus | 43 | 3.14E-05 | 6.93E-03 |
| chromatin organization | 2 | 3.44E-05 | 7.48E-03 |
| response to bacterium | 31 | 3.69E-05 | 7.93E-03 |
| mitochondrial respiratory chain complex assembly | 10 | 3.90E-05 | 8.25E-03 |
| proton motive force-driven mitochondrial ATP synthesis | 8 | 4.06E-05 | 8.36E-03 |
| nervous system process | 10 | 4.02E-05 | 8.40E-03 |
| cellular response to lipopolysaccharide | 14 | 4.41E-05 | 8.96E-03 |
| protein-DNA complex organization | 3 | 4.91E-05 | 9.85E-03 |
| antibacterial humoral response | 8 | 5.08E-05 | 1.01E-02 |
| negative regulation of innate immune response | 9 | 6.21E-05 | 1.21E-02 |
| chromatin remodeling | 1 | 6.44E-05 | 1.24E-02 |
| metabolic process | 200 | 6.56E-05 | 1.25E-02 |
| antiviral innate immune response | 7 | 6.71E-05 | 1.26E-02 |
| regulation of innate immune response | 22 | 6.80E-05 | 1.26E-02 |
| nucleoside triphosphate biosynthetic process | 10 | 6.89E-05 | 1.26E-02 |
| response to fungus | 8 | 7.02E-05 | 1.27E-02 |
| cellular biosynthetic process | 110 | 7.44E-05 | 1.33E-02 |
| disruption of plasma membrane integrity in another organism | 3 | 7.77E-05 | 1.36E-02 |
| disruption of cellular anatomical structure in another organism | 3 | 7.77E-05 | 1.38E-02 |
| cellular response to molecule of bacterial origin | 14 | 7.97E-05 | 1.38E-02 |
| mitochondrion organization | 22 | 8.59E-05 | 1.47E-02 |
| regulation of cytokine-mediated signaling pathway | 12 | 9.64E-05 | 1.63E-02 |
| response to type I interferon | 7 | 9.75E-05 | 1.63E-02 |
| cellular nitrogen compound metabolic process | 96 | 1.02E-04 | 1.69E-02 |
| proton motive force-driven ATP synthesis | 8 | 1.05E-04 | 1.73E-02 |
| macromolecule biosynthetic process | 88 | 1.17E-04 | 1.90E-02 |
| mitochondrial cytochrome c oxidase assembly | 5 | 1.21E-04 | 1.94E-02 |
| adaptive immune response based on somatic recombination of immune receptors built from immunoglobulin superfamily domains | 16 | 1.26E-04 | 2.00E-02 |
| cellular metabolic process | 165 | 1.34E-04 | 2.11E-02 |
| organic substance biosynthetic process | 111 | 1.46E-04 | 2.27E-02 |
| positive regulation of inflammatory response | 11 | 1.50E-04 | 2.30E-02 |
| positive regulation of response to external stimulus | 25 | 1.56E-04 | 2.37E-02 |
| biosynthetic process | 112 | 1.57E-04 | 2.38E-02 |
| immune effector process | 23 | 1.72E-04 | 2.57E-02 |
| regulation of response to cytokine stimulus | 12 | 1.81E-04 | 2.68E-02 |
| RNA capping | 4 | 2.40E-04 | 3.51E-02 |
| antimicrobial humoral immune response mediated by antimicrobial peptide | 9 | 2.47E-04 | 3.58E-02 |
| nitrogen compound metabolic process | 164 | 2.54E-04 | 3.65E-02 |
| regulation of immune response | 35 | 2.59E-04 | 3.70E-02 |
| negative regulation of immune system process | 23 | 2.69E-04 | 3.72E-02 |
| regulation of response to stress | 47 | 2.66E-04 | 3.73E-02 |
| regulation of inflammatory response | 19 | 2.65E-04 | 3.75E-02 |
| cellular response to biotic stimulus | 14 | 2.75E-04 | 3.78E-02 |
| ATP biosynthetic process | 8 | 2.82E-04 | 3.81E-02 |
| macromolecule metabolic process | 147 | 2.81E-04 | 3.83E-02 |
| respiratory chain complex IV assembly | 5 | 2.98E-04 | 3.96E-02 |
| mitochondrial electron transport, NADH to ubiquinone | 6 | 2.98E-04 | 3.99E-02 |
| *Molecular functions* | | | |
| structural constituent of ribosome | 41 | 8.53E-33 | 4.33E-29 |
| structural molecule activity | 55 | 9.84E-16 | 2.49E-12 |
| antigen binding | 18 | 1.17E-07 | 1.98E-04 |
| RNA binding | 65 | 2.52E-07 | 3.19E-04 |
| ubiquitin ligase inhibitor activity | 5 | 3.74E-07 | 3.79E-04 |
| ubiquitin-protein transferase inhibitor activity | 5 | 2.23E-06 | 1.89E-03 |
| 2'-5'-oligoadenylate synthetase activity | 3 | 8.01E-06 | 5.80E-03 |
| sequence-specific double-stranded DNA binding | 11 | 2.11E-05 | 1.34E-02 |
| DNA-binding transcription factor activity | 10 | 2.84E-05 | 1.60E-02 |
| sequence-specific DNA binding | 13 | 3.83E-05 | 1.76E-02 |
| DNA-binding transcription factor activity, RNA polymerase II-specific | 9 | 3.78E-05 | 1.92E-02 |
| transcription regulatory region nucleic acid binding | 11 | 5.30E-05 | 2.07E-02 |
| transcription cis-regulatory region binding | 11 | 5.29E-05 | 2.24E-02 |
| RNA polymerase II transcription regulatory region sequence-specific DNA binding | 10 | 7.17E-05 | 2.60E-02 |
| mRNA 5'-UTR binding | 5 | 9.83E-05 | 3.32E-02 |
| oxidoreductase activity, acting on NAD(P)H, quinone or similar compound as acceptor | 7 | 1.38E-04 | 4.38E-02 |
| *Cellular components* | | | |
| ribosomal subunit | 41 | 6.54E-30 | 1.31E-26 |
| ribosome | 44 | 7.59E-29 | 7.58E-26 |
| cytosolic ribosome | 33 | 6.41E-28 | 4.27E-25 |
| cytosolic large ribosomal subunit | 20 | 1.41E-19 | 7.05E-17 |
| large ribosomal subunit | 25 | 8.27E-19 | 3.31E-16 |
| ribonucleoprotein complex | 52 | 7.56E-16 | 2.52E-13 |
| mitochondrial protein-containing complex | 29 | 4.09E-12 | 1.17E-09 |
| cytosolic small ribosomal subunit | 13 | 5.34E-12 | 1.33E-09 |
| small ribosomal subunit | 16 | 6.40E-12 | 1.42E-09 |
| mitochondrial inner membrane | 35 | 1.67E-10 | 3.34E-08 |
| extracellular region | 141 | 2.95E-10 | 5.35E-08 |
| mitochondrial membrane | 44 | 5.64E-10 | 9.39E-08 |
| organelle inner membrane | 36 | 8.40E-10 | 1.29E-07 |
| extracellular space | 115 | 1.81E-09 | 2.58E-07 |
| mitochondrial envelope | 44 | 3.57E-09 | 4.75E-07 |
| inner mitochondrial membrane protein complex | 18 | 4.26E-09 | 5.32E-07 |
| specific granule lumen | 11 | 3.75E-08 | 4.41E-06 |
| immunoglobulin complex | 18 | 4.90E-08 | 5.44E-06 |
| specific granule | 16 | 1.50E-07 | 1.58E-05 |
| extracellular membrane-bounded organelle | 77 | 2.91E-07 | 2.65E-05 |
| extracellular organelle | 77 | 2.91E-07 | 2.77E-05 |
| extracellular vesicle | 77 | 2.89E-07 | 2.89E-05 |
| respiratory chain complex | 12 | 3.48E-07 | 3.02E-05 |
| mitochondrial respirasome | 12 | 4.94E-07 | 4.12E-05 |
| extracellular exosome | 75 | 6.63E-07 | 5.30E-05 |
| respirasome | 12 | 1.18E-06 | 9.10E-05 |
| organelle envelope | 52 | 1.62E-06 | 1.12E-04 |
| protein-DNA complex | 7 | 1.70E-06 | 1.13E-04 |
| envelope | 52 | 1.62E-06 | 1.16E-04 |
| cytochrome complex | 8 | 1.59E-06 | 1.18E-04 |
| chromatin | 7 | 4.60E-06 | 2.97E-04 |
| membrane | 246 | 5.55E-06 | 3.46E-04 |
| mitochondrion | 61 | 7.05E-06 | 4.27E-04 |
| secretory granule lumen | 20 | 8.49E-06 | 4.99E-04 |
| cytoplasmic vesicle lumen | 20 | 1.07E-05 | 6.08E-04 |
| vesicle lumen | 20 | 1.11E-05 | 6.19E-04 |
| tertiary granule lumen | 8 | 1.31E-05 | 7.06E-04 |
| cellular anatomical entity | 399 | 2.09E-05 | 1.07E-03 |
| cytoplasm | 286 | 2.06E-05 | 1.08E-03 |
| U4 snRNP | 4 | 4.71E-05 | 2.35E-03 |
| Unclassified | 13 | 5.18E-05 | 2.46E-03 |
| cellular_component | 400 | 5.18E-05 | 2.52E-03 |
| chromosome | 17 | 5.61E-05 | 2.61E-03 |
| protein-containing complex | 169 | 7.31E-05 | 3.32E-03 |
| methylosome | 4 | 9.88E-05 | 4.29E-03 |
| mitochondrial respiratory chain complex IV | 5 | 9.83E-05 | 4.37E-03 |
| small-subunit processome | 8 | 1.16E-04 | 4.94E-03 |
| tertiary granule | 12 | 1.22E-04 | 5.08E-03 |
| secretory granule | 36 | 1.30E-04 | 5.29E-03 |
| vesicle | 112 | 1.55E-04 | 6.08E-03 |
| pICln-Sm protein complex | 3 | 1.53E-04 | 6.12E-03 |
| respiratory chain complex IV | 5 | 1.78E-04 | 6.83E-03 |
| cell periphery | 163 | 3.94E-04 | 1.46E-02 |
| SMN-Sm protein complex | 4 | 3.90E-04 | 1.47E-02 |
| mitochondrial ribosome | 8 | 4.52E-04 | 1.61E-02 |
| organellar ribosome | 8 | 4.52E-04 | 1.64E-02 |
| secretory vesicle | 39 | 4.75E-04 | 1.66E-02 |
| U1 snRNP | 4 | 4.87E-04 | 1.68E-02 |
| U5 snRNP | 4 | 5.99E-04 | 2.03E-02 |
| U4/U6 x U5 tri-snRNP complex | 5 | 6.26E-04 | 2.09E-02 |
| spliceosomal tri-snRNP complex | 5 | 7.15E-04 | 2.34E-02 |
| mitochondrial proton-transporting ATP synthase complex | 4 | 8.76E-04 | 2.82E-02 |
| proton-transporting ATP synthase complex | 4 | 1.04E-03 | 3.31E-02 |
| primary lysosome | 10 | 1.12E-03 | 3.44E-02 |
| complement component C1q complex | 2 | 1.19E-03 | 3.44E-02 |
| focal adhesion | 19 | 1.15E-03 | 3.48E-02 |
| complement component C1 complex | 2 | 1.19E-03 | 3.49E-02 |
| azurophil granule | 10 | 1.12E-03 | 3.49E-02 |
| mitochondrial proton-transporting ATP synthase complex, coupling factor F(o) | 3 | 1.17E-03 | 3.50E-02 |
| cell-substrate junction | 19 | 1.42E-03 | 4.06E-02 |
| U2 snRNP | 4 | 1.68E-03 | 4.73E-02 |
| preribosome | 8 | 1.79E-03 | 4.97E-02 |
| ***MDD responders with CRP>1 vs. controls*** | | | |
| *Biological processes* | | | |
| response to external biotic stimulus | 106 | 2.17E-29 | 1.65E-25 |
| response to other organism | 106 | 1.81E-29 | 2.75E-25 |
| response to biotic stimulus | 106 | 2.97E-28 | 1.51E-24 |
| defense response to other organism | 87 | 3.62E-27 | 1.38E-23 |
| defense response to virus | 46 | 8.40E-27 | 2.56E-23 |
| biological process involved in interspecies interaction between organisms | 108 | 2.23E-26 | 5.67E-23 |
| defense response to symbiont | 80 | 1.12E-25 | 2.44E-22 |
| defense response | 101 | 7.32E-25 | 1.39E-21 |
| response to virus | 50 | 2.74E-24 | 4.64E-21 |
| immune response | 108 | 8.04E-24 | 1.23E-20 |
| innate immune response | 70 | 1.68E-23 | 2.33E-20 |
| response to external stimulus | 128 | 1.14E-22 | 1.45E-19 |
| immune system process | 127 | 6.94E-22 | 8.13E-19 |
| negative regulation of viral process | 22 | 2.42E-16 | 2.63E-13 |
| regulation of response to biotic stimulus | 47 | 6.57E-16 | 6.68E-13 |
| negative regulation of viral genome replication | 17 | 2.66E-15 | 2.54E-12 |
| regulation of response to external stimulus | 69 | 1.36E-14 | 1.22E-11 |
| regulation of defense response | 56 | 3.17E-14 | 2.68E-11 |
| regulation of viral life cycle | 23 | 2.27E-13 | 1.73E-10 |
| response to stress | 141 | 2.17E-13 | 1.74E-10 |
| response to bacterium | 50 | 4.45E-13 | 3.23E-10 |
| regulation of viral process | 24 | 8.22E-13 | 5.69E-10 |
| regulation of innate immune response | 37 | 4.97E-12 | 3.29E-09 |
| regulation of viral genome replication | 17 | 6.66E-12 | 4.23E-09 |
| regulation of response to stress | 71 | 3.96E-11 | 2.41E-08 |
| negative regulation of response to biotic stimulus | 18 | 1.59E-10 | 9.30E-08 |
| positive regulation of defense response | 33 | 4.21E-10 | 2.38E-07 |
| regulation of immune response | 53 | 4.57E-10 | 2.49E-07 |
| response to stimulus | 251 | 1.01E-09 | 5.33E-07 |
| negative regulation of innate immune response | 15 | 1.05E-09 | 5.33E-07 |
| antiviral innate immune response | 12 | 1.17E-09 | 5.77E-07 |
| positive regulation of response to external stimulus | 38 | 1.47E-09 | 7.02E-07 |
| positive regulation of innate immune response | 25 | 6.68E-09 | 3.08E-06 |
| positive regulation of response to biotic stimulus | 26 | 1.00E-08 | 4.48E-06 |
| regulation of response to stimulus | 140 | 2.90E-08 | 1.26E-05 |
| cytoplasmic translation | 16 | 3.55E-08 | 1.50E-05 |
| regulation of cytokine production | 44 | 4.55E-08 | 1.82E-05 |
| defense response to bacterium | 25 | 4.46E-08 | 1.84E-05 |
| negative regulation of response to external stimulus | 29 | 7.28E-08 | 2.84E-05 |
| interleukin-27-mediated signaling pathway | 5 | 1.20E-07 | 4.58E-05 |
| response to type I interferon | 10 | 3.27E-07 | 1.21E-04 |
| negative regulation of immune response | 18 | 3.68E-07 | 1.34E-04 |
| regulation of signaling | 121 | 4.30E-07 | 1.52E-04 |
| positive regulation of immune response | 37 | 6.04E-07 | 2.09E-04 |
| regulation of immune system process | 66 | 6.97E-07 | 2.36E-04 |
| response to interferon-beta | 8 | 7.24E-07 | 2.40E-04 |
| regulation of type I interferon production | 14 | 8.15E-07 | 2.64E-04 |
| regulation of cell communication | 120 | 8.50E-07 | 2.70E-04 |
| response to cytokine | 42 | 1.07E-06 | 3.25E-04 |
| positive regulation of immune system process | 48 | 1.05E-06 | 3.28E-04 |
| negative regulation of biological process | 165 | 1.18E-06 | 3.47E-04 |
| regulation of signal transduction | 108 | 1.17E-06 | 3.49E-04 |
| activation of innate immune response | 16 | 1.30E-06 | 3.74E-04 |
| negative regulation of defense response | 20 | 1.51E-06 | 4.27E-04 |
| regulation of response to cytokine stimulus | 16 | 1.93E-06 | 5.35E-04 |
| regulation of catalytic activity | 60 | 2.63E-06 | 7.16E-04 |
| response to molecule of bacterial origin | 23 | 3.48E-06 | 9.31E-04 |
| response to interferon-alpha | 6 | 3.95E-06 | 1.02E-03 |
| positive regulation of interferon-beta production | 8 | 4.01E-06 | 1.02E-03 |
| regulation of cytokine-mediated signaling pathway | 15 | 3.93E-06 | 1.03E-03 |
| positive regulation of inflammatory response | 14 | 4.97E-06 | 1.22E-03 |
| translation | 25 | 4.95E-06 | 1.24E-03 |
| negative regulation of immune system process | 29 | 5.15E-06 | 1.25E-03 |
| positive regulation of type I interferon production | 10 | 5.77E-06 | 1.37E-03 |
| negative regulation of multicellular organismal process | 50 | 6.27E-06 | 1.47E-03 |
| inflammatory response | 31 | 7.62E-06 | 1.76E-03 |
| regulation of interferon-beta production | 9 | 7.92E-06 | 1.80E-03 |
| regulation of defense response to virus by host | 8 | 8.20E-06 | 1.81E-03 |
| cellular response to type I interferon | 8 | 8.20E-06 | 1.84E-03 |
| B cell mediated immunity | 16 | 9.23E-06 | 2.01E-03 |
| negative regulation of cytokine-mediated signaling pathway | 10 | 1.33E-05 | 2.85E-03 |
| response to lipopolysaccharide | 21 | 1.60E-05 | 3.39E-03 |
| peptide biosynthetic process | 25 | 1.66E-05 | 3.46E-03 |
| regulation of defense response to virus | 9 | 1.76E-05 | 3.62E-03 |
| pattern recognition receptor signaling pathway | 12 | 2.06E-05 | 4.13E-03 |
| negative regulation of response to cytokine stimulus | 10 | 2.05E-05 | 4.17E-03 |
| cellular response to molecule of bacterial origin | 16 | 2.21E-05 | 4.38E-03 |
| regulation of hydrolase activity | 36 | 2.34E-05 | 4.58E-03 |
| regulation of multicellular organismal process | 101 | 2.45E-05 | 4.72E-03 |
| immunoglobulin mediated immune response | 15 | 2.75E-05 | 5.23E-03 |
| regulation of ribonuclease activity | 4 | 3.03E-05 | 5.44E-03 |
| sensory perception of smell | 0 | 2.91E-05 | 5.48E-03 |
| antibody-dependent cellular cytotoxicity | 4 | 3.03E-05 | 5.50E-03 |
| type IIa hypersensitivity | 4 | 3.03E-05 | 5.57E-03 |
| negative regulation of catalytic activity | 25 | 3.18E-05 | 5.63E-03 |
| type II hypersensitivity | 4 | 3.03E-05 | 5.64E-03 |
| positive regulation of cytokine production | 27 | 3.72E-05 | 6.52E-03 |
| positive regulation of response to stimulus | 80 | 4.48E-05 | 7.67E-03 |
| cellular response to lipopolysaccharide | 15 | 4.46E-05 | 7.72E-03 |
| MDA-5 signaling pathway | 3 | 4.59E-05 | 7.77E-03 |
| adaptive immune response based on somatic recombination of immune receptors built from immunoglobulin superfamily domains | 18 | 5.25E-05 | 8.80E-03 |
| regulation of type I interferon-mediated signaling pathway | 7 | 5.54E-05 | 9.18E-03 |
| negative regulation of protein metabolic process | 37 | 5.67E-05 | 9.29E-03 |
| interferon-mediated signaling pathway | 8 | 6.09E-05 | 9.77E-03 |
| mucosal immune response | 6 | 6.06E-05 | 9.83E-03 |
| respiratory electron transport chain | 11 | 6.32E-05 | 1.00E-02 |
| regulation of inflammatory response | 22 | 6.47E-05 | 1.01E-02 |
| detection of chemical stimulus involved in sensory perception of smell | 0 | 6.55E-05 | 1.01E-02 |
| type I interferon-mediated signaling pathway | 7 | 6.44E-05 | 1.01E-02 |
| cellular response to cytokine stimulus | 34 | 6.94E-05 | 1.06E-02 |
| regulation of interferon-alpha production | 6 | 7.32E-05 | 1.10E-02 |
| positive regulation of RIG-I signaling pathway | 4 | 7.66E-05 | 1.14E-02 |
| activation of immune response | 22 | 8.45E-05 | 1.25E-02 |
| lymphocyte mediated immunity | 17 | 8.86E-05 | 1.29E-02 |
| regulation of pattern recognition receptor signaling pathway | 13 | 8.79E-05 | 1.29E-02 |
| cellular response to biotic stimulus | 16 | 9.18E-05 | 1.32E-02 |
| innate immune response-activating signaling pathway | 12 | 9.31E-05 | 1.33E-02 |
| sensory perception of chemical stimulus | 1 | 1.00E-04 | 1.41E-02 |
| positive regulation of defense response to virus by host | 6 | 1.05E-04 | 1.46E-02 |
| positive regulation of pattern recognition receptor signaling pathway | 8 | 1.09E-04 | 1.51E-02 |
| hypersensitivity | 4 | 1.13E-04 | 1.55E-02 |
| peptide metabolic process | 28 | 1.14E-04 | 1.56E-02 |
| positive regulation of response to cytokine stimulus | 8 | 1.21E-04 | 1.61E-02 |
| electron transport chain | 13 | 1.20E-04 | 1.61E-02 |
| antibacterial humoral response | 8 | 1.21E-04 | 1.62E-02 |
| organ or tissue specific immune response | 6 | 1.24E-04 | 1.63E-02 |
| cell chemotaxis | 15 | 1.27E-04 | 1.65E-02 |
| immune effector process | 25 | 1.32E-04 | 1.71E-02 |
| negative regulation of peptidase activity | 11 | 1.44E-04 | 1.82E-02 |
| negative regulation of type I interferon-mediated signaling pathway | 5 | 1.42E-04 | 1.82E-02 |
| detection of chemical stimulus | 1 | 1.44E-04 | 1.82E-02 |
| negative regulation of cytokine production | 18 | 1.48E-04 | 1.85E-02 |
| cytoplasmic pattern recognition receptor signaling pathway | 8 | 1.50E-04 | 1.86E-02 |
| branching involved in labyrinthine layer morphogenesis | 4 | 1.60E-04 | 1.97E-02 |
| regulation of intracellular signal transduction | 65 | 1.62E-04 | 1.97E-02 |
| negative regulation of viral entry into host cell | 5 | 1.76E-04 | 2.12E-02 |
| filopodium assembly | 5 | 1.76E-04 | 2.13E-02 |
| amide biosynthetic process | 27 | 1.94E-04 | 2.32E-02 |
| negative regulation of response to stimulus | 61 | 2.04E-04 | 2.40E-02 |
| ISG15-protein conjugation | 3 | 2.22E-04 | 2.60E-02 |
| positive regulation of interferon-alpha production | 5 | 2.63E-04 | 3.06E-02 |
| regulation of molecular function | 70 | 2.74E-04 | 3.16E-02 |
| positive regulation of multicellular organismal process | 60 | 2.80E-04 | 3.21E-02 |
| detection of chemical stimulus involved in sensory perception | 1 | 3.02E-04 | 3.43E-02 |
| adaptive immune response | 31 | 3.18E-04 | 3.56E-02 |
| positive regulation of canonical NF-kappaB signal transduction | 14 | 3.21E-04 | 3.57E-02 |
| cellular response to interferon-beta | 5 | 3.17E-04 | 3.58E-02 |
| acute inflammatory response | 8 | 3.27E-04 | 3.61E-02 |
| leukocyte mediated immunity | 18 | 3.31E-04 | 3.63E-02 |
| positive regulation of cytokine-mediated signaling pathway | 7 | 3.68E-04 | 4.01E-02 |
| chemotaxis | 19 | 3.76E-04 | 4.07E-02 |
| positive regulation of NF-kappaB transcription factor activity | 11 | 3.86E-04 | 4.14E-02 |
| negative regulation of endopeptidase activity | 10 | 3.91E-04 | 4.16E-02 |
| taxis | 19 | 4.04E-04 | 4.28E-02 |
| cell killing | 12 | 4.27E-04 | 4.49E-02 |
| organonitrogen compound biosynthetic process | 51 | 4.45E-04 | 4.65E-02 |
| positive regulation of cellular process | 164 | 4.55E-04 | 4.72E-02 |
| *Molecular functions* | | | |
| structural constituent of ribosome | 20 | 1.65E-09 | 8.38E-06 |
| binding | 424 | 6.24E-09 | 1.58E-05 |
| Unclassified | 22 | 1.09E-06 | 9.21E-04 |
| molecular_function | 446 | 1.09E-06 | 1.10E-03 |
| double-stranded RNA binding | 11 | 7.43E-07 | 1.26E-03 |
| protein binding | 375 | 1.07E-06 | 1.36E-03 |
| 2'-5'-oligoadenylate synthetase activity | 3 | 1.17E-05 | 8.45E-03 |
| structural molecule activity | 38 | 1.62E-05 | 1.03E-02 |
| guanyl-nucleotide exchange factor activity | 16 | 7.89E-05 | 3.63E-02 |
| olfactory receptor activity | 0 | 6.49E-05 | 3.65E-02 |
| RNA binding | 63 | 7.58E-05 | 3.84E-02 |
| *Cellular components* | | | |
| cytosolic ribosome | 17 | 3.19E-09 | 6.38E-06 |
| ribosome | 23 | 1.32E-08 | 8.82E-06 |
| specific granule lumen | 12 | 1.27E-08 | 1.27E-05 |
| tertiary granule lumen | 11 | 3.61E-08 | 1.80E-05 |
| ribosomal subunit | 19 | 1.22E-07 | 4.86E-05 |
| specific granule | 16 | 7.94E-07 | 2.65E-04 |
| tertiary granule | 15 | 5.33E-06 | 1.52E-03 |
| cytosolic large ribosomal subunit | 9 | 7.92E-06 | 1.98E-03 |
| cytosolic small ribosomal subunit | 8 | 9.68E-06 | 2.15E-03 |
| secretory vesicle | 48 | 1.27E-05 | 2.54E-03 |
| secretory granule | 42 | 1.68E-05 | 2.81E-03 |
| small ribosomal subunit | 10 | 1.66E-05 | 3.01E-03 |
| cytosol | 167 | 2.34E-05 | 3.59E-03 |
| extracellular space | 110 | 3.36E-05 | 4.80E-03 |
| cytoplasm | 319 | 6.17E-05 | 8.22E-03 |
| extracellular region | 134 | 6.79E-05 | 8.48E-03 |
| vesicle | 123 | 3.01E-04 | 3.54E-02 |
| intracellular protein-containing complex | 7 | 4.39E-04 | 4.62E-02 |
| secretory granule lumen | 18 | 4.30E-04 | 4.78E-02 |
| ***MDD non-responders/unmedicated with CRP<1 vs. controls*** | | | |
| *Biological processes* | | | |
| immune response | 54 | 6.36E-08 | 9.69E-04 |
| adaptive immune response | 29 | 4.05E-07 | 3.09E-03 |
| regulation of ribonuclease activity | 4 | 5.68E-06 | 1.44E-02 |
| B cell mediated immunity | 13 | 6.71E-06 | 1.46E-02 |
| cell surface receptor signaling pathway | 57 | 5.24E-06 | 1.60E-02 |
| response to stimulus | 161 | 5.23E-06 | 1.99E-02 |
| immune system process | 62 | 3.92E-06 | 1.99E-02 |
| regulation of viral life cycle | 11 | 1.13E-05 | 2.15E-02 |
| defense response | 43 | 1.28E-05 | 2.17E-02 |
| negative regulation of viral genome replication | 7 | 1.65E-05 | 2.51E-02 |
| immunoglobulin mediated immune response | 12 | 2.59E-05 | 3.29E-02 |
| complement activation, classical pathway | 6 | 2.57E-05 | 3.56E-02 |
| regulation of viral genome replication | 8 | 3.87E-05 | 4.54E-02 |
| *Molecular functions* | | | |
| 2'-5'-oligoadenylate synthetase activity | 3 | 3.25E-06 | 8.24E-03 |
| antigen binding | 14 | 1.78E-06 | 9.02E-03 |
| chondroitin sulfate binding | 4 | 9.36E-06 | 1.58E-02 |
| *Cellular components* | | | |
| immunoglobulin complex | 16 | 2.32E-08 | 4.64E-05 |
| IgG immunoglobulin complex | 5 | 8.19E-07 | 5.45E-04 |
| cell periphery | 137 | 7.76E-07 | 7.76E-04 |
| plasma membrane | 121 | 6.01E-05 | 3.00E-02 |
| extracellular region | 93 | 8.91E-05 | 3.56E-02 |
| ***MDD responders with CRP<1 vs. controls*** | | | |
| *Cellular components* | | | |
| plasma membrane protein complex | 28 | 5.99E-07 | 1.20E-03 |
| T cell receptor complex | 11 | 5.80E-06 | 5.79E-03 |
| plasma membrane signaling receptor complex | 16 | 8.77E-06 | 5.84E-03 |
| ***MDD responders with CRP>1 vs. non-responders/unmedicated with CRP>1*** | | | |
| adaptive immune response | 43 | 5.36E-17 | 8.17E-13 |
| immune response | 63 | 1.20E-13 | 6.11E-10 |
| production of molecular mediator of immune response | 19 | 1.75E-13 | 6.66E-10 |
| immunoglobulin production | 19 | 9.21E-14 | 7.02E-10 |
| immune system process | 72 | 1.72E-11 | 5.24E-08 |
| leukocyte mediated immunity | 19 | 7.95E-08 | 1.73E-04 |
| B cell mediated immunity | 15 | 7.67E-08 | 1.95E-04 |
| immune effector process | 23 | 2.46E-07 | 4.17E-04 |
| adaptive immune response based on somatic recombination of immune receptors built from immunoglobulin superfamily domains | 17 | 2.34E-07 | 4.45E-04 |
| immunoglobulin mediated immune response | 14 | 3.54E-07 | 5.40E-04 |
| lymphocyte mediated immunity | 16 | 5.89E-07 | 8.16E-04 |
| *Molecular functions* | | | |
| antigen binding | 27 | 3.20E-19 | 1.62E-15 |
| *Cellular components* | | | |
| immunoglobulin complex | 31 | 1.51E-24 | 3.02E-21 |
| cell periphery | 127 | 1.53E-06 | 1.53E-03 |
| extracellular region | 92 | 4.28E-06 | 2.85E-03 |
| plasma membrane | 116 | 1.59E-05 | 7.93E-03 |
| nucleus | 72 | 2.39E-05 | 9.56E-03 |
| adherens junction | 11 | 6.42E-05 | 2.14E-02 |
| vacuole | 1 | 1.30E-04 | 3.71E-02 |
| ***MDD non-responders/unmedicated with CRP<1 vs. non-responders/unmedicated with CRP>1*** | | | |
| *Biological processes* | | | |
| cytoplasmic translation | 42 | 9.48E-29 | 1.45E-24 |
| peptide biosynthetic process | 63 | 2.16E-21 | 1.65E-17 |
| translation | 60 | 6.68E-21 | 3.40E-17 |
| amide biosynthetic process | 68 | 9.19E-19 | 3.50E-15 |
| peptide metabolic process | 67 | 7.87E-18 | 2.40E-14 |
| oxidative phosphorylation | 30 | 3.59E-17 | 9.13E-14 |
| organonitrogen compound biosynthetic process | 113 | 2.00E-16 | 4.36E-13 |
| immune system process | 160 | 9.24E-16 | 1.76E-12 |
| immune response | 126 | 5.07E-15 | 8.58E-12 |
| amide metabolic process | 79 | 5.92E-15 | 9.03E-12 |
| cellular nitrogen compound biosynthetic process | 118 | 1.25E-14 | 1.74E-11 |
| aerobic respiration | 30 | 2.85E-13 | 3.63E-10 |
| aerobic electron transport chain | 22 | 5.92E-13 | 6.95E-10 |
| ATP synthesis coupled electron transport | 22 | 1.99E-12 | 2.02E-09 |
| mitochondrial ATP synthesis coupled electron transport | 22 | 1.99E-12 | 2.17E-09 |
| nucleoside triphosphate biosynthetic process | 23 | 8.01E-12 | 7.64E-09 |
| cellular respiration | 30 | 2.82E-11 | 2.53E-08 |
| proton motive force-driven mitochondrial ATP synthesis | 17 | 8.99E-11 | 7.21E-08 |
| ribonucleoside triphosphate biosynthetic process | 21 | 8.72E-11 | 7.39E-08 |
| proton motive force-driven ATP synthesis | 18 | 9.73E-11 | 7.41E-08 |
| ATP biosynthetic process | 19 | 1.48E-10 | 1.02E-07 |
| organonitrogen compound metabolic process | 247 | 1.44E-10 | 1.04E-07 |
| respiratory electron transport chain | 22 | 1.73E-10 | 1.14E-07 |
| purine ribonucleoside triphosphate biosynthetic process | 20 | 1.93E-10 | 1.23E-07 |
| purine nucleoside triphosphate biosynthetic process | 20 | 2.36E-10 | 1.44E-07 |
| cellular biosynthetic process | 207 | 3.28E-09 | 1.92E-06 |
| adaptive immune response | 57 | 8.28E-09 | 4.67E-06 |
| organic substance biosynthetic process | 209 | 1.07E-08 | 5.84E-06 |
| response to external stimulus | 136 | 1.15E-08 | 6.02E-06 |
| energy derivation by oxidation of organic compounds | 31 | 1.30E-08 | 6.60E-06 |
| detection of chemical stimulus involved in sensory perception | 0 | 1.48E-08 | 7.30E-06 |
| gene expression | 144 | 2.01E-08 | 9.57E-06 |
| ribose phosphate biosynthetic process | 27 | 2.79E-08 | 1.29E-05 |
| cellular metabolic process | 306 | 3.00E-08 | 1.31E-05 |
| biosynthetic process | 209 | 2.95E-08 | 1.32E-05 |
| defense response to bacterium | 33 | 3.86E-08 | 1.64E-05 |
| electron transport chain | 23 | 4.90E-08 | 2.02E-05 |
| ribonucleotide biosynthetic process | 26 | 5.55E-08 | 2.23E-05 |
| nucleotide biosynthetic process | 30 | 6.58E-08 | 2.57E-05 |
| nucleoside phosphate biosynthetic process | 30 | 7.76E-08 | 2.96E-05 |
| Unclassified | 56 | 9.41E-08 | 3.41E-05 |
| biological_process | 699 | 9.41E-08 | 3.50E-05 |
| metabolic process | 363 | 1.14E-07 | 3.96E-05 |
| detection of chemical stimulus involved in sensory perception of smell | 0 | 1.13E-07 | 4.00E-05 |
| purine ribonucleotide biosynthetic process | 24 | 1.81E-07 | 6.11E-05 |
| defense response to symbiont | 66 | 1.86E-07 | 6.18E-05 |
| defense response to other organism | 71 | 2.14E-07 | 6.94E-05 |
| ribosomal small subunit biogenesis | 17 | 2.62E-07 | 8.32E-05 |
| macromolecule biosynthetic process | 159 | 3.34E-07 | 1.04E-04 |
| purine nucleotide biosynthetic process | 26 | 3.77E-07 | 1.15E-04 |
| production of molecular mediator of immune response | 20 | 4.44E-07 | 1.33E-04 |
| inflammatory response | 46 | 4.87E-07 | 1.43E-04 |
| ribonucleoprotein complex biogenesis | 40 | 5.47E-07 | 1.57E-04 |
| defense response | 90 | 6.45E-07 | 1.73E-04 |
| sensory perception of chemical stimulus | 2 | 6.38E-07 | 1.74E-04 |
| sensory perception of smell | 1 | 6.62E-07 | 1.74E-04 |
| mitochondrial electron transport, NADH to ubiquinone | 11 | 6.18E-07 | 1.74E-04 |
| innate immune response | 57 | 6.37E-07 | 1.77E-04 |
| response to other organism | 87 | 7.85E-07 | 2.03E-04 |
| response to biotic stimulus | 89 | 8.17E-07 | 2.04E-04 |
| response to external biotic stimulus | 87 | 8.12E-07 | 2.06E-04 |
| purine-containing compound biosynthetic process | 26 | 8.60E-07 | 2.11E-04 |
| mitochondrial respiratory chain complex assembly | 16 | 9.11E-07 | 2.20E-04 |
| mitochondrial electron transport, cytochrome c to oxygen | 8 | 1.37E-06 | 3.27E-04 |
| detection of stimulus involved in sensory perception | 3 | 2.05E-06 | 4.80E-04 |
| ribosome biogenesis | 30 | 2.09E-06 | 4.83E-04 |
| mitochondrial respiratory chain complex I assembly | 12 | 2.17E-06 | 4.86E-04 |
| NADH dehydrogenase complex assembly | 12 | 2.17E-06 | 4.93E-04 |
| ribonucleoside triphosphate metabolic process | 22 | 2.56E-06 | 5.64E-04 |
| nucleoside triphosphate metabolic process | 23 | 2.79E-06 | 6.07E-04 |
| protein metabolic process | 180 | 2.84E-06 | 6.10E-04 |
| response to stress | 174 | 3.32E-06 | 7.04E-04 |
| response to stimulus | 362 | 3.62E-06 | 7.55E-04 |
| regulation of cytokine production | 55 | 3.95E-06 | 8.14E-04 |
| immunoglobulin production | 18 | 4.63E-06 | 9.42E-04 |
| nitrogen compound metabolic process | 294 | 4.83E-06 | 9.68E-04 |
| purine ribonucleoside triphosphate metabolic process | 21 | 5.00E-06 | 9.90E-04 |
| organic substance metabolic process | 332 | 6.09E-06 | 1.19E-03 |
| cellular nitrogen compound metabolic process | 167 | 6.24E-06 | 1.20E-03 |
| chemotaxis | 31 | 6.73E-06 | 1.28E-03 |
| nervous system process | 25 | 6.93E-06 | 1.30E-03 |
| taxis | 31 | 7.12E-06 | 1.32E-03 |
| ATP metabolic process | 19 | 8.04E-06 | 1.48E-03 |
| purine nucleoside triphosphate metabolic process | 21 | 8.93E-06 | 1.60E-03 |
| detection of chemical stimulus | 3 | 8.88E-06 | 1.61E-03 |
| antimicrobial humoral response | 18 | 1.01E-05 | 1.78E-03 |
| cell adhesion mediated by integrin | 9 | 1.39E-05 | 2.44E-03 |
| cellular process | 599 | 1.49E-05 | 2.59E-03 |
| locomotion | 31 | 1.72E-05 | 2.95E-03 |
| immune response-regulating signaling pathway | 30 | 2.35E-05 | 3.98E-03 |
| antimicrobial humoral immune response mediated by antimicrobial peptide | 14 | 2.64E-05 | 4.41E-03 |
| generation of precursor metabolites and energy | 33 | 2.75E-05 | 4.56E-03 |
| humoral immune response | 24 | 3.00E-05 | 4.91E-03 |
| biological process involved in interspecies interaction between organisms | 89 | 3.12E-05 | 5.06E-03 |
| regulation of inflammatory response | 31 | 3.97E-05 | 6.30E-03 |
| regulation of defense response | 52 | 3.95E-05 | 6.33E-03 |
| positive regulation of cytokine production | 37 | 4.55E-05 | 7.08E-03 |
| regulation of response to external stimulus | 67 | 4.54E-05 | 7.14E-03 |
| chromatin remodeling | 6 | 4.69E-05 | 7.14E-03 |
| leukocyte mediated immunity | 27 | 4.69E-05 | 7.21E-03 |
| cell activation | 49 | 5.54E-05 | 8.36E-03 |
| myeloid leukocyte activation | 17 | 5.65E-05 | 8.44E-03 |
| positive regulation of interleukin-1 beta production | 10 | 5.93E-05 | 8.77E-03 |
| macromolecule metabolic process | 256 | 6.50E-05 | 9.44E-03 |
| regulation of immune response | 58 | 6.46E-05 | 9.46E-03 |
| detection of stimulus | 8 | 7.54E-05 | 1.08E-02 |
| lymphocyte mediated immunity | 23 | 7.91E-05 | 1.13E-02 |
| positive regulation of defense response | 32 | 8.42E-05 | 1.19E-02 |
| proton transmembrane transport | 16 | 9.35E-05 | 1.31E-02 |
| regulation of immune system process | 86 | 9.66E-05 | 1.34E-02 |
| positive regulation of signal transduction by p53 class mediator | 7 | 1.05E-04 | 1.45E-02 |
| 7-methylguanosine cap hypermethylation | 4 | 1.12E-04 | 1.50E-02 |
| response to bacterium | 46 | 1.11E-04 | 1.51E-02 |
| immune effector process | 35 | 1.16E-04 | 1.54E-02 |
| positive regulation of response to external stimulus | 39 | 1.18E-04 | 1.55E-02 |
| organophosphate biosynthetic process | 39 | 1.16E-04 | 1.55E-02 |
| regulation of response to stress | 77 | 1.27E-04 | 1.65E-02 |
| positive regulation of cytosolic calcium ion concentration | 17 | 1.33E-04 | 1.70E-02 |
| cell killing | 17 | 1.33E-04 | 1.71E-02 |
| response to cytokine | 52 | 1.37E-04 | 1.73E-02 |
| antibacterial humoral response | 10 | 1.36E-04 | 1.73E-02 |
| complement receptor mediated signaling pathway | 5 | 1.45E-04 | 1.81E-02 |
| antibody-dependent cellular cytotoxicity | 4 | 1.95E-04 | 2.38E-02 |
| type IIa hypersensitivity | 4 | 1.95E-04 | 2.40E-02 |
| type II hypersensitivity | 4 | 1.95E-04 | 2.42E-02 |
| immunoglobulin mediated immune response | 18 | 2.00E-04 | 2.42E-02 |
| modulation of process of another organism | 5 | 2.04E-04 | 2.45E-02 |
| U2-type prespliceosome assembly | 6 | 2.32E-04 | 2.76E-02 |
| positive regulation of inflammatory response | 15 | 2.49E-04 | 2.94E-02 |
| blood coagulation | 17 | 2.51E-04 | 2.94E-02 |
| chromatin organization | 11 | 2.62E-04 | 3.05E-02 |
| ribonucleotide metabolic process | 30 | 2.68E-04 | 3.09E-02 |
| B cell mediated immunity | 18 | 2.77E-04 | 3.18E-02 |
| positive regulation of interleukin-1 production | 10 | 2.84E-04 | 3.23E-02 |
| coagulation | 17 | 2.87E-04 | 3.24E-02 |
| ribose phosphate metabolic process | 31 | 3.04E-04 | 3.41E-02 |
| immune response-regulating cell surface receptor signaling pathway | 23 | 3.51E-04 | 3.91E-02 |
| positive regulation of immune system process | 59 | 3.66E-04 | 4.04E-02 |
| hemostasis | 17 | 3.73E-04 | 4.10E-02 |
| carbohydrate derivative biosynthetic process | 40 | 3.85E-04 | 4.16E-02 |
| response to organic substance | 123 | 3.84E-04 | 4.18E-02 |
| positive regulation of response to stimulus | 113 | 4.30E-04 | 4.62E-02 |
| wound healing | 25 | 4.41E-04 | 4.70E-02 |
| energy coupled proton transmembrane transport, against electrochemical gradient | 3 | 4.64E-04 | 4.85E-02 |
| sensory perception | 17 | 4.61E-04 | 4.88E-02 |
| electron transport coupled proton transport | 3 | 4.64E-04 | 4.88E-02 |
| adaptive immune response based on somatic recombination of immune receptors built from immunoglobulin superfamily domains | 22 | 4.82E-04 | 5.00E-02 |
| *Molecular functions* | | | |
| structural constituent of ribosome | 52 | 6.75E-34 | 3.42E-30 |
| structural molecule activity | 74 | 2.39E-13 | 6.05E-10 |
| oxidoreduction-driven active transmembrane transporter activity | 14 | 1.13E-07 | 1.15E-04 |
| olfactory receptor activity | 0 | 1.10E-07 | 1.40E-04 |
| proton-transporting ATP synthase activity, rotational mechanism | 8 | 9.94E-08 | 1.68E-04 |
| binding | 661 | 6.91E-07 | 5.83E-04 |
| ubiquitin-protein transferase inhibitor activity | 6 | 1.82E-06 | 1.15E-03 |
| NADH dehydrogenase (quinone) activity | 10 | 2.10E-06 | 1.18E-03 |
| NADH dehydrogenase (ubiquinone) activity | 10 | 1.65E-06 | 1.20E-03 |
| NADH dehydrogenase activity | 10 | 3.31E-06 | 1.53E-03 |
| immune receptor activity | 19 | 3.13E-06 | 1.59E-03 |
| NAD(P)H dehydrogenase (quinone) activity | 10 | 4.12E-06 | 1.74E-03 |
| electron transfer activity | 16 | 5.18E-06 | 1.88E-03 |
| immunoglobulin receptor activity | 6 | 5.56E-06 | 1.88E-03 |
| proton channel activity | 8 | 5.10E-06 | 1.99E-03 |
| ubiquitin ligase inhibitor activity | 5 | 7.29E-06 | 2.31E-03 |
| RNA binding | 96 | 1.28E-05 | 3.82E-03 |
| IgG binding | 5 | 2.51E-05 | 7.08E-03 |
| immunoglobulin binding | 7 | 3.10E-05 | 8.26E-03 |
| oxidoreductase activity, acting on NAD(P)H, quinone or similar compound as acceptor | 10 | 3.75E-05 | 9.50E-03 |
| antigen binding | 20 | 4.59E-05 | 1.11E-02 |
| IgG receptor activity | 4 | 5.74E-05 | 1.32E-02 |
| Toll-like receptor binding | 5 | 6.58E-05 | 1.45E-02 |
| molecular_function | 703 | 7.48E-05 | 1.52E-02 |
| Unclassified | 52 | 7.48E-05 | 1.58E-02 |
| ubiquitin-protein transferase regulator activity | 7 | 8.43E-05 | 1.58E-02 |
| protein binding | 578 | 8.22E-05 | 1.60E-02 |
| primary active transmembrane transporter activity | 17 | 1.23E-04 | 2.23E-02 |
| pattern recognition receptor activity | 7 | 1.96E-04 | 3.21E-02 |
| protein-disulfide reductase (NAD(P)) activity | 4 | 1.95E-04 | 3.29E-02 |
| Toll-like receptor 4 binding | 3 | 1.91E-04 | 3.34E-02 |
| *Cellular components* | | | |
| cytosolic ribosome | 43 | 1.49E-30 | 2.97E-27 |
| ribosomal subunit | 52 | 3.17E-30 | 3.17E-27 |
| ribosome | 55 | 1.26E-27 | 8.42E-25 |
| cytosolic large ribosomal subunit | 23 | 4.44E-18 | 2.22E-15 |
| large ribosomal subunit | 30 | 2.16E-17 | 8.64E-15 |
| cytosolic small ribosomal subunit | 20 | 5.79E-17 | 1.93E-14 |
| mitochondrial protein-containing complex | 45 | 1.29E-15 | 3.69E-13 |
| inner mitochondrial membrane protein complex | 32 | 3.94E-15 | 9.84E-13 |
| small ribosomal subunit | 22 | 1.22E-13 | 2.72E-11 |
| ribonucleoprotein complex | 69 | 1.91E-13 | 3.81E-11 |
| extracellular region | 241 | 5.44E-13 | 9.89E-11 |
| specific granule | 29 | 9.08E-13 | 1.51E-10 |
| extracellular space | 196 | 2.51E-12 | 3.58E-10 |
| membrane | 460 | 2.42E-12 | 3.72E-10 |
| mitochondrial inner membrane | 53 | 4.52E-12 | 6.03E-10 |
| tertiary granule | 28 | 1.01E-11 | 1.26E-09 |
| organelle inner membrane | 55 | 3.04E-11 | 3.37E-09 |
| mitochondrial membrane | 68 | 2.90E-11 | 3.41E-09 |
| extracellular membrane-bounded organelle | 136 | 6.94E-11 | 6.30E-09 |
| mitochondrial envelope | 70 | 6.26E-11 | 6.59E-09 |
| extracellular organelle | 136 | 6.94E-11 | 6.60E-09 |
| extracellular vesicle | 136 | 6.81E-11 | 6.80E-09 |
| secretory granule | 74 | 9.16E-11 | 7.96E-09 |
| respiratory chain complex | 20 | 1.29E-10 | 1.03E-08 |
| extracellular exosome | 134 | 1.25E-10 | 1.04E-08 |
| vesicle | 219 | 1.46E-10 | 1.12E-08 |
| mitochondrial respirasome | 20 | 2.36E-10 | 1.75E-08 |
| respirasome | 20 | 1.06E-09 | 7.55E-08 |
| secretory vesicle | 80 | 2.12E-09 | 1.46E-07 |
| protein-containing complex | 313 | 8.60E-09 | 5.73E-07 |
| secretory granule membrane | 34 | 2.96E-08 | 1.91E-06 |
| mitochondrial proton-transporting ATP synthase complex | 9 | 3.70E-08 | 2.31E-06 |
| proton-transporting ATP synthase complex | 9 | 5.88E-08 | 3.56E-06 |
| organelle membrane | 197 | 9.26E-08 | 5.44E-06 |
| cellular anatomical entity | 725 | 1.94E-07 | 1.11E-05 |
| cytochrome complex | 11 | 2.27E-07 | 1.26E-05 |
| tertiary granule membrane | 14 | 3.50E-07 | 1.89E-05 |
| Unclassified | 27 | 4.07E-07 | 2.09E-05 |
| cellular_component | 728 | 4.07E-07 | 2.14E-05 |
| cell periphery | 301 | 4.37E-07 | 2.19E-05 |
| cytoplasmic vesicle lumen | 32 | 4.60E-07 | 2.24E-05 |
| mitochondrion | 102 | 4.74E-07 | 2.26E-05 |
| vesicle lumen | 32 | 4.92E-07 | 2.29E-05 |
| tertiary granule lumen | 12 | 5.57E-07 | 2.53E-05 |
| specific granule membrane | 15 | 1.04E-06 | 4.62E-05 |
| immunoglobulin complex | 22 | 1.65E-06 | 7.15E-05 |
| specific granule lumen | 12 | 2.17E-06 | 9.22E-05 |
| organelle envelope | 81 | 2.48E-06 | 1.01E-04 |
| envelope | 81 | 2.48E-06 | 1.03E-04 |
| membrane-bounded organelle | 546 | 2.67E-06 | 1.07E-04 |
| secretory granule lumen | 30 | 2.88E-06 | 1.13E-04 |
| proton-transporting ATP synthase complex, coupling factor F(o) | 6 | 5.56E-06 | 2.14E-04 |
| cytoplasmic vesicle | 134 | 9.99E-06 | 3.77E-04 |
| membrane protein complex | 85 | 1.03E-05 | 3.81E-04 |
| intracellular membrane-bounded organelle | 504 | 1.39E-05 | 4.64E-04 |
| mitochondrial respiratory chain complex I | 10 | 1.35E-05 | 4.66E-04 |
| cytoplasm | 504 | 1.39E-05 | 4.72E-04 |
| intracellular vesicle | 134 | 1.30E-05 | 4.73E-04 |
| NADH dehydrogenase complex | 10 | 1.35E-05 | 4.74E-04 |
| small-subunit processome | 12 | 1.47E-05 | 4.82E-04 |
| respiratory chain complex I | 10 | 1.35E-05 | 4.83E-04 |
| organelle | 571 | 1.55E-05 | 5.00E-04 |
| mitochondrial respiratory chain complex IV | 7 | 1.74E-05 | 5.51E-04 |
| proton-transporting two-sector ATPase complex | 10 | 1.93E-05 | 6.04E-04 |
| plasma membrane | 272 | 2.04E-05 | 6.27E-04 |
| mitochondrial proton-transporting ATP synthase complex, coupling factor F(o) | 5 | 2.51E-05 | 7.50E-04 |
| oxidoreductase complex | 16 | 2.48E-05 | 7.51E-04 |
| intracellular organelle | 542 | 2.71E-05 | 7.85E-04 |
| platelet alpha granule | 13 | 2.68E-05 | 7.87E-04 |
| canonical inflammasome complex | 6 | 3.03E-05 | 8.65E-04 |
| side of membrane | 50 | 3.46E-05 | 9.76E-04 |
| respiratory chain complex IV | 7 | 4.05E-05 | 1.12E-03 |
| external side of plasma membrane | 32 | 8.24E-05 | 2.26E-03 |
| cell surface | 57 | 1.05E-04 | 2.84E-03 |
| focal adhesion | 32 | 1.14E-04 | 3.04E-03 |
| endomembrane system | 221 | 1.32E-04 | 3.48E-03 |
| external encapsulating structure | 39 | 1.46E-04 | 3.69E-03 |
| mitochondrial respiratory chain complex III | 5 | 1.45E-04 | 3.70E-03 |
| respiratory chain complex III | 5 | 1.45E-04 | 3.75E-03 |
| AIM2 inflammasome complex | 3 | 1.91E-04 | 4.71E-03 |
| IPAF inflammasome complex | 3 | 1.91E-04 | 4.77E-03 |
| cell-substrate junction | 32 | 2.23E-04 | 5.44E-03 |
| anchoring junction | 55 | 2.63E-04 | 6.35E-03 |
| U2 snRNP | 6 | 2.92E-04 | 6.87E-03 |
| proton-transporting two-sector ATPase complex, proton-transporting domain | 6 | 2.92E-04 | 6.96E-03 |
| extracellular matrix | 38 | 3.47E-04 | 8.06E-03 |
| SMN-Sm protein complex | 5 | 3.76E-04 | 8.64E-03 |
| chromatin | 27 | 4.02E-04 | 9.14E-03 |
| protein-DNA complex | 29 | 4.16E-04 | 9.34E-03 |
| peptidase inhibitor complex | 4 | 4.81E-04 | 1.05E-02 |
| U4 snRNP | 4 | 4.81E-04 | 1.06E-02 |
| U1 snRNP | 5 | 4.95E-04 | 1.06E-02 |
| ficolin-1-rich granule | 17 | 4.81E-04 | 1.07E-02 |
| U12-type spliceosomal complex | 6 | 5.49E-04 | 1.17E-02 |
| spliceosomal snRNP complex | 9 | 6.51E-04 | 1.35E-02 |
| precatalytic spliceosome | 8 | 6.47E-04 | 1.36E-02 |
| preribosome | 12 | 7.71E-04 | 1.59E-02 |
| integrin complex | 6 | 8.00E-04 | 1.63E-02 |
| NLRP1 inflammasome complex | 3 | 9.04E-04 | 1.81E-02 |
| pICln-Sm protein complex | 3 | 9.04E-04 | 1.82E-02 |
| collagen-containing extracellular matrix | 30 | 9.59E-04 | 1.90E-02 |
| methylosome | 4 | 9.84E-04 | 1.93E-02 |
| protein complex involved in cell adhesion | 8 | 1.19E-03 | 2.29E-02 |
| Sm-like protein family complex | 10 | 1.19E-03 | 2.30E-02 |
| cytolytic granule lumen | 2 | 1.34E-03 | 2.51E-02 |
| calprotectin complex | 2 | 1.34E-03 | 2.53E-02 |
| cytoplasmic vesicle membrane | 67 | 1.37E-03 | 2.53E-02 |
| cytolytic granule | 4 | 1.34E-03 | 2.55E-02 |
| proton-transporting ATP synthase complex, catalytic core F(1) | 3 | 1.54E-03 | 2.82E-02 |
| U4/U6 x U5 tri-snRNP complex | 6 | 1.56E-03 | 2.83E-02 |
| ficolin-1-rich granule membrane | 8 | 1.66E-03 | 2.97E-02 |
| small nuclear ribonucleoprotein complex | 9 | 1.65E-03 | 2.97E-02 |
| spliceosomal tri-snRNP complex | 6 | 1.81E-03 | 3.20E-02 |
| vesicle membrane | 67 | 1.88E-03 | 3.30E-02 |
| U2-type precatalytic spliceosome | 7 | 2.19E-03 | 3.81E-02 |
| protease inhibitor complex | 3 | 2.39E-03 | 4.09E-02 |
| proteasome core complex, alpha-subunit complex | 3 | 2.39E-03 | 4.13E-02 |
| membrane-enclosed lumen | 244 | 2.76E-03 | 4.60E-02 |
| intracellular organelle lumen | 244 | 2.76E-03 | 4.63E-02 |
| organelle lumen | 244 | 2.76E-03 | 4.67E-02 |
| platelet alpha granule membrane | 4 | 2.91E-03 | 4.81E-02 |
| platelet alpha granule lumen | 8 | 3.04E-03 | 4.98E-02 |
| ***MDD responders with CRP<1 vs. non-responders/unmedicated with CRP>1*** | | | |
| *Biological processes* | | | |
| immune system process | 104 | 1.65E-16 | 1.25E-12 |
| immune response | 85 | 1.61E-16 | 2.46E-12 |
| response to stimulus | 221 | 5.00E-10 | 2.54E-06 |
| mucosal immune response | 9 | 5.19E-09 | 1.98E-05 |
| negative regulation of viral genome replication | 11 | 7.58E-09 | 2.31E-05 |
| organ or tissue specific immune response | 9 | 1.70E-08 | 2.87E-05 |
| biological process involved in interspecies interaction between organisms | 64 | 1.59E-08 | 3.02E-05 |
| response to external biotic stimulus | 59 | 1.45E-08 | 3.16E-05 |
| response to other organism | 59 | 1.37E-08 | 3.49E-05 |
| regulation of viral life cycle | 16 | 2.58E-08 | 3.57E-05 |
| response to biotic stimulus | 60 | 2.35E-08 | 3.58E-05 |
| defense response | 60 | 2.84E-08 | 3.60E-05 |
| negative regulation of viral process | 13 | 4.60E-08 | 5.39E-05 |
| defense response to other organism | 47 | 6.99E-08 | 7.61E-05 |
| regulation of defense response | 39 | 9.85E-08 | 9.38E-05 |
| regulation of viral genome replication | 12 | 9.48E-08 | 9.63E-05 |
| defense response to symbiont | 43 | 1.12E-07 | 1.01E-04 |
| innate immune response in mucosa | 7 | 1.36E-07 | 1.15E-04 |
| regulation of viral process | 16 | 2.22E-07 | 1.53E-04 |
| defense response to bacterium | 22 | 1.95E-07 | 1.56E-04 |
| cytoplasmic translation | 14 | 2.15E-07 | 1.56E-04 |
| response to external stimulus | 80 | 2.10E-07 | 1.60E-04 |
| regulation of inflammatory response | 24 | 5.20E-07 | 3.45E-04 |
| antibacterial humoral response | 10 | 5.73E-07 | 3.64E-04 |
| response to bacterium | 34 | 7.91E-07 | 4.83E-04 |
| antimicrobial humoral immune response mediated by antimicrobial peptide | 12 | 8.92E-07 | 5.23E-04 |
| regulation of immune system process | 58 | 1.24E-06 | 6.98E-04 |
| antimicrobial humoral response | 14 | 1.50E-06 | 7.64E-04 |
| innate immune response | 36 | 1.47E-06 | 7.73E-04 |
| regulation of response to external stimulus | 46 | 1.43E-06 | 7.77E-04 |
| defense response to fungus | 9 | 1.72E-06 | 8.47E-04 |
| defense response to virus | 19 | 1.86E-06 | 8.85E-04 |
| positive regulation of inflammatory response | 13 | 4.77E-06 | 2.14E-03 |
| response to virus | 22 | 4.73E-06 | 2.18E-03 |
| positive regulation of defense response | 23 | 7.13E-06 | 3.10E-03 |
| response to fungus | 9 | 7.58E-06 | 3.21E-03 |
| regulation of response to stress | 50 | 1.51E-05 | 6.06E-03 |
| defense response to Gram-negative bacterium | 10 | 1.51E-05 | 6.23E-03 |
| response to stress | 100 | 1.83E-05 | 7.16E-03 |
| cell surface receptor signaling pathway | 68 | 2.44E-05 | 9.29E-03 |
| negative regulation of cysteine-type endopeptidase activity | 8 | 2.98E-05 | 1.11E-02 |
| positive regulation of response to external stimulus | 26 | 5.19E-05 | 1.84E-02 |
| humoral immune response | 16 | 5.16E-05 | 1.87E-02 |
| myeloid leukocyte activation | 12 | 5.49E-05 | 1.90E-02 |
| granulocyte activation | 6 | 6.33E-05 | 2.14E-02 |
| disruption of plasma membrane integrity in another organism | 3 | 7.17E-05 | 2.28E-02 |
| ERBB2-ERBB4 signaling pathway | 3 | 7.17E-05 | 2.33E-02 |
| disruption of cellular anatomical structure in another organism | 3 | 7.17E-05 | 2.38E-02 |
| cell chemotaxis | 14 | 8.98E-05 | 2.79E-02 |
| detection of chemical stimulus involved in sensory perception | 0 | 9.54E-05 | 2.91E-02 |
| locomotion | 19 | 1.25E-04 | 3.74E-02 |
| positive regulation of exit from mitosis | 3 | 1.41E-04 | 4.14E-02 |
| chemotaxis | 18 | 1.65E-04 | 4.73E-02 |
| taxis | 18 | 1.77E-04 | 4.99E-02 |
| leukocyte chemotaxis | 11 | 1.80E-04 | 5.00E-02 |
| *Molecular functions* | | | |
| structural constituent of ribosome | 15 | 1.12E-06 | 5.68E-03 |
| hemoglobin binding | 4 | 1.67E-05 | 4.23E-02 |
| Toll-like receptor 4 binding | 3 | 2.91E-05 | 4.92E-02 |
| *Cellular components* | | | |
| specific granule lumen | 11 | 2.85E-08 | 2.85E-05 |
| specific granule | 17 | 1.67E-08 | 3.34E-05 |
| cytosolic ribosome | 14 | 1.45E-07 | 9.63E-05 |
| cytosolic large ribosomal subunit | 10 | 2.27E-07 | 1.14E-04 |
| vesicle lumen | 22 | 5.42E-07 | 1.55E-04 |
| secretory granule lumen | 22 | 3.96E-07 | 1.58E-04 |
| cytoplasmic vesicle lumen | 22 | 5.15E-07 | 1.71E-04 |
| secretory vesicle | 45 | 2.21E-06 | 5.53E-04 |
| secretory granule | 39 | 3.63E-06 | 8.06E-04 |
| ribosomal subunit | 15 | 7.59E-06 | 1.52E-03 |
| tertiary granule | 13 | 2.11E-05 | 3.51E-03 |
| large ribosomal subunit | 11 | 2.02E-05 | 3.67E-03 |
| extracellular region | 119 | 3.08E-05 | 4.74E-03 |
| primary lysosome | 12 | 5.15E-05 | 6.87E-03 |
| cell periphery | 164 | 5.51E-05 | 6.88E-03 |
| azurophil granule | 12 | 5.15E-05 | 7.36E-03 |
| tertiary granule lumen | 7 | 9.28E-05 | 1.09E-02 |
| ribosome | 15 | 1.16E-04 | 1.29E-02 |
| pICln-Sm protein complex | 3 | 1.41E-04 | 1.49E-02 |
| extracellular membrane-bounded organelle | 66 | 1.75E-04 | 1.52E-02 |
| plasma membrane | 151 | 1.90E-04 | 1.58E-02 |
| extracellular organelle | 66 | 1.75E-04 | 1.59E-02 |
| vesicle | 109 | 1.66E-04 | 1.66E-02 |
| extracellular vesicle | 66 | 1.75E-04 | 1.66E-02 |
| extracellular space | 93 | 2.70E-04 | 2.16E-02 |
| cytoplasm | 273 | 3.24E-04 | 2.49E-02 |
| calprotectin complex | 2 | 3.80E-04 | 2.62E-02 |
| galectin complex | 2 | 3.80E-04 | 2.71E-02 |
| azurophil granule lumen | 8 | 3.78E-04 | 2.80E-02 |
| extracellular exosome | 63 | 5.99E-04 | 3.99E-02 |
| intracellular vesicle | 73 | 7.07E-04 | 4.42E-02 |
| cytoplasmic vesicle | 73 | 6.96E-04 | 4.49E-02 |
| haptoglobin-hemoglobin complex | 3 | 8.00E-04 | 4.85E-02 |
| ***MDD non-responders/unmedicated with CRP<1 vs. responders with CRP>1*** | | | |
| *Biological processes* | | | |
| response to external biotic stimulus | 130 | 7.48E-17 | 2.85E-13 |
| response to other organism | 130 | 6.60E-17 | 3.35E-13 |
| response to biotic stimulus | 133 | 6.51E-17 | 4.96E-13 |
| defense response | 134 | 5.69E-17 | 8.67E-13 |
| defense response to other organism | 104 | 6.83E-16 | 2.08E-12 |
| response to stress | 243 | 1.41E-15 | 3.58E-12 |
| response to external stimulus | 178 | 1.13E-14 | 2.46E-11 |
| defense response to symbiont | 93 | 3.63E-14 | 6.92E-11 |
| biological process involved in interspecies interaction between organisms | 132 | 1.44E-13 | 2.44E-10 |
| innate immune response | 81 | 2.30E-13 | 3.50E-10 |
| immune system process | 175 | 2.96E-13 | 4.10E-10 |
| immune response | 133 | 1.51E-11 | 1.92E-08 |
| positive regulation of defense response | 50 | 7.47E-11 | 8.76E-08 |
| regulation of defense response | 76 | 9.69E-11 | 1.05E-07 |
| respiratory electron transport chain | 24 | 1.47E-10 | 1.50E-07 |
| organonitrogen compound metabolic process | 285 | 3.32E-10 | 3.16E-07 |
| ATP synthesis coupled electron transport | 21 | 4.45E-10 | 3.77E-07 |
| mitochondrial ATP synthesis coupled electron transport | 21 | 4.45E-10 | 3.99E-07 |
| regulation of immune response | 82 | 8.42E-10 | 6.75E-07 |
| electron transport chain | 28 | 9.54E-10 | 7.27E-07 |
| regulation of cytokine production | 73 | 1.15E-09 | 8.35E-07 |
| regulation of response to biotic stimulus | 55 | 1.48E-09 | 1.02E-06 |
| oxidative phosphorylation | 23 | 2.08E-09 | 1.38E-06 |
| response to organic substance | 168 | 3.56E-09 | 2.17E-06 |
| regulation of innate immune response | 48 | 3.49E-09 | 2.22E-06 |
| detection of chemical stimulus involved in sensory perception of smell | 0 | 4.67E-09 | 2.74E-06 |
| positive regulation of response to external stimulus | 56 | 5.25E-09 | 2.96E-06 |
| response to bacterium | 65 | 6.41E-09 | 3.49E-06 |
| aerobic electron transport chain | 19 | 6.65E-09 | 3.49E-06 |
| cytoplasmic translation | 23 | 7.63E-09 | 3.88E-06 |
| regulation of response to external stimulus | 90 | 8.79E-09 | 4.32E-06 |
| positive regulation of inflammatory response | 24 | 1.46E-08 | 6.98E-06 |
| biological_process | 833 | 3.81E-08 | 1.71E-05 |
| positive regulation of cytokine production | 50 | 4.01E-08 | 1.74E-05 |
| Unclassified | 70 | 3.81E-08 | 1.76E-05 |
| regulation of signaling | 213 | 4.93E-08 | 2.03E-05 |
| response to cytokine | 71 | 4.93E-08 | 2.09E-05 |
| response to virus | 41 | 6.58E-08 | 2.57E-05 |
| regulation of cell communication | 213 | 6.44E-08 | 2.58E-05 |
| regulation of response to stress | 102 | 6.91E-08 | 2.63E-05 |
| regulation of response to stimulus | 239 | 1.04E-07 | 3.87E-05 |
| response to stimulus | 436 | 1.17E-07 | 4.25E-05 |
| defense response to virus | 33 | 1.29E-07 | 4.56E-05 |
| detection of chemical stimulus involved in sensory perception | 2 | 1.36E-07 | 4.72E-05 |
| positive regulation of immune response | 59 | 1.56E-07 | 5.28E-05 |
| inflammatory response | 53 | 1.62E-07 | 5.38E-05 |
| sensory perception of smell | 2 | 2.76E-07 | 8.76E-05 |
| defense response to bacterium | 35 | 2.70E-07 | 8.76E-05 |
| organonitrogen compound biosynthetic process | 100 | 3.17E-07 | 9.86E-05 |
| positive regulation of response to stimulus | 148 | 3.24E-07 | 9.88E-05 |
| cellular respiration | 26 | 4.44E-07 | 1.33E-04 |
| regulation of signal transduction | 187 | 5.19E-07 | 1.46E-04 |
| activation of immune response | 40 | 5.10E-07 | 1.47E-04 |
| positive regulation of canonical NF-kappaB signal transduction | 27 | 5.08E-07 | 1.49E-04 |
| response to molecule of bacterial origin | 37 | 5.62E-07 | 1.56E-04 |
| sensory perception of chemical stimulus | 4 | 6.76E-07 | 1.84E-04 |
| positive regulation of innate immune response | 32 | 7.68E-07 | 2.05E-04 |
| aerobic respiration | 23 | 8.29E-07 | 2.18E-04 |
| protein metabolic process | 212 | 1.05E-06 | 2.70E-04 |
| detection of stimulus involved in sensory perception | 5 | 1.86E-06 | 4.72E-04 |
| regulation of canonical NF-kappaB signal transduction | 31 | 2.08E-06 | 5.19E-04 |
| regulation of inflammatory response | 38 | 2.72E-06 | 6.70E-04 |
| positive regulation of response to biotic stimulus | 33 | 2.99E-06 | 7.25E-04 |
| innate immune response-activating signaling pathway | 20 | 3.56E-06 | 8.48E-04 |
| activation of innate immune response | 22 | 3.71E-06 | 8.70E-04 |
| cellular response to cytokine stimulus | 59 | 5.04E-06 | 1.15E-03 |
| cellular response to organic substance | 115 | 4.99E-06 | 1.15E-03 |
| regulation of immune system process | 105 | 5.30E-06 | 1.19E-03 |
| pattern recognition receptor signaling pathway | 18 | 5.48E-06 | 1.21E-03 |
| response to lipopolysaccharide | 33 | 5.89E-06 | 1.28E-03 |
| positive regulation of cell communication | 116 | 7.67E-06 | 1.65E-03 |
| positive regulation of signaling | 116 | 7.78E-06 | 1.65E-03 |
| immune response-regulating signaling pathway | 35 | 9.40E-06 | 1.96E-03 |
| defense response to protozoan | 8 | 1.02E-05 | 2.11E-03 |
| positive regulation of immune system process | 74 | 1.11E-05 | 2.27E-03 |
| negative regulation of complement activation | 6 | 1.55E-05 | 3.11E-03 |
| regulation of protein metabolic process | 135 | 1.65E-05 | 3.27E-03 |
| regulation of non-canonical NF-kappaB signal transduction | 15 | 1.79E-05 | 3.50E-03 |
| regulation of complement activation | 7 | 2.06E-05 | 3.98E-03 |
| nucleoside triphosphate biosynthetic process | 16 | 2.13E-05 | 4.06E-03 |
| response to abiotic stimulus | 79 | 2.20E-05 | 4.14E-03 |
| response to protozoan | 8 | 2.50E-05 | 4.59E-03 |
| response to lipid | 64 | 2.49E-05 | 4.63E-03 |
| nitrogen compound metabolic process | 340 | 2.75E-05 | 4.99E-03 |
| ribonucleoside triphosphate biosynthetic process | 15 | 3.33E-05 | 5.96E-03 |
| cellular response to biotic stimulus | 25 | 3.56E-05 | 6.31E-03 |
| cellular process | 709 | 3.67E-05 | 6.43E-03 |
| cellular response to chemical stimulus | 145 | 4.09E-05 | 7.09E-03 |
| immune response-activating signaling pathway | 31 | 4.31E-05 | 7.29E-03 |
| metabolic process | 409 | 4.29E-05 | 7.35E-03 |
| G protein-coupled receptor signaling pathway | 28 | 4.43E-05 | 7.42E-03 |
| translation | 36 | 5.24E-05 | 8.69E-03 |
| mitochondrial electron transport, cytochrome c to oxygen | 7 | 5.47E-05 | 8.97E-03 |
| response to type II interferon | 17 | 6.63E-05 | 1.06E-02 |
| purine ribonucleoside triphosphate biosynthetic process | 14 | 6.58E-05 | 1.07E-02 |
| ATP biosynthetic process | 13 | 7.10E-05 | 1.13E-02 |
| purine nucleoside triphosphate biosynthetic process | 14 | 7.40E-05 | 1.16E-02 |
| regulation of interleukin-8 production | 13 | 8.05E-05 | 1.25E-02 |
| negative regulation of humoral immune response | 6 | 8.25E-05 | 1.27E-02 |
| negative regulation of complement activation, alternative pathway | 3 | 8.41E-05 | 1.28E-02 |
| cellular response to stimulus | 337 | 8.52E-05 | 1.29E-02 |
| response to glucocorticoid | 17 | 8.82E-05 | 1.32E-02 |
| cellular response to mechanical stimulus | 12 | 9.61E-05 | 1.42E-02 |
| hypersensitivity | 5 | 9.81E-05 | 1.42E-02 |
| peptide biosynthetic process | 37 | 9.75E-05 | 1.43E-02 |
| response to oxygen-containing compound | 100 | 1.03E-04 | 1.49E-02 |
| response to chemical | 211 | 1.07E-04 | 1.53E-02 |
| positive regulation of interleukin-8 production | 11 | 1.11E-04 | 1.56E-02 |
| cellular response to molecule of bacterial origin | 22 | 1.18E-04 | 1.65E-02 |
| energy derivation by oxidation of organic compounds | 26 | 1.19E-04 | 1.65E-02 |
| negative regulation of cytokine production | 28 | 1.30E-04 | 1.78E-02 |
| mitochondrial electron transport, NADH to ubiquinone | 9 | 1.47E-04 | 1.98E-02 |
| negative regulation of protein metabolic process | 59 | 1.47E-04 | 2.00E-02 |
| regulation of intracellular signal transduction | 111 | 1.64E-04 | 2.18E-02 |
| positive regulation of multicellular organismal process | 104 | 1.63E-04 | 2.18E-02 |
| adaptive immune response | 51 | 1.87E-04 | 2.44E-02 |
| response to corticosteroid | 18 | 1.87E-04 | 2.45E-02 |
| programmed cell death | 74 | 1.91E-04 | 2.46E-02 |
| positive regulation of tumor necrosis factor production | 14 | 1.97E-04 | 2.53E-02 |
| negative regulation of complement activation, classical pathway | 4 | 2.23E-04 | 2.83E-02 |
| positive regulation of signal transduction | 99 | 2.40E-04 | 3.03E-02 |
| apoptotic process | 71 | 2.48E-04 | 3.10E-02 |
| cell death | 74 | 2.55E-04 | 3.16E-02 |
| positive regulation of interleukin-1 beta production | 10 | 2.57E-04 | 3.16E-02 |
| amide biosynthetic process | 43 | 2.73E-04 | 3.33E-02 |
| positive regulation of tumor necrosis factor superfamily cytokine production | 14 | 2.93E-04 | 3.52E-02 |
| T cell mediated immunity | 8 | 2.92E-04 | 3.53E-02 |
| regulation of complement activation, alternative pathway | 3 | 3.25E-04 | 3.70E-02 |
| positive regulation of gene expression | 78 | 3.12E-04 | 3.72E-02 |
| ATP export | 3 | 3.25E-04 | 3.73E-02 |
| negative regulation of B cell mediated immunity | 5 | 3.33E-04 | 3.74E-02 |
| platelet activating factor biosynthetic process | 3 | 3.25E-04 | 3.76E-02 |
| regulation of nervous system process | 15 | 3.21E-04 | 3.76E-02 |
| negative regulation of immunoglobulin mediated immune response | 5 | 3.33E-04 | 3.76E-02 |
| detection of chemical stimulus | 8 | 3.39E-04 | 3.77E-02 |
| regulation of viral genome replication | 12 | 3.24E-04 | 3.77E-02 |
| cellular response to stress | 99 | 3.42E-04 | 3.77E-02 |
| proton motive force-driven ATP synthesis | 11 | 3.20E-04 | 3.79E-02 |
| protein monoubiquitination | 8 | 3.49E-04 | 3.80E-02 |
| positive regulation of cellular process | 298 | 3.48E-04 | 3.82E-02 |
| positive regulation of biological process | 321 | 3.68E-04 | 3.95E-02 |
| response to mechanical stimulus | 22 | 3.66E-04 | 3.96E-02 |
| regulation of complement activation, classical pathway | 4 | 3.88E-04 | 4.05E-02 |
| regulation of interferon-alpha production | 7 | 3.92E-04 | 4.06E-02 |
| antibody-dependent cellular cytotoxicity | 4 | 3.88E-04 | 4.08E-02 |
| type IIa hypersensitivity | 4 | 3.88E-04 | 4.10E-02 |
| type II hypersensitivity | 4 | 3.88E-04 | 4.13E-02 |
| regulation of adaptive immune response | 21 | 4.06E-04 | 4.18E-02 |
| nucleotide biosynthetic process | 25 | 4.21E-04 | 4.31E-02 |
| detection of stimulus | 13 | 4.30E-04 | 4.37E-02 |
| proton motive force-driven mitochondrial ATP synthesis | 10 | 4.41E-04 | 4.45E-02 |
| acute inflammatory response | 11 | 4.58E-04 | 4.56E-02 |
| nucleoside phosphate biosynthetic process | 25 | 4.57E-04 | 4.58E-02 |
| modulation of process of another organism | 5 | 4.67E-04 | 4.60E-02 |
| positive regulation of NF-kappaB transcription factor activity | 16 | 4.67E-04 | 4.63E-02 |
| regulation of adaptive immune response based on somatic recombination of immune receptors built from immunoglobulin superfamily domains | 20 | 4.84E-04 | 4.73E-02 |
| *Molecular functions* | | | |
| binding | 799 | 3.04E-10 | 1.54E-06 |
| protein binding | 714 | 9.27E-10 | 2.35E-06 |
| structural constituent of ribosome | 27 | 4.92E-09 | 6.24E-06 |
| olfactory receptor activity | 0 | 4.47E-09 | 7.55E-06 |
| Unclassified | 57 | 5.74E-07 | 4.85E-04 |
| molecular_function | 846 | 5.74E-07 | 5.82E-04 |
| oxidoreduction-driven active transmembrane transporter activity | 13 | 5.73E-06 | 4.15E-03 |
| electron transfer activity | 17 | 1.22E-05 | 6.85E-03 |
| pattern recognition receptor activity | 9 | 1.12E-05 | 7.11E-03 |
| mRNA 5'-UTR binding | 7 | 5.47E-05 | 2.77E-02 |
| *Cellular components* | | | |
| cytosolic ribosome | 25 | 1.34E-10 | 2.68E-07 |
| cytoplasm | 622 | 7.95E-10 | 7.94E-07 |
| tertiary granule | 27 | 2.87E-09 | 1.91E-06 |
| endomembrane system | 286 | 4.68E-09 | 2.34E-06 |
| specific granule | 26 | 7.45E-09 | 2.98E-06 |
| intracellular organelle | 663 | 1.12E-08 | 3.72E-06 |
| respiratory chain complex | 19 | 1.74E-08 | 4.36E-06 |
| vesicle | 244 | 1.61E-08 | 4.60E-06 |
| respirasome | 20 | 2.18E-08 | 4.85E-06 |
| intracellular membrane-bounded organelle | 613 | 4.16E-08 | 8.32E-06 |
| cytoplasmic vesicle | 167 | 4.58E-08 | 8.32E-06 |
| intracellular vesicle | 167 | 6.27E-08 | 1.04E-05 |
| membrane-bounded organelle | 656 | 7.50E-08 | 1.07E-05 |
| cytosolic small ribosomal subunit | 13 | 7.16E-08 | 1.10E-05 |
| ribosomal subunit | 27 | 1.37E-07 | 1.82E-05 |
| mitochondrial respirasome | 18 | 1.65E-07 | 2.06E-05 |
| intracellular anatomical structure | 723 | 1.91E-07 | 2.25E-05 |
| organelle | 688 | 2.70E-07 | 3.00E-05 |
| ribosome | 30 | 5.07E-07 | 5.33E-05 |
| cytochrome complex | 11 | 1.33E-06 | 1.33E-04 |
| specific granule lumen | 13 | 2.30E-06 | 2.09E-04 |
| inner mitochondrial membrane protein complex | 22 | 2.22E-06 | 2.12E-04 |
| organelle membrane | 219 | 5.72E-06 | 4.97E-04 |
| canonical inflammasome complex | 7 | 6.34E-06 | 5.28E-04 |
| cytosol | 303 | 6.80E-06 | 5.43E-04 |
| secretory granule | 69 | 8.75E-06 | 6.73E-04 |
| cytosolic large ribosomal subunit | 12 | 9.41E-06 | 6.97E-04 |
| respiratory chain complex IV | 8 | 1.40E-05 | 9.98E-04 |
| tertiary granule membrane | 13 | 1.53E-05 | 1.05E-03 |
| tertiary granule lumen | 11 | 2.21E-05 | 1.47E-03 |
| secretory vesicle | 77 | 3.38E-05 | 1.78E-03 |
| extracellular space | 193 | 3.04E-05 | 1.79E-03 |
| bounding membrane of organelle | 137 | 2.77E-05 | 1.79E-03 |
| extracellular membrane-bounded organelle | 132 | 3.36E-05 | 1.82E-03 |
| extracellular exosome | 131 | 3.03E-05 | 1.84E-03 |
| membrane protein complex | 96 | 2.97E-05 | 1.85E-03 |
| extracellular organelle | 132 | 3.36E-05 | 1.87E-03 |
| extracellular vesicle | 132 | 3.33E-05 | 1.90E-03 |
| cellular_component | 861 | 4.65E-05 | 2.33E-03 |
| Unclassified | 42 | 4.65E-05 | 2.38E-03 |
| membrane | 497 | 4.98E-05 | 2.43E-03 |
| small ribosomal subunit | 13 | 5.49E-05 | 2.49E-03 |
| protein-containing complex | 342 | 5.63E-05 | 2.50E-03 |
| mitochondrial inner membrane | 42 | 5.78E-05 | 2.51E-03 |
| mitochondrial respiratory chain complex IV | 7 | 5.47E-05 | 2.54E-03 |
| organelle inner membrane | 46 | 5.41E-05 | 2.57E-03 |
| cellular anatomical entity | 854 | 1.17E-04 | 4.98E-03 |
| cytoplasmic vesicle membrane | 83 | 1.38E-04 | 5.75E-03 |
| mitochondrial protein-containing complex | 28 | 2.01E-04 | 8.04E-03 |
| vesicle membrane | 83 | 2.00E-04 | 8.16E-03 |
| secretory granule membrane | 29 | 2.55E-04 | 9.99E-03 |
| AIM2 inflammasome complex | 3 | 3.25E-04 | 1.25E-02 |
| large ribosomal subunit | 14 | 6.63E-04 | 2.50E-02 |
| extracellular region | 230 | 6.92E-04 | 2.56E-02 |
| cytoplasmic side of rough endoplasmic reticulum membrane | 3 | 7.86E-04 | 2.86E-02 |
| cytoplasmic side of endoplasmic reticulum membrane | 5 | 8.52E-04 | 3.04E-02 |
| peptidase inhibitor complex | 4 | 9.46E-04 | 3.32E-02 |
| Golgi apparatus | 99 | 1.05E-03 | 3.62E-02 |
| Golgi membrane | 48 | 1.12E-03 | 3.80E-02 |
| transporter complex | 36 | 1.39E-03 | 4.62E-02 |
| growth cone | 17 | 1.59E-03 | 4.90E-02 |
| mitochondrial respiratory chain complex I | 8 | 1.58E-03 | 4.94E-02 |
| NLRP1 inflammasome complex | 3 | 1.52E-03 | 4.99E-02 |
| NADH dehydrogenase complex | 8 | 1.58E-03 | 5.02E-02 |
| respiratory chain complex I | 8 | 1.58E-03 | 5.10E-02 |
| ***MDD responders with CRP<1 vs. responders with CRP>1*** | | | |
| *Biological processes* | | | |
| defense response to virus | 43 | 6.19E-24 | 9.44E-20 |
| immune system process | 130 | 1.82E-23 | 1.39E-19 |
| immune response | 105 | 2.94E-22 | 1.49E-18 |
| defense response | 95 | 1.67E-21 | 6.36E-18 |
| response to virus | 45 | 4.08E-20 | 1.24E-16 |
| defense response to other organism | 75 | 1.28E-19 | 3.25E-16 |
| response to other organism | 88 | 5.66E-19 | 1.23E-15 |
| response to external biotic stimulus | 88 | 6.50E-19 | 1.24E-15 |
| response to biotic stimulus | 88 | 4.70E-18 | 7.95E-15 |
| response to external stimulus | 117 | 1.46E-17 | 2.23E-14 |
| defense response to symbiont | 67 | 1.73E-17 | 2.40E-14 |
| biological process involved in interspecies interaction between organisms | 90 | 9.86E-17 | 1.25E-13 |
| negative regulation of viral process | 22 | 2.31E-16 | 2.71E-13 |
| innate immune response | 58 | 8.20E-16 | 8.93E-13 |
| regulation of viral life cycle | 25 | 2.59E-15 | 2.47E-12 |
| negative regulation of viral genome replication | 17 | 2.57E-15 | 2.61E-12 |
| regulation of response to external stimulus | 69 | 1.22E-14 | 1.04E-11 |
| regulation of viral process | 26 | 1.22E-14 | 1.09E-11 |
| response to stress | 144 | 1.59E-14 | 1.28E-11 |
| regulation of defense response | 56 | 2.89E-14 | 2.20E-11 |
| regulation of viral genome replication | 18 | 5.42E-13 | 3.94E-10 |
| response to stimulus | 258 | 1.06E-11 | 7.31E-09 |
| negative regulation of biological process | 181 | 1.02E-10 | 6.75E-08 |
| regulation of cytokine production | 49 | 1.41E-10 | 8.96E-08 |
| regulation of response to biotic stimulus | 38 | 2.44E-10 | 1.49E-07 |
| regulation of response to stress | 69 | 2.82E-10 | 1.66E-07 |
| response to organic substance | 103 | 5.31E-10 | 3.00E-07 |
| response to cytokine | 49 | 7.96E-10 | 4.33E-07 |
| positive regulation of response to external stimulus | 37 | 4.73E-09 | 2.48E-06 |
| positive regulation of defense response | 31 | 6.12E-09 | 3.11E-06 |
| antiviral innate immune response | 11 | 1.53E-08 | 7.50E-06 |
| regulation of innate immune response | 31 | 1.85E-08 | 8.81E-06 |
| response to bacterium | 41 | 2.05E-08 | 9.48E-06 |
| response to type I interferon | 11 | 2.88E-08 | 1.29E-05 |
| positive regulation of cytokine production | 32 | 1.17E-07 | 5.12E-05 |
| cellular response to cytokine stimulus | 41 | 1.32E-07 | 5.58E-05 |
| defense response to bacterium | 24 | 1.71E-07 | 7.05E-05 |
| regulation of immune system process | 67 | 2.59E-07 | 1.04E-04 |
| negative regulation of multicellular organismal process | 53 | 3.99E-07 | 1.56E-04 |
| cellular response to chemical stimulus | 91 | 5.45E-07 | 2.08E-04 |
| inflammatory response | 33 | 6.70E-07 | 2.49E-04 |
| response to interferon-beta | 8 | 7.13E-07 | 2.59E-04 |
| negative regulation of response to external stimulus | 27 | 8.08E-07 | 2.86E-04 |
| positive regulation of inflammatory response | 15 | 9.29E-07 | 3.22E-04 |
| regulation of immune response | 45 | 1.17E-06 | 3.96E-04 |
| negative regulation of cellular process | 155 | 1.86E-06 | 6.16E-04 |
| positive regulation of biological process | 189 | 1.90E-06 | 6.17E-04 |
| regulation of inflammatory response | 25 | 2.20E-06 | 6.98E-04 |
| positive regulation of cellular process | 176 | 2.30E-06 | 7.16E-04 |
| response to chemical | 126 | 3.81E-06 | 1.16E-03 |
| cellular response to organic substance | 69 | 3.93E-06 | 1.18E-03 |
| negative regulation of defense response | 19 | 5.65E-06 | 1.57E-03 |
| regulation of cell communication | 116 | 5.65E-06 | 1.59E-03 |
| positive regulation of immune system process | 46 | 5.54E-06 | 1.59E-03 |
| regulation of signaling | 116 | 5.48E-06 | 1.61E-03 |
| positive regulation of innate immune response | 20 | 6.83E-06 | 1.86E-03 |
| regulation of multicellular organismal process | 103 | 6.99E-06 | 1.87E-03 |
| regulation of response to stimulus | 130 | 7.90E-06 | 2.08E-03 |
| cellular response to type I interferon | 8 | 8.07E-06 | 2.08E-03 |
| regulation of signal transduction | 104 | 8.44E-06 | 2.14E-03 |
| interleukin-27-mediated signaling pathway | 4 | 8.66E-06 | 2.16E-03 |
| regulation of defense response to virus | 9 | 1.73E-05 | 4.25E-03 |
| positive regulation of response to stimulus | 81 | 2.26E-05 | 5.46E-03 |
| positive regulation of response to biotic stimulus | 20 | 2.61E-05 | 6.13E-03 |
| positive regulation of acute inflammatory response | 6 | 2.60E-05 | 6.20E-03 |
| positive regulation of multicellular organismal process | 64 | 2.95E-05 | 6.81E-03 |
| cell chemotaxis | 16 | 3.43E-05 | 7.80E-03 |
| regulation of response to cytokine stimulus | 14 | 3.72E-05 | 8.34E-03 |
| regulation of catalytic activity | 56 | 3.85E-05 | 8.50E-03 |
| MDA-5 signaling pathway | 3 | 4.56E-05 | 9.93E-03 |
| sensory perception of smell | 0 | 4.76E-05 | 1.02E-02 |
| negative regulation of viral life cycle | 6 | 4.93E-05 | 1.04E-02 |
| regulation of molecular function | 73 | 5.09E-05 | 1.06E-02 |
| mucosal immune response | 6 | 5.99E-05 | 1.23E-02 |
| type I interferon-mediated signaling pathway | 7 | 6.35E-05 | 1.29E-02 |
| detection of chemical stimulus involved in sensory perception of smell | 0 | 6.52E-05 | 1.31E-02 |
| response to interferon-alpha | 5 | 6.87E-05 | 1.36E-02 |
| regulation of defense response to virus by host | 7 | 7.35E-05 | 1.40E-02 |
| cytokine-mediated signaling pathway | 22 | 7.31E-05 | 1.41E-02 |
| negative regulation of response to biotic stimulus | 11 | 7.26E-05 | 1.42E-02 |
| regulation of cytokine-mediated signaling pathway | 13 | 7.58E-05 | 1.43E-02 |
| regulation of pattern recognition receptor signaling pathway | 13 | 8.60E-05 | 1.60E-02 |
| positive regulation of defense response to virus by host | 6 | 1.03E-04 | 1.88E-02 |
| negative regulation of immune system process | 26 | 1.03E-04 | 1.90E-02 |
| regulation of biological process involved in symbiotic interaction | 8 | 1.07E-04 | 1.92E-02 |
| lipid storage | 7 | 1.11E-04 | 1.97E-02 |
| regulation of type I interferon production | 11 | 1.14E-04 | 2.00E-02 |
| organ or tissue specific immune response | 6 | 1.22E-04 | 2.12E-02 |
| regulation of viral entry into host cell | 7 | 1.27E-04 | 2.17E-02 |
| negative regulation of gene expression | 41 | 1.37E-04 | 2.32E-02 |
| negative regulation of cytokine production | 18 | 1.44E-04 | 2.41E-02 |
| response to yeast | 4 | 1.59E-04 | 2.60E-02 |
| negative regulation of innate immune response | 9 | 1.57E-04 | 2.61E-02 |
| response to fungus | 8 | 1.64E-04 | 2.66E-02 |
| negative regulation of metabolic process | 93 | 1.75E-04 | 2.75E-02 |
| negative regulation of viral entry into host cell | 5 | 1.75E-04 | 2.77E-02 |
| regulation of hormone biosynthetic process | 5 | 1.75E-04 | 2.80E-02 |
| negative regulation of macromolecule metabolic process | 87 | 1.84E-04 | 2.86E-02 |
| localization | 136 | 2.22E-04 | 3.34E-02 |
| ISG15-protein conjugation | 3 | 2.20E-04 | 3.36E-02 |
| lipid droplet formation | 4 | 2.18E-04 | 3.36E-02 |
| negative regulation of cell development | 17 | 2.52E-04 | 3.72E-02 |
| negative regulation of catalytic activity | 23 | 2.51E-04 | 3.76E-02 |
| regulation of hormone metabolic process | 6 | 2.63E-04 | 3.85E-02 |
| defense response to Gram-negative bacterium | 9 | 2.84E-04 | 4.12E-02 |
| modulation by symbiont of entry into host | 7 | 2.93E-04 | 4.21E-02 |
| acute-phase response | 6 | 3.02E-04 | 4.30E-02 |
| regeneration | 12 | 3.17E-04 | 4.48E-02 |
| acute inflammatory response | 8 | 3.22E-04 | 4.51E-02 |
| defense response to fungus | 7 | 3.27E-04 | 4.53E-02 |
| regulation of cell adhesion | 34 | 3.45E-04 | 4.74E-02 |
| regulation of chemokine production | 9 | 3.60E-04 | 4.89E-02 |
| *Molecular functions* | | | |
| double-stranded RNA binding | 10 | 5.66E-06 | 2.87E-02 |
| *Cellular components* | | | |
| specific granule lumen | 14 | 8.44E-11 | 1.69E-07 |
| secretory granule | 52 | 7.71E-10 | 5.14E-07 |
| secretory vesicle | 58 | 1.32E-09 | 6.62E-07 |
| specific granule | 20 | 6.68E-10 | 6.67E-07 |
| tertiary granule | 17 | 2.08E-07 | 8.33E-05 |
| tertiary granule lumen | 10 | 3.84E-07 | 1.28E-04 |
| secretory granule lumen | 22 | 4.66E-06 | 1.33E-03 |
| vesicle lumen | 22 | 6.27E-06 | 1.39E-03 |
| cytoplasmic vesicle lumen | 22 | 5.97E-06 | 1.49E-03 |
| endomembrane system | 150 | 1.13E-05 | 2.27E-03 |
| cytoplasm | 321 | 1.74E-05 | 2.67E-03 |
| cellular anatomical entity | 450 | 1.48E-05 | 2.68E-03 |
| ankyrin-1 complex | 4 | 1.70E-05 | 2.83E-03 |
| intracellular vesicle | 89 | 2.45E-05 | 3.26E-03 |
| cytoplasmic vesicle | 89 | 2.36E-05 | 3.37E-03 |
| cellular_component | 451 | 4.85E-05 | 5.71E-03 |
| Unclassified | 16 | 4.85E-05 | 6.06E-03 |
| cell periphery | 185 | 1.21E-04 | 1.35E-02 |
| vesicle | 122 | 3.77E-04 | 3.96E-02 |
| membrane | 264 | 4.33E-04 | 4.33E-02 |
| presynaptic intermediate filament cytoskeleton | 2 | 5.13E-04 | 4.89E-02 |
| ***MDD responders with CRP<1 vs. non-responders/unmedicated with CRP<1*** | | | |
| *Biological processes* | | | |
| adaptive immune response | 59 | 3.57E-17 | 5.45E-13 |
| immune response | 93 | 8.86E-14 | 6.76E-10 |
| immune system process | 114 | 2.49E-13 | 1.26E-09 |
| oxidative phosphorylation | 19 | 1.14E-10 | 4.35E-07 |
| aerobic electron transport chain | 16 | 4.08E-10 | 1.24E-06 |
| aerobic respiration | 21 | 7.02E-10 | 1.78E-06 |
| ATP synthesis coupled electron transport | 16 | 9.59E-10 | 1.83E-06 |
| mitochondrial ATP synthesis coupled electron transport | 16 | 9.59E-10 | 2.09E-06 |
| cellular respiration | 21 | 1.64E-08 | 2.77E-05 |
| respiratory electron transport chain | 16 | 2.29E-08 | 3.50E-05 |
| proton motive force-driven mitochondrial ATP synthesis | 11 | 4.14E-07 | 5.74E-04 |
| detection of stimulus involved in sensory perception | 0 | 1.26E-06 | 1.60E-03 |
| proton motive force-driven ATP synthesis | 11 | 1.62E-06 | 1.90E-03 |
| sensory perception of chemical stimulus | 0 | 1.82E-06 | 1.98E-03 |
| nucleoside triphosphate biosynthetic process | 13 | 2.84E-06 | 2.71E-03 |
| energy derivation by oxidation of organic compounds | 21 | 2.83E-06 | 2.88E-03 |
| cytoplasmic translation | 14 | 3.36E-06 | 3.01E-03 |
| purine ribonucleoside triphosphate biosynthetic process | 12 | 3.73E-06 | 3.16E-03 |
| electron transport chain | 16 | 3.94E-06 | 3.16E-03 |
| purine nucleoside triphosphate biosynthetic process | 12 | 4.17E-06 | 3.18E-03 |
| response to stimulus | 252 | 5.03E-06 | 3.65E-03 |
| ATP biosynthetic process | 11 | 6.64E-06 | 4.60E-03 |
| ribonucleoside triphosphate biosynthetic process | 12 | 7.12E-06 | 4.72E-03 |
| detection of stimulus | 2 | 8.09E-06 | 5.14E-03 |
| detection of chemical stimulus involved in sensory perception | 0 | 9.43E-06 | 5.53E-03 |
| cell surface receptor signaling pathway | 83 | 9.22E-06 | 5.62E-03 |
| sensory perception | 6 | 1.19E-05 | 6.70E-03 |
| sensory perception of smell | 0 | 1.33E-05 | 7.25E-03 |
| mitochondrial electron transport, NADH to ubiquinone | 8 | 1.46E-05 | 7.70E-03 |
| response to virus | 24 | 2.00E-05 | 8.97E-03 |
| nucleoside triphosphate metabolic process | 17 | 1.77E-05 | 9.00E-03 |
| mitochondrial electron transport, cytochrome c to oxygen | 6 | 2.00E-05 | 9.22E-03 |
| mitochondrial respiratory chain complex I assembly | 9 | 1.97E-05 | 9.39E-03 |
| NADH dehydrogenase complex assembly | 9 | 1.97E-05 | 9.69E-03 |
| nervous system process | 14 | 2.31E-05 | 1.00E-02 |
| purine nucleoside triphosphate metabolic process | 16 | 2.46E-05 | 1.04E-02 |
| detection of chemical stimulus involved in sensory perception of smell | 0 | 3.04E-05 | 1.25E-02 |
| detection of chemical stimulus | 1 | 4.48E-05 | 1.80E-02 |
| negative regulation of viral genome replication | 8 | 5.63E-05 | 2.20E-02 |
| purine ribonucleoside triphosphate metabolic process | 15 | 6.03E-05 | 2.30E-02 |
| purine ribonucleotide biosynthetic process | 15 | 9.17E-05 | 3.33E-02 |
| ribonucleoside triphosphate metabolic process | 15 | 9.17E-05 | 3.41E-02 |
| regulation of cell-cell adhesion mediated by integrin | 4 | 1.04E-04 | 3.70E-02 |
| negative regulation of viral process | 10 | 1.27E-04 | 4.40E-02 |
| *Molecular functions* | | | |
| oxidoreduction-driven active transmembrane transporter activity | 13 | 7.91E-09 | 4.01E-05 |
| structural constituent of ribosome | 18 | 1.87E-07 | 4.75E-04 |
| primary active transmembrane transporter activity | 16 | 3.37E-06 | 5.69E-03 |
| NADH dehydrogenase (quinone) activity | 8 | 7.22E-06 | 7.32E-03 |
| NADH dehydrogenase (ubiquinone) activity | 8 | 5.97E-06 | 7.57E-03 |
| NADH dehydrogenase activity | 8 | 1.04E-05 | 8.77E-03 |
| NAD(P)H dehydrogenase (quinone) activity | 8 | 1.24E-05 | 8.95E-03 |
| olfactory receptor activity | 0 | 2.99E-05 | 1.68E-02 |
| electron transfer activity | 12 | 2.94E-05 | 1.87E-02 |
| oxidoreductase activity, acting on NAD(P)H, quinone or similar compound as acceptor | 8 | 7.32E-05 | 3.37E-02 |
| MHC protein binding | 7 | 6.73E-05 | 3.41E-02 |
| G protein-coupled receptor activity | 6 | 8.62E-05 | 3.64E-02 |
| oxidoreduction-driven active transmembrane transporter activity | 13 | 7.91E-09 | 4.01E-05 |
| structural constituent of ribosome | 18 | 1.87E-07 | 4.75E-04 |
| primary active transmembrane transporter activity | 16 | 3.37E-06 | 5.69E-03 |
| NADH dehydrogenase (quinone) activity | 8 | 7.22E-06 | 7.32E-03 |
| NADH dehydrogenase (ubiquinone) activity | 8 | 5.97E-06 | 7.57E-03 |
| NADH dehydrogenase activity | 8 | 1.04E-05 | 8.77E-03 |
| NAD(P)H dehydrogenase (quinone) activity | 8 | 1.24E-05 | 8.95E-03 |
| olfactory receptor activity | 0 | 2.99E-05 | 1.68E-02 |
| electron transfer activity | 12 | 2.94E-05 | 1.87E-02 |
| oxidoreductase activity, acting on NAD(P)H, quinone or similar compound as acceptor | 8 | 7.32E-05 | 3.37E-02 |
| MHC protein binding | 7 | 6.73E-05 | 3.41E-02 |
| G protein-coupled receptor activity | 6 | 8.62E-05 | 3.64E-02 |
| *Cellular components* | | | |
| T cell receptor complex | 35 | 4.42E-24 | 8.83E-21 |
| plasma membrane signaling receptor complex | 39 | 5.76E-16 | 5.76E-13 |
| receptor complex | 42 | 9.38E-11 | 6.25E-08 |
| respiratory chain complex | 16 | 9.59E-10 | 4.79E-07 |
| mitochondrial respirasome | 16 | 1.56E-09 | 5.19E-07 |
| membrane protein complex | 74 | 1.36E-09 | 5.43E-07 |
| plasma membrane protein complex | 47 | 3.73E-09 | 1.06E-06 |
| respirasome | 16 | 5.21E-09 | 1.30E-06 |
| inner mitochondrial membrane protein complex | 19 | 1.78E-08 | 3.96E-06 |
| cytosolic ribosome | 15 | 4.10E-07 | 8.20E-05 |
| mitochondrial protein-containing complex | 24 | 5.95E-07 | 1.08E-04 |
| ribosomal subunit | 18 | 1.83E-06 | 3.05E-04 |
| protein-containing complex | 210 | 3.23E-06 | 4.97E-04 |
| cytochrome complex | 8 | 7.22E-06 | 1.03E-03 |
| mitochondrial respiratory chain complex IV | 6 | 2.00E-05 | 2.66E-03 |
| mitochondrial respiratory chain complex I | 8 | 3.21E-05 | 3.56E-03 |
| NADH dehydrogenase complex | 8 | 3.21E-05 | 3.77E-03 |
| respiratory chain complex I | 8 | 3.21E-05 | 4.01E-03 |
| respiratory chain complex IV | 6 | 4.12E-05 | 4.34E-03 |
| ribosome | 18 | 4.64E-05 | 4.64E-03 |
| BRCA1-B complex | 3 | 5.83E-05 | 5.29E-03 |
| mitochondrial inner membrane | 28 | 5.67E-05 | 5.39E-03 |
| cytosolic large ribosomal subunit | 8 | 1.06E-04 | 9.23E-03 |
| mitochondrial membrane | 37 | 1.27E-04 | 1.05E-02 |
| cytosolic small ribosomal subunit | 7 | 1.41E-04 | 1.13E-02 |
| membrane | 288 | 1.52E-04 | 1.17E-02 |
| large ribosomal subunit | 11 | 1.62E-04 | 1.20E-02 |
| mitochondrial envelope | 38 | 2.03E-04 | 1.45E-02 |
| organelle inner membrane | 29 | 2.33E-04 | 1.61E-02 |
| oxidoreductase complex | 11 | 4.03E-04 | 2.68E-02 |
| Golgi to plasma membrane transport vesicle | 2 | 6.05E-04 | 3.56E-02 |
| plasma membrane raft | 10 | 5.55E-04 | 3.58E-02 |
| BRCA1-BARD1 complex | 2 | 6.05E-04 | 3.67E-02 |
| cellular anatomical entity | 483 | 6.03E-04 | 3.77E-02 |
| PANTHER overrepresentation tests on transcripts differentially expressed (FDR p-adjusted <0.1) using the Gene Ontology (GO) database (DOI: 10.5281/zenodo.10536401; released 2024-01-17). Fisher's exact test with FDR correction (<0.05); *homo sapiens* analysed and reference gene lists. No. of genes: number of transcripts in the analysed comparison matching to a biological process, molecular function, or cellular component. Results are sorted based on their FDR-adjusted p-values (smaller to the top). | | | |


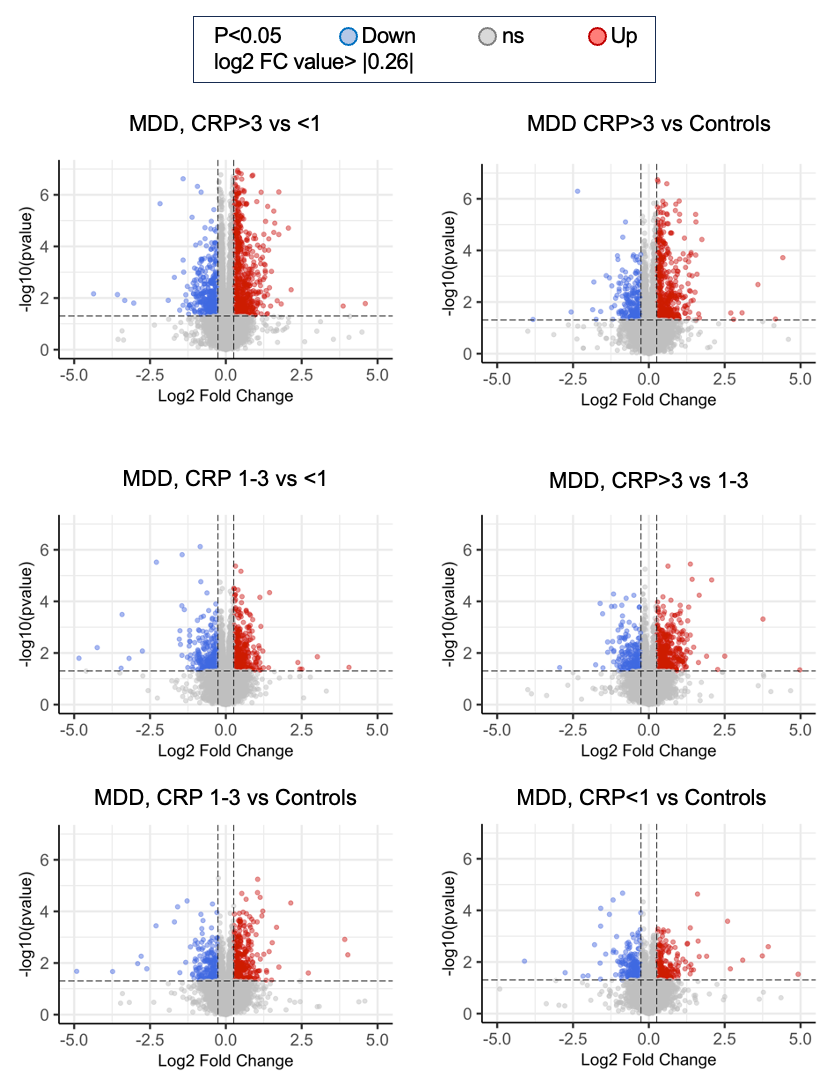


# ***Supplementary Figure S1:*** *Volcano plots of differentially expressed transcripts (p<0.05, FC>|1.2|) in CRP-based group comparisons*

Each dot represents a transcript comparing the conditions stated in the heading. The horizontal line corresponds to an uncorrected significance value of <0.05. The vertical line corresponds to a log2 value of |0.26| corresponding to a FC value of |1.2|.


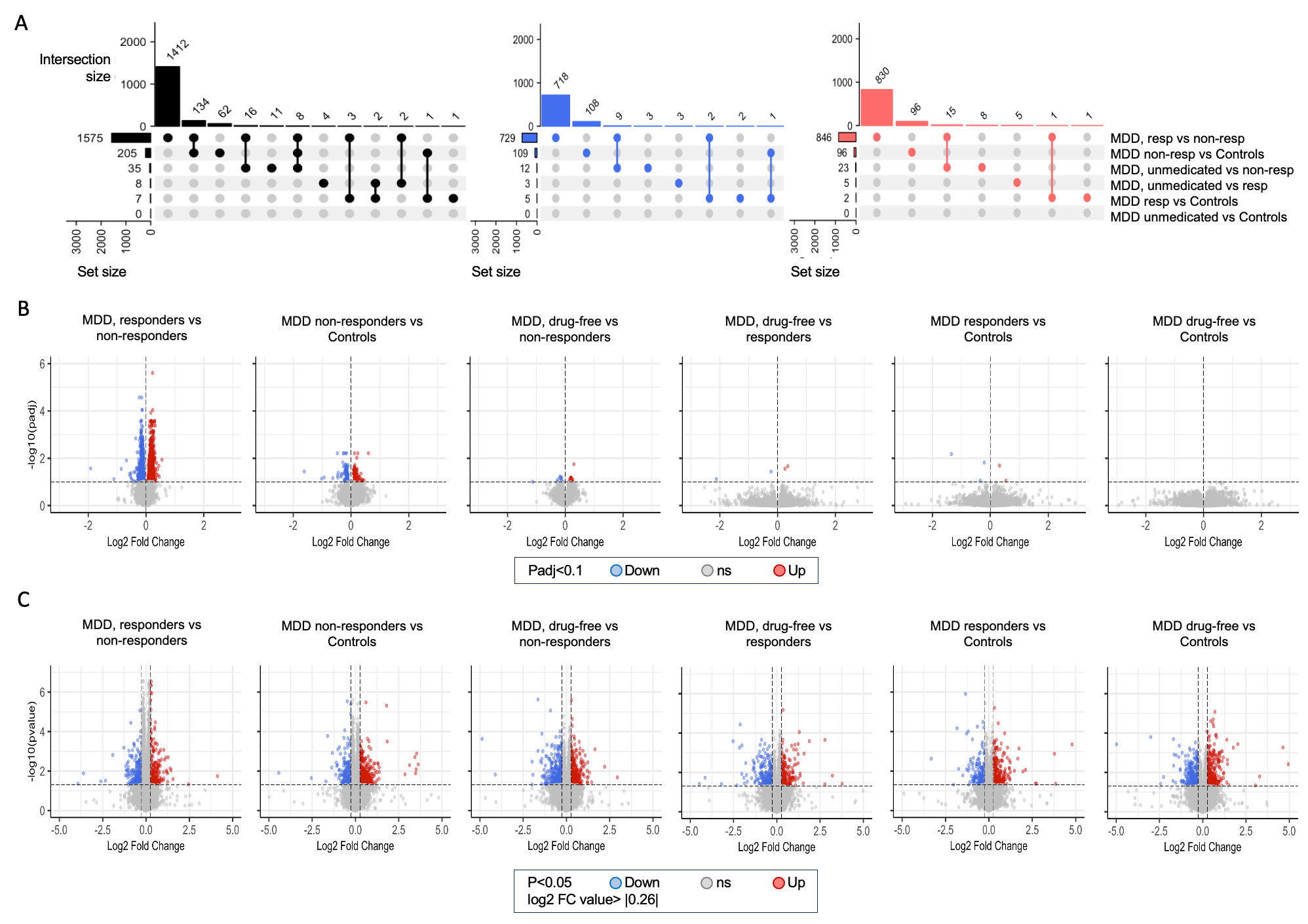


# ***Supplementary Figure S2:*** *Differentially expressed transcripts in treatment-based group comparisons*

**A)** UpSet plots to summarise key differentially expressed (DE) transcripts (FDR p-adjusted <0.1). These panels summarise the DE transcript overlap between comparisons for up-or down-regulated DE transcripts (in black), for down-regulated DE transcripts (in blue) and, upregulated DE transcripts (in red). In each panel, the bottom left horizontal bar graph labeled Set Size shows the total number of DE transcripts per comparison. The circles in each panel's matrix represent what would be the different Venn diagram sections (unique and overlapping DE transcripts). Connected circles indicate a certain intersection of DE transcripts between comparisons. The top bar graph in each panel summarises the number of DE genes for each unique or overlapping combination. In the top left panel, for example, the first vertical bar shows those DE transcripts that are unique to MDD treatment responders vs. MDD treatment non-responders (1412 DE transcripts). The second shows those DE transcripts that are shared only between MDD treatment responders vs. non-responders and MDD non-responders vs. Controls (134 DE transcripts).

**B-C)** Volcano plots of RNA-seq expression analysis. Each dot represents a transcript comparing the conditions stated in the heading. The horizontal line corresponds to a Benjamini-Hochberg FDR-adjusted significance value of <0.1 **(B)** or to an uncorrected significance value of <0.05 **(C)**. The vertical lines in C correspond to a log2 value of |0.26| corresponding to an FC value of |1.2|.

**A**

***CRP>1 mg/L, responders vs. non-responders/unmedicated***


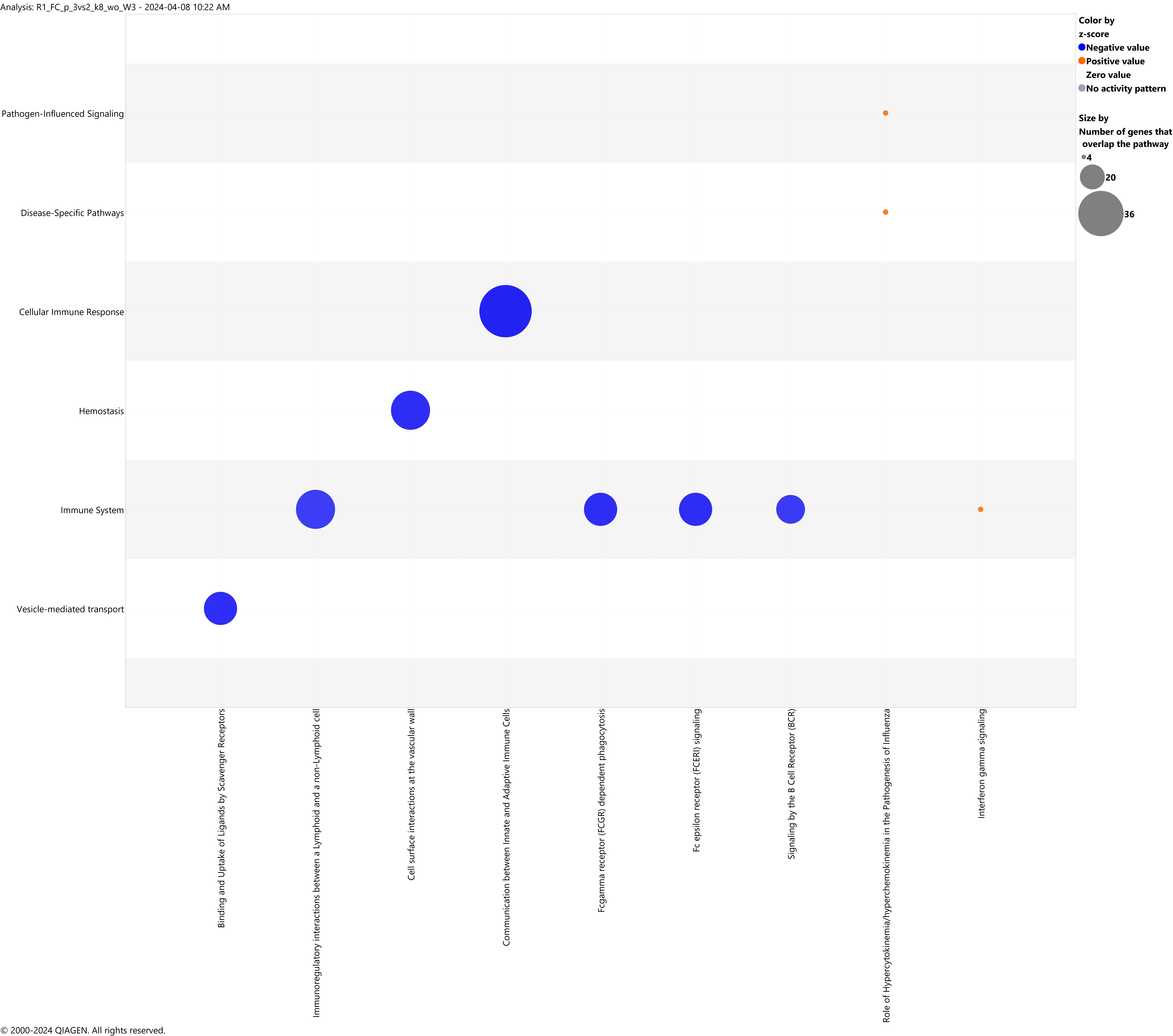


**B**

***CRP<1 mg/L, responders vs. non-responders/unmedicated***


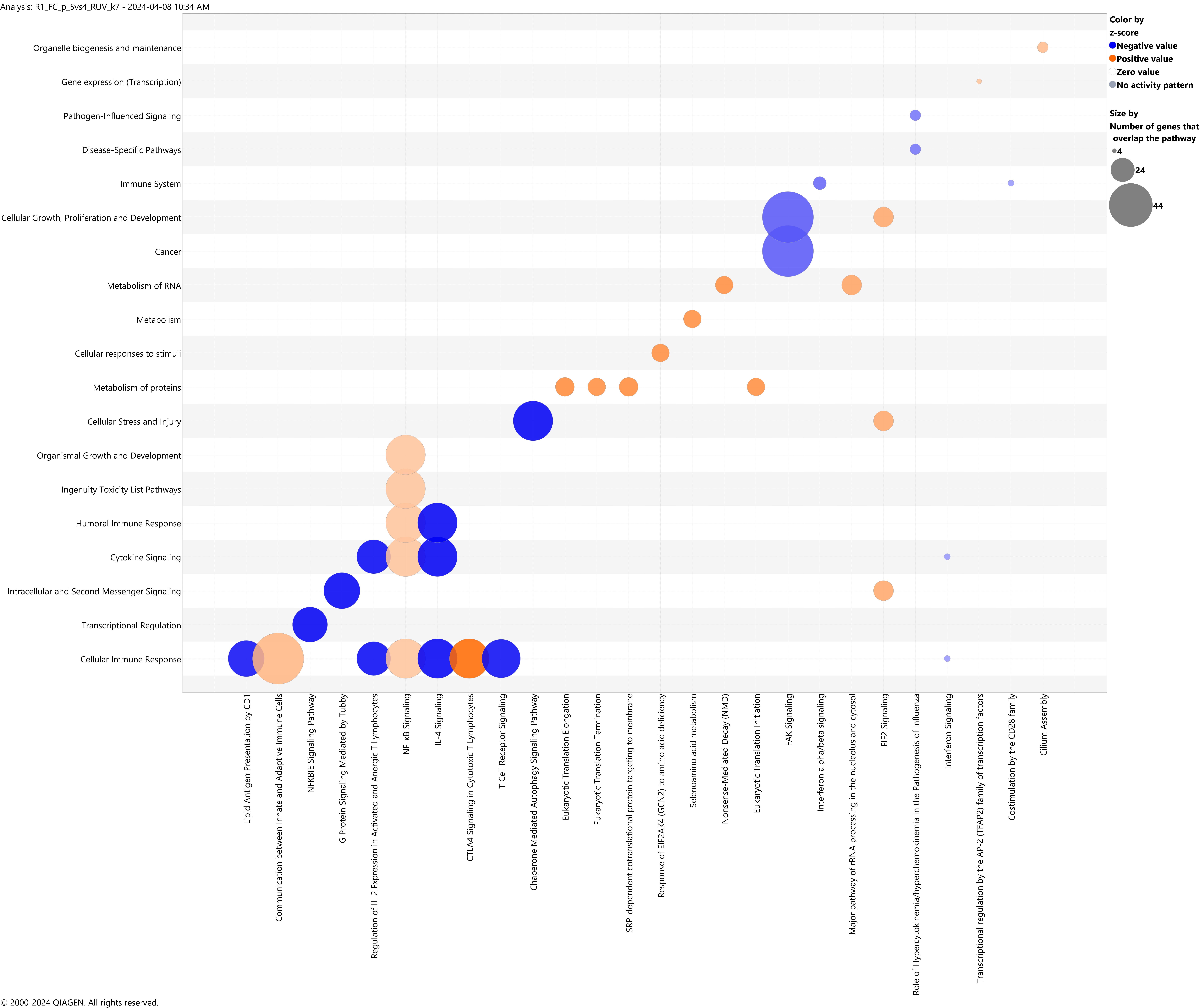


# ***Supplementary Figure S3:*** *Bubble charts of pathway clusters differentially activated in responders vs. current MDD patients (non-responders and unmedicated) selected for similar levels of CRP.*

A= CRP>1, B= CRP<1 mg/L. Orange indicates a predicted activation (positive z-scores), blue a predicted inhibition (negative z-scores); the intensity of colours reflects the z-score values. Canonical pathways (x-axis) have been selected based on p<0.05 and z-scores >|2| and clusetered into categories (y-axis). The bubble size is based on the number of genes that overlap the pathway. Generated by QIAGEN IPA.

# **References (Appendix)**

1. The World Medical Association. WMA Declaration of Helsinki – Ethical Principles for Medical Research Involving Human Subjects. 2008.

2. Williams JR. The Declaration of Helsinki and public health. Bull World Health Organ. 2008;86:650.

3. American Psychiatric Association. Diagnostic and statistical manual of mental disorders (5th ed.). 2013. 22 May 2013. https://doi.org/10.1176/APPI.BOOKS.9780890425596.

4. First MB. Structured Clinical Interview for the DSM (SCID) . The Encyclopedia of Clinical Psychology. 2015:1–6.

5. Hamilton M. A Rating Scale For Depression. J Neurol Neurosurg Psychiatry. 1960;23:56.

6. Sforzini L, Cattaneo A, Ferrari C, Turner L, Mariani N, Enache D, et al. Higher immune-related gene expression in major depression is independent of CRP levels: results from the BIODEP study. Translational Psychiatry 2023 13:1. 2023;13:1–13.

7. Cole JJ, McColl A, Shaw R, Lynall ME, Cowen PJ, de Boer P, et al. No evidence for differential gene expression in major depressive disorder PBMCs, but robust evidence of elevated biological ageing. Transl Psychiatry. 2021;11.

8. Cattaneo A, Ferrari C, Turner L, Mariani N, Enache D, Hastings C, et al. Whole-blood expression of inflammasome- and glucocorticoid-related mRNAs correctly separates treatment-resistant depressed patients from drug-free and responsive patients in the BIODEP study. Transl Psychiatry. 2020;10.

9. Chamberlain SR, Cavanagh J, De Boer P, Mondelli V, Jones DNC, Drevets WC, et al. Treatment-resistant depression and peripheral C-reactive protein. The British Journal of Psychiatry. 2019;214:11.

10. Patro R, Duggal G, Love MI, Irizarry RA, Kingsford C. Salmon provides fast and bias-aware quantification of transcript expression. Nat Methods. 2017;14:417–419.

11. Soneson C, Love MI, Robinson MD. Differential analyses for RNA-seq: transcript-level estimates improve gene-level inferences. F1000Research 2016 4:1521. 2016;4:1521.

12. Love MI, Huber W, Anders S. Moderated estimation of fold change and dispersion for RNA-seq data with DESeq2. Genome Biol. 2014;15:1–21.

13. Risso D, Ngai J, Speed TP, Dudoit S. Normalization of RNA-seq data using factor analysis of control genes or samples. Nat Biotechnol. 2014;32:896–902.

14. Benjamini Y, Hochberg Y. Controlling the False Discovery Rate: A Practical and Powerful Approach to Multiple Testing. Journal of the Royal Statistical Society: Series B (Methodological). 1995;57:289–300.

15. Thomas PD, Ebert D, Muruganujan A, Mushayahama T, Albou LP, Mi H. PANTHER: Making genome-scale phylogenetics accessible to all. Protein Science. 2022;31:8–22.
